# Selective deoxygenative alkylation of alcohols via photocatalytic domino radical fragmentations

**DOI:** 10.1038/s41467-021-25702-4

**Published:** 2021-09-10

**Authors:** Hong-Mei Guo, Xuesong Wu

**Affiliations:** Hubei Key Laboratory of Bioinorganic Chemistry & Materia Medica, School of Chemistry and Chemical Engineering, Huazhong University of Science and Technology, Wuhan, China

**Keywords:** Reactive precursors, Synthetic chemistry methodology, Photocatalysis

## Abstract

The delivery of alkyl radicals through photocatalytic deoxygenation of primary alcohols under mild conditions is a so far unmet challenge. In this report, we present a one-pot strategy for deoxygenative Giese reaction of alcohols with electron-deficient alkenes, by using xanthate salts as alcohol-activating groups for radical generation under visible-light photoredox conditions in the presence of triphenylphosphine. The convenient generation of xanthate salts and high reactivity of sequential C–S/C–O bond homolytic cleavage enable efficient deoxygenation of primary, secondary and tertiary alcohols with diverse functionality and structure to generate the corresponding alkyl radicals, including methyl radical. Moreover, chemoselective radical monodeoxygenation of diols is achieved via selective formation of xanthate salts.

## Abstract (web summary)

The generation of alkyl radicals through deoxygenation of abundant alcohols via photoredox catalysis is of interest. In this study, the authors report a one-pot strategy for visible-light-promoted photoredox coupling of alcohols with electron-deficient alkenes, assisted by carbon disulfide and triphenylphosphine.

## Introduction

**Fig. 1:**
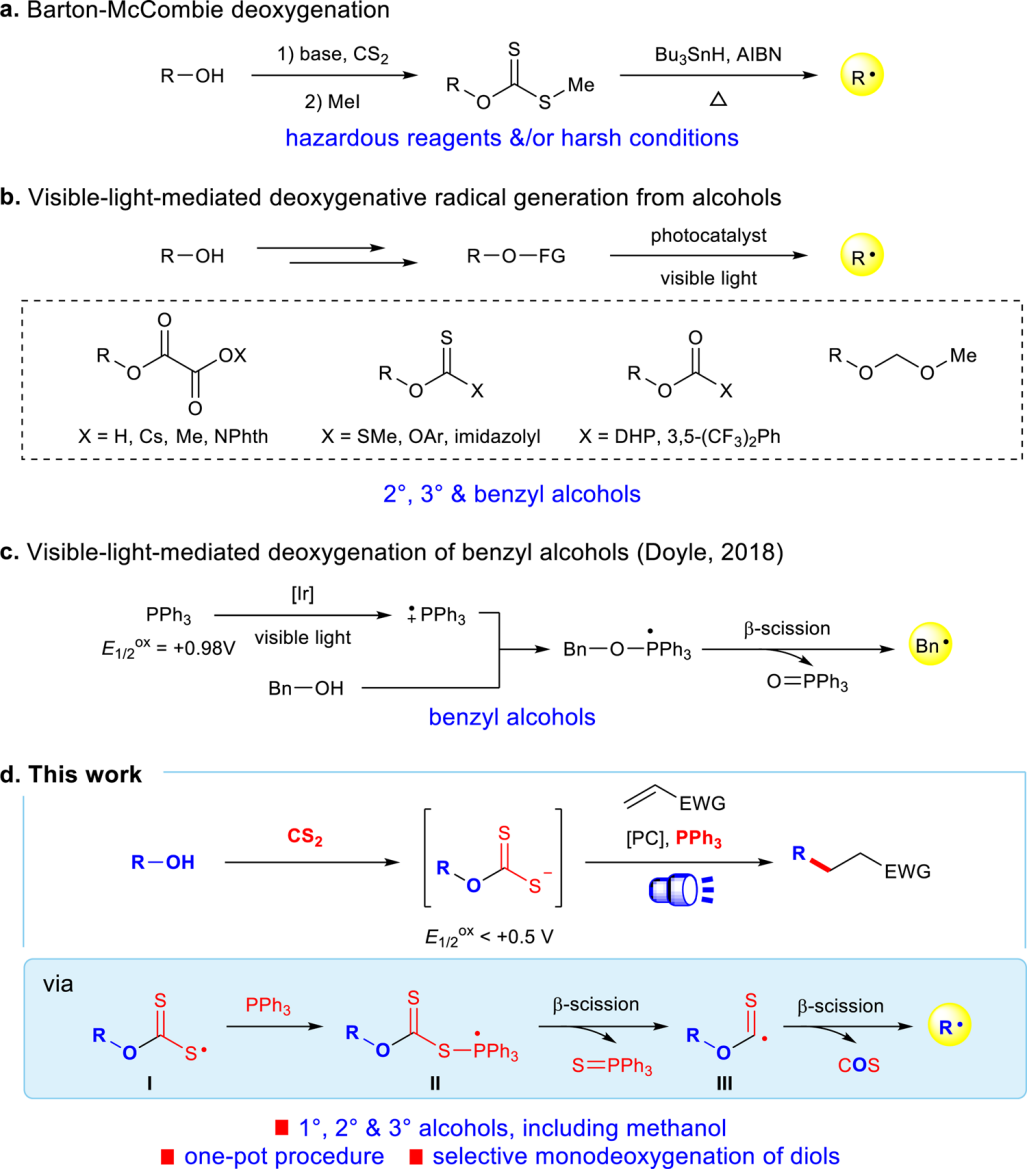
Deoxygenative alkyl radical generation from alcohols under visible-light photoredox conditions. **a** Barton–McCombie deoxygenation; **b** visible-light-mediated deoxygenative radical generation from alcohols; **c** visible-light-mediated deoxygenation of benzyl alcohols. **d** Our work. FG functional group, [Ir] [Ir(dFMeppy)_2_dtbbpy]PF_6_, PC photocatalyst, EWG electron-withdrawing group.

Over the past decades, visible-light photoredox catalysis has emerged as a pre-eminent handle for the generation of alkyl radicals via single electron transfer (SET)^1–4^. Avoiding the use of stoichiometric amounts of hazardous reagents and/or harsh reaction conditions in traditional methods such as Barton–McCombie deoxygenation (Fig. 1a)^5–8^, the delivery of alkyl radicals through deoxygenation of widely occurring, naturally abundant alcohols via photoredox catalysis is strategically appealing^9–11^. However, the strong bonding energy and the high redox potentials of C–O bonds have deterred the identification of general solutions in the field of photoredox catalysis^12,13^. To solve this problem, redox auxiliaries that include oxalates^14–23^, xanthates^19,24–27^, carboxylates^28–30^, and ethers^31^ have emerged to transform hydroxyl groups into activating groups for alkyl radical generation via C–O bond homolysis under visible-light photoredox conditions (Fig. 1b). Nevertheless, these approaches are generally not amenable to primary aliphatic alcohols. For example, oxalates derived from primary alcohols can only generate alkyl radicals at high temperatures, due to the relatively low reactivity of the intermediate alkoxycarbonyl radicals in losing carbon dioxide. Minisci-type reactions using primary alcohols, including methanol, as alkyl precursors have been achieved under photoredox conditions via α-H abstraction of alcohols and C–O bond cleavage by spin-center shift elimination of water; however, these approaches are limited to the alkylation of heteroarenes^32–34^. In 2018, Doyle and Rovis reported the elegant deoxygenative generation of alkyl radicals directly from alcohols under visible-light photoredox conditions without preactivation steps (Fig. 1c)^35^. In this catalytic system, triphenylphosphine is first oxidized to a radical cation by photoexcited *Ir(III) catalyst. The triphenylphosphine radical cation reacts ionically with an alcohol to form a phosphoranyl radical, which proceeds through β-scission to deliver an alkyl radical. However, due to the limited substrate scope of the β-scission of phosphoranyl radical^36–38^, this approach is only amenable to benzylic alcohols. To date, there is not a universally applicable photo-induced deoxygenation method for aliphatic alcohols with great selectivity and efficiency, especially one that is compatible with multiple free hydroxyl groups ubiquitously present in biological molecules^39–42^. Therefore, a more convenient and compatible protocol for deoxygenative generation of alkyl radicals from alcohols represents unmet challenge and urgent demand.

While Barton–McCombie deoxygenations with xanthate esters have been well established^5–7,43,44^, to the best of our knowledge, the direct use of xanthate salts for radical activations of alcohols has not been reported. Very recently, the strategy of visible-light photoredox-catalyzed phosphoranyl radical fragmentation^45–49^ provides a powerful platform for C–O and C–S bond homolysis of carboxylic acids^50–56^ and thiols^57–59^. Inspired by these discoveries, we proposed a masked strategy by switching the redox site from the C–O bond to the weaker and SET-amenable C–S bond (Fig. 1d). Treatment of an alcohol with carbon disulfide under basic conditions can readily generate a xanthate anion. We envisioned that the xanthate anion, with low oxidation potential, would undergo SET with an excited state photocatalyst to produce the xanthate radical **I**^60,61^. This sulfur-centered radical will subsequently couple with a phosphine source to afford the phosphoranyl radical **II**^62–65^, which then undergoes β-scission to form the alkoxythiocarbonyl radical **III**. Theoretical calculations have demonstrated that alkoxythiocarbonyl radicals **III** extrude carbonyl sulfide (COS) rapidly through β-scission to produce the corresponding alkyl radicals, including the methyl radical, in three orders of magnitude faster than the loss of carbon dioxide from alkoxycarbonyl radicals^66^. In comparison to the phosphine-mediated direct deoxygenation of alcohols developed by Doyle and co-workers (Fig. 1c)^35^, the high reactivity and low voltage gating of xanthate anion will advantage this strategy by energetically more accessible electron transfer and diverse substrate scope other than benzylic alcohols, providing a unique solution for the longstanding challenge on structure generality limitation of alcohols as alkyl radical precursors.

Here we show a visible-light-promoted photoredox coupling of alcohols with electron-deficient alkenes assisted by carbon disulfide and triphenylphosphine. This one-pot protocol enables the selective C–O bond homolysis of primary, secondary, and tertiary alcohols to form C–C bonds efficiently under mild conditions.

## Results

### Optimization of reaction conditions

To evaluate the reaction platform, we first explored the photoredox-catalyzed deoxygenative alkylation of *N*-Boc-4-hydroxy-piperidine **1aa** with benzyl acrylate **3a** by a carbon disulfide and triphenylphosphine promoted one-pot protocol. Through extensive condition optimization, we found that deprotonation of *N*-Boc-4-hydroxypiperidine **1aa** using NaO^*t*^Bu (1.0 equiv) as a base followed by treatment with carbon disulfide (1.5 equiv) in tetrahydrofuran (THF) readily formed the corresponding sodium xanthate **2aa** (Table 1). After evaporating all volatiles, exposure of a solution of the residue with benzyl acrylate **3a**, photocatalyst [Ir(ppy)_2_dtbbpy](PF_6_), and triphenylphosphine in acetonitrile containing water to a 30 W blue light-emitting diode (LED) lamp provided the desired alkylation product **4aa** in 89% isolated yield, along with triphenylphosphine sulfide in 86% yield indicating a desulfurization process. Meanwhile, the carbonyl sulfur (COS) was detected by gas chromatography–mass spectrometry (Table 1, entry 1). Triethyl phosphite could also be used as the phosphorus source in place of triphenylphosphine, providing the product in 84% yield (entry 2). Use of [Ir(dFCF_3_ppy)_2_dtbbpy)](PF_6_) as the photocatalyst resulted in a slightly less efficient reaction (entry 3). Weak bases such as Na_2_CO_3_ were not suitable for the generation of the xanthate salt (entry 4). No reaction was observed without treatment with carbon disulfide, and this observation excluded the direct activation of alcohols by triphenylphosphine under the photoredox conditions (entry 5). Further control experiments conducted in the absence of phosphine, photocatalyst, or light resulted in no product formation, emphasizing the crucial role of all these components in the catalytic cycle (entries 6–8). Significantly lower yield was observed in the absence of water, perhaps due to its role as the proton source (entry 9). Reaction with 1.0 equiv of benzyl acrylate **3a** provided the product in 66% yield (entry 10). Reducing the amount of triphenylphosphine to 0.20 equiv decreased the yield to 20%, correspondingly excluding a triphenylphosphine-initiated/catalyzed pathway (entry 11). In addition, our attempts on using 0.20 equiv of triphenylphosphine combined with stoichiometric reductants failed to increase the yield to exceed 20% (Supplementary Table 5).

**Table 1:** Optimization and control studies.


Entry	Variation from optimized conditions	Yield (%)^a^
1	None	92 (89)^b^
2	P(OEt)_3_ instead of PPh_3_	84
3	[Ir(dFCF_3_ppy)_2_dtbbpy)](PF_6_) (1 mol %)	83
4	Na_2_CO_3_ instead of NaO^*t*^Bu	0
5	No CS_2_	0
6	No PPh_3_	0
7	No photocatalyst	0
8	No light	0
9	Without H_2_O	26
10	**3a** (1.0 equiv)	66
11	PPh_3_ (0.20 equiv)	20
12	PPh_3_ (0.20 equiv) + reductants (2.0 equiv)	<20

Standard procedure: **1aa** (0.20 mmol), NaO^*t*^Bu (1.0 equiv), THF (1.2 mL), CS_2_ (1.5 equiv), 3 h. After removing solvent in vacuo, then **3a** (2.0 equiv), [Ir(ppy)_2_dtbbpy](PF_6_) (1 mol%), PPh_3_ (1.2 equiv), H_2_O (7.0 equiv), MeCN (2.0 mL), 24 h blue LED irradiation.

Boc *t*-butoxycarbonyl.

^a^Determined by ^1^H NMR analysis using 1,3,5-trimethoxybenzene as an internal standard.

^b^Isolated yield.

### Substrate scope

**Fig. 2:**
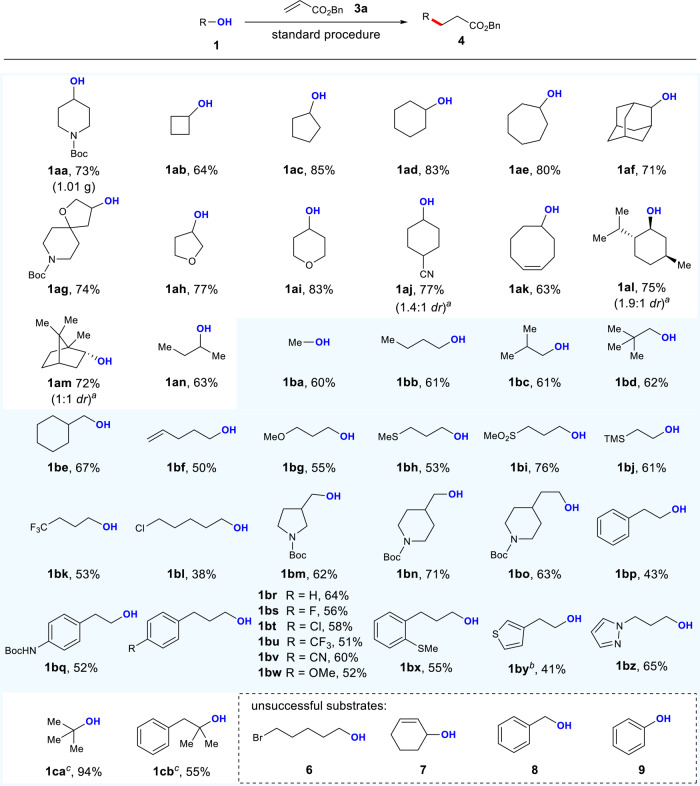
Scope of alcohols. The standard procedure is as shown in Table 1. Isolated yields are reported. ^a^The *dr* was determined by ^1^H NMR analysis of the product after flash chromatography. ^b^48 h. ^c^NaH (1.0 equiv) as base. *dr* diastereoselectivity ratio.

With the optimized conditions in hand, we investigated the scope of alcohols using this convenient one-pot protocol (Fig. 2). It is noteworthy that the reaction could be run on a 4.0 mmol scale in a Schlenk tube to afford 1.01 g of the desired product **4aa** (73% yield). Cyclic alcohols (**1ab**–**aj**), ranging from four- to eight-membered rings, including spirocyclic or bridged motifs, adorned with nitrogen or oxygen atoms, successfully delivered the corresponding products in 64–85% yields upon Giese reaction with benzyl acrylate **3a**. The alkene-retained product was obtained in 63% yield from cyclooct-4-en-1-ol **1ak**, while intramolecular radical cyclization was not observed. This result suggests that the Giese addition of alkyl radical is faster than the 5-*exo*-trig cyclization (~1 × 10^5^ s^−1^) under the reaction conditions^67^. It is noteworthy that natural products L-menthol **1al** and (-)-borneol **1am** reacted smoothly in this transformation, providing the deoxy-alkylated products in 75% and 72% yields, respectively. The linear secondary alcohol **1an** also provided the desired adduct in 63% yield. Consistent with the expectation, we were pleased to find that a wide variety of primary alcohols (**1ba**–**bz**) including methanol **1ba** were successfully applied in this protocol, furnishing the desired products in moderate-to-good yields. Functional groups such as terminal alkenes, ethers, thioethers, sulfones, silanes, trifluoromethyl, chlorides, fluorides, nitriles, Boc-protected amines, phenyls, and heteroarenes were well tolerated, with the deoxygenative alkylated products obtained in 38–76% yields. Furthermore, by using NaH as a base for sodium xanthate construction, tertiary alcohols (**1ca**, **1cb**) could also be efficiently converted to the corresponding products. In terms of limitations, the current protocol is not applicable to alcohols containing highly electrophilic groups, like alkyl bromides **6**. Unfortunately, neither allylic alcohols nor benzylic alcohols were competent substrates (**7**, **8**). Deviating from radical conjugate addition, deoxygenation products cyclohexene and toluene were observed in these cases, respectively. Finally, using this protocol, the deoxygenative alkylation of phenol **9** via Csp^2^–O bond cleavage was not successful^68–73^.

**Fig. 3:**
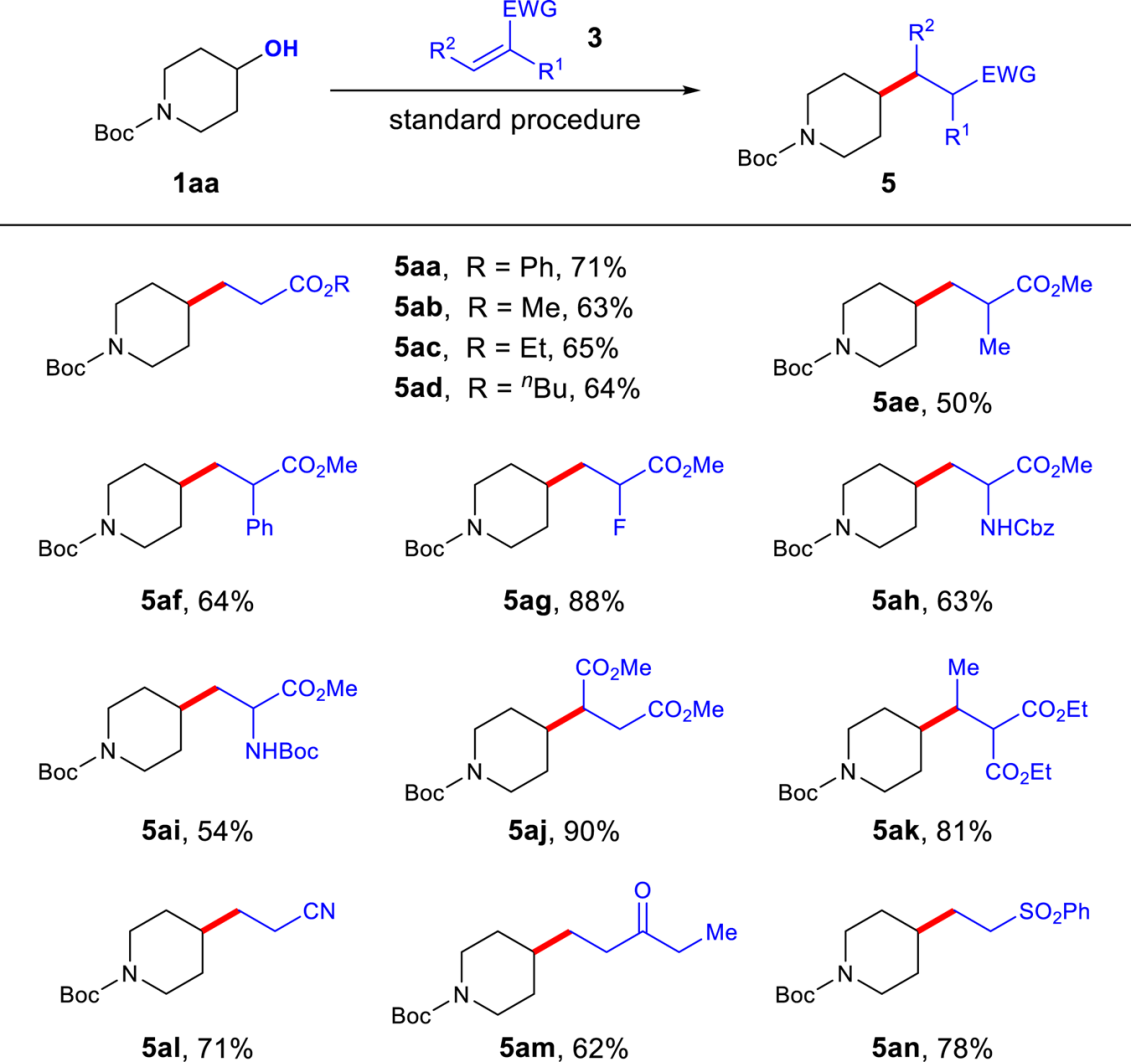
Scope of alkenes. The standard procedure is as shown in Table 1. Isolated yields are reported. Cbz benzyloxycarbonyl.

To further demonstrate the versatility of this method, a range of electron-deficient alkenes was tested (Fig. 3). As expected, various acrylates were capable acceptors in the reaction (**5aa**–**ad**). The reactions of *α*-aryl, *α*-fluoro, or *α*-alkyl substituted acrylates afforded desired products in 50–88% yields (**5ae**–**ag**). Notably, dehydroalanine derivatives participated in the reaction with good efficiency, providing convenient access to unnatural amino acids (**5ah**, 63%; **5ai**, 54%). Substitution at the β-position was tolerated for more electron-deficient alkenes such as maleate and dimethyl ethylidenemalonate, furnishing the expected adducts with high efficiency (**5aj**, 90%; **5ak**, 81%). Other electron-deficient alkenes, such as acrylonitrile, enone, and vinyl sulfone, all worked well (**5al**–**an**).

**Fig. 4:**
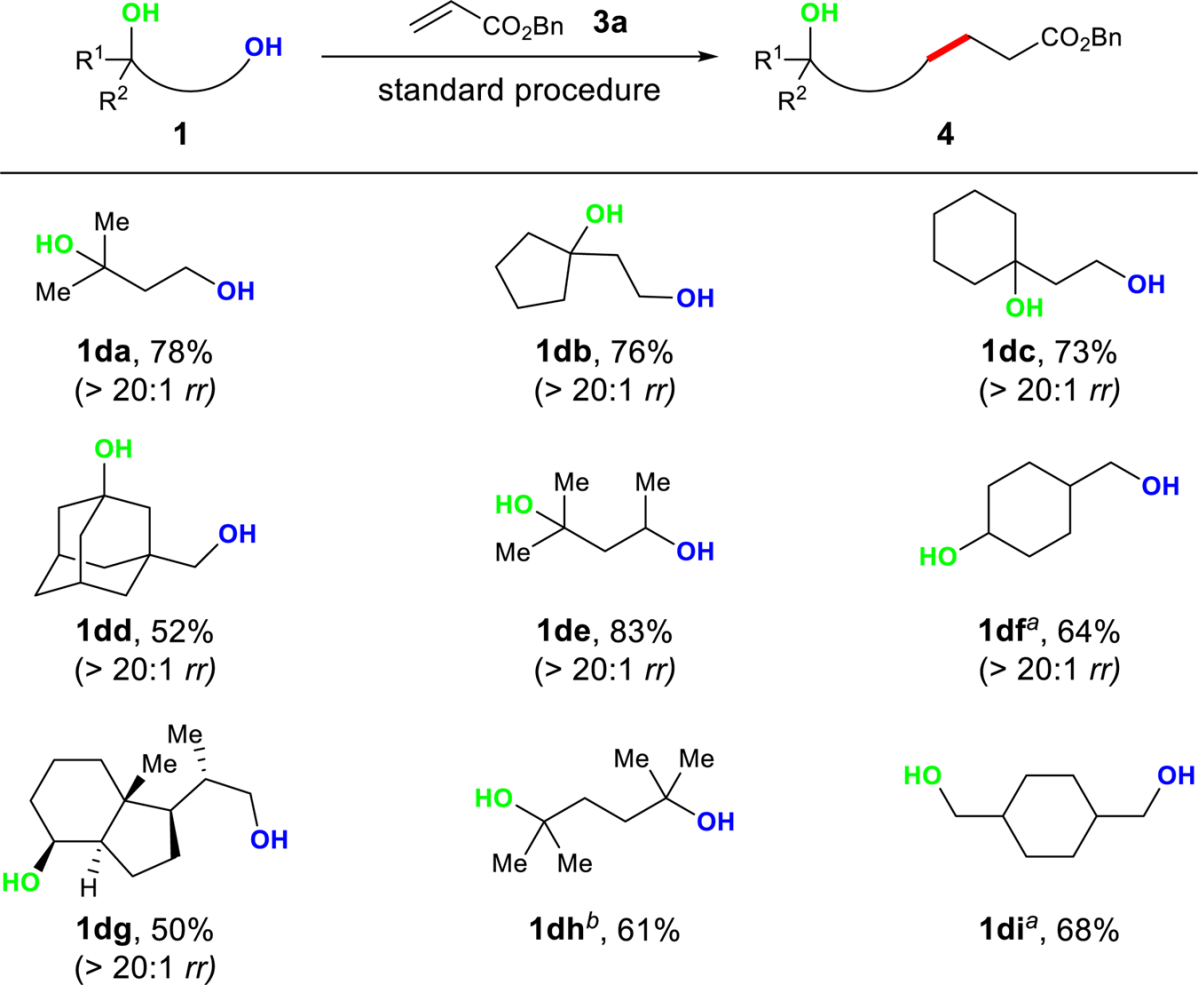
Selective deoxygenative alkylation of diols. The standard procedure is as shown in Table 1. Isolated yields are reported. ^a^48 h. ^b^NaH as base. Unreacted hydroxyl groups are marked in green. *rr* regioselectivity ratio.

Polyols are a ubiquitous structural motif widely found in natural products and pharmaceuticals^74^. The selective transformation of hydroxyl groups in diols and polyols is a frequently encountered challenge^42,75–80^. We hypothesized that a proper base could identify primary, secondary, and tertiary alcohols in diols and polyols based on difference in acidity and steric hindrance, to form the corresponding xanthate salts selectively and realize the chemoselective radical O–H bond activation of diols and polyols. To our delight, by using NaO^*t*^Bu as a base, high selectivity was observed when different diols were employed in the deoxygenative alkylation protocol, which can be summarized in decreasing order of reactivity as follows: primary alcohol > secondary alcohol > tertiary alcohol (Fig. 4). Selective deoxygenation of primary or secondary alcohol in diols (**1da**–**de**) over tertiary alcohol was achieved by the one-pot protocol to afford the corresponding products with free hydroxyl groups in 52–83% yields and >20:1 chemoselectivity. In addition, the deoxygenative transformation showed a predominant preference (>20:1 chemoselectivity) for primary alcohols over secondary ones (**1df**, **1dg**). Mono-deoxylated products were obtained with the symmetrical diols (**1dh**, **1di**). It is worth mentioning that no dialkylation products from these diols were observed under the standard conditions or with double the amount of other components in the protocol. Using 2.0 equiv of NaO^*t*^Bu resulted in decreased yields of the desired products, while the deoxygenative alkylated compound from NaO^*t*^Bu was formed.

**Fig. 5:**
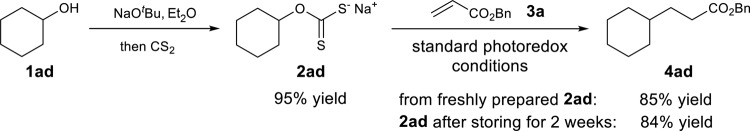
Preparation and reaction of xanthate salts. Deoxygenative alkylation of alcohols performed in steps.

In addition to the one-pot procedure, the deoxygenative alkylation of alcohols can also be performed in steps (Fig. 5). Sodium cyclohexylxanthate **2ad** was easily isolated as a pale yellow solid in 95% yield from the reaction mixture at the first step by filtration. Although xanthate salt is hygroscopic, it can be stored for extended periods in a capped container at room temperature without inert gas protection. Compared with freshly prepared salt (85%), the reaction of xanthate salt **2ad** after storing for 2 weeks provided the deoxygenative alkylated product with no detriment to yield (84%).

### Synthetic applications

**Fig. 6:**
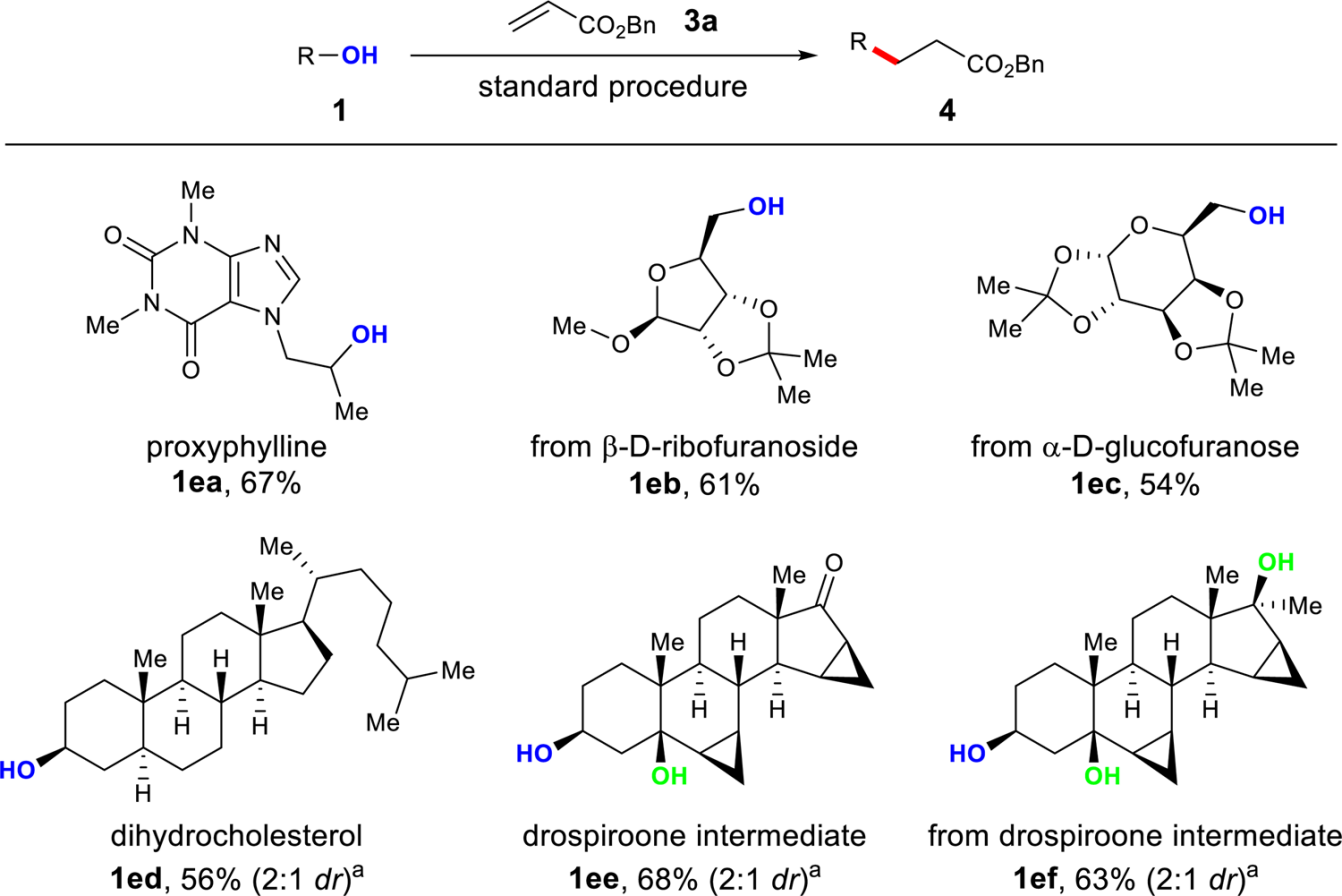
Synthetic applications of the methodology. The standard procedure is as shown in Table 1. Isolated yields are reported. ^a^The *dr* was determined by ^1^H NMR analysis of the product after flash chromatography.

The generality of this method implies applications in the late-stage functionalization of pharmaceuticals and natural products (Fig. 6)^81^. Bronchodilator proxyphylline **1ea** showed good reactivity to deliver the product in 67% yield. Acetonide D-ribofuranoside and D-glucofuranose derivatives (**1eb**, **1ec**) were deoxygenated to afford the corresponding alkylated compounds in 61% and 54% yields, respectively. Naturally occurring steroids (**1ed**–**ef**) were successfully employed, furnishing the desired products in synthetically useful yields. The remarkable selectivity of the reaction was again showcased in alkaloids **1ee** and **1ef** with multiple hydroxyl groups by the selective deoxy-alkylation of secondary alcohol over tertiary alcohols.

### Mechanistic studies

**Fig. 7:**
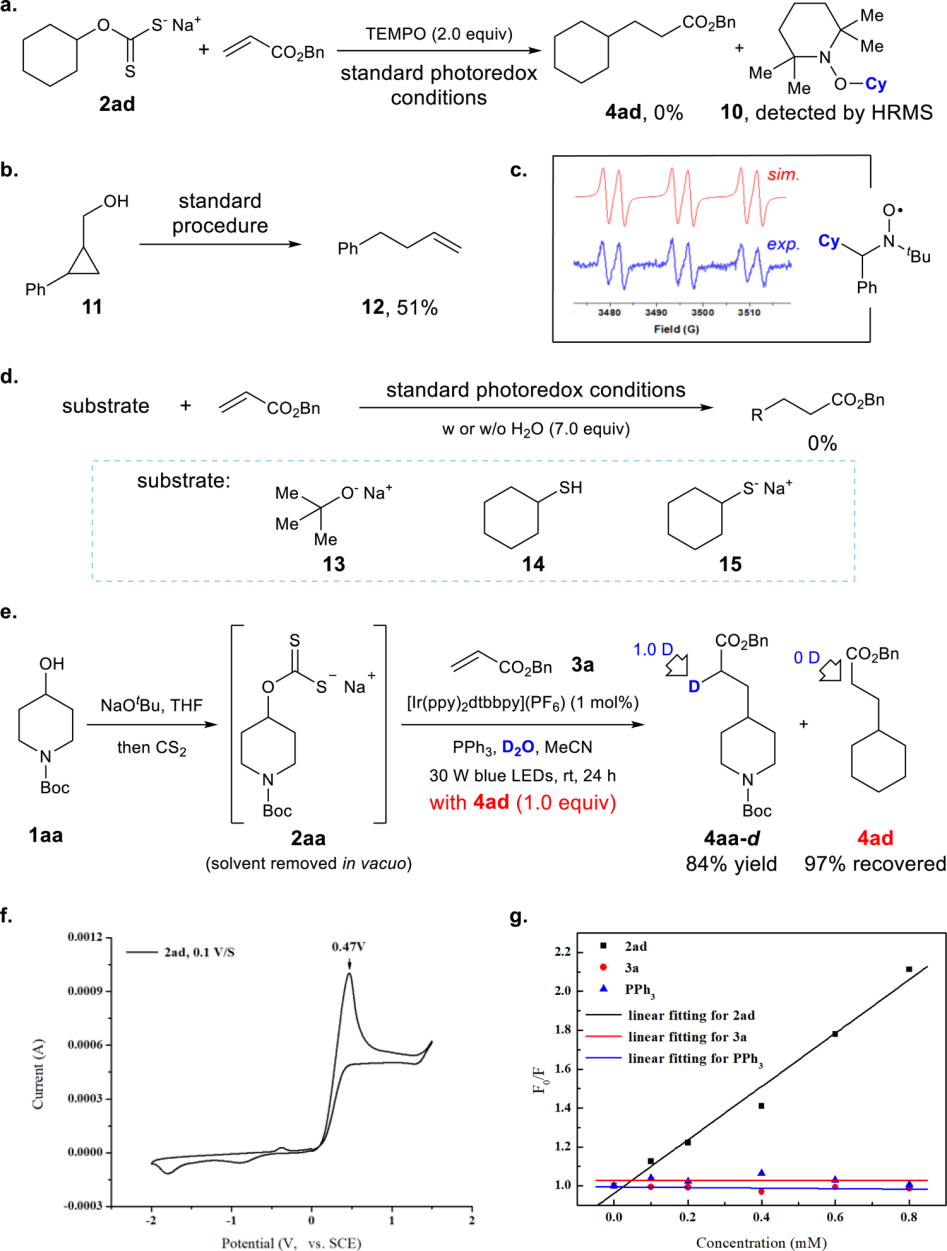
Probing the mechanism. **a** Radical trap experiment; **b** radical clock experiment; **c** EPR experiment (*g* = 2.00619, *A*_N_ = 14.9123 G, *A*_H_ = 3.41209 G); **d** probing the intermediate; **e** deuterium-labeling experiment; **f** cyclic voltammogram; **g** Stern–Volmer fluorescence quenching experiment.

**Fig. 8:**
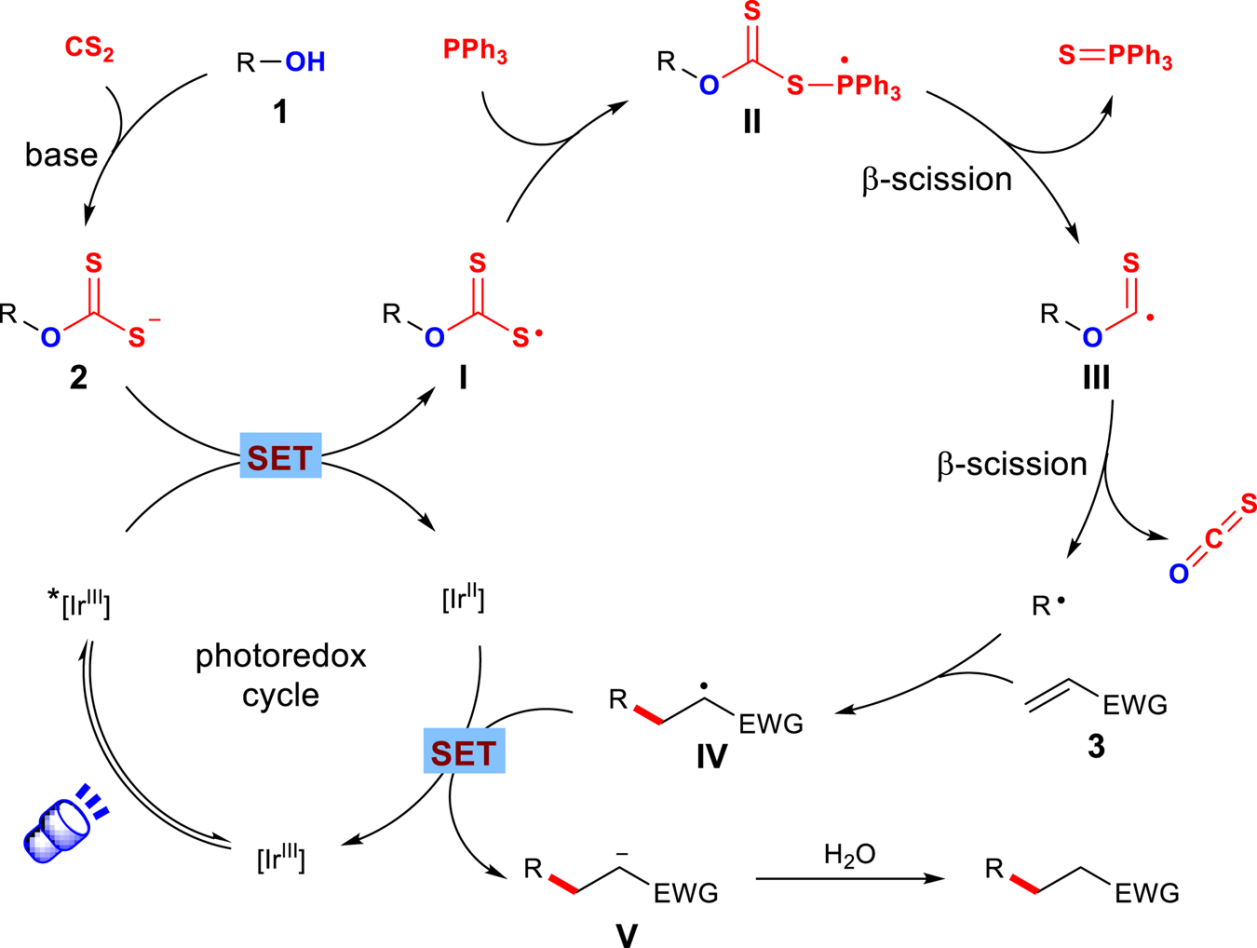
Proposed reaction mechanism. SET single-electron transfer, [Ir] [Ir(ppy)_2_dtbbpy](PF_6_).

To gain insight into the possible reaction pathway, a set of preliminary mechanistic studies have been carried out^82^. The standard photoredox catalysis was completely inhibited in the presence of a stoichiometric amount of TEMPO, and the TEMPO-trapped cyclohexyl radical could be identified by high-resolution mass spectrometry (Fig. 7a). Ring opening was observed when cyclopropanemethanol **11** was employed, consistent with a radical intermediate (Fig. 7b). Next, electron paramagnetic resonance experiments were carried out with sodium cyclohexylxanthate **2ad** by utilizing PBN as radical scavenger (Fig. 7c). It provided a signal that was consistent with that of cyclohexyl radical according to the literature data^83^. These results provide support for the radical nature of the deoxygenation process. When sodium alkoxide **13** was directly employed in the photoredox catalysis step, no reaction was observed, excluding the direct deoxygenation of alcohols with triphenylphosphine (Fig. 7d). No reaction was observed with thiol **14** or sodium thiol **15**, indicating that O, S-rearrangement^84^ was not involved in the reaction pathway. When replacing water with deuterium oxide, the reaction of alcohol **1aa** in the presence of ester **4ad** (1.0 equiv) led to complete deuterium incorporation into the α-position of the corresponding product **4aa-*d*** (Fig. 7e). Meanwhile, no deuterium incorporation was observed in recovered **4ad**, indicating that no H/D exchange between the ester product and deuterium oxide via keto–enol tautomerism occurred under the reaction conditions. These results support the intermediacy of α-acyl carbon anion **V** in the catalytic cycle (Fig. 8). Oxidation potential of xanthate salt **2ad** (*E*_1/2_^ox^ = +0.47 V vs saturated calomel electrode (SCE) in MeCN) measured by cyclic voltammogram shows that it is significantly easier to oxidize compared with triphenylphosphine (*E*_1/2_^ox^ = +0.98 V vs SCE)^85^ (Fig. 7f).

Stern–Volmer fluorescence quenching experiments verified that the excited state *Ir(III) was quenched effectively by xanthate anion but not by triphenylphosphine or the alkene (Fig. 7g). “Light–dark” experiments confirmed that the reaction required continuous light irradiation. This result and the quantum yield (*Φ* = 0.124) of the reaction suggest that a radical chain process, based on either a HAT process of an alkyl radical with a xanthate acid or an SET from xanthate anion to intermediate **IV**, is unlikely.

On the basis of the above experiments and precedent reports, a plausible mechanism was proposed (Fig. 8). Deprotonation of alcohols followed by the reaction with carbon disulfide generates the corresponding xanthate salt **2**. The photoexcited iridium catalyst (*E*_1/2_^ox^[*Ir(III)/Ir(II)] = +0.66 V vs SCE in MeCN)^86^ oxidizes the xanthate anion **2** via SET to form the sulfur-centered radical **I**. The xanthate radical **I** couples with the phosphine to afford the phosphoranyl radical **II** and undergoes β-scission to form alkoxythiocarbonyl radical **III**. Fragmentation of the alkoxythiocarbonyl radical **III** extrudes carbonyl sulfide and produces the corresponding alkyl radical, which subsequently undergoes radical conjugate addition on the acceptor. Reduction of the resulting adduct radical **IV** (*E*_1/2_^red^ = −0.59 to −0.73 V vs SCE in MeCN)^87^ by SET from the Ir(II) species (*E*_1/2_^red^[Ir(III)/Ir(II)] = −1.51 V vs SCE in MeCN)^86^ yields the product after protonation and regenerates the ground-state photocatalyst, completing the catalytic cycle.

## Discussion

In conclusion, we have developed a visible-light photoredox-catalyzed method for the deoxygenative generation of alkyl radicals from various primary, secondary, and tertiary alcohols enabled by inexpensive carbon disulfide and triphenylphosphine-assisted C–O bond activation via xanthate salt intermediates. A broad range of alkyl groups from alcohols can be efficiently incorporated into various electron-deficient alkenes, and this easily handled one-pot protocol is scalable to the gram level. The two-step sequence is highly chemoselective, demonstrated by the monodeoxygenation of diols and steroids with multiple hydroxyl groups. This method features mild conditions, broad substrate scope, and good functional group tolerance, which can be further applied in the late-stage modification of biologically important complex molecules. The generality of this methodology, as well as the ready availability of the starting materials and reagents, will allow it to become a useful synthetic tool and enjoy extensive use in a range of chemical applications. Further synthetic applications of xanthate salts as alkyl radical precursors in photo-mediated radical processes are underway in our laboratory and will be reported in due course.

## Methods

### General procedure for the synthesis of products

In a nitrogen-filled glovebox, an oven-dried 5 mL quartz tube equipped with a magnetic stir bar was charged sequentially with alcohol **1** (0.20 mmol) and NaO^*t*^Bu (19.2 mg, 0.20 mmol), followed by the addition of dry THF (1.2 mL). The quartz tube was sealed with a septum cap and transferred out of the glovebox, then stirred at room temperature for 30 min. The resulting solution was cooled to 0 °C followed by addition of CS_2_ (22.8 mg, 19.0 µL, 0.30 mmol) via microsyringe, then stirred at 0 °C for 3 h before removing the solvent in vacuo. The system was transferred into the glovebox, and PPh_3_ (63.0 mg, 0.24 mmol), [Ir(ppy)_2_dtbbpy](PF_6_) (1.8 mg, 1.0 mol%), alkene **3** (0.40 mmol), and H_2_O (25.2 mg, 1.4 mmol) in MeCN (2.0 mL) was added. Then the quartz tube was sealed with a septum cap and transferred out of the glovebox. The reaction mixture was irradiated with a 30 W blue LED lamp, maintained at a temperature of 26 °C, and stirred for 24 h. After removing the solvent in vacuo, the residue was purified by column chromatography to afford the product. The reaction could also be performed under nitrogen using a Schlenk tube, without the need of a glovebox (see Section 4 in Supplementary Information for details).

## Supplementary information

Supplementary Information

## Data Availability

The authors declare that the data supporting the findings of this study, including experimental details and compound characterization, are available within the article and its Supplementary Information file. All data are available from the corresponding author upon reasonable request.
