# Supplementary material for: Selective deoxygenative alkylation of alcohols via photocatalytic domino radical fragmentations
Source: Nat Commun. 2021 Sep 10;12:5365. doi: 10.1038/s41467-021-25702-4 (PMC8433232; doi:10.1038/s41467-021-25702-4)
Supplement: Supplementary file 1 — Supplementary Information [file 41467_2021_25702_MOESM1_ESM.pdf]

Supplementary Information

Selective Deoxygenative Alkylation of Alcohols via Photocatalytic Domino Radical Fragmentations

Hong-Mei Guo[1] and Xuesong Wu[1*]

[1]Hubei Key Laboratory of Bioinorganic Chemistry & Materia Medica, School of Chemistry and Chemical Engineering, Huazhong University of Science and Technology, Wuhan 430074, China.

Table of Contents

1. General Information **S2**

2. Detailed Optimization of Reaction Conditions **S3**

2.1 Optimization of Reaction Conditions S4

2.2 Control Experiments S6

3. General Procedure and Spectral Data of Products **S7**

3.1 Procedure for Synthesis of 2ad S7

3.2 General Procedure for Deoxygenative Alkylation of Alcohols S7

3.3 Spectral Data of Products S8

4. Gram-Scale Reaction **S29**

4.1 Procedure for Gram-Scale Reaction S29

5. Synthetic Application of the Reaction **S30**

5.1 Preparation of compound 1ef S30

5.2 Synthetic applications of the methodology S30

5.3 Spectral Data S31

6. Mechanism Studies **S35**

6.1 Luminescence Quenching Experiments S35

6.2 Light On-Off Experiments S36

6.3 Determination of Quantum Yields S36

6.4 TEMPO Trapping Experiment S37

6.5 Radical Clock Experiment S38

6.6 Electrochemical Studies S39

6.7 EPR Experiment S40

6.8 Deuterium-Labeling Experiment S41

6.9 Carbonyl Sulfide (COS) Detecting Experiment S42

7. NMR Spectra **S44**

8. Supplementary References **S125**

1. General Information

Commercially Reagents: Commercially reagents were purchased from Sigma Aldrich, Energy Chemical, TCI or Alfa Aesar and used without further purification. All experiments were performed in oven-dried or flame-dried glassware under an atmosphere of N_2. Tetrahydrofuran, acetonitrile were ultra-dry solvents with MS purchased from Energy Chemical and stored within a N_2 filled glove box.

NMR Spectra: ^{1}H NMR spectra were recorded on a 400 or 600 MHz spectrometer. Chemical shifts are reported in parts per million (ppm) and the spectra are calibrated to the resonance resulting from incomplete deuteration of the solvent ($CDCl_3$: 7.26 ppm). ^{13}C NMR spectra were recorded on the same spectrometer with complete proton decoupling. Chemical shifts are reported in ppm with the solvent resonance as the internal standard ($CDCl_3$: 77.16 ppm, t). Data are reported as follows: chemical shift δ/ppm, integration (^{1}H only), multiplicity (s = singlet, d = doublet, t = triplet, q = quartet, m = multiplet or combinations thereof; ^{13}C signals are singlets unless otherwise stated), coupling constants *J* in Hz, assignment. ^{19}F NMR spectra and ^{31}P NMR spectra were recorded on the same spectrometer.

Gas Chromatograph-Mass Spectrometer (GC-MS): All GC-MS were recorded on Agilent 5977B-7890B. Measured values are reported to 3 decimal places of the calculated value. The calculated values are based on the most abundant isotope.

Gas Chromatograph (GC): All GC were recorded on Fuli GC9790II.

Infra-Red Spectrometer (IR): All IR were recorded on Bruker INVENIO-R.

Chromatography: Analytical thin layer chromatography was performed using Qingdao Puke Parting Materials Co. silica gel plates (Silicagel 60 F254). Visualisation was by ultraviolet fluorescence (λ = 254 nm) and/or staining with phosphomolybdic acid or potassium permanganate ($KMnO_4$). Flash column chromatography was performed using 200-300 mesh silica gel.

UV/Vis: Measurements were made on Shanghai JiaPeng technology co. ZF-7 Spectro Fluorophotometer.

Photoreactor: The photoreactors used in this research were purchased from Taobao (Supplementary Figure 1: 30W blue LEDs).

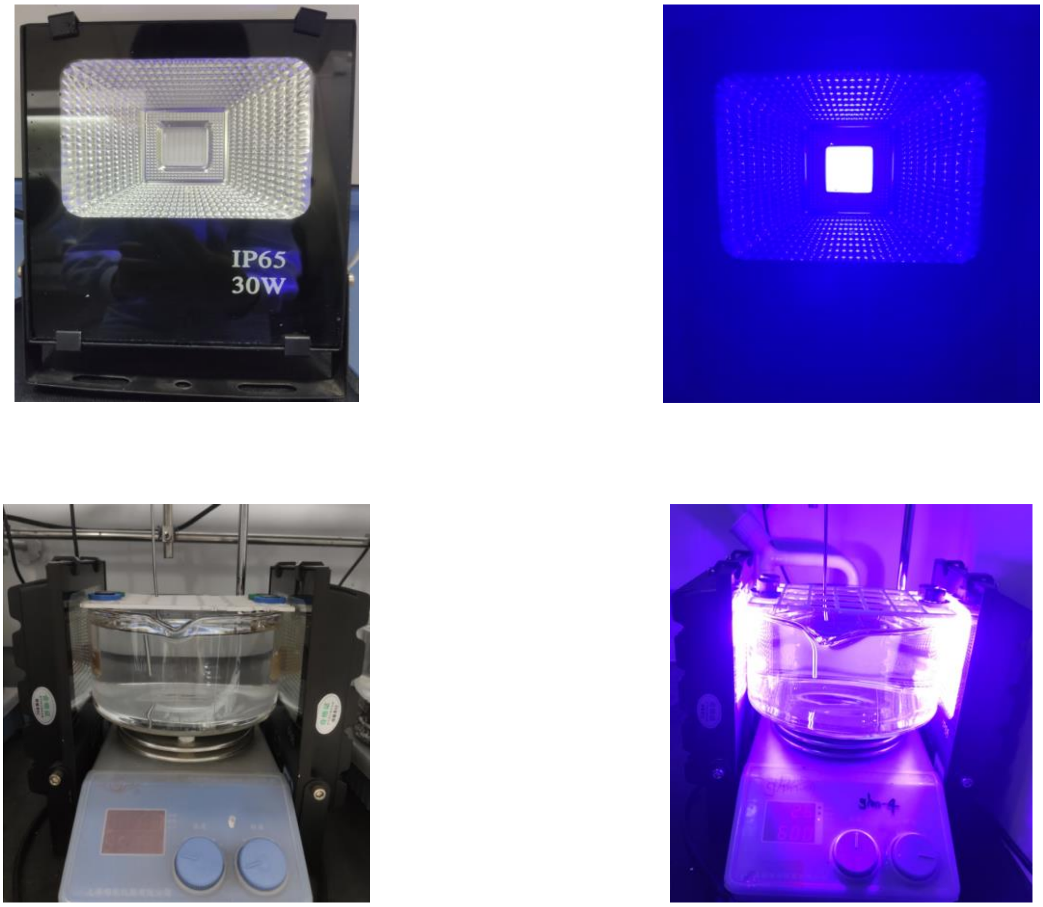

Supplementary Figure 1. Photoreactor used in this research (30 W blue LEDs)

2. Detailed Optimization of Reaction Conditions

2.1 Optimization of Reaction Conditions

Supplementary Table 1. Screening of Bases[a]

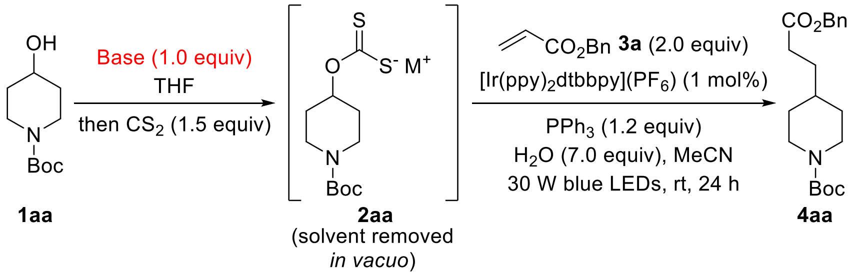

Entry	Base	Yield (%)[b]
1	Na_2CO_3	0
2	Cs_2CO_3	0
3	NaOH	45
4	KOH	52
5	NaH	29
6	KO^{t}Bu	84
7	NaO^{t}Bu	92
8	K_3PO_4	43

[a]Standard procedure: **1aa** (0.2 mmol), base (1.0 equiv), THF (1.2 mL), CS_2 (1.5 equiv), 3h. After removing solvent *in vacuo*, then **3a** (2.0 equiv), [Ir(ppy)$_2$dtbbpy](PF$_6$) (1 mol%), PPh_3 (1.2 equiv), H_2O (7.0 equiv), MeCN (2.0 mL), 24h. [b]Determined by ^{1}H NMR analysis with 1,3,5-trimethoxybenzene as an internal standard.

Supplementary Table 2. Screening of Solvents[a]

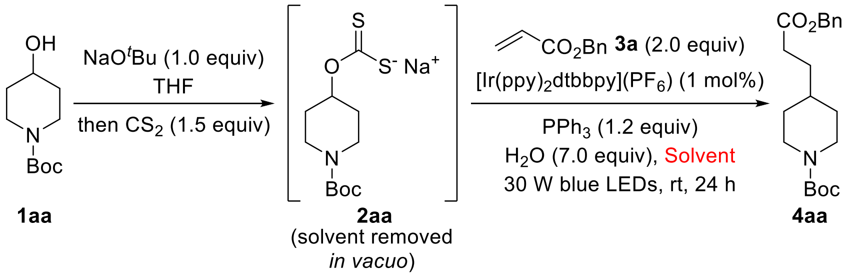

Entry	Solvent	Yield (%)[b]
1	MeCN	92
2	THF	56
3	DCM	73
4	DCE	78
5	DMF	74
6	Dioxane	51
7	$CHCl_3$	21

[a]Standard procedure: **1aa** (0.2 mmol), NaO^{t}Bu (1.0 equiv), THF (1.2 mL), CS_2 (1.5 equiv), 3h. After removing solvent *in vacuo*, then **3a** (2.0 equiv), [Ir(ppy)$_2$dtbbpy](PF$_6$) (1 mol%), PPh_3 (1.2 equiv), H_2O (7.0 equiv), solvent (2.0 mL), 24h. [b]Determined by ^{1}H NMR analysis with 1,3,5-trimethoxybenzene as an internal standard.

Supplementary Table 3. Screening of Photocatalyst[a]

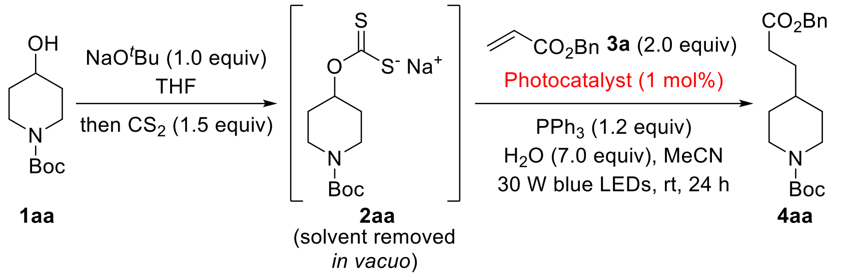

Entry	Photocatalyst	Yield (%)[b]
1	[Ir(dFCF$_3$ppy)$_2$dtbbpy)](PF$_6$)	83
2	[Ir(ppy)$_2$dtbbpy](PF$_6$)	92
3	*fac*-Ir(ppy)$_3$	0
4	Ru(bpy)$_3$Cl$_2$	18
5	[Mes$^-$ Acr$^+$](ClO$_4$)	0
6	Rhodamin B	0

[a]Standard procedure: **1aa** (0.2 mmol), NaO^{t}Bu (1.0 equiv), THF (1.2 mL), CS$_2$ (1.5 equiv), 3h. After removing solvent *in vacuo*, then **3a** (2.0 equiv), photocatalyst (1 mol%), PPh$_3$ (1.2 equiv), H$_2$O (7.0 equiv), MeCN (2.0 mL), 24h. [b]Determined by ^{1}H NMR analysis with 1,3,5-trimethoxybenzene as an internal standard.

Supplementary Table 4. Amount of H$_2$O[a]

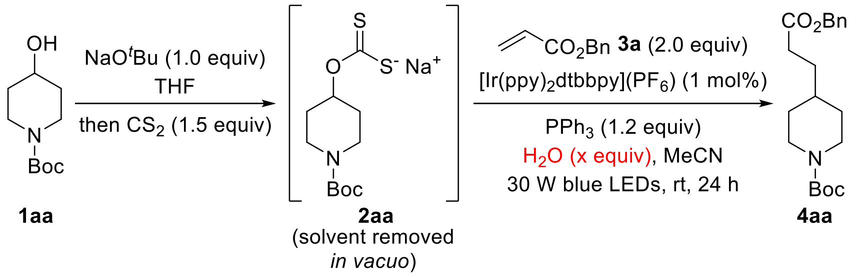

Entry	H$_2$O (x equiv)	Yield (%)[b]
1	5.0	85
2	6.0	87
3	7.0	92
4	8.0	87
5	9.0	88
6	no	26

[a]Standard procedure: **1aa** (0.2 mmol), NaO^{t}Bu (1.0 equiv), THF (1.2 mL), CS$_2$ (1.5 equiv), 3h. After removing solvent *in vacuo*, then **3a** (2.0 equiv), [Ir(ppy)$_2$dtbbpy](PF$_6$) (1 mol%), PPh$_3$ (1.2 equiv), H$_2$O (x equiv), MeCN (2.0 mL), 24h. [b]Determined by ^{1}H NMR analysis with 1,3,5-trimethoxybenzene as an internal standard.

Supplementary Table 5. Screening of reductants[a]

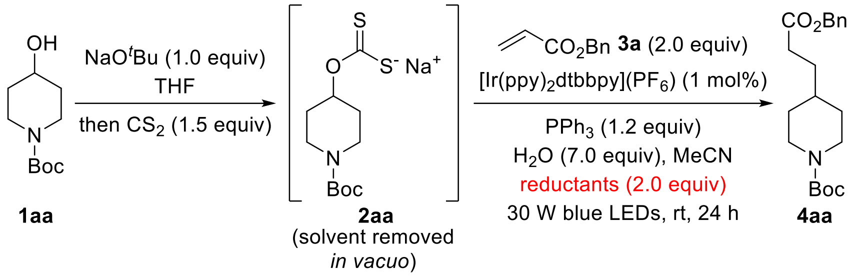

Entry	reductants	Yield (%)[b]
1	none	20
2	Zn	13
3	Fe	11
4	$PhSiH_3$	14
5	Et_3SiH	9
6	$(TMS)_3SiH$	7
7	Cp_2ZrHCl	0

[a]Standard procedure: **1aa** (0.2 mmol), NaO^{t}Bu (1.0 equiv), THF (1.2 mL), CS_2 (1.5 equiv), 3h. After removing solvent *in vacuo*, then **3a** (2.0 equiv), [Ir(ppy)$_2$dtbbpy](PF_6) (1 mol%), PPh_3 (1.2 equiv), reductants (2.0 equiv) H_2O (7.0 equiv), MeCN (2.0 mL), 24h. [b]Determined by ^{1}H NMR analysis with 1,3,5-trimethoxybenzene as an internal standard.

2.2 Control Experiments

Supplementary Table 6. Control Experiments[a]

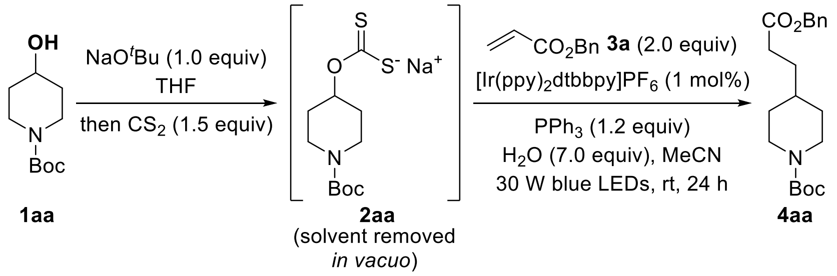

Entry	NaO^{t}Bu	CS_2	[Ir(ppy)$_2$dtbbpy](PF_6)	*hv*	PPh_3	Yield (%)[b]
1[c]	×	√	√	√	√	N.D.
2[d]	√	×	√	√	√	N.D.
3[e]	√	√	×	√	√	N.D.
4[f]	√	√	√	×	√	N.D.
5[g]	√	√	√	√	×	N.D.

[a]Standard procedure: **1aa** (0.2 mmol), NaO^{t}Bu (1.0 equiv), THF (1.2 mL), CS_2 (1.5 equiv), 3h. After removing solvent *in vacuo*, then **3a** (2.0 equiv), [Ir(ppy)$_2$dtbbpy](PF_6) (1 mol%), PPh_3 (1.2 equiv), H_2O (7.0 equiv), MeCN (2.0 mL), 24h. [b]Determined by ^{1}H NMR analysis with 1,3,5-trimethoxybenzene as an internal standard. [c]Without NaO^{t}Bu. [d]Without CS_2. [e]Without photocatalyst [Ir(ppy)$_2$dtbbpy](PF_6). [f] Without *hv*. [g]Without PPh_3.

3. General Procedure and Spectral Data of Products

3.1 Procedure for Synthesis of 2ad

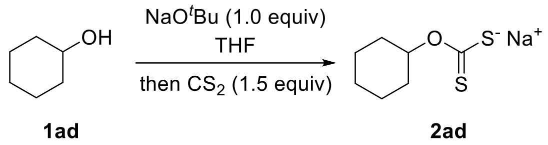

In a nitrogen-filled glovebox, an oven-dried 25 mL round-bottom flask equipped with a magnetic stir bar was charged sequentially with **1ad** (0.20 g, 2.0 mmol), NaO^{t}Bu (0.19 g, 2.0 mmol), followed by addition of dry Et_2O (12 mL). The flask was sealed with a septum cap and transferred out of the glovebox. Then stirred at room temperature for 30 min. The resulting solution was cooled to 0 °C followed by addition of CS_2 (0.457 g, 0.38 mL, 6.0 mmol) via syringe, then stirred at 0 °C for 3 h. The pale-yellow precipitate formed was collected by filtration, washed with Et_2O (2 × 10 mL), and dried *in vacuo* to afford the desired product as a pale-yellow solid (0.38 g, 95% yield).

3.2 General Procedure for Deoxygenative Alkylation of Alcohols

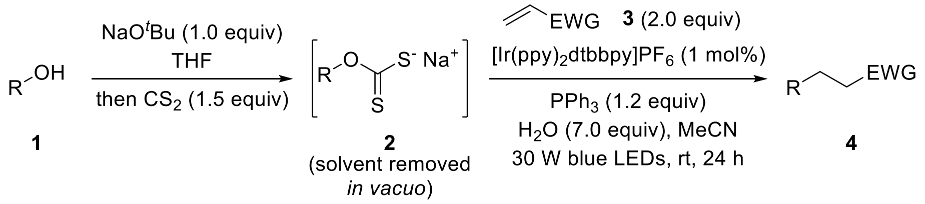

In a nitrogen-filled glovebox, an oven-dried 5 mL quartz tube equipped with a magnetic stir bar was charged sequentially with alcohol **1** (0.20 mmol), NaO^{t}Bu (19.2 mg, 0.20 mmol), followed by addition of dry THF (1.2 mL). The quartz tube was sealed with a septum cap and transferred out of the glovebox, then stirred at room temperature for 30 min. The resulting solution was cooled to 0 °C followed by addition of CS_2 (22.8 mg, 19.0 µL, 0.30 mmol) via microsyringe, then stirred at 0 °C for 3 h before removing the solvent *in vacuo*. The system was transferred into the glovebox, and PPh_3 (63.0 mg, 0.24 mmol), [Ir(ppy)$_2$dtbbpy](PF$_6$) (1.8 mg, 1.0 mol%), alkene **3** (0.40 mmol), H_2O (25.2 mg, 1.4 mmol) in MeCN (2.0 mL) was added. Then, the quartz tube was sealed with a septum cap and transferred out of the glovebox. The reaction mixture was irradiated with a 30 W blue LEDs lamp, maintained at a temperature of 26 °C, and stirred for 24 hours. The solvent was removed *in vacuo* and the residue was purified by column chromatography to afford the product.

3.3 Spectral Data of Products

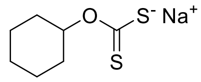
2ad

sodium O-cyclohexyl carbonodithioate (2ad): ^{1}H NMR (400 MHz, DMSO-*d6*) δ 5.21 (m, *J* = 9.3, 3.9 Hz, 1H), 1.87 (m, *J* = 13.0, 3.1 Hz, 2H), 1.67 (m, *J* = 12.1, 4.6 Hz, 2H), 1.54 – 1.47 (m, 1H), 1.37 – 1.14 (m, 5H). ^{13}C NMR (101 MHz, DMSO-*d6*) δ 229.7 (C=S), 78.1, 32.0, 25.7, 24.4. This compound is known.[1]

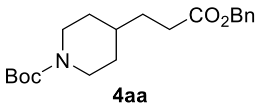
4aa

tert-butyl-4-(3-(benzyloxy)-3-oxopropyl)piperidine-1-carboxylate (4aa): The title compound was isolated by column chromatography with petroleum ether and ethyl acetate (PE/EA = 10:1) as a colorless oil (61.8 mg, 89% yield). ^{1}H NMR (400 MHz, $CDCl_3$) δ 7.37 – 7.32 (m, 5H), 5.11 (s, 2H), 4.06 (s, 2H), 2.68 – 2.56 (m, 2H), 2.38 (m, *J* = 7.7 Hz, 2H), 1.61 (m, *J* = 10.1 Hz, 4H), 1.45 (s, 9H), 1.37 (m, *J* = 7.6, 3.5 Hz, 1H), 1.07 (m, *J* = 12.5, 4.4 Hz, 2H). ^{13}C NMR (101 MHz, $CDCl_3$) δ 173.5, 154.9, 136.1, 128.6, 128.3, 79.3, 66.3, 44.0, 35.5, 31.9, 31.6, 31.5, 28.5. IR (ATR): 3033, 2974, 2929, 2853, 1737, 1423, 1366, 1246, 1161, 972, 866, 751, 699 cm^{-1}. HRMS (EI): m/z $[M + Na]^+$ calcd for $C_{20}H_{29}NO_4Na$: 370.1989, found: 370.1993.

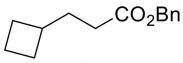
4ab

benzyl 3-cyclobutylpropanoate (4ab): The title compound was isolated by column chromatography with petroleum ether and ethyl acetate (PE/EA = 400:1) as a colorless oil (31.5 mg, 64% yield). ^{1}H NMR (400 MHz, $CDCl_3$) δ 7.38 – 7.31 (m, 5H), 5.10 (s, 2H), 2.26 (m, *J* = 8.3, 7.1 Hz, 3H), 2.06 – 1.98 (m, 2H), 1.87 – 1.77 (m, 2H), 1.76 – 1.69 (m, 2H), 1.59 (m, *J* = 10.8, 8.3, 2.6 Hz, 2H). ^{13}C NMR (101 MHz, $CDCl_3$) δ 173.8, 136.3, 128.7, 128.3, 66.2, 35.6, 32.3, 32.1, 28.1, 18.4. IR (ATR): 3035, 2924, 2852, 1738, 1498, 1452, 1160, 1125, 748, 698 cm^{-1}. HRMS (EI): m/z $[M + Na]^+$ calcd for $C_{14}H_{18}O_2Na$: 269.1512, found: 269.1515.

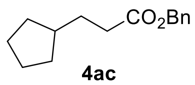
4ac

benzyl 3-cyclopentylpropanoate (4ac): The title compound was isolated by column chromatography with petroleum ether and ethyl acetate (PE/EA = 400:1) as a colorless oil (39.4 mg, 85% yield). ^{1}H NMR (400 MHz, $CDCl_3$) δ 7.36 (d, *J* = 3.1 Hz, 5H), 5.11 (s, 2H), 2.37 (dd, *J* = 8.3, 7.2 Hz, 2H), 1.75 (m, *J* = 6.0, 2.9 Hz, 3H), 1.70 – 1.58 (m, 4H), 1.50 (dd, *J* = 7.8, 4.4 Hz, 2H), 1.14 – 1.00 (m, 2H). ^{13}C NMR (101 MHz, $CDCl_3$) δ 174.0, 136.3, 128.7, 128.3, 128.3, 66.2, 39.8, 33.8, 32.5, 31.3, 25.2. IR (ATR): 3034, 2949, 2865, 1738, 1453, 1382, 1350, 1168, 747, 698 cm^{-1}. HRMS (EI): m/z $[M + Na]^+$ calcd for $C_{15}H_{20}O_2Na$: 255.1356, found: 255.1359. This

compound is known.[2]

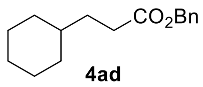

benzyl 3-cyclohexylpropanoate (4ad): The title compound was isolated by column chromatography with petroleum ether and ethyl acetate (PE/EA = 400:1) as a colorless oil (40.8 mg, 83% yield). ^{1}H NMR (400 MHz, $CDCl_3$) δ 7.43 – 7.28 (m, 5H), 5.11 (s, 2H), 2.40 – 2.33 (m, 2H), 1.73 – 1.61 (m, 5H), 1.57 – 1.50 (m, 2H), 1.26 – 1.11 (m, 4H), 0.89 (dd, *J* = 12.2, 9.6 Hz, 2H). ^{13}C NMR (101 MHz, $CDCl_3$) δ 174.1, 136.3, 128.7, 128.3, 128.3, 66.2, 37.3, 33.1, 32.4, 32.0, 26.6, 26.3. IR (ATR): 3034, 2924, 2852, 1738, 1498, 1452, 1160, 1125, 748, 698 cm^{-1}. HRMS (EI): m/z $[M + Na]^+$ calcd for $C_{16}H_{22}O_2Na$: 269.1512, found: 269.1515. This compound is known.[2]

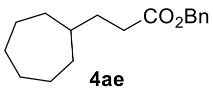

benzyl 3-cycloheptylpropanoate (4ae): The title compound was isolated by column chromatography with petroleum ether and ethyl acetate (PE/EA = 400:1) as a colorless oil (41.6 mg, 80% yield). ^{1}H NMR (400 MHz, $CDCl_3$) δ 7.35 (d, *J* = 3.6 Hz, 5H), 5.11 (s, 2H), 2.38 – 2.32 (m, 2H), 1.72 – 1.62 (m, 3H), 1.61 – 1.52 (m, 5H), 1.49 – 1.34 (m, 5H), 1.21 – 1.12 (m, 2H). ^{13}C NMR (101 MHz, $CDCl_3$) δ 174.0, 136.3, 128.6, 128.3, 128.2, 66.2, 38.9, 34.3, 33.1, 32.5, 28.6, 26.4. IR (ATR): 3034, 2924, 2854, 1738, 1498, 1457, 1383, 1170, 750, 698 cm^{-1}. HRMS (EI): m/z $[M + Na]^+$ calcd for $C_{17}H_{24}O_2Na$: 283.1669, found: 283.1671.

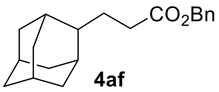

benzyl 3-((1r,3r,5r,7r)-adamantan-2-yl)propanoate (4af): The title compound was isolated by column chromatography with petroleum ether and ethyl acetate (PE/EA = 400:1) as a colorless oil (42.3 mg, 71% yield). ^{1}H NMR (400 MHz, $CDCl_3$) δ 7.40 – 7.29 (m, 5H), 5.12 (s, 2H), 2.38 – 2.32 (m, 2H), 1.90 – 1.83 (m, 4H), 1.81 – 1.74 (m, 4H), 1.69 (d, *J* = 13.7 Hz, 6H), 1.61 (d, *J* = 9.2 Hz, 1H), 1.49 (d, *J* = 11.2 Hz, 2H). ^{13}C NMR (101 MHz, $CDCl_3$) δ 174.2, 136.3, 128.7, 128.3, 128.3, 66.2, 44.1, 39.2, 38.4, 32.8, 31.7, 31.6, 28.3, 28.1, 28.0. IR (ATR): 3034, 2906, 2851, 2666, 1738, 1498, 1454, 1152, 1100, 748, 698 cm^{-1}. HRMS (EI): m/z $[M + Na]^+$ calcd for $C_{20}H_{26}O_2Na$: 321.1825, found: 321.1830. This compound is known.[2]

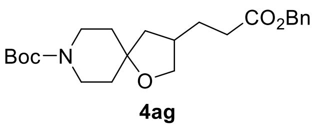

tert-butyl-3-(3-(benzyloxy)-3-oxopropyl)-1-oxa-8-azaspiro[4.5]decane -8-carboxylate (4ag): The title compound was isolated by column chromatography with petroleum ether and ethyl acetate (PE/EA = 8:1) as a colorless oil (59.6 mg, 74% yield). ^{1}H NMR (400 MHz, $CDCl_3$) δ 7.39 – 7.31 (m, 5H), 5.12 (s, 2H), 4.00 – 3.89 (m, 1H), 3.55 (d, *J* = 13.3 Hz, 2H), 3.40 (m, *J* = 8.5 Hz,

1H), 3.36 – 3.23 (m, 2H), 2.32 (m, *J* = 33.5, 7.8 Hz, 3H), 1.93 (dd, *J* = 12.4, 7.9 Hz, 1H), 1.83 – 1.62 (m, 3H), 1.57 (dd, *J* = 9.3, 4.6 Hz, 3H), 1.45 (s, 9H), 1.28 (dd, *J* = 12.3, 9.2 Hz, 1H). ^{13}C NMR (101 MHz, $CDCl_3$) δ 173.1, 154.9, 136.0, 128.7, 128.4, 128.4, 80.5, 79.5, 71.9, 66.4, 43.5, 41.2, 38.8, 37.2, 36.5, 33.3, 28.6, 28.5. IR (ATR): 3033, 2938, 2866, 1737, 1692, 1422, 1244, 1154, 964, 752, 699 cm^{-1}. HRMS (EI): m/z [M + Na]$^+$ calcd for $C_{22}H_{33}NO_5Na$: 426.2251, found: 426.2253.

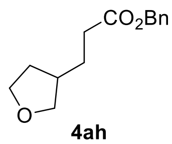

4ah

benzyl 3-(tetrahydrofuran-3-yl)propanoate (4ah): The title compound was isolated by column chromatography with petroleum ether and ethyl acetate (PE/EA = 10:1) as a colorless oil (36.0 mg, 77% yield). ^{1}H NMR (400 MHz, $CDCl_3$) δ 7.40 – 7.30 (m, 5H), 5.12 (s, 2H), 3.92 – 3.81 (m, 2H), 3.77 – 3.69 (m, 1H), 3.34 (dd, *J* = 8.4, 7.1 Hz, 1H), 2.39 (m, *J* = 7.9, 2.9 Hz, 2H), 2.24 – 2.12 (m, 1H), 2.04 (m, *J* = 12.2, 7.5, 4.7 Hz, 1H), 1.77 – 1.71 (m, 2H), 1.56 – 1.45 (m, 1H). ^{13}C NMR (101 MHz, $CDCl_3$) δ 173.2, 136.0, 128.7, 128.4, 128.4, 73.1, 68.0, 66.4, 38.9, 33.3, 32.2, 28.4. IR (ATR): 3034, 2934, 2864, 1736, 1454, 1385, 1260, 1162, 908, 750, 670 cm^{-1}. HRMS (EI): m/z [M + Na]$^+$ calcd for $C_{14}H_{18}O_3Na$: 257.1148, found: 257.1150.

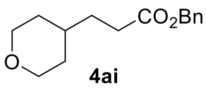

4ai

benzyl 3-(tetrahydro-2H-pyran-4-yl)propanoate (4ai): The title compound was isolated by column chromatography with petroleum ether and ethyl acetate (PE/EA = 10:1) as a colorless oil (41.2 mg, 83% yield). ^{1}H NMR (400 MHz, $CDCl_3$) δ 7.40 – 7.30 (m, 5H), 5.12 (s, 2H), 3.93 (m, *J* = 11.6, 4.5, 1.1 Hz, 2H), 3.33 (m, *J* = 11.8, 2.1 Hz, 2H), 2.42 – 2.35 (m, 2H), 1.65 – 1.57 (m, 3H), 1.56 (dd, *J* = 3.8, 1.9 Hz, 1H), 1.47 (m, *J* = 16.3, 8.1, 4.6 Hz, 1H), 1.32 – 1.23 (m, 2H). ^{13}C NMR (101 MHz, $CDCl_3$) δ 173.6, 136.1, 128.7, 128.4, 68.0, 66.3, 34.5, 32.8, 31.9, 31.5. IR (ATR): 3034, 2928, 2843, 1737, 1453, 1385, 1265, 1235, 1166, 988, 749, 699 cm^{-1}. HRMS (EI): m/z [M + Na]$^+$ calcd for $C_{15}H_{20}O_3Na$: 271.1305, found: 271.1308.

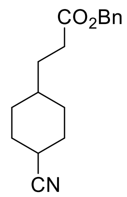

4aj

benzyl 3-(4-cyanocyclohexyl)propanoate (4aj): The title compound was isolated by column chromatography with petroleum ether and ethyl acetate (PE/EA = 20:1) as a colorless oil (41.7 mg, 77% yield, *dr* = 1.4:1). Data of major isomer: ^{1}H NMR (400 MHz, $CDCl_3$) δ 7.35 (m, *J* = 5.1, 4.5 Hz, 5H), 5.11 (s, 2H), 2.93 – 2.62 (m, 1H), 2.37 (m, *J* = 10.4, 7.6 Hz, 2H), 2.08 (dd, *J* = 14.0, 3.7 Hz, 2H), 1.80 (dd, *J* = 13.8, 3.7 Hz, 2H), 1.66 – 1.43 (m, 4H), 1.28 (m, *J* = 11.3, 7.6, 2.9 Hz, 2H), 0.91 (dd, *J* = 11.7, 3.4 Hz, 1H). ^{13}C NMR (101 MHz, $CDCl_3$) δ 173.5, 136.1, 128.7, 128.4, 128.3, 122.7, 66.4, 35.8, 31.7, 31.6, 31.3, 28.6, 28.2. Data of minor isomer: ^{1}H NMR (400 MHz, $CDCl_3$) δ 7.35 (m, *J* = 5.1, 4.5 Hz, 5H), 5.13

(s, 2H), 2.93 – 2.62 (m, 1H), 2.37 (m, *J* = 10.4, 7.6 Hz, 2H), 1.96 (dd, *J* = 13.8, 3.5 Hz, 2H), 1.71 (d, *J* = 10.0, 2H), 1.66 – 1.43 (m, 4H), 1.28 (m, *J* = 11.3, 7.6, 2.9 Hz, 2H), 0.91 (dd, *J* = 11.7, 3.4 Hz, 1H). ^{13}C NMR (101 MHz, $CDCl_3$) δ 173.6, 136.1, 128.7, 128.4, 128.3, 122.1, 66.3, 36.1, 31.7, 31.5, 29.6, 28.3, 27.2. IR (ATR): 2934, 2859, 1735, 1453, 1384, 1257, 1163, 749, 699 cm^{-1}. HRMS (EI): m/z $[M + H]^+$ calcd for $C_{17}H_{22}NO_2$: 272.1645, found: 272.1639.

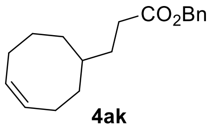

4ak

benzyl (*Z*)-3-(cyclooct-4-en-1-yl)propanoate (4ak): The title compound was isolated by column chromatography with petroleum ether and ethyl acetate (PE/EA = 400:1) as a colorless oil (34.3 mg, 63% yield). ^{1}H NMR (400 MHz, $CDCl_3$) δ 7.38 – 7.30 (m, 5H), 5.67 – 5.55 (m, 2H), 5.11 (s, 2H), 2.32 (m, *J* = 25.8, 14.0, 6.6, 4.0 Hz, 3H), 2.08 (m, *J* = 30.0, 10.7, 7.3, 3.9 Hz, 3H), 1.67 – 1.59 (m, 2H), 1.57 – 1.50 (m, 2H), 1.45 – 1.34 (m, 3H), 1.30 – 1.24 (m, 1H), 1.21 – 1.12 (m, 1H). ^{13}C NMR (101 MHz, $CDCl_3$) δ 174.0, 136.2, 130.3, 130.1, 128.7, 128.3, 128.3, 66.2, 37.1, 35.3, 33.9, 32.7, 32.4, 28.2, 26.1, 25.2. IR (ATR): 3016, 2925, 2849, 1740, 1454, 1383, 1265, 1155, 994, 750, 697 cm^{-1}. HRMS (EI): m/z $[M + Na]^+$ calcd for $C_{18}H_{24}O_2Na$: 295.1669, found: 295.1666.

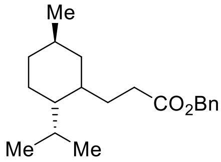

4al

benzyl 3-(2-isopropyl-5-methylcyclohexyl)propanoate (4al): The title compound was isolated by column chromatography with petroleum ether and ethyl acetate (PE/EA = 400:1) as a colorless oil (45.3 mg, 75% yield, *dr* = 1.3:0.7). Data of major isomer: ^{1}H NMR (400 MHz, $CDCl_3$) δ 7.38 – 7.31 (m, 5H), 5.12 (s, 2H), 2.44 – 2.24 (m, 2H), 2.05 – 1.73 (m, 2H), 1.69 (m, *J* = 9.9, 5.3, 2.5 Hz, 2H), 1.65 – 1.60 (m, 1H), 1.46 – 1.21 (m, 3H), 1.11 – 0.90 (m, 2H), 0.89 (d, *J* = 1.5 Hz, 2H), 0.88 – 0.85 (m, 3H), 0.83 (d, *J* = 7.9 Hz, 3H), 0.72 – 0.59 (m, 3H). ^{13}C NMR (101 MHz, $CDCl_3$) δ 174.3, 136.3, 128.7, 128.4, 128.3, 66.2, 46.6, 40.9, 38.3, 35.4, 32.9, 31.1, 27.9, 26.5, 24.4, 22.9, 21.7, 15.3. Data of minor isomer: ^{1}H NMR (400 MHz, $CDCl_3$) δ 7.38 – 7.31 (m, 5H), 5.11 (s, 2H), 2.44 – 2.24 (m, 2H), 2.05 – 1.73 (m, 2H), 1.69 (m, *J* = 9.9, 5.3, 2.5 Hz, 2H), 1.65 – 1.60 (m, 1H), 1.46 – 1.21 (m, 3H), 1.11 – 0.90 (m, 2H), 0.89 (d, *J* = 1.5 Hz, 2H), 0.88 – 0.85 (m, 3H), 0.83 (d, *J* = 7.9 Hz, 3H), 0.72 – 0.59 (m, 3H). ^{13}C NMR (101 MHz, $CDCl_3$) δ 174.2, 136.2, 128.7, 128.4, 128.3, 66.3, 48.4, 38.0, 35.9, 34.8, 32.8, 29.3, 26.0, 25.2, 22.9, 21.8, 20.9, 20.6. IR (ATR): 3035, 3017, 2923, 2851, 1732, 1453, 1379, 1264, 1155, 991, 749, 698 cm^{-1}. HRMS (EI): m/z $[M + Na]^+$ calcd for $C_{20}H_{30}O_2Na$: 325.2138, found: 325.2130. This compound is known.[2]

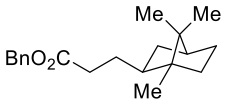
4am

benzyl-3-((1*S*,2*S*,4*S*)-1,7,7-trimethylbicyclo[2.2.1]heptan-2-yl)propanoate (4am): The title compound was isolated by column chromatography with petroleum ether and ethyl acetate (PE/EA = 400:1) as a colorless oil (43.2 mg, 72% yield, *dr* = 1:1). Data of one isomer: ^{1}H NMR (600 MHz, $CDCl_3$) δ 7.39 – 7.29 (m, 5H), 5.12 (s, 2H), 2.45 – 2.17 (m, 2H), 1.99 – 1.86 (m, 1H), 1.79 – 1.63 (m, 2H), 1.58 – 1.43 (m, 4H), 1.37 – 1.25 (m, 1H), 1.07 (m, *J* = 24.1, 12.5, 9.4, 4.7 Hz, 2H), 0.85 (d, *J* = 5.7 Hz, 3H), 0.82 (d, *J* = 2.3 Hz, 3H), 0.78 (d, *J* = 29.0 Hz, 3H). ^{13}C NMR (151 MHz, $CDCl_3$) δ 174.1, 136.3, 128.7, 128.4, 128.3, 66.2, 48.9, 47.4, 47.4, 45.4, 35.9, 34.1, 29.4, 28.6, 27.1, 20.8, 19.7, 14.4. Data of the other isomer: ^{1}H NMR (600 MHz, $CDCl_3$) δ 7.39 – 7.29 (m, 5H), 5.11 (s, 2H), 2.45 – 2.17 (m, 2H), 1.99 – 1.86 (m, 1H), 1.79 – 1.63 (m, 2H), 1.58 – 1.43 (m, 4H), 1.37 – 1.25 (m, 1H), 1.07 (m, *J* = 24.1, 12.5, 9.4, 4.7 Hz, 2H), 0.85 (d, *J* = 5.7 Hz, 3H), 0.82 (d, *J* = 2.3 Hz, 3H), 0.78 (d, *J* = 29.0 Hz, 3H). ^{13}C NMR (151 MHz, $CDCl_3$) δ 174.0, 136.3, 128.7, 128.4, 128.3, 66.2, 48.0, 47.3, 45.3, 43.4, 39.9, 36.2, 33.5, 28.7, 27.4, 20.9, 18.6, 13.2. IR (ATR): 3070, 3034, 2950, 2876, 1730, 1456, 1387, 1157, 990, 750, 699 cm^{-1}. HRMS (EI): m/z $[M + Na]^+$ calcd for $C_{20}H_{28}O_2Na$: 323.1982, found: 323.1988.

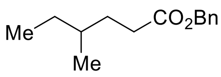
4an

benzyl 4-methylhexanoate (4an): The title compound was isolated by column chromatography with petroleum ether and ethyl acetate (PE/EA = 400:1) as a colorless oil (27.7 mg, 63% yield). ^{1}H NMR (400 MHz, $CDCl_3$) δ 7.38 – 7.30 (m, 5H), 5.11 (s, 2H), 2.36 (m, *J* = 9.5, 6.3 Hz, 2H), 1.75 – 1.65 (m, 1H), 1.52 – 1.42 (m, 1H), 1.34 (m, *J* = 12.3, 7.0, 3.3 Hz, 2H), 1.20 – 1.09 (m, 1H), 0.87 (d, *J* = 3.4 Hz, 3H), 0.86 – 0.84 (m, 3H). ^{13}C NMR (101 MHz, $CDCl_3$) δ 174.1, 136.3, 128.7, 128.3, 128.3, 66.2, 34.1, 32.3, 31.6, 29.2, 18.9, 11.4. IR (ATR): 3034, 2962, 2930, 2875, 1739, 1459, 1381, 1169, 748, 698 cm^{-1}. HRMS (EI): m/z $[M + Na]^+$ calcd for $C_{14}H_{20}O_2Na$: 243.1356, found 243.1358. This compound is known.[2]

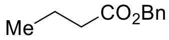
4ba

benzyl butyrate (4ba): The title compound was isolated by column chromatography with petroleum ether and ethyl acetate (PE/EA = 400:1) as a colorless oil (21.4 mg, 60% yield). 1H NMR (400 MHz, $CDCl_3$) δ 7.42 – 7.30 (m, 5H), 5.13 (s, 2H), 2.35 (m, *J* = 7.4 Hz, 2H), 1.69 (m, *J* = 7.4 Hz, 2H), 0.96 (m, *J* = 7.4 Hz, 3H). ^{13}C NMR (101 MHz, $CDCl_3$) δ 173.6, 136.3, 128.6, 128.3, 66.1, 36.3, 18.6, 13.8. IR (ATR): 3034, 2965, 2932, 1737, 1437, 1260, 1174, 746, 699 cm^{-1}. HRMS (EI): m/z $[M + Na]^+$ calcd for $C_{11}H_{14}O_2Na$: 201.0886, found 201.0883. This compound is known.[2]

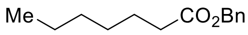

4bb

benzyl heptanoate (4bb): The title compound was isolated by column chromatography with petroleum ether and ethyl acetate (PE/EA = 400:1) as a colorless oil (26.4 mg, 60% yield). ^{1}H NMR (400 MHz, $CDCl_3$) δ 7.38 – 7.30 (m, 5H), 5.11 (s, 2H), 2.35 (m, *J* = 7.5 Hz, 2H), 1.62 (m, *J* = 10.8, 6.1 Hz, 2H), 1.38 – 1.20 (m, 6H), 0.88 (d, *J* = 5.9 Hz, 3H). ^{13}C NMR (101 MHz, $CDCl_3$) δ 173.8, 136.3, 128.7, 128.3, 128.3, 66.2, 34.5, 31.6, 28.9, 25.1, 22.6, 14.1. IR (ATR): 3034, 2955, 2828, 2853, 1737, 1457, 1382, 1165, 1104, 750, 699 cm^{-1}. HRMS (EI): m/z [M + Na]$^+$ calcd for $C_{14}H_{20}O_2Na$: 243.1356, found 243.1353. This compound is known.[2]

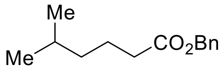

4bc

benzyl 5-methylhexanoate (4bc): The title compound was isolated by column chromatography with petroleum ether and ethyl acetate (PE/EA = 400:1) as a colorless oil (26.8 mg, 61% yield). ^{1}H NMR (400 MHz, $CDCl_3$) δ 7.39 – 7.31 (m, 5H), 5.12 (s, 2H), 2.34 (m, *J* = 7.6 Hz, 2H), 1.69 – 1.61 (m, 2H), 1.53 (m, *J* = 13.3, 6.7 Hz, 1H), 1.19 (dd, *J* = 16.0, 6.9 Hz, 2H), 0.87 (d, *J* = 6.7 Hz, 6H). ^{13}C NMR (101 MHz, $CDCl_3$) δ 173.8, 136.3, 128.7, 128.3, 128.3, 66.2, 38.5, 34.7, 27.9, 23.0, 22.6. IR (ATR): 3034, 2956, 2871, 1739, 1458, 1384, 1252, 1168, 1110, 1001, 749, 698 cm^{-1}. HRMS (EI): m/z [M + Na]$^+$ calcd for $C_{14}H_{20}O_2Na$: 243.1356, found 243.1353. This compound is known.[3]

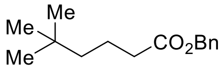

4bd

benzyl 5,5-dimethylhexanoate (4bd): The title compound was isolated by column chromatography with petroleum ether and ethyl acetate (PE/EA = 400:1) as a colorless oil (29.0 mg, 62% yield). ^{1}H NMR (400 MHz, $CDCl_3$) δ 7.38 – 7.31 (m, 5H), 5.12 (s, 2H), 2.33 (m, *J* = 7.5 Hz, 2H), 1.65 – 1.59 (m, 2H), 1.59 – 1.58 (m, 2H), 0.87 (s, 9H). ^{13}C NMR (101 MHz, $CDCl_3$) δ 173.9, 136.3, 128.7, 128.4, 128.3, 66.2, 43.7, 35.2, 30.4, 29.4, 20.4. IR (ATR): 3035, 2953, 1734, 1465, 1363, 1254, 1134, 1064, 748, 699 cm^{-1}. HRMS (EI): m/z [M + Na]$^+$ calcd for $C_{15}H_{22}O_2Na$: 257.1512, found 257.1510. This compound is known.[4]

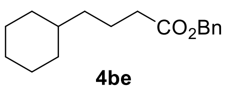

4be

benzyl 4-cyclohexylbutanoate (4be): The title compound was isolated by column chromatography with petroleum ether and ethyl acetate (PE/EA = 400:1) as a colorless oil (34.8 mg, 67% yield). ^{1}H NMR (400 MHz, $CDCl_3$) δ 7.40 – 7.29 (m, 5H), 5.11 (s, 2H), 2.33 (m, *J* = 7.6 Hz, 2H), 1.67 (m, *J* = 13.7, 10.2, 6.3 Hz, 7H), 1.18 (m, *J* = 14.3, 10.4, 6.3 Hz, 6H), 0.91 – 0.80 (m, 2H). ^{13}C NMR (101 MHz, $CDCl_3$) δ 173.8, 136.3, 128.7, 128.3, 128.3, 66.2, 37.5, 37.0, 34.7, 33.4, 26.8, 26.5, 22.5. IR (ATR): 3034, 2924, 2850, 1739, 1498, 1452, 1382, 1259, 1160, 747, 698 cm^{-1}. HRMS (EI): m/z [M + Na]$^+$ calcd for $C_{17}H_{24}O_2Na$: 283.1669, found 283.1671.

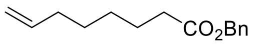

4bf

benzyl oct-7-enoate (4bf): The title compound was isolated by column chromatography with petroleum ether and ethyl acetate (PE/EA = 400:1) as a colorless oil (23.2 mg, 50% yield). ^{1}H NMR (400 MHz, $CDCl_3$) δ 7.39 – 7.31 (m, 5H), 5.85 – 5.72 (m, 1H), 5.11 (s, 2H), 4.99 (dd, *J* = 17.1, 1.9 Hz, 1H), 4.96 – 4.91 (m, 1H), 2.36 (m, *J* = 7.5 Hz, 2H), 2.07 – 2.00 (m, 2H), 1.68 – 1.61 (m, 2H), 1.38 (m, *J* = 6.0, 3.5 Hz, 2H), 1.34 – 1.26 (m, 2H). ^{13}C NMR (101 MHz, $CDCl_3$) δ 173.8, 138.9, 136.2, 128.7, 128.3, 114.6, 66.2, 34.4, 33.7, 28.7, 28.6, 24.9. IR (ATR): 3034, 2929, 2856, 1737, 1640, 1497, 1455, 1382, 1163, 994, 910, 750, 697 cm^{-1}. HRMS (EI): m/z $[M + Na]^+$ calcd for $C_{15}H_{20}O_2Na$: 255.1356, found 255.1350.

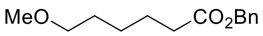

4bg

benzyl 6-methoxyhexanoate (4bg): The title compound was isolated by column chromatography with petroleum ether and ethyl acetate (PE/EA = 20:1) as a colorless oil (26.0 mg, 55% yield). ^{1}H NMR (400 MHz, $CDCl_3$) δ 7.37 – 7.31 (m, 5H), 5.11 (s, 2H), 3.35 (m, *J* = 6.5 Hz, 2H), 3.32 (s, 3H), 2.37 (m, *J* = 7.5 Hz, 2H), 1.72 – 1.64 (m, 2H), 1.63 – 1.57 (m, 2H), 1.43 – 1.34 (m, 2H). ^{13}C NMR (101 MHz, $CDCl_3$) δ 173.6, 136.2, 128.7, 128.3, 72.7, 66.2, 58.7, 34.4, 29.4, 25.9, 24.9. IR (ATR): 3034, 2934, 2865, 1737, 1498, 1456, 1385, 1163, 1119, 750, 699 cm^{-1}. HRMS (EI): m/z $[M + Na]^+$ calcd for $C_{14}H_{20}O_3Na$: 259.1305, found 259.1305.

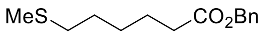

4bh

benzyl 6-(methylthio)hexanoate (4bh): The title compound was isolated by column chromatography with petroleum ether and ethyl acetate (PE/EA = 20:1) as a colorless oil (26.7 mg, 53% yield). ^{1}H NMR (400 MHz, $CDCl_3$) δ 7.35 (d, *J* = 5.4 Hz, 5H), 5.11 (s, 2H), 2.47 (m, *J* = 7.3 Hz, 2H), 2.37 (m, *J* = 7.5 Hz, 2H), 2.08 (s, 3H), 1.66 (m, *J* = 7.6 Hz, 2H), 1.60 (d, *J* = 9.3 Hz, 2H), 1.46 – 1.39 (m, 2H). ^{13}C NMR (101 MHz, $CDCl_3$) δ 173.6, 136.2, 128.7, 128.3, 66.3, 34.3, 34.1, 28.9, 28.4, 24.7, 15.6. IR (ATR): 3034, 2934, 2858, 1736, 1456, 1258, 1166, 975, 747, 698 cm^{-1}. HRMS (EI): m/z $[M + H]^+$ calcd for $C_{14}H_{21}O_2S$: 253.1257, found 253.1252.

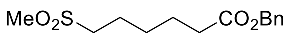

4bi

benzyl 6-(methylsulfonyl)hexanoate (4bi): The title compound was isolated by column chromatography with petroleum ether and ethyl acetate (PE/EA = 2:1) as a colorless oil (43.2 mg, 76% yield). ^{1}H NMR (400 MHz, $CDCl_3$) δ 7.34 (m, *J* = 7.1 Hz, 5H), 5.11 (s, 2H), 3.01 – 2.95 (m, 2H), 2.87 (s, 3H), 2.39 (m, *J* = 7.3 Hz, 2H), 1.85 (m, *J* = 7.8 Hz, 2H), 1.70 (d, *J* = 7.7 Hz, 2H), 1.49 (m, *J* = 8.0 Hz, 2H). ^{13}C NMR (101 MHz, $CDCl_3$) δ 173.2, 136.0, 128.7, 128.4, 128.4, 66.4, 54.6, 40.6, 33.9, 27.9, 24.4, 22.2 IR (ATR): 3709, 3031, 2933, 1729, 1456, 1293, 1135, 963, 748, 699, 519, 471 cm-1. HRMS (EI): m/z $[M + H]^+$ calcd for $C_{14}H_{21}O_4S$:

285.1155, found 285.1150.

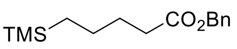

4bj

benzyl 6-(methylsulfonyl)hexanoate (4bj): The title compound was isolated by column chromatography with petroleum ether and ethyl acetate (PE/EA = 40:1) as a colorless oil (32.2 mg, 61% yield). ^{1}H NMR (400 MHz, $CDCl_3$) δ 7.39 – 7.32 (m, 5H), 5.12 (s, 2H), 2.37 (m, J = 7.5 Hz, 2H), 1.70 – 1.64 (m, 2H), 1.37 – 1.30 (m, 2H), 0.52 – 0.46 (m, 2H), -0.03 (s, 9H). ^{13}C NMR (101 MHz, $CDCl_3$) δ 173.8, 136.3, 128.7, 128.3, 66.2, 34.2, 28.9, 23.7, 16.5, -1.6. IR (ATR): 3067, 2953, 1739, 1456, 1381, 1248, 1185, 1159, 863, 836, 749, 697 cm-1. HRMS (EI): m/z $[M + H]^+$ calcd for $C_{15}H_{25}O_2Si$: 265.1618, found 265.1614.

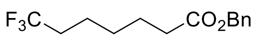

4bk

benzyl 7,7,7-trifluoroheptanoate (4bk): The title compound was isolated by column chromatography with petroleum ether and ethyl acetate (PE/EA = 400:1) as a colorless oil (29.1 mg, 53% yield). ^{1}H NMR (400 MHz, $CDCl_3$) δ 7.39 – 7.31 (m, 5H), 5.12 (s, 2H), 2.37 (m, J = 7.4 Hz, 2H), 2.11 – 2.00 (m, 2H), 1.72 – 1.65 (m, 2H), 1.61 – 1.56 (m, 2H), 1.40 (m, J = 8.4 Hz, 2H). ^{13}C NMR (101 MHz, $CDCl_3$) δ 173.4, 136.1, 128.7, 128.4, 128.4, 127.3 (q, J = 274.7 Hz), 66.4, 34.1, 33.7 (d, J = 28.1 Hz), 28.3, 24.6, 21.7 (q, J = 3.1 Hz). ^{19}F NMR (376 MHz, $CDCl_3$) δ -66.4. IR (ATR): 3045, 2953, 2868, 1739, 1463, 1394, 1263, 1170, 1139, 743, 697, 654 cm^{-1}. HRMS (EI): m/z $[M + Na]^+$ calcd for $C_{14}H_{17}F_3O_2Na$: 297.1073, found 297.1089. This compound is known.[2]

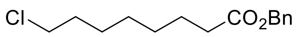

4bl

benzyl 8-chlorooctanoate (4bl): The title compound was isolated by column chromatography with petroleum ether and ethyl acetate (PE/EA = 400:1) as a colorless oil (20.4 mg, 38% yield). ^{1}H NMR (400 MHz, $CDCl_3$) δ 7.35 (d, J = 5.2 Hz, 5H), 5.11 (s, 2H), 3.52 (m, J = 6.7 Hz, 2H), 2.36 (m, J = 7.5 Hz, 2H), 1.74 (m, J = 7.1 Hz, 2H), 1.65 (m, J = 6.9 Hz, 2H), 1.45 – 1.38 (m, 2H), 1.32 (m, J = 3.6 Hz, 4H). ^{13}C NMR (101 MHz, $CDCl_3$) δ 173.7, 136.2, 128.7, 128.3, 66.3, 45.2, 34.4, 32.6, 29.0, 28.6, 26.8, 25.0. IR (ATR): 3034, 2933, 2858, 1738, 1456, 1381, 1166, 736, 698, 649 cm^{-1}. HRMS (EI): m/z $[M + H]^+$ calcd for $C_{15}H_{22}ClO_2$: 269.1303, found 269.1305.

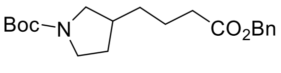

4bm

tert-butyl 3-(4-(benzyloxy)-4-oxobutyl)pyrrolidine-1-carboxylate (4bm): The title compound was isolated by column chromatography with petroleum ether and ethyl acetate (PE/EA = 10:1) as a colorless oil (43.0 mg, 62% yield, mixture of rotamers). Data of one isomer: ^{1}H NMR (400 MHz, $CDCl_3$) δ 7.40 – 7.30

(m, 5H), 5.11 (s, 2H), 3.58 – 3.35 (m, 2H), 3.24 (dd, *J* = 20.4, 9.6 Hz, 1H), 2.83 (m, *J* = 19.7, 9.7 Hz, 1H), 2.37 (m, *J* = 7.5 Hz, 2H), 2.12 – 2.03 (m, 2H), 1.70 – 1.63 (m, 3H), 1.45 (s, 9H), 1.39 (m, *J* = 7.7 Hz, 2H). ^{13}C NMR (101 MHz, $CDCl_3$) δ 173.4, 154.7, 136.1, 128.7, 128.4, 79.1, 66.3, 51.7, 45.5, 39.0, 34.4, 32.8, 31.8, 28.7. Data of the other isomer: ^{1}H NMR (400 MHz, $CDCl_3$) δ 7.40 – 7.30 (m, 5H), 5.11 (s, 2H), 3.58 – 3.35 (m, 2H), 3.24 (dd, *J* = 20.4, 9.6 Hz, 1H), 2.83 (m, *J* = 19.7, 9.7 Hz, 1H), 2.37 (m, *J* = 7.5 Hz, 2H), 1.99 – 1.92 (m, 2H), 1.70 – 1.63 (m, 3H), 1.45 (s, 9H), 1.39 (m, *J* = 7.7 Hz, 2H). ^{13}C NMR (101 MHz, $CDCl_3$) δ 173.4, 154.7, 136.1, 128.7, 128.4, 79.1, 66.3, 51.3, 45.8, 38.1, 32.8, 31.2, 28.7, 23.7. IR (ATR): 3034, 2975, 2931, 2869, 1740, 1691, 1451, 1403, 1173, 1124, 879, 751, 699 cm^{-1}. HRMS (EI): m/z [M + Na]$^+$ calcd for $C_{20}H_{29}NO_4Na$: 370.1989, found 370.1900.

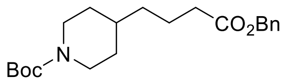

4bn

tert-butyl 4-(4-(benzyloxy)-4-oxobutyl)piperidine-1-carboxylate (4bn): The title compound was isolated by column chromatography with petroleum ether and ethyl acetate (PE/EA = 10:1) as a colorless oil (51.3 mg, 71% yield). ^{1}H NMR (400 MHz, $CDCl_3$) δ 7.38 – 7.30 (m, 5H), 5.11 (s, 2H), 4.06 (s, 2H), 2.65 (m, *J* = 12.5 Hz, 2H), 2.35 (m, *J* = 7.5 Hz, 2H), 1.73 – 1.58 (m, 5H), 1.45 (s, 9H), 1.28 – 1.23 (m, 2H), 1.06 (m, *J* = 12.4, 4.3 Hz, 2H). ^{13}C NMR (101 MHz, $CDCl_3$) δ 173.5, 155.0, 136.2, 128.7, 128.4, 79.3, 66.3, 44.1, 36.0, 35.9, 34.5, 32.2, 28.6, 22.2. IR (ATR): 3033, 2973, 2929, 2851, 1737, 1692, 1423, 1245, 1161, 750, 699 cm^{-1}. HRMS (EI): m/z [M + Na]$^+$ calcd for $C_{21}H_{31}NO_4Na$: 384.2145, found 384.2148.

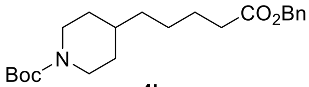

4bo

tert-butyl 4-(5-(benzyloxy)-5-oxopentyl)piperidine-1-carboxylate (4bo): The title compound was isolated by column chromatography with petroleum ether and ethyl acetate (PE/EA = 10:1) as a colorless oil (47.3 mg, 63% yield). ^{1}H NMR (400 MHz, $CDCl_3$) δ 7.38 – 7.32 (m, 5H), 5.11 (s, 2H), 4.05 (s, 2H), 2.66 (d, *J* = 13.1 Hz, 2H), 2.36 (m, *J* = 7.5 Hz, 2H), 1.65 (d, *J* = 4.0 Hz, 2H), 1.62 (d, *J* = 7.6 Hz, 2H), 1.45 (s, 9H), 1.36 – 1.29 (m, 3H), 1.26 – 1.21 (m, 2H), 1.04 (m, *J* = 12.4, 4.3 Hz, 2H). ^{13}C NMR (101 MHz, $CDCl_3$) δ 173.6, 155.0, 136.2, 128.7, 128.3, 128.3, 79.2, 66.2, 44.1, 36.2, 35.9, 34.4, 32.2, 28.6, 26.2, 25.2. IR (ATR): 3034, 2973, 2930, 2853, 1738, 1692, 1423, 1244, 1161, 750, 699 cm^{-1}. HRMS (EI): m/z [M + Na]$^+$ calcd for $C_{22}H_{33}NO_4Na$: 398.2302, found 398.2305.

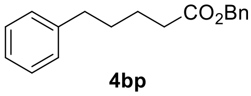

4bp

benzyl 5-phenylpentanoate (4bp): The title compound was isolated by column chromatography with petroleum ether and ethyl acetate (PE/EA =

400:1) as a colorless oil (23.0 mg, 43% yield). ^{1}H NMR (400 MHz, $CDCl_3$) δ 7.39 – 7.31 (m, 5H), 7.30 – 7.25 (m, 2H), 7.16 (m, *J* = 7.3, 5.7 Hz, 3H), 5.11 (s, 2H), 2.62 (m, *J* = 7.2 Hz, 2H), 2.38 (m, *J* = 7.0 Hz, 2H), 1.68 (m, *J* = 10.7, 8.1, 4.0 Hz, 4H). ^{13}C NMR (101 MHz, $CDCl_3$) δ 173.6, 142.2, 136.2, 128.7, 128.5, 128.4, 128.3, 125.9, 66.3, 35.7, 34.3, 31.0, 24.7. IR (ATR): 3063, 3029, 2937, 2860, 1736, 1496, 1455, 1169, 748, 699 cm^{-1}. HRMS (EI): m/z $[M + Na]^+$ calcd for $C_{18}H_{20}O_2Na$: 291.1356, found 291.1358. This compound is known.[2]

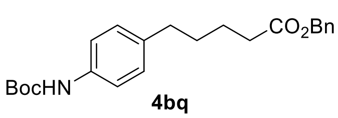

4bq

benzyl 5-phenylpentanoate (4bq): The title compound was isolated by column chromatography with petroleum ether and ethyl acetate (PE/EA = 10:1) as a colorless oil (39.8 mg, 52% yield). ^{1}H NMR (400 MHz, $CDCl_3$) δ 7.34 (d, *J* = 4.8 Hz, 5H), 7.24 (d, *J* = 7.5 Hz, 2H), 7.06 (d, *J* = 8.1 Hz, 2H), 6.46 (s, 1H), 5.10 (s, 2H), 2.55 (m, *J* = 7.3 Hz, 2H), 2.37 (m, *J* = 7.1 Hz, 2H), 1.69 – 1.59 (m, 4H), 1.51 (s, 9H). ^{13}C NMR (101 MHz, $CDCl_3$) δ 173.6, 153.0, 136.9, 136.2, 136.2, 128.9, 128.7, 128.3, 118.8, 80.4, 66.2, 34.9, 34.3, 31.0, 28.5, 24.6. IR (ATR): 3350, 3033, 2933, 2861, 1725, 1595, 1525, 1313, 1159, 1053, 1022, 832, 746, 698 cm^{-1}. HRMS (EI): m/z $[M + H]^+$ calcd for $C_{23}H_{30}NO_4$: 384.2169, found 384.2166.

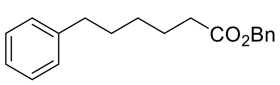

4br

benzyl 6-phenylhexanoate (4br): The title compound was isolated by column chromatography with petroleum ether and ethyl acetate (PE/EA = 400:1) as a colorless oil (36.1 mg, 64% yield). ^{1}H NMR (400 MHz, $CDCl_3$) δ 7.39 – 7.30 (m, 5H), 7.29 – 7.25 (m, 2H), 7.19 – 7.13 (m, 3H), 5.11 (s, 2H), 2.59 (m, *J* = 7.7 Hz, 2H), 2.35 (m, *J* = 7.5 Hz, 2H), 1.71 – 1.59 (m, 4H), 1.41 – 1.33 (m, 2H). ^{13}C NMR (101 MHz, $CDCl_3$) δ 173.7, 142.6, 136.2, 128.7, 128.5, 128.4, 128.3, 125.8, 66.2, 35.8, 34.4, 31.2, 28.9, 24.9. IR (ATR): 3063, 3029, 2933, 2857, 1737, 1496, 1456, 1167, 747, 699 cm^{-1}. HRMS (EI): m/z $[M + Na]^+$ calcd for $C_{19}H_{22}O_2Na$: 305.1512, found 305.1515. This compound is known.[2]

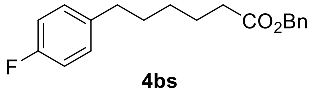

4bs

benzyl 6-(4-fluorophenyl)hexanoate (4bs): The title compound was isolated by column chromatography with petroleum ether and ethyl acetate (PE/EA = 400:1) as a colorless oil (33.6 mg, 56% yield). ^{1}H NMR (400 MHz, $CDCl_3$) δ 7.41 – 7.28 (m, 5H), 7.09 (dd, *J* = 8.4, 5.6 Hz, 2H), 6.94 (m, *J* = 8.7 Hz, 2H), 5.11 (s, 2H), 2.56 (m, *J* = 7.7 Hz, 2H), 2.35 (m, *J* = 7.5 Hz, 2H), 1.68 (m, *J* = 7.6 Hz, 2H), 1.62 – 1.56 (m, 2H), 1.39 – 1.31 (m, 2H). ^{13}C NMR (101 MHz, $CDCl_3$) δ 173.7, 161.3 (d, *J* = 241.6 Hz),

138.2 (d, *J* = 3.1 Hz), 136.2, 129.8 (d, *J* = 7.8 Hz), 128.7, 128.3, 115.2, 115.0, 66.3, 35.0, 34.3, 31.3, 28.7, 24.9. ^{19}F NMR (376 MHz, $CDCl_3$) δ -118.0. IR (ATR): 3039, 2932, 2862, 1736, 1603, 1511, 1455, 1220, 1161, 828, 751, 699 cm^{-1}. HRMS (EI): m/z $[M + Na]^+$ calcd for $C_{19}H_{21}FO_2Na$: 323.1418, found 305.1415.

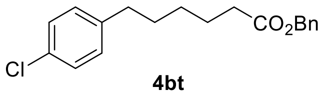

4bt

benzyl 6-(4-chlorophenyl)hexanoate (4bt): The title compound was isolated by column chromatography with petroleum ether and ethyl acetate (PE/EA = 400:1) as a colorless oil (36.6 mg, 58% yield). ^{1}H NMR (400 MHz, $CDCl_3$) δ 7.39 – 7.30 (m, 5H), 7.26 – 7.21 (m, 2H), 7.07 (d, *J* = 8.3 Hz, 2H), 5.11 (s, 2H), 2.56 (m, *J* = 7.7 Hz, 2H), 2.35 (m, *J* = 7.5 Hz, 2H), 1.72 – 1.64 (m, 2H), 1.64 – 1.57 (m, 2H), 1.40 – 1.31 (m, 2H). ^{13}C NMR (101 MHz, $CDCl_3$) δ 173.6, 141.0, 136.2, 131.5, 129.8, 128.7, 128.5, 128.3, 128.3, 66.3, 35.2, 34.3, 31.1, 28.7, 24.9. IR (ATR): 3033, 2934, 2858, 1736, 1493, 1458, 1382, 1167, 1091, 1013, 830, 748, 699 cm^{-1}. HRMS (EI): m/z $[M + Na]^+$ calcd for $C_{19}H_{21}ClO_2Na$: 339.1122, found 339.1122.

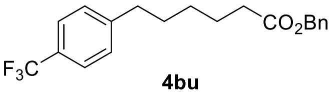

4bu

benzyl 6-(4-(trifluoromethyl)phenyl)hexanoate (4bu): The title compound was isolated by column chromatography with petroleum ether and ethyl acetate (PE/EA = 400:1) as a colorless oil (33.3 mg, 51% yield). ^{1}H NMR (400 MHz, $CDCl_3$) δ 7.51 (d, *J* = 8.0 Hz, 2H), 7.39 – 7.31 (m, 5H), 7.28 – 7.24 (m, 2H), 5.11 (s, 2H), 2.64 (m, *J* = 7.7 Hz, 2H), 2.36 (m, *J* = 7.5 Hz, 2H), 1.72 – 1.61 (m, 4H), 1.40 – 1.32 (m, 2H). ^{13}C NMR (101 MHz, $CDCl_3$) δ 173.6, 146.7, 136.2, 128.8, 128.7, 128.4, 128.3, 125.3 (q, *J* = 3.7 Hz), 124.5 (q, *J* = 270.0 Hz), 66.3, 35.7, 34.3, 30.9, 28.8, 24.8. ^{19}F NMR (376 MHz, $CDCl_3$) δ -62.2. IR (ATR): 3035, 2936, 2861, 1737, 1618, 1456, 1417, 1326, 1164, 1123, 1067, 1018, 843, 736, 698 cm-1. HRMS (EI): m/z $[M + H]^+$ calcd for $C_{20}H_{22}F_3O_2$: 351.1566, found 351.1562.

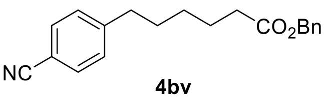

4bv

benzyl 6-(4-(trifluoromethyl)phenyl)hexanoate (4bv): The title compound was isolated by column chromatography with petroleum ether and ethyl acetate (PE/EA = 20:1) as a colorless oil (36.8 mg, 60% yield). ^{1}H NMR (400 MHz, $CDCl_3$) δ 7.55 (d, *J* = 7.9 Hz, 2H), 7.38 – 7.32 (m, 5H), 7.25 (d, *J* = 8.1 Hz, 2H), 5.11 (s, 2H), 2.65 (m, *J* = 7.7 Hz, 2H), 2.36 (m, *J* = 7.4 Hz, 2H), 1.70 – 1.60 (m, 4H), 1.39 – 1.30 (m, 2H). ^{13}C NMR (101 MHz, $CDCl_3$) δ 173.5, 148.2, 136.1, 132.3, 129.3, 128.7, 128.3, 128.3, 119.2, 109.7, 66.3, 35.9, 34.2, 30.7, 28.7, 24.8. IR (ATR): 3065, 3034, 2928, 2858, 2227, 1732, 1607,

1501, 1457, 1164, 843, 739, 698, 561 cm-1. HRMS (EI): m/z [M + H]$^+$ calcd for $C_{20}H_{22}NO_2$: 308.1645, found 308.1639.

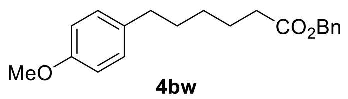

4bw

benzyl 6-(4-methoxyphenyl)hexanoate (4bw): The title compound was isolated by column chromatography with petroleum ether and ethyl acetate (PE/EA = 400:1) as a colorless oil (32.4 mg, 52% yield). ^{1}H NMR (400 MHz, $CDCl_3$) δ 7.39 – 7.29 (m, 5H), 7.07 (d, *J* = 8.6 Hz, 2H), 6.81 (d, *J* = 8.6 Hz, 2H), 5.11 (s, 2H), 3.78 (s, 3H), 2.56 – 2.50 (m, 2H), 2.35 (m, *J* = 7.5 Hz, 2H), 1.68 (m, *J* = 7.6 Hz, 2H), 1.61 – 1.56 (m, 2H), 1.39 – 1.31 (m, 2H). ^{13}C NMR (101 MHz, $CDCl_3$) δ 173.7, 157.8, 136.2, 134.7, 129.4, 128.7, 128.3, 113.8, 66.2, 55.4, 34.9, 34.4, 31.4, 28.8, 24.9. IR (ATR): 3032, 2932, 2856, 1736, 1611, 1584, 1512, 1460, 1382, 1298, 1246, 1176, 1036, 830, 749, 699 cm-1. HRMS (EI): m/z [M + Na]$^+$ calcd for $C_{20}H_{24}O_3Na$: 335.1618, found 335.1621.

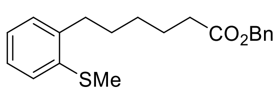

4bx

benzyl 6-(2-(methylthio)phenyl)hexanoate (4bx): The title compound was isolated by column chromatography with petroleum ether and ethyl acetate (PE/EA = 400:1) as a colorless oil (36.1 mg, 55% yield). ^{1}H NMR (400 MHz, $CDCl_3$) δ 7.34 (d, *J* = 5.5 Hz, 5H), 7.20 – 7.16 (m, 2H), 7.14 – 7.04 (m, 2H), 5.11 (s, 2H), 2.73 – 2.65 (m, 2H), 2.44 (s, 3H), 2.37 (m, *J* = 7.5 Hz, 2H), 1.74 – 1.66 (m, 2H), 1.65 – 1.60 (m, 2H), 1.45 – 1.36 (m, 2H). ^{13}C NMR (101 MHz, $CDCl_3$) δ 173.7, 140.4, 137.2, 136.2, 129.1, 128.7, 128.3, 126.7, 125.6, 125.0, 66.2, 34.4, 33.6, 29.6, 29.1, 24.9, 15.9. IR (ATR): 3061, 3034, 2930, 2856, 1736, 1594, 1498, 1468, 1435, 1166, 1073, 973, 746, 699, 679 cm^{-1}. HRMS (EI): m/z [M + Na]$^+$ calcd for $C_{20}H_{24}SO_2Na$: 351.1389, found 351.1382.

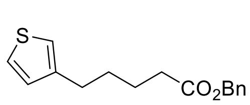

4by

benzyl 5-(thiophen-3-yl)pentanoate (4by): The title compound was isolated by column chromatography with petroleum ether and ethyl acetate (PE/EA = 50:1) as a colorless oil (22.5 mg, 41% yield). ^{1}H NMR (400 MHz, $CDCl_3$) δ 7.35 (d, *J* = 4.7 Hz, 5H), 7.23 (m, *J* = 3.9 Hz, 1H), 6.91 (d, *J* = 4.0 Hz, 2H), 5.11 (s, 2H), 2.64 (m, *J* = 7.0 Hz, 2H), 2.39 (m, *J* = 6.9 Hz, 2H), 1.73 – 1.64 (m, 4H). ^{13}C NMR (101 MHz, $CDCl_3$) δ 173.6, 142.5, 136.2, 128.7, 128.3, 128.3, 125.4, 120.2, 66.3, 34.2, 30.1, 30.0, 24.7. IR (ATR): 3034, 2934, 2859, 1734, 1456, 1382, 1166, 773, 748, 697, 632 cm^{-1}. HRMS (EI): m/z [M + H]$^+$ calcd for $C_{16}H_{19}O_2S$: 275.1100, found 275.1095.

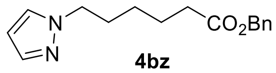
4bz

benzyl 6-(1H-pyrazol-1-yl)hexanoate (4bz): The title compound was isolated by column chromatography with petroleum ether and ethyl acetate (PE/EA = 5:1) as a colorless oil (35.4 mg, 65% yield). ^{1}H NMR (400 MHz, $CDCl_3$) δ 7.49 (d, *J* = 1.9 Hz, 1H), 7.35 – 7.31 (m, 6H), 6.22 (m, *J* = 2.1 Hz, 1H), 5.10 (s, 2H), 4.11 (m, *J* = 7.1 Hz, 2H), 2.35 (m, *J* = 7.4 Hz, 2H), 1.87 (dd, *J* = 8.5, 6.7 Hz, 2H), 1.71 – 1.63 (m, 2H), 1.34 – 1.27 (m, 2H). ^{13}C NMR (101 MHz, $CDCl_3$) δ 173.4, 139.2, 136.1, 129.0, 128.7, 128.3, 105.3, 66.3, 51.9, 34.2, 30.2, 26.2, 24.5. IR (ATR): 3034, 2942, 2865, 1733, 1513, 1498, 1455, 1396, 1167, 1091, 966, 751, 699, 620 cm^{-1}. HRMS (EI): m/z [M + H]$^+$ calcd for $C_{16}H_{21}N_2O_2$: 273.1598, found 273.1593.

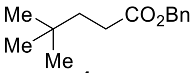
4ca

benzyl 4,4-dimethylpentanoate (4ca): The title compound was isolated by column chromatography with petroleum ether and ethyl acetate (PE/EA = 400:1) as a colorless oil (41.4 mg, 94% yield). ^{1}H NMR (400 MHz, $CDCl_3$) δ 7.40 – 7.31 (m, 5H), 5.11 (s, 2H), 2.36 – 2.31 (m, 2H), 1.60 – 1.57 (m, 2H), 0.89 (s, 9H). ^{13}C NMR (101 MHz, $CDCl_3$) δ 174.4, 136.2, 128.7, 128.4, 128.3, 66.3, 38.7, 30.3, 30.2, 29.2. IR (ATR): 3034, 2957, 2867, 1739, 1460, 1367, 1296, 1260, 1213, 1144, 747, 698 cm^{-1}. HRMS (EI): m/z [M + Na]$^+$ calcd for $C_{14}H_{20}O_2Na$: 243.1356, found 243.1358. This compound is known.[5]

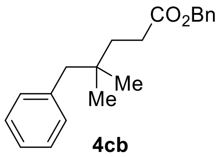
4cb

benzyl 4,4-dimethyl-5-phenylpentanoate (4cb): The title compound was isolated by column chromatography with petroleum ether and ethyl acetate (PE/EA = 400:1) as a colorless oil (32.6 mg, 55% yield). ^{1}H NMR (400 MHz, $CDCl_3$) δ 7.41 – 7.29 (m, 5H), 7.27 – 7.19 (m, 3H), 7.13 – 7.08 (m, 2H), 5.11 (s, 2H), 2.50 (s, 2H), 2.43 – 2.37 (m, 2H), 1.64 – 1.59 (m, 2H), 0.85 (s, 6H). ^{13}C NMR (101 MHz, $CDCl_3$) δ 174.2, 138.9, 136.2, 130.7, 128.7, 128.3, 127.9, 126.1, 66.4, 48.4, 36.8, 34.0, 29.9, 26.4. IR (ATR): 3030, 2959, 2932, 1736, 1453, 1292, 1159, 736, 701 cm^{-1}. HRMS (EI): m/z [M + Na]$^+$ calcd for $C_{20}H_{24}O_2Na$: 319.1669, found 319.1671. This compound is known.[5]

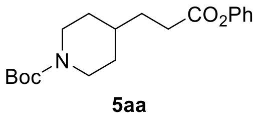
5aa

tert-butyl 4-(3-oxo-3-phenoxypropyl)piperidine-1-carboxylate (5aa): The title compound was isolated by column chromatography with petroleum ether and ethyl acetate (PE/EA = 10:1) as a colorless oil (47.3 mg, 71% yield). ^{1}H NMR (400 MHz, $CDCl_3$) δ 7.38 (dd, *J* = 8.4, 7.4 Hz, 2H), 7.26 – 7.20 (m, 1H), 7.10 – 7.03 (m, 2H), 4.11 (s, 2H), 2.68 (d, *J* = 12.6 Hz, 2H), 2.59 (m, *J* = 7.7 Hz, 2H), 1.72 (m, *J* = 7.0 Hz, 4H), 1.51 (m, *J* = 7.3, 3.8 Hz, 1H), 1.46 (s, 9H), 1.15 (m, *J* = 12.4, 4.4 Hz, 2H). ^{13}C NMR (101 MHz,

$CDCl_3$) δ 172.3, 155.0, 150.8, 129.6, 125.9, 121.6, 79.5, 44.0, 35.7, 31.9, 31.8, 31.5, 28.6. IR (ATR): 2975, 2928, 2853, 1761, 1692, 1594, 1423, 1366, 1277, 1244, 1164, 969, 932, 867, 753, 691 cm^{-1}. HRMS (EI): m/z $[M + Na]^+$ calcd for $C_{19}H_{27}NO_4Na$: 356.1832, found 356.1829.

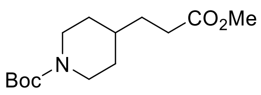

5ab

tert-butyl 4-(3-methoxy-3-oxopropyl)piperidine-1-carboxylate (5ab): The title compound was isolated by column chromatography with petroleum ether and ethyl acetate (PE/EA = 10:1) as a colorless oil (34.1 mg, 63% yield). ^{1}H NMR (400 MHz, $CDCl_3$) δ 4.08 (s, 2H), 3.67 (s, 3H), 2.73 – 2.60 (m, 2H), 2.34 (m, *J* = 7.7 Hz, 2H), 1.62 (dd, *J* = 24.7, 10.4 Hz, 5H), 1.45 (s, 9H), 1.11 (m, *J* = 12.3, 4.4 Hz, 2H). ^{13}C NMR (101 MHz, $CDCl_3$) δ 174.2, 154.9, 79.3, 51.6, 43.9, 35.6, 31.9, 31.5, 31.4, 28.6. IR (ATR): 2975, 2929, 2852, 1741, 1694, 1424, 1366, 1278, 1246, 1165, 1125, 982, 866, 769 cm^{-1}. HRMS (EI): m/z $[M + H]^+$ calcd for $C_{14}H_{26}NO_4$: 272.1856, found 272.1856.

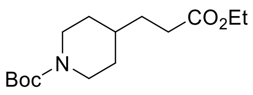

5ac

tert-butyl 4-(3-ethoxy-3-oxopropyl)piperidine-1-carboxylate (5ac): The title compound was isolated by column chromatography with petroleum ether and ethyl acetate (PE/EA = 10:1) as a colorless oil (37.0 mg, 65% yield). ^{1}H NMR (400 MHz, $CDCl_3$) δ 4.13 (m, *J* = 7.1 Hz, 4H), 2.76 – 2.58 (m, 2H), 2.35 – 2.29 (m, 2H), 1.70 – 1.55 (m, 4H), 1.45 (s, 9H), 1.39 (m, *J* = 10.9, 7.3, 3.9 Hz, 1H), 1.26 (m, *J* = 7.1 Hz, 3H), 1.09 (m, *J* = 12.4, 4.4 Hz, 2H). ^{13}C NMR (101 MHz, $CDCl_3$) δ 173.8, 155.0, 79.4, 60.5, 44.0, 35.6, 31.9, 31.7, 31.5, 28.6, 14.4. IR (ATR): 2975, 2932, 2851, 1738, 1694, 1424, 1366, 1280, 1248, 1171, 971, 866, 768 cm^{-1}. HRMS (EI): m/z $[M + Na]^+$ calcd for $C_{15}H_{27}NO_4Na$: 308.1832, found 308.1833. This compound is known.[6]

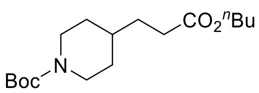

5ad

tert-butyl 4-(3-butoxy-3-oxopropyl)piperidine-1-carboxylate (5ad): The title compound was isolated by column chromatography with petroleum ether and ethyl acetate (PE/EA = 10:1) as a colorless oil (40.1 mg, 64% yield). ^{1}H NMR (400 MHz, $CDCl_3$) δ 4.07 (m, *J* = 6.7 Hz, 4H), 2.66 (m, *J* = 11.8 Hz, 2H), 2.33 (m, *J* = 7.7 Hz, 2H), 1.69 – 1.56 (m, 6H), 1.45 (s, 9H), 1.37 (m, *J* = 14.6, 7.5 Hz, 3H), 1.09 (m, *J* = 12.4, 4.4 Hz, 2H), 0.94 (m, *J* = 7.4 Hz, 3H). ^{13}C NMR (101 MHz, $CDCl_3$) δ 173.9, 154.9, 79.4, 64.4, 44.0, 35.6, 31.9, 31.7, 31.6, 30.8, 28.6, 19.2, 13.8. IR (ATR): 2962, 2932, 2869, 1736, 1695, 1423, 1366, 1277, 1246, 1165, 1124, 970, 867, 769 cm^{-1}. HRMS (EI): m/z $[M + H]^+$ calcd for $C_{17}H_{32}NO_4$: 314.2326, found 314.2326.

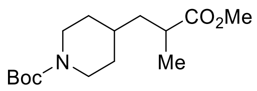

5ae

tert-butyl 4-(3-methoxy-2-methyl-3-oxopropyl)piperidine-1-carboxylate (5ae): The title compound was isolated by column chromatography with petroleum ether and ethyl acetate (PE/EA = 10:1) as a colorless oil (28.5 mg, 50% yield, mixture of rotamers). Data of one isomer: ^{1}H NMR (400 MHz, $CDCl_3$) δ 4.04 (s, 2H), 3.66 (s, 3H), 2.64 (m, *J* = 12.6 Hz, 2H), 2.54 (m, *J* = 8.3, 6.6 Hz, 1H), 1.77 – 1.54 (m, 4H), 1.43 (s, 9H), 1.28 – 1.23 (m, 1H), 1.14 (s, 3H), 1.09 – 0.99 (m, 2H). ^{13}C NMR (101 MHz, $CDCl_3$) δ 177.4, 155.0, 79.4, 51.7, 44.0, 40.7, 36.7, 34.0, 32.2, 28.6, 17.8. Data of other isomer: ^{1}H NMR (400 MHz, $CDCl_3$) δ 4.04 (s, 2H), 3.66 (s, 3H), 2.64 (m, *J* = 12.6 Hz, 2H), 2.54 (m, *J* = 8.3, 6.6 Hz, 1H), 1.77 – 1.54 (m, 4H), 1.43 (s, 9H), 1.28 – 1.23 (m, 1H), 1.12 (s, 3H), 1.09 – 0.99 (m, 2H). ^{13}C NMR (101 MHz, $CDCl_3$) δ 177.4, 155.0, 79.4, 51.7, 44.0, 40.7, 36.7, 34.0, 32.1, 28.6, 17.8. IR (ATR): 2975, 2932, 2851, 1738, 1694, 1424, 1366, 1280, 1248, 1171, 971, 866, 768 cm^{-1}. HRMS (EI): m/z $[M + Na]^+$ calcd for $C_{15}H_{27}NO_4Na$: 308.1832, found 308.1833.

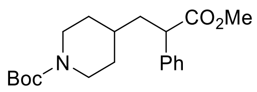

5af

tert-butyl 4-(3-methoxy-3-oxo-2-phenylpropyl)piperidine-1-carboxylate (5af): The title compound was isolated by column chromatography with petroleum ether and ethyl acetate (PE/EA = 10:1) as a colorless oil (44.4 mg, 64% yield, mixture of rotamers). Data of both isomer: ^{1}H NMR (400 MHz, $CDCl_3$) δ 7.35 – 7.27 (m, 5H), 4.04 (s, 2H), 3.69 (d, *J* = 7.8 Hz, 1H), 3.65 (s, 3H), 2.60 (m, *J* = 12.7 Hz, 2H), 2.10 – 1.96 (m, 1H), 1.77 – 1.62 (m, 3H), 1.44 (s, 9H), 1.33 – 1.25 (m, 1H), 1.12 (m, *J* = 12.2, 4.3 Hz, 2H). Data of one isomer: ^{13}C NMR (101 MHz, $CDCl_3$) δ 174.5, 154.9, 139.1, 128.8, 128.0, 127.5, 79.4, 52.2, 48.7, 43.9, 40.2, 33.9, 32.2, 28.6. Data of other isomer: ^{13}C NMR (101 MHz, $CDCl_3$) δ 174.5, 154.9, 139.1, 128.8, 128.0, 127.5, 79.4, 52.2, 48.7, 43.9, 40.2, 33.9, 32.0, 28.6. IR (ATR): 2930, 2850, 1737, 1692, 1425, 1365, 1278, 1245, 1166, 1127, 1006, 978, 921, 866, 770, 734, 700, 647 cm^{-1}. HRMS (EI): m/z $[M + H]^+$ calcd for $C_{20}H_{30}NO_4$: 348.2169, found 348.2171.

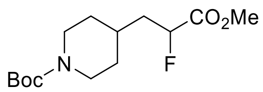

5ag

tert-butyl 4-(2-fluoro-3-methoxy-3-oxopropyl)piperidine-1-carboxylate (5ag): The title compound was isolated by column chromatography with petroleum ether and ethyl acetate (PE/EA = 10:1) as a colorless oil (50.9 mg, 88% yield, mixture of rotamers). Data of one isomer: ^{1}H NMR (400 MHz, $CDCl_3$) δ 5.04 (dd, *J* = 9.2, 3.5 Hz, 1H), 4.10 (s, 2H), 3.80 (s, 3H), 2.72 (d, *J* = 12.9 Hz, 2H), 1.96 – 1.74 (m, 3H), 1.72 – 1.62 (m, 2H), 1.45 (s, 9H), 1.24 – 1.11 (m, 2H). ^{13}C NMR (101 MHz, $CDCl_3$) δ 170.6 (d, *J* = 23.7 Hz), 154.9, 87.3 (d, *J* = 183.4 Hz), 79.5, 52.5, 43.7, 39.1, 32.5, 32.3, 28.6. Data of other isomer: ^{1}H

NMR (400 MHz, $CDCl_3$) δ 4.92 (dd, *J* = 9.3, 2.9 Hz, 1H), 4.10 (s, 2H), 3.80 (s, 3H), 2.72 (d, *J* = 12.9 Hz, 2H), 1.96 – 1.74 (m, 3H), 1.72 – 1.62 (m, 2H), 1.45 (s, 9H), 1.24 – 1.11 (m, 2H). ^{13}C NMR (101 MHz, $CDCl_3$) δ 170.6 (d, *J* = 23.7 Hz), 154.9, 87.3 (d, *J* = 183.4 Hz), 79.5, 52.5, 43.7, 38.9, 32.3, 31.4, 28.6. ^{19}F NMR (376 MHz, $CDCl_3$) δ -191.2. IR (ATR): 2926, 2853, 1766, 1745, 1692, 1426, 1366, 1280, 1248, 1171, 1137, 1087, 1019, 973, 870, 770 cm^{-1}. HRMS (EI): m/z [M + H]$^+$ calcd for $C_{14}H_{25}FNO_4$: 290.1762, found 290.1763.

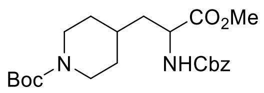

5ah

tert-butyl 4-(2-(((benzyloxy)carbonyl)amino)-3-methoxy-3-oxopropyl) piperidine-1-carboxylate (5ah): The title compound was isolated by column chromatography with petroleum ether and ethyl acetate (PE/EA = 5:1) as a colorless oil (50.9 mg, 63% yield). Data of one isomer: ^{1}H NMR (400 MHz, $CDCl_3$) δ 7.37 – 7.30 (m, 5H), 5.24 (d, *J* = 8.7 Hz, 1H), 5.10 (d, *J* = 4.9 Hz, 2H), 4.43 (m, *J* = 9.0, 4.9 Hz, 1H), 4.14 – 3.97 (m, 2H), 3.73 (s, 3H), 2.64 (s, 2H), 1.73 (s, 2H), 1.56 (m, *J* = 20.3, 9.9, 4.7 Hz, 3H), 1.44 (s, 9H), 1.11 (dd, *J* = 21.2, 9.1 Hz, 2H). ^{13}C NMR (101 MHz, $CDCl_3$) δ 173.4, 156.1, 154.9, 136.3, 128.7, 128.4, 128.2, 79.5, 67.2, 52.6, 51.7, 43.8, 39.7, 32.4, 31.5, 28.6. Data of the other isomer: ^{1}H NMR (400 MHz, $CDCl_3$) δ 7.37 – 7.30 (m, 5H), 5.24 (d, *J* = 8.7 Hz, 1H), 5.10 (d, *J* = 4.9 Hz, 2H), 4.43 (m, *J* = 9.0, 4.9 Hz, 1H), 4.14 – 3.97 (m, 2H), 3.73 (s, 3H), 2.64 (s, 2H), 1.73 (s, 2H), 1.56 (m, *J* = 20.3, 9.9, 4.7 Hz, 3H), 1.44 (s, 9H), 1.11 (dd, *J* = 21.2, 9.1 Hz, 2H). ^{13}C NMR (101 MHz, $CDCl_3$) δ 173.4, 156.1, 154.9, 136.3, 128.7, 128.4, 128.2, 79.5, 67.2, 52.6, 51.7, 43.8, 39.7, 32.6, 31.5, 28.6. IR (ATR): 3319, 2930, 2852, 1724, 1694, 1532, 1431, 1366, 1278, 1242, 1164, 1048, 740, 698 cm^{-1}. HRMS (EI): m/z [M + H]$^+$ calcd for $C_{22}H_{33}N_2O_6$: 421.2333, found 421.2329.

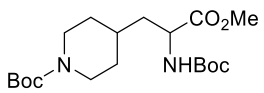

5ai

tert-butyl 4-(2-((tert-butoxycarbonyl)amino)-3-methoxy-3-oxopropyl) piperidine-1-carboxylate (5ai): The title compound was isolated by column chromatography with petroleum ether and ethyl acetate (PE/EA = 5:1) as a colorless oil (41.7 mg, 54% yield). Data of one isomer: ^{1}H NMR (400 MHz, $CDCl_3$) δ 4.94 (d, *J* = 8.9 Hz, 1H), 4.35 (m, *J* = 7.9 Hz, 1H), 4.08 (dd, *J* = 16.0, 8.9 Hz, 2H), 3.72 (s, 3H), 2.63 (d, *J* = 15.2 Hz, 2H), 1.71 (dd, *J* = 25.5, 6.7 Hz, 2H), 1.52 (m, *J* = 9.7, 7.5 Hz, 2H), 1.43 (d, *J* = 2.1 Hz, 18H), 1.28 – 1.21 (m, 1H), 1.10 (m, *J* = 19.5, 11.2, 4.1 Hz, 2H). ^{13}C NMR (101 MHz, $CDCl_3$) δ 173.8, 155.5, 154.9, 80.2, 79.5, 52.4, 51.3, 43.9, 39.8, 32.4, 31.5, 28.6, 28.4. Data of the other isomer: ^{1}H NMR (400 MHz, $CDCl_3$) δ 4.94 (d, *J* = 8.9 Hz, 1H), 4.35 (m, *J* = 7.9 Hz, 1H), 4.08 (dd, *J* = 16.0, 8.9 Hz, 2H), 3.72 (s, 3H), 2.63 (d, *J* = 15.2 Hz, 2H), 1.71 (dd, *J* = 25.5, 6.7 Hz, 2H), 1.52 (m, *J* = 9.7, 7.5

Hz, 2H), 1.43 (d, J = 2.1 Hz, 18H), 1.28 – 1.21 (m, 1H), 1.10 (m, J = 19.5, 11.2, 4.1 Hz, 2H). ^{13}C NMR (101 MHz, $CDCl_3$) δ 173.8, 155.5, 154.9, 80.2, 79.5, 52.4, 51.3, 43.9, 39.8, 32.7, 31.5, 28.6, 28.4. IR (ATR): 3339, 2977, 2930, 2853, 1748, 1695, 1522, 1429, 1366, 1166, 1020, 864, 733 cm^{-1}. HRMS (EI): m/z $[M + H]^+$ calcd for $C_{19}H_{35}N_2O_6$: 387.2490, found 387.2488.

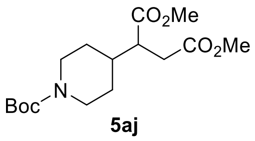

5aj

dimethyl 2-(1-(tert-butoxycarbonyl)piperidin-4-yl)succinate (5aj): The title compound was isolated by column chromatography with petroleum ether and ethyl acetate (PE/EA = 10:1) as a white solid (59.2 mg, 90% yield mixture of rotamers). Data of one isomer: ^{1}H NMR (400 MHz, $CDCl_3$) δ 4.09 (s, 2H), 3.66 (s, 3H), 3.63 (s, 3H), 2.79 – 2.66 (m, 2H), 2.61 (dd, J = 23.2, 10.8 Hz, 2H), 2.43 (d, J = 2.1 Hz, 1H), 1.70 (m, J = 14.6, 8.9, 3.0 Hz, 1H), 1.59 – 1.49 (m, 2H), 1.40 (s, 9H), 1.24 – 1.13 (m, 2H). ^{13}C NMR (101 MHz, $CDCl_3$) δ 174.4, 172.5, 154.7, 79.5, 51.9, 46.3, 43.9, 38.3, 33.3, 29.6, 29.3, 28.5. Data of the other isomer: ^{1}H NMR (400 MHz, $CDCl_3$) δ 4.09 (s, 2H), 3.66 (s, 3H), 3.63 (s, 3H), 2.79 – 2.66 (m, 2H), 2.61 (dd, J = 23.2, 10.8 Hz, 2H), 2.40 (d, J = 2.7 Hz, 1H), 1.70 (m, J = 14.6, 8.9, 3.0 Hz, 1H), 1.59 – 1.49 (m, 2H), 1.40 (s, 9H), 1.24 – 1.13 (m, 2H). ^{13}C NMR (101 MHz, $CDCl_3$) δ 174.4, 172.5, 154.7, 79.5, 51.8, 46.3, 43.9, 38.3, 33.3, 29.6, 29.3, 28.5. IR (ATR): 2970, 2956, 2941, 1736, 1720, 1676, 1474, 1435, 1367, 1293, 1231, 1172, 1142, 936, 870, 769 cm^{-1}. HRMS (EI): m/z $[M + H]^+$ calcd for $C_{16}H_{28}NO_6$: 330.1911, found 330.1911.

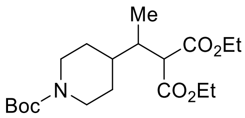

5ak

diethyl 2-(1-(1-(tert-butoxycarbonyl)piperidin-4-yl)ethyl)malonate (5ak): The title compound was isolated by column chromatography with petroleum ether and ethyl acetate (PE/EA = 10:1) as a colorless oil (60.1 mg, 81% yield mixture of rotamers). Data of one isomer: ^{1}H NMR (400 MHz, $CDCl_3$) δ 4.28 – 4.09 (m, 6H), 3.40 (d, J = 8.7 Hz, 1H), 2.72 – 2.47 (m, 2H), 2.22 (m, J = 8.6, 6.8, 4.4, 1.9 Hz, 1H), 1.63 – 1.53 (m, 2H), 1.47 (s, 1H), 1.45 (s, 9H), 1.27 (m, J = 7.1, 0.6 Hz, 7H), 1.15 (m, J = 12.7, 4.9 Hz, 1H), 0.92 (s, 3H). ^{13}C NMR (101 MHz, $CDCl_3$) δ 169.2, 154.9, 79.5, 61.5, 55.5, 44.1, 38.9, 37.9, 30.5, 28.6, 14.3, 13.2. Data of the other isomer: ^{1}H NMR (400 MHz, $CDCl_3$) δ 4.28 – 4.09 (m, 6H), 3.40 (d, J = 8.7 Hz, 1H), 2.72 – 2.47 (m, 2H), 2.22 (m, J = 8.6, 6.8, 4.4, 1.9 Hz, 1H), 1.63 – 1.53 (m, 2H), 1.47 (s, 1H), 1.45 (s, 9H), 1.27 (m, J = 7.1, 0.6 Hz, 7H), 1.15 (m, J = 12.7, 4.9 Hz, 1H), 0.90 (s, 3H). ^{13}C NMR (101 MHz, $CDCl_3$) δ 168.8, 154.9, 79.5, 61.4, 55.5, 44.1, 38.9, 37.9, 28.6, 28.5, 14.3, 13.2. IR (ATR): 2978, 2938, 2855, 1754, 1732, 1424, 1367, 1280, 1236, 1175, 1033, 866, 769 cm^{-1}. HRMS (EI): m/z $[M + H]^+$ calcd for $C_{19}H_{34}NO_6$: 372.2381, found 372.2383.

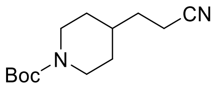

5al

tert-butyl 4-(2-cyanoethyl)piperidine-1-carboxylate (5al): The title compound was isolated by column chromatography with petroleum ether and ethyl acetate (PE/EA = 10:1) as a colorless oil (33.8 mg, 71% yield). ^{1}H NMR (400 MHz, $CDCl_3$) δ 4.12 (s, 2H), 2.82 – 2.63 (m, 2H), 2.39 (m, *J* = 7.0 Hz, 2H), 1.72 – 1.67 (m, 1H), 1.67 – 1.60 (m, 4H), 1.46 (s, 9H), 1.17 – 1.05 (m, 2H). ^{13}C NMR (101 MHz, $CDCl_3$) δ 154.9, 119.7, 79.6, 43.7, 35.1, 31.8, 31.5, 28.6, 14.7. IR (ATR): 2976, 2930, 2859, 2245, 1692, 1425, 1366, 1277, 1247, 1168, 1128, 966, 864, 769 cm^{-1}. HRMS (EI): m/z $[M + H]^+$ calcd for $C_{13}H_{23}N_2O_2H$: 239.1754, found 239.1755.

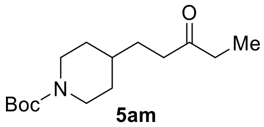

5am

tert-butyl 4-(3-oxopentyl)piperidine-1-carboxylate (5am): The title compound was isolated by column chromatography with petroleum ether and ethyl acetate (PE/EA = 10:1) as a colorless oil (33.3 mg, 62% yield). ^{1}H NMR (400 MHz, $CDCl_3$) δ 4.07 (s, 2H), 2.66 (m, *J* = 12.9 Hz, 2H), 2.46 – 2.40 (m, 4H), 1.63 (d, *J* = 13.1 Hz, 2H), 1.53 (m, *J* = 7.3 Hz, 2H), 1.45 (s, 9H), 1.36 (m, *J* = 11.2, 7.5, 3.9 Hz, 1H), 1.14 – 1.07 (m, 2H), 1.05 (d, *J* = 7.3 Hz, 3H). ^{13}C NMR (101 MHz, $CDCl_3$) δ 211.6, 154.9, 79.3, 44.0, 39.5, 36.0, 35.7, 32.1, 30.3, 28.6, 8.0. IR (ATR): 2976, 2932, 2851, 1693, 1423, 1365, 1277, 1247, 1167, 1133, 962, 869, 769 cm^{-1}. HRMS (EI): m/z $[M + H]^+$ calcd for $C_{15}H_{28}NO_3$: 270.2064, found 270.2064.

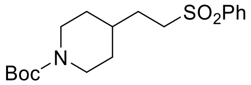

5an

tert-butyl 4-(2-(phenylsulfonyl)ethyl)piperidine-1-carboxylate (5an): The title compound was isolated by column chromatography with petroleum ether and ethyl acetate (PE/EA = 10:1) as a white solid. (55.1 mg, 78% yield). ^{1}H NMR (400 MHz, $CDCl_3$) δ 7.89 (d, *J* = 7.7 Hz, 2H), 7.65 (m, *J* = 7.4 Hz, 1H), 7.56 (m, *J* = 7.6 Hz, 2H), 4.04 (s, 2H), 3.11 – 3.05 (m, 2H), 2.61 (s, 2H), 1.65 (dd, *J* = 10.2, 5.6 Hz, 2H), 1.57 (d, *J* = 13.0 Hz, 2H), 1.48 – 1.43 (m, 1H), 1.42 (s, 9H), 1.09 – 0.99 (m, 2H). ^{13}C NMR (101 MHz, $CDCl_3$) δ 154.8, 139.2, 133.9, 129.4, 128.1, 79.5, 54.0, 43.8, 35.0, 31.7, 29.0, 28.5. IR (ATR): 2975, 2930, 2853, 1689, 1448, 1424, 1366, 1307, 1246, 1147, 1087, 866, 740, 691 cm^{-1}. HRMS (EI): m/z $[M + Na]^+$ calcd for $C_{18}H_{27}NSO_4Na$: 376.1553, found 376.1553.

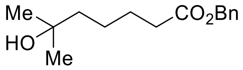

4da

benzyl 6-hydroxy-6-methylheptanoate (4da): The title compound was isolated by column chromatography with petroleum ether and ethyl acetate (PE/EA = 5:1) as a colorless oil (39.0 mg, 78% yield). ^{1}H NMR (400 MHz,

CDCl3) δ 7.36 (d, *J* = 3.7 Hz, 5H), 5.12 (s, 2H), 2.39 (m, *J* = 7.5 Hz, 2H), 1.70 – 1.63 (m, 2H), 1.51 – 1.44 (m, 2H), 1.42 – 1.35 (m, 2H), 1.19 (s, 6H). ^{13}C NMR (101 MHz, $CDCl_3$) δ 173.6, 136.2, 128.7, 128.4, 128.3, 71.0, 66.3, 43.6, 34.4, 29.4, 25.6, 24.0. IR (ATR): 3514, 3034, 2963, 2933, 2864, 1735, 1498, 1456, 1383, 1165, 954, 908, 750, 699 cm^{-1}. HRMS (EI): m/z [M + Na]$^+$ calcd for $C_{15}H_{22}O_3Na$: 273.1461, found 273.1460.

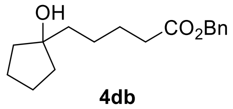

4db

benzyl 5-(1-hydroxycyclopentyl)pentanoate (4db): The title compound was isolated by column chromatography with petroleum ether and ethyl acetate (PE/EA = 5:1) as a colorless oil (41.9 mg, 76% yield). ^{1}H NMR (400 MHz, $CDCl_3$) δ 7.37 – 7.33 (m, 5H), 5.12 (s, 2H), 2.39 (m, *J* = 7.5 Hz, 2H), 1.78 (s, 2H), 1.67 (m, *J* = 7.5 Hz, 3H), 1.60 (m, *J* = 8.1, 4.5, 2.7 Hz, 5H), 1.54 – 1.40 (m, 4H). ^{13}C NMR (101 MHz, $CDCl_3$) δ 173.7, 136.2, 128.7, 128.4, 128.3, 82.5, 66.3, 41.2, 39.8, 34.4, 25.6, 24.4, 23.9. IR (ATR): 3515, 3034, 2954, 2868, 1735, 1498, 1455, 1385, 1162, 982, 750, 698 cm^{-1}. HRMS (EI): m/z [M + Na]$^+$ calcd for $C_{17}H_{24}O_3Na$: 299.1618, found 299.1618.

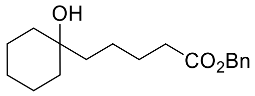

4dc

benzyl 5-(1-hydroxycyclohexyl)pentanoate (4dc): The title compound was isolated by column chromatography with petroleum ether and ethyl acetate (PE/EA = 5:1) as a colorless oil (42.3 mg, 73% yield). ^{1}H NMR (400 MHz, $CDCl_3$) δ 7.37 – 7.31 (m, 5H), 5.11 (s, 2H), 2.38 (m, *J* = 7.5 Hz, 2H), 1.69 – 1.61 (m, 3H), 1.58 – 1.33 (m, 13H). ^{13}C NMR (101 MHz, $CDCl_3$) δ 173.7, 136.2, 128.7, 128.4, 128.3, 71.4, 66.2, 42.1, 37.5, 34.5, 25.9, 25.7, 22.6, 22.4. IR (ATR): 3535, 3065, 3034, 2929, 2853, 1736, 1497, 1455, 1159, 966, 749, 698 cm^{-1}. HRMS (EI): m/z [M + Na]$^+$ calcd for $C_{18}H_{26}O_3Na$: 313.1774, found 313.1776.

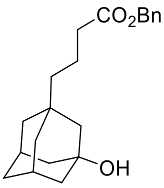

4dd

benzyl 4-((1s,3s,5R,7S)-3-hydroxyadamantan-1-yl)butanoate (4dd): The title compound was isolated by column chromatography with petroleum ether and ethyl acetate (PE/EA = 5:1) as a colorless oil (34.1 mg, 52% yield). ^{1}H NMR (400 MHz, $CDCl_3$) δ 7.40 – 7.30 (m, 5H), 5.11 (s, 2H), 2.32 (m, *J* = 7.5 Hz, 2H), 2.17 (m, *J* = 3.1 Hz, 2H), 1.70 – 1.62 (m, 4H), 1.61 – 1.58 (m, 2H), 1.52 (m, *J* = 3.3, 1.7 Hz, 2H), 1.42 – 1.34 (m, 6H), 1.17 – 1.11 (m, 2H). ^{13}C NMR (101 MHz, $CDCl_3$) δ 173.7, 136.2, 128.7, 128.4, 128.3, 69.1, 66.2, 50.1, 44.9, 43.0, 41.0, 36.2, 35.7, 35.1, 30.8, 18.6. IR (ATR): 3381, 3034, 2908, 2848, 1736, 1498, 1454, 1339, 1152, 1058, 909, 747, 699 cm^{-1}. HRMS (EI): m/z [M + Na]$^+$ calcd for $C_{21}H_{28}O_3Na$: 351.1931, found 351.1934.

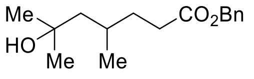

4de

benzyl 6-hydroxy-4,6-dimethylheptanoate (4de): The title compound was isolated by column chromatography with petroleum ether and ethyl acetate (PE/EA = 5:1) as a colorless oil (43.8 mg, 83% yield). ^{1}H NMR (600 MHz, $CDCl_3$) δ 7.37 – 7.31 (m, 5H), 5.11 (s, 2H), 2.44 – 2.34 (m, 2H), 1.77 (m, *J* = 13.9, 8.8, 6.9, 5.4 Hz, 1H), 1.68 – 1.63 (m, 1H), 1.53 – 1.46 (m, 2H), 1.32 (dd, *J* = 14.2, 6.4 Hz, 1H), 1.21 (s, 6H), 0.97 (d, *J* = 6.7 Hz, 3H). ^{13}C NMR (101 MHz, $CDCl_3$) δ 174.1, 136.2, 128.7, 128.4, 128.3, 71.4, 66.3, 50.3, 33.5, 32.1, 30.1, 30.0, 28.8, 21.8. IR (ATR): 3514, 3034, 2963, 2927, 1735, 1498, 1455, 1382, 1165, 962, 750, 698 cm^{-1}. HRMS (EI): m/z [M + Na]$^+$ calcd for $C_{16}H_{24}O_3Na$: 287.1618, found 287.1617.

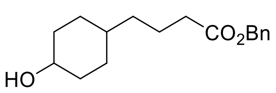

4df

benzyl 4-(4-hydroxycyclohexyl)butanoate (4df): The title compound was isolated by column chromatography with petroleum ether and ethyl acetate (PE/EA = 5:1) as a colorless oil (35.3 mg, 64% yield). The major isomer: ^{1}H NMR (400 MHz, $CDCl_3$) δ 7.34 (d, *J* = 7.2 Hz, 5H), 5.11 (s, 2H), 3.94 (m, *J* = 4.1 Hz, 1H), 2.34 (m, *J* = 7.7, 4.9 Hz, 2H), 1.95 (d, *J* = 11.9 Hz, 1H), 1.80 – 1.60 (m, 4H), 1.58 – 1.40 (m, 4H), 1.39 – 1.15 (m, 4H). ^{13}C NMR (101 MHz, $CDCl_3$) δ 173.8, 136.2, 128.7, 128.4, 128.3, 67.2, 66.2, 36.1, 35.6, 34.6, 32.3, 27.0, 22.6.The minor isomer: ^{1}H NMR (400 MHz, $CDCl_3$) δ 7.34 (d, *J* = 7.2 Hz, 5H), 5.11 (s, 2H), 3.52 (m, *J* = 4.1 Hz, 1H), 2.34 (m, *J* = 7.7, 4.9 Hz, 2H), 1.95 (d, *J* = 11.9 Hz, 1H), 1.80 – 1.60 (m, 4H), 1.58 – 1.40 (m, 4H), 1.39 – 1.15 (m, 4H). ^{13}C NMR (101 MHz, $CDCl_3$) δ 173.7, 136.2, 128.7, 128.4, 128.3, 71.2, 66.4, 36.5, 36.1, 35.5, 32.1, 31.2, 22.7. IR (ATR): 3415, 3033, 2925, 2853, 1732, 1498, 1455, 1383, 1256, 1155, 1033, 965, 751, 698 cm^{-1}. HRMS (EI): m/z [M + Na]$^+$ calcd for $C_{17}H_{24}O_3Na$: 299.1618, found 299.1612.

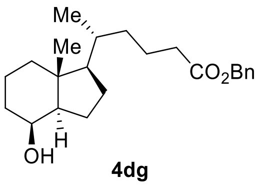

4dg

benzyl (*R*)-5-((1*R*,3a*R*,4*S*,7a*R*)-4-hydroxy-7a-methyloctahydro-1H-inden-1-yl)hexanoate (4dg): The title compound was isolated by column chromatography with petroleum ether and ethyl acetate (PE/EA = 5:1) as a colorless oil (35.8 mg, 50% yield). ^{1}H NMR (400 MHz, $CDCl_3$) δ 7.38 – 7.33 (m, 5H), 5.11 (s, 2H), 4.07 (m, *J* = 2.8 Hz, 1H), 2.38 – 2.27 (m, 2H), 1.98 (m, *J* = 13.3, 3.1 Hz, 1H), 1.84 – 1.76 (m, 3H), 1.56 – 1.45 (m, 3H), 1.44 – 1.34 (m, 4H), 1.33 – 1.14 (m, 4H), 1.12 – 1.04 (m, 2H), 0.91 (s, 3H), 0.90 (d, *J* = 6.6 Hz, 3H). ^{13}C NMR (101 MHz, $CDCl_3$) δ 173.8, 136.3, 128.7, 128.4, 128.3, 69.5, 66.2, 56.5, 52.7, 42.0, 40.5, 35.3, 35.1, 34.9, 33.7, 27.2, 22.6, 21.6, 18.6, 17.6,

13.6. IR (ATR): 3542, 3034, 2940, 2872, 1736, 1498, 1456, 1380, 1350, 1243, 1164, 992, 748, 698 cm^{-1}. HRMS (EI): m/z $[M + Na]^+$ calcd for $C_{23}H_{34}O_3Na$: 381.2400, found 381.2404.

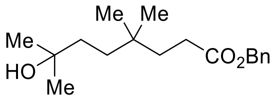

4dh

benzyl 7-hydroxy-4,4,7-trimethyloctanoate (4dh): The title compound was isolated by column chromatography with petroleum ether and ethyl acetate (PE/EA = 5:1) as a colorless oil (35.6 mg, 61% yield). ^1H NMR (400 MHz, $CDCl_3$) δ 7.38 – 7.32 (m, 5H), 5.11 (s, 2H), 2.35 – 2.29 (m, 2H), 1.61 – 1.54 (m, 2H), 1.44 – 1.38 (m, 2H), 1.26 – 1.23 (m, 2H), 1.20 (s, 6H), 0.86 (s, 6H). ^{13}C NMR (101 MHz, $CDCl_3$) δ 174.4, 136.2, 128.7, 128.4, 128.3, 71.0, 66.4, 37.9, 36.4, 35.7, 32.2, 29.7, 29.4, 26.9. IR (ATR): 3454, 3035, 2962, 2868, 1736, 1459, 1383, 1296, 1156, 970, 912, 748, 699 cm^{-1}. HRMS (EI): m/z $[M + Na]^+$ calcd for $C_{18}H_{28}O_3Na$: 315.1931, found 315.1934.

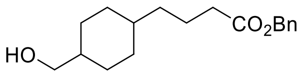

4di

benzyl 4-(4-(hydroxymethyl)cyclohexyl)butanoate (4di): The title compound was isolated by column chromatography with petroleum ether and ethyl acetate (PE/EA = 5:1) as a colorless oil (39.4 mg, 68% yield, mixture of cis and trans). The major isomer: ^1H NMR (400 MHz, $CDCl_3$) δ 7.43 – 7.27 (m, 5H), 5.11 (s, 2H), 3.44 (d, *J* = 6.3 Hz, 2H), 2.34 (m, *J* = 7.5, 4.7 Hz, 2H), 1.77 (d, *J* = 9.4 Hz, 3H), 1.70 – 1.62 (m, 2H), 1.51 – 1.40 (m, 2H), 1.31 – 1.25 (m, 2H), 1.23 – 1.18 (m, 2H), 0.92 (m, *J* = 7.5, 7.1, 3.5 Hz, 3H). ^{13}C NMR (101 MHz, $CDCl_3$) δ 173.8, 136.2, 128.7, 128.3, 128.3, 68.8, 66.2, 40.7, 37.6, 36.8, 34.7, 32.6, 29.5, 22.5. The minor isomer: ^1H NMR (400 MHz, $CDCl_3$) δ 7.43 – 7.27 (m, 5H), 5.11 (s, 2H), 3.52 (d, *J* = 6.3 Hz, 2H), 2.34 (m, *J* = 7.5, 4.7 Hz, 2H), 1.77 (d, *J* = 9.4 Hz, 3H), 1.70 – 1.62 (m, 2H), 1.51 – 1.40 (m, 2H), 1.31 – 1.25 (m, 2H), 1.23 – 1.18 (m, 2H), 0.92 (m, *J* = 7.5, 7.1, 3.5 Hz, 3H). ^{13}C NMR (101 MHz, $CDCl_3$) δ 173.8, 136.2, 128.7, 128.3, 128.3, 66.6, 66.5, 38.4, 37.2, 34.9, 34.6, 28.7, 25.4, 22.9. IR (ATR): 3425, 3035, 2921, 2847, 1735, 1452, 1385, 1166, 1034, 748, 698 cm^{-1}. HRMS (EI): m/z $[M + Na]^+$ calcd for $C_{18}H_{26}O_3Na$: 313.1774, found 313.1776.

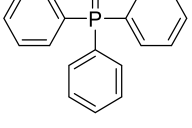

16

triphenylphosphine sulfide (16): The title compound was isolated by column chromatography with petroleum ether and ethyl acetate (PE/EA = 20:1) as a white solid (50.6 mg, 86% yield obtained by **1aa**). ^1H NMR (400 MHz, $CDCl_3$) δ 7.78 – 7.66 (m, 6H), 7.51 (m, *J* = 7.2, 1.8 Hz, 3H), 7.44 (m, *J* = 7.4, 3.0 Hz, 6H). ^{13}C NMR (101 MHz, $CDCl_3$) δ 133.1 (d, *J* = 85.1 Hz), 132.4 (d, *J* = 10.7 Hz), 131.7 (d, *J* = 3.0 Hz),

128.6 (d, J = 12.5 Hz). ^{31}P NMR (162 MHz, $CDCl_3$) δ 43.3. IR (ATR): 3448, 1434, 1104, 754, 715, 692, 638, 517, 510 cm^{-1}. HRMS (EI): m/z $[M + H]^+$ calcd for $C_{18}H_{16}PS$: 295.0705, found 295.0707. This compound is known.[7]

4. Gram-Scale Reaction

4.1 Procedure for Gram-Scale Reaction

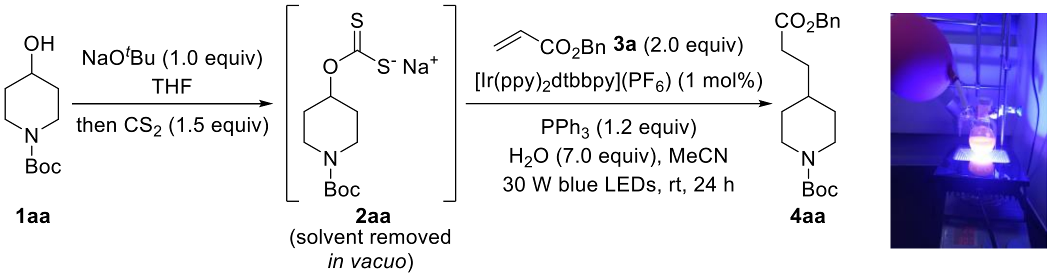

An oven-dried Schlenk tube equipped with a magnetic stir bar was charged with **1aa** (0.805 g, 4.0 mmol), NaO^{t}Bu (0.384 g, 4.0 mmol). The reaction vessel was evacuated and backfilled with nitrogen (threre cycles) and dry THF (25 mL) was added under nitrogen atmosphere. Then the mixture was stirred at room temperature for 30 min. The resulting solution was cooled to 0 °C followed by addition of CS_2 (0.457 g, 0.38 mL, 6.0 mmol) via syringe, then stirred at 0 °C for 3 h before removing the solvent *in vacuo*. Then PPh_3 (1.30 g, 4.8 mmol) and $[Ir(ppy)_2dtbbpy](PF_6)$ (36.5 mg, 1.0 mol%) were rapidly weighed into the Schlenk tube. The Schlenk tube was sealed with a septum cap and wrapped with electrical tape, then evacuated and backfilled with nitrogen (three cycles), and a nitrogen balloon was attached. Then MeCN (40 mL), **3a** (1.30 g, 8.0 mmol) and H_2O (0.504 g, 0.50 mL, 28 mmol) was added via syringe. The resulting reaction mixture was irradiated with a 30 W blue LEDs lamp, and stirred for 24 hours. The solvent was removed *in vacuo* and the residue was purified by column chromatography (petroleum ether/ ethyl acetate = 10:1) to give the desired product **4aa** as a colorless oil (1.01 g, 73% yield).

5. Synthetic Application of the Reaction

5.1 Preparation of compound 1ef

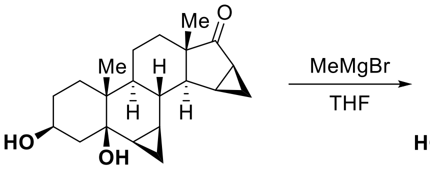

1ee **1ef**

To a solution of **1ee** (0.33 g, 1.0 mmol) in THF (5 mL) was added dropwise a solution of MeMgBr (1.0 M in THF, 2 mL, 2.0 mmol) in THF (2 mL) at 0 ºC. The cooling bath was removed and the mixture was allowed to warm to 26 °C. After 1 h, excess MeMgBr was carefully quenched with saturated aqueous ammonium chloride solution (2 mL). The layers were separated and the aqueous phase was extracted with ether (3 × 10 mL). The combined organic extracts were washed with brine, dried over Na_2SO_4, filtered and concentrated *in vacuo*. The residue was purified by column chromatography (petroleum ether/ ethyl acetate = 1:1) to give the desired product **1ef** as a white solid (0.18 g, 52% yield).[8]

5.2 Synthetic applications of the methodology

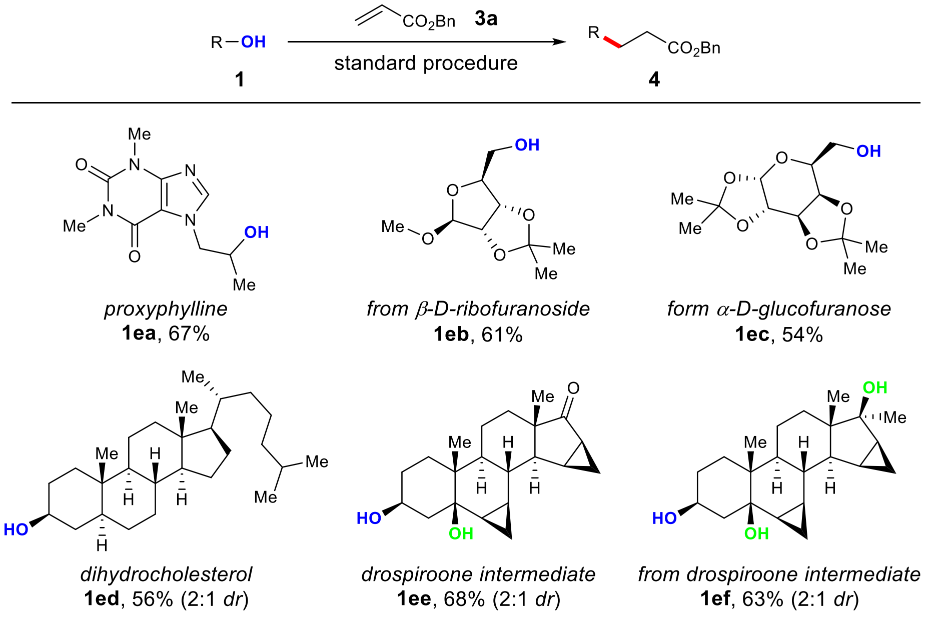

In a nitrogen-filled glovebox, an oven-dried 5 mL quartz tube equipped with a magnetic stir bar was charged sequentially with alcohol **1** (0.20 mmol), NaO^{t}Bu (19.2 mg, 0.20 mmol), followed by addition of dry THF (1.2 mL). The quartz tube was sealed with a septum cap and transferred out of

the glovebox. Then stirred at room temperature for 30 min. The resulting solution was cooled to 0 °C followed by addition of CS_2 (22.8 mg, 19.0 µL, 0.30 mmol) via microsyringe, then stirred at 0 °C for 3 h before removing the solvent *in vacuo*. The system is transferred into the glovebox, and PPh_3 (63.0 mg, 0.24 mmol), [Ir(ppy)$_2$dtbbpy](PF_6) (1.8 mg, 1.0 mol%), alkene **3** (0.40 mmol), H_2O (25.2 mg, 1.4 mmol) in MeCN (2.0 mL) (for **1ea-1ec**) or MeCN (4.0 mL) and DMF (1.0 mL) (for **1ed-1ef**) was added. The quartz tube was sealed with a septum cap and transferred out of the glovebox. The resulting solution was irradiated with a 30 W blue LEDs lamp, maintained at a temperature of 26 °C, and stirred for 24 hours. The solvent was removed *in vacuo* and the residue was purified by column chromatography to afford the product.

5.3 Spectral Data

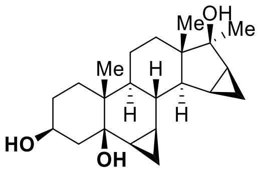

1ef

(2S,4aR,4bS,6aS,7aS,8aS,8bS,8cR,8dR,9aR,9bR)-4a,6a,7-trimethyloctadecahydro-9bH-cyclopropa[4,5]cyclopenta[1,2-a]cyclopropa[l]phenanthrene-2,7,9b-triol (1ef): The title compound was isolated by column chromatography with petroleum ether and ethyl acetate (PE/EA = 1:1) as a white solid (179.9 mg, 52% yield). ^{1}H NMR (400 MHz, $CDCl_3$) δ 4.11 (d, *J* = 7.1 Hz, 1H), 4.06 – 4.00 (m, 1H), 3.36 (d, *J* = 6.9 Hz, 1H), 2.56 (s, 1H), 2.23 (dd, *J* = 15.1, 3.3 Hz, 1H), 2.04 (s, 1H), 2.02 – 1.94 (m, 1H), 1.82 (m, *J* = 14.1, 12.8, 3.7 Hz, 2H), 1.73 (d, *J* = 4.9 Hz, 2H), 1.61 – 1.52 (m, 1H), 1.40 (m, *J* = 22.2, 11.2, 3.2 Hz, 4H), 1.31 (s, 3H), 1.26 – 1.20 (m, 4H), 1.16 – 1.09 (m, 1H), 1.05 (m, *J* = 6.9, 3.6 Hz, 1H), 0.86 (d, *J* = 4.9 Hz, 6H), 0.78 (m, *J* = 5.1 Hz, 1H), 0.63 (m, *J* = 9.0, 4.4 Hz, 1H), 0.34 – 0.23 (m, 1H). ^{13}C NMR (101 MHz, $CDCl_3$) δ 81.1, 75.1, 67.3, 60.5, 53.3, 45.2, 43.3, 42.0, 40.7, 36.7, 34.5, 27.9, 26.9, 25.5, 21.9, 19.3, 18.9, 16.1, 15.5, 14.3, 11.9, 8.2. IR (ATR): 3361, 2946, 1739, 1370, 1243, 1154, 1101, 1061, 740, 913, 864, 828 cm^{-1}. HRMS (EI): m/z [M + Na]$^+$ calcd for $C_{22}H_{34}O_3Na$: 369.2400, found 369.2403.

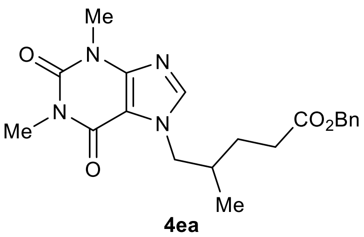

4ea

benzyl 5-(1,3-dimethyl-2,6-dioxo-1,2,3,6-tetrahydro-7H-purin-7-yl)-4-methylpentanoate (4ea): The title compound was isolated by column chromatography with petroleum ether and ethyl acetate (EA/PE = 2:1) as a colorless oil (51.5 mg, 67% yield). ^{1}H NMR (400 MHz, $CDCl_3$) δ 7.49 (s, 1H), 7.36 – 7.30 (m, 5H), 5.09 (s, 2H), 4.20 (dd, *J* = 13.5, 6.6 Hz, 1H), 4.00 (dd, *J* = 13.5, 7.9 Hz, 1H), 3.58 (s, 3H), 3.38 (s, 3H), 2.52 – 2.34 (m, 2H), 2.16 – 2.05

(m, 1H), 1.71 (m, J = 11.6, 5.3, 3.2 Hz, 1H), 1.57 – 1.46 (m, 1H), 0.89 (d, J = 6.7 Hz, 3H). ^{13}C NMR (101 MHz, $CDCl_3$) δ 173.1, 155.3, 151.8, 149.0, 141.4, 136.0, 128.7, 128.4, 128.4, 107.2, 66.5, 52.9, 34.1, 31.6, 29.9, 28.8, 28.1, 16.8. IR (ATR): 3112, 3033, 2956, 2396, 2348, 1734, 1707, 1665, 1603, 1548, 1027, 977, 748, 622, 504 cm^{-1}. HRMS (EI): m/z [M + H]$^+$ calcd for $C_{20}H_{25}N_4O_4$: 385.1870, found 385.1868.

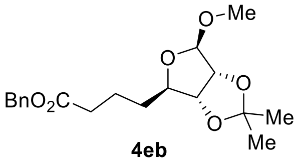

4eb

benzyl 4-((3a*R*,4*R*,6*R*,6a*R*)-6-methoxy-2,2-dimethyltetrahydrofuro[3,4-*d*][1,3]dioxol-4-yl) butanoate (4eb): The title compound was isolated by column chromatography with petroleum ether and ethyl acetate (PE/EA = 10:1) as a colorless oil (42.7 mg, 61% yield). ^{1}H NMR (600 MHz, $CDCl_3$) δ 7.39 – 7.30 (m, 5H), 5.12 (d, J = 3.0 Hz, 2H), 4.93 (s, 1H), 4.58 (d, J = 5.9 Hz, 1H), 4.50 (d, J = 5.9 Hz, 1H), 4.15 – 4.11 (m, 1H), 3.32 (s, 3H), 2.41 (s, 2H), 1.85 – 1.70 (m, 2H), 1.66 – 1.61 (m, 1H), 1.54 (m, J = 13.5, 10.4, 6.5 Hz, 1H), 1.47 (s, 3H), 1.31 (s, 3H). ^{13}C NMR (151 MHz, $CDCl_3$) δ 173.2, 136.1, 128.7, 128.4, 112.4, 109.7, 86.9, 85.6, 84.2, 66.4, 55.1, 34.5, 34.0, 26.6, 25.1, 21.8. IR (ATR): 3034, 2986, 2939, 2834, 1737, 1456, 1379, 1211, 1159, 1105, 1060, 1012, 963, 870, 750, 699 cm^{-1}. HRMS (EI): m/z [M + Na]$^+$ calcd for $C_{19}H_{26}O_6Na$: 373.1622, found 373.1626.

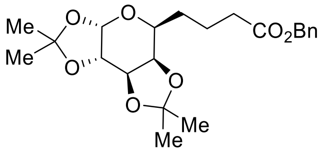

4ec

benzyl 4-((3aS,5S,5aR,8aR,8bS)-2,2,7,7-tetramethyltetrahydro-5H-bis([1,3]dioxolo)[4,5-*b*:4',5'-*d*]pyran-5-yl)butanoate (4ec): The title compound was isolated by column chromatography with petroleum ether and ethyl acetate (PE/EA = 10:1) as a colorless oil (43.8 mg, 54% yield). ^{1}H NMR (400 MHz, $CDCl_3$) δ 7.37 – 7.31 (m, 5H), 5.52 (d, J = 5.1 Hz, 1H), 5.11 (s, 2H), 4.57 (dd, J = 7.9, 2.3 Hz, 1H), 4.29 (dd, J = 5.1, 2.3 Hz, 1H), 4.09 (dd, J = 7.9, 1.9 Hz, 1H), 3.77 – 3.67 (m, 1H), 2.41 (m, J = 6.5 Hz, 2H), 1.88 – 1.72 (m, 2H), 1.71 – 1.65 (m, 1H), 1.58 (m, J = 10.1, 7.1, 4.5 Hz, 1H), 1.51 (s, 3H), 1.45 (s, 3H), 1.33 (d, J = 4.6 Hz, 6H). ^{13}C NMR (151 MHz, $CDCl_3$) δ 173.5, 136.2, 128.7, 128.3, 128.3, 109.2, 108.4, 96.7, 72.9, 71.1, 70.6, 67.1, 66.3, 34.0, 29.5, 26.2, 26.1, 25.1, 24.5, 21.4. IR (ATR): 3034, 2986, 2937, 1736, 1498, 1456, 1380, 1255, 1212, 1107, 1070, 1004, 900, 749, 699 cm^{-1}. HRMS (EI): m/z [M + Na]$^+$ calcd for $C_{22}H_{30}O_7Na$: 429.1884, found 429.1888.

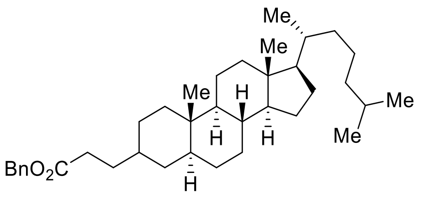

4ed

benzyl 3-((5*S*,8*R*,9*S*,10*S*,13*R*,14*S*,17*R*)-10,13-dimethyl-17-((*R*)-6-methylheptan-2-yl)hexadecahydro-1H-cyclopenta[*a*]phenanthren-3-yl)propanoate (4ed): The title compound was isolated by column chromatography with petroleum ether and ethyl acetate (PE/EA = 400:1) as a colorless oil (59.8 mg, 56% yield, *dr* = 2:1). The major isomer: ^{1}H NMR (400 MHz, $CDCl_3$) δ 7.35 (m, *J* = 5.4, 4.5 Hz, 5H), 5.11 (d, *J* = 2.0 Hz, 2H), 2.39 – 2.31 (m, 2H), 1.95 (d, *J* = 12.4 Hz, 1H), 1.77 (dd, *J* = 15.5, 6.8 Hz, 2H), 1.63 (dd, *J* = 12.8, 3.1 Hz, 3H), 1.58 – 1.42 (m, 5H), 1.40 – 1.29 (m, 6H), 1.29 – 1.15 (m, 7H), 1.12 (dd, *J* = 6.8, 3.3 Hz, 3H), 1.10 – 1.04 (m, 4H), 1.00 (m, *J* = 7.8, 4.4 Hz, 3H), 0.90 (d, *J* = 6.5 Hz, 3H), 0.87 (d, *J* = 1.8 Hz, 3H), 0.85 (d, *J* = 1.9 Hz, 3H), 0.78 (s, 3H), 0.64 (s, 3H). ^{13}C NMR (101 MHz, $CDCl_3$) δ 174.1, 136.3, 128.7, 128.3, 128.3, 66.3, 56.8, 56.4, 54.8, 42.8, 40.4, 40.2, 39.7, 36.6, 36.4, 36.0, 35.7, 33.3, 33.2, 33.0, 32.8, 32.2, 29.1, 28.4, 28.2, 27.3, 25.4, 24.3, 24.0, 23.0, 22.7, 20.9, 18.8, 12.2, 11.9. The minor isomer: ^{1}H NMR (400 MHz, $CDCl_3$) δ 7.35 (m, *J* = 5.4, 4.5 Hz, 5H), 5.11 (d, *J* = 2.0 Hz, 2H), 2.39 – 2.31 (m, 2H), 1.95 (d, *J* = 12.4 Hz, 1H), 1.77 (dd, *J* = 15.5, 6.8 Hz, 2H), 1.63 (dd, *J* = 12.8, 3.1 Hz, 3H), 1.58 – 1.42 (m, 5H), 1.40 – 1.29 (m, 6H), 1.29 – 1.15 (m, 7H), 1.12 (dd, *J* = 6.8, 3.3 Hz, 3H), 1.10 – 1.04 (m, 4H), 1.00 (m, *J* = 7.8, 4.4 Hz, 3H), 0.90 (d, *J* = 6.5 Hz, 3H), 0.87 (d, *J* = 1.8 Hz, 3H), 0.85 (d, *J* = 1.9 Hz, 3H), 0.73 (s, 3H), 0.64 (s, 3H). ^{13}C NMR (101 MHz, $CDCl_3$) δ 174.1, 136.3, 128.7, 128.3, 128.3, 66.2, 56.7, 56.5, 54.8, 46.7, 42.8, 39.7, 38.6, 37.7, 36.3, 36.2, 35.7, 35.4, 35.1, 32.4, 32.3, 32.1, 31.6, 30.3, 29.8, 29.1, 28.7, 28.2, 24.4, 23.0, 22.7, 21.2, 18.8, 12.2, 12.4. IR (ATR): 3446, 2934, 2857, 1736, 1456, 1383, 1219, 1184, 1151, 959, 753, 698 cm^{-1}. HRMS (EI): m/z $[M + Na]^+$ calcd for $C_{37}H_{58}O_2Na$: 557.4329, found 557.4326. This compound is known.[2]

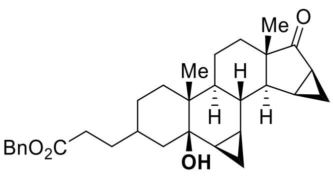

4ee

benzyl 3-((4a*R*,4b*S*,6a*S*,7a*S*,8a*S*,8b*S*,8c*R*,8d*R*,9a*R*,9b*R*)-9b-hydroxy-4a,6a-dimethyl-7-oxooctadecahydro-1H-cyclopropa[4,5]cyclopenta[1,2-a]cyclopropa[*l*]phenanthren-2-yl)propanoate (4ee): The title compound was isolated by column chromatography with petroleum ether and ethyl acetate (PE/EA = 4:1) as a colorless oil (64.7 mg, 68% yield, *dr* = 2:1). The major isomer: ^{1}H NMR (400 MHz, $CDCl_3$) δ 7.34 (m, *J* = 5.4, 4.6 Hz, 5H), 5.10 (d, *J* = 2.2 Hz, 2H), 2.46 – 2.33 (m, 2H), 2.27 – 1.97 (m, 2H), 1.86 – 1.05 (m, 20H), 0.94 (s, 3H), 0.84 – 0.80 (m, 1H), 0.79 (s, 3H), 0.75 (d, *J* = 5.1 Hz, 1H), 0.71 – 0.49 (m, 1H). ^{13}C NMR (101 MHz, $CDCl_3$) δ 216.3, 173.9, 136.2, 128.7, 128.4, 128.3, 73.2, 66.3, 52.1, 45.8, 45.7,

43.1, 40.3, 35.1, 33.6, 33.1, 32.0, 31.9, 26.7, 26.1, 26.0, 25.9, 22.5, 20.9, 20.3, 19.0, 17.7, 14.7, 11.8. The minor isomer: ^{1}H NMR (400 MHz, $CDCl_3$) δ 7.34 (m, *J* = 5.4, 4.6 Hz, 5H), 5.10 (d, *J* = 2.2 Hz, 2H), 2.46 – 2.33 (m, 2H), 2.27 – 1.97 (m, 2H), 1.86 – 1.05 (m, 20H), 0.91 (s, 3H), 0.85 (s, 3H), 0.84 – 0.80 (m, 1H), 0.75 (d, *J* = 5.1 Hz, 1H), 0.71 – 0.49 (m, 1H). ^{13}C NMR (101 MHz, $CDCl_3$) δ 216.9, 173.9, 136.2, 128.7, 128.4, 128.3, 73.2, 66.3, 53.3, 45.8, 45.7, 42.5, 40.2, 35.8, 34.4, 33.7, 33.1, 32.3, 32.0, 26.1, 26.0, 22.9, 22.5, 20.3, 19.0, 17.7, 14.7, 14.6, 11.8. IR (ATR): 3520, 2928, 2866, 1722, 1455, 1375, 1243, 1168, 1048, 918, 735, 698 cm^{-1}. HRMS (EI): m/z $[M + Na]^+$ calcd for $C_{31}H_{40}O_4Na$: 499.2819, found 499.2814.

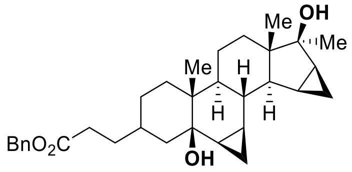

4ef

benzyl 3-((4a*R*,4b*S*,6a*S*,7a*S*,8a*S*,8b*S*,8c*R*,8d*R*,9a*R*,9b*R*)-7,9b-dihydroxy-4a,6a,7-trimethyloctadecahydro-1H-cyclopropa[4,5]cyclopenta[1,2-*a*]cyclopropa[l]phenanthren-2-yl)propanoate (4ef): The title compound was isolated by column chromatography with petroleum ether and ethyl acetate (PE/EA = 3:1) as a colorless oil (62.0 mg, 63% yield, *dr* = 2:1). The major isomer: ^{1}H NMR (400 MHz, $CDCl_3$) δ 7.38 – 7.30 (m, 5H), 5.11 (d, *J* = 1.8 Hz, 2H), 2.44 – 2.35 (m, 2H), 1.77 – 1.68 (m, 3H), 1.59 (m, *J* = 17.2, 13.9, 8.0, 5.3 Hz, 5H), 1.40 (m, J = 8.0, 4.2 Hz, 4H), 1.32 (d, *J* = 8.8 Hz, 6H), 1.25 – 1.04 (m, 5H), 0.86 (s, 3H), 0.83 (s, 3H), 0.79 (s, 3H), 0.72 – 0.47 (m, 2H), 0.32 – 0.21 (m, 1H). ^{13}C NMR (101 MHz, $CDCl_3$) δ 174.0, 136.2, 128.7, 128.4, 128.3, 81.1, 77.4, 66.3, 53.2, 45.8, 45.3, 41.9, 40.3, 36.7, 34.5, 33.8, 33.2, 32.1, 31.9, 26.9, 26.8, 26.1, 25.5, 21.5, 19.0, 18.9, 16.1, 15.8, 11.8, 8.2. The minor isomer: ^{1}H NMR (400 MHz, $CDCl_3$) δ 7.38 – 7.30 (m, 5H), 5.11 (d, *J* = 1.8 Hz, 2H), 2.44 – 2.35 (m, 2H), 1.77 – 1.68 (m, 3H), 1.59 (m, *J* = 17.2, 13.9, 8.0, 5.3 Hz, 5H), 1.40 (m, *J* = 8.0, 4.2 Hz, 4H), 1.32 (d, *J* = 8.8 Hz, 6H), 1.25 – 1.04 (m, 5H), 0.88 (s, 3H), 0.84 (s, 3H), 0.79 (s, 3H), 0.72 – 0.47 (m, 2H), 0.32 – 0.21 (m, 1H). ^{13}C NMR (101 MHz, $CDCl_3$) δ 174.0, 136.2, 128.7, 128.4, 128.3, 81.2, 73.4, 66.3, 54.26, 53.2, 45.3, 41.3, 40.2, 37.1, 35.2, 33.8, 33.2, 32.4, 32.1, 26.9, 26.7, 26.1, 25.4, 19.0, 18.9, 17.5, 16.4, 15.8, 11.8, 8.0. IR (ATR): 3469, 3010, 2925, 2861, 1732, 1671, 1454, 1373, 1242, 1170, 1050, 941, 751, 698 cm^{-1}. HRMS (EI): m/z $[M + Na]^+$ calcd for $C_{32}H_{44}O_4Na$: 515.3132, found 515.3135.

6. Mechanism Studies

6.1 Luminescence Quenching Experiments

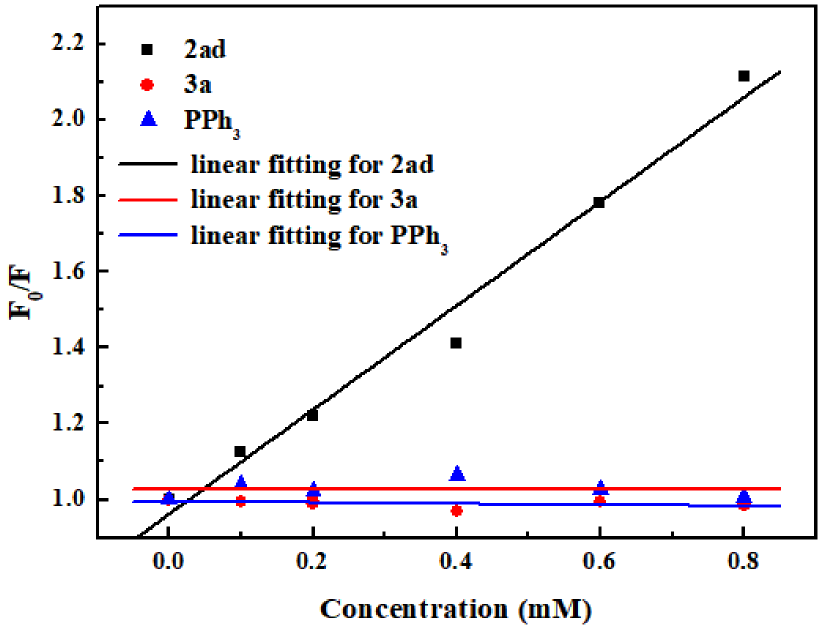

Supplementary Figure 2. [Ir(ppy)$_2$dtbbpy](PF$_6$) emission quenching by **2ad**, **3a** and PPh$_3$.

Fluorescence spectra was collected on Shimadzu Fluorescence Spectrophotometer RF-5301PC for all experiments. All [Ir(ppy)$_2$dtbbpy](PF$_6$) solutions were excited at 416 nm and the emission intensity was collected at 558 nm. In a typical experiment, the emission spectrum of a 2×10^{-5} M solution of [Ir(ppy)$_2$dtbbpy](PF$_6$) in MeCN was collected. The significant decrease of Ir(ppy)$_2$dtbbpy luminescence could be observed in the presence of substrate **2ad**.

6.2 Light On-Off Experiments

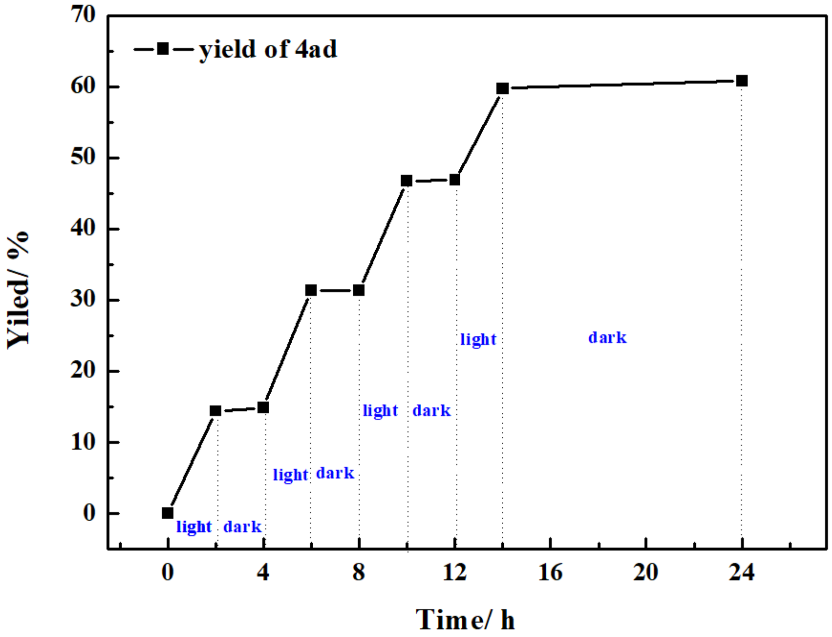

Supplementary Figure 3. Light on-off experiments

The yield of **4ad** was determined by GC using 1,3,5-trimethoxybenzene as an internal standard. The results shows that a radical chain process is not the major reaction pathway, while it could not be completely ruled out at the current stage.

6.3 Determination of Quantum Yields

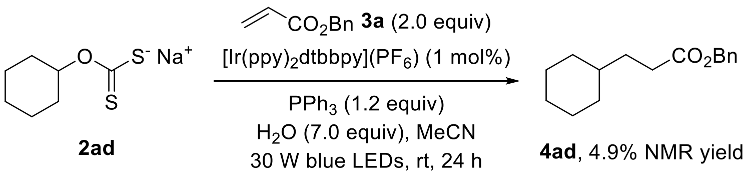

In a nitrogen-filled glovebox, an oven-dried 5 mL quartz tube equipped with a magnetic stirrer bar was charged sequentially with **2ad** (39.7 mg, 0.2 mmol), PPh_3 (63.0 mg, 0.24 mmol), $[Ir(ppy)_2dtbbpy](PF_6)$ (1.8 mg, 1.0 mol%), **3a** (64.9 mg, 0.4 mmol), H_2O (25.2 mg, 1.4 mmol) in MeCN (2.0 mL). Then, the quartz tube was sealed with a septum cap and transferred out of the glovebox. The reaction mixture was irradiated (λ = 455 nm, slit width = 3.0 mm, slit height 5.0 mm with intensity of 0.80 mW•cm^{-2}) for 37554 s. After irradiation, the yield of product formed was

determined by ^{1}H NMR based on a 1,3,5-trimethoxybenzene standard. The quantum yield was determined as follows.

$$\Phi = \frac{n_{\mathbf{4ad}}\, N_A/t}{f\, P\, \lambda/hc}$$

ϕ = Mole number for product/Mole number for absorption of photons = 0.124

n_{4ad}: the mole number of the product **4ad**; t: reaction time (37554 s); N_A: 6.02×10^{23}/mol; f: $1\text{-}10^{-A}$ (455 nm, A=1.5853); P: P=E*S (E: illumination intensity, E=1.88 mW/cm^2; S: the area that irradiated S = 0.15 cm^2); λ: wavelength ($\lambda = 4.55\times10^{-7}$ m); h: planck constant ($h = 6.626\times10^{-34}$ J*s); c: velocity of light ($c = 3\times10^{8}$ m/s).

This result shows that the radical chain process is not main pathway.

6.4 TEMPO Trapping Experiment

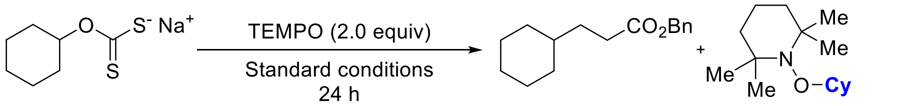

2ad **4ad**, 0% **10**, detected by HRMS

In a nitrogen-filled glovebox, an oven-dried 5 mL quartz tube equipped with a magnetic stir bar was charged sequentially with **2ad** (39.7 mg, 0.20 mmol), PPh_3 (63.0 mg, 0.24 mmol), $[Ir(ppy)_2dtbbpy](PF_6)$ (1.8 mg, 1.0 mol%), **3a** (64.9 mg, 0.40 mmol), H_2O (25.2 mg, 1.4 mmol) and TEMPO (62.5 mg, 0.40 mmol) in MeCN (2.0 mL). The quartz tube was sealed with a septum cap and transferred out of the glovebox. The reaction mixture was irradiated with a 30 W blue LED lamp, maintained at a temperature of 26 °C, and stirred for 24 hours. This result showed that the standard reaction was completely inhibited, and the TEMPO trapped cyclohexyl radical could be identified by HRMS (ESI, m/z). Calcd for $C_{15}H_{29}NO$ (M+H$^+$): 240.2322, found: 240.2322.

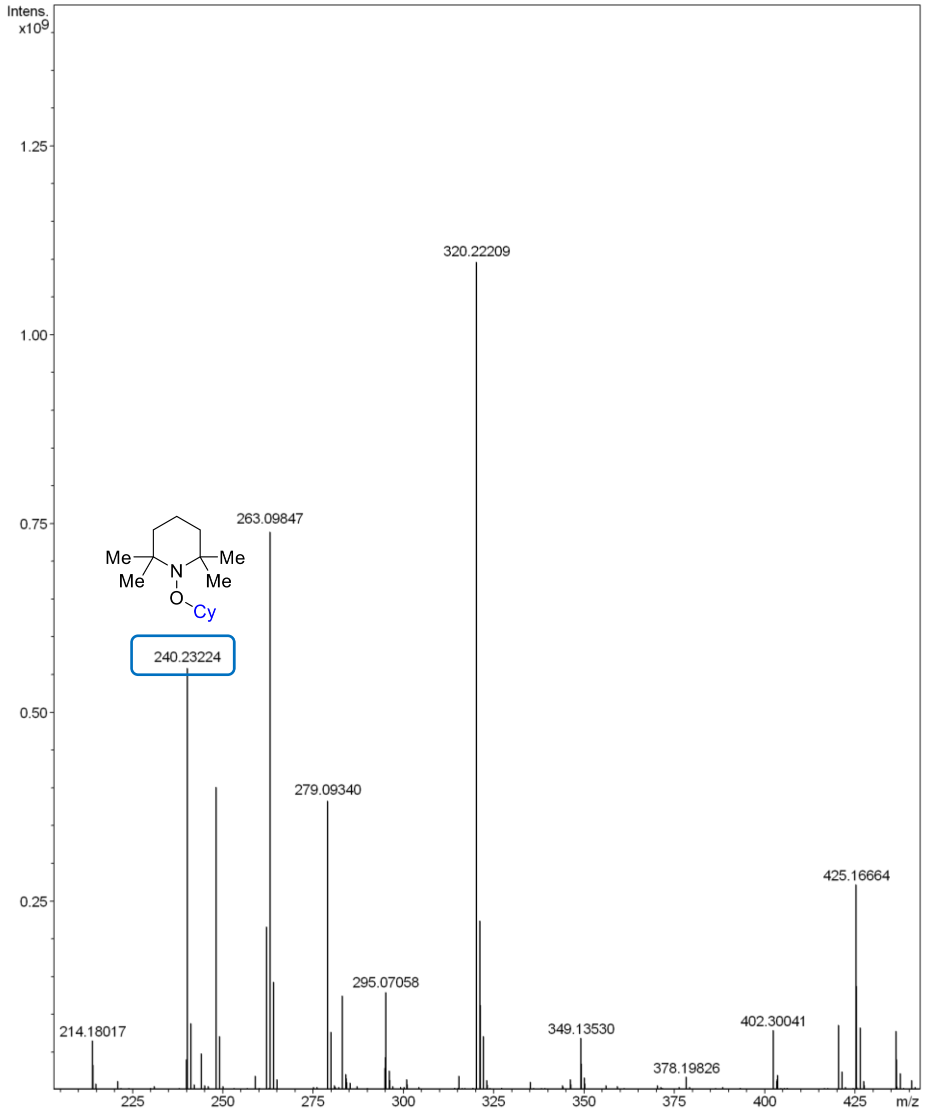

Supplementary Figure 4. TEMPO Trapping Experiment

6.5 Radical Clock Experiment

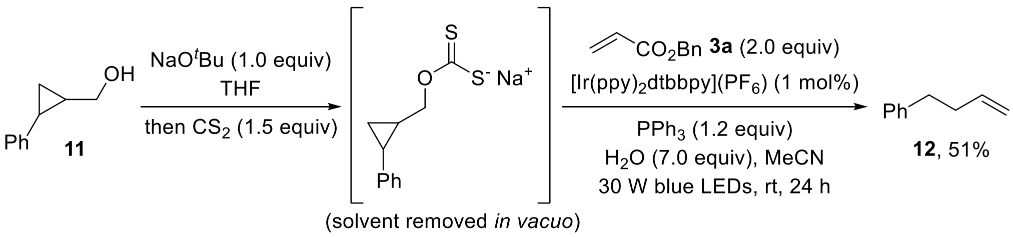

In a nitrogen-filled glovebox, an oven-dried 5 mL quartz tube equipped with a magnetic stir bar was charged sequentially with alcohol **11** (29.6 mg, 0.20 mmol), NaO^{t}Bu (19.2 mg, 0.20 mmol), followed by addition of dry THF (1.2 mL). The quartz tube was sealed with a septum cap and

transferred out of the glovebox. Then stirred at room temperature for 30 min. The resulting solution was cooled to 0 °C followed by addition of CS_2 (22.8 mg, 19.0 µL, 0.30 mmol) via microsyringe and stirred at 0 °C for 3 h before removing the solvent *in vacuo*. The system is transferred into the glovebox, and PPh_3 (63.0 mg, 0.24 mmol), [Ir(ppy)$_2$dtbbpy](PF_6) (1.8 mg, 1.0 mol%), **3a** (64.9 mg, 0.40 mmol), H_2O (25.2 mg, 1.4 mmol) in MeCN (2.0 mL) was added. The quartz tube was sealed with a septum cap and transferred out of the glovebox. The reaction mixture was irradiated with a 30 W blue LEDs lamp, maintained at a temperature of 26 °C, and stirred for 24 hours. The solvent was removed *in vacuo* and the residue was purified by column chromatography with petroleum ether to afford **12** (13.5 mg, 51% yield). ^{1}H NMR (400 MHz, $CDCl_3$) δ 7.27 (m, *J* = 7.7 Hz, 2H), 7.18 (d, *J* = 7.3 Hz, 3H), 5.94 – 5.77 (m, 1H), 5.09 – 4.93 (m, 2H), 2.75 – 2.66 (m, 2H), 2.37 (m, *J* = 7.9, 7.2 Hz, 2H). ^{13}C NMR (101 MHz, $CDCl_3$) δ 142.0, 138.2, 128.6, 128.4, 125.9, 115.0, 35.7, 35.5. IR (ATR): 3027, 2926, 1641, 1496, 1454, 912, 746, 698 cm^{-1}. HRMS (EI): m/z [M + Na]$^+$ calcd for $C_{10}H_{12}Na$: 155.0831, found 155.0828.

6.6 Electrochemical Studies

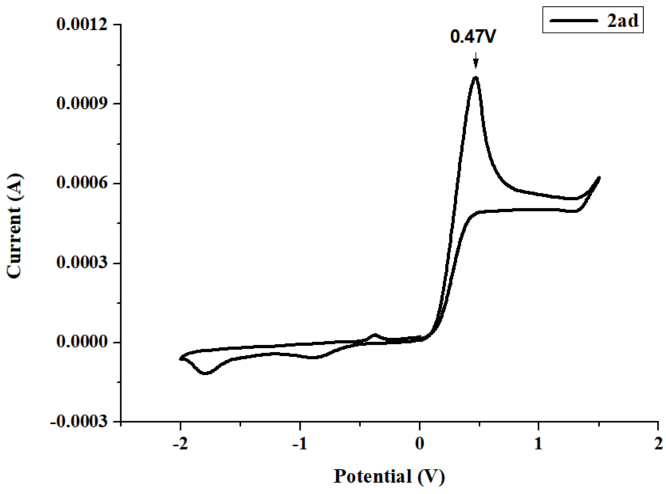

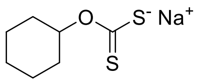

2ad

E = +0.47 V

Supplementary Figure 5. Cyclic voltammetry of **2ad**

Cyclic voltammetry (CV) was taken using a CHI830C potentiostation. CV measurement was performed in a three-electrode cell (volume 30 mL; MeCN as solvent, ^{n}Bu$_4$PF$_6$ (0.1 M) as the supporting electrolyte, 0.01 M concentration of **2ad**) with glassy carbon as working electrode, Pt

wire as the auxiliary electrode, and saturated calomel electrode as the reference electrode. Samples were examined at a scan rate of 0.1 V/s with the protection of N_2. As a result, $E_{\mathbf{2ad}}$ = +0.47V versus SCE in MeCN. These results showed that **2ad** was suitable for SET in the excited state of the $*Ir^{III}$ ($E_{1/2}$$\{[Ir(ppy)_2dtbbpy](PF_6)\}^{III}*/\{[Ir(ppy)_2dtbbpy](PF_6)\}^{II}$ = +0.66 versus SCE in MeCN).[9]

6.7 EPR Experiment

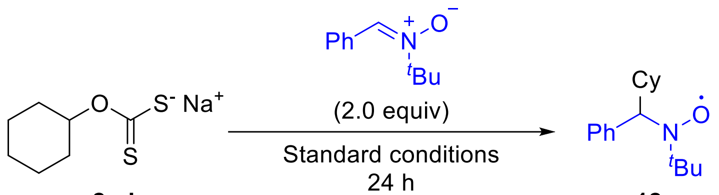

The electron paramagnetic resonance (EPR) spectroscopy was recorded on a Bruker EMXmicro-6/1. With the addition of tert-butyl-α-phenylnitrone (PBN) as a free radical spin trap, we detected signals that are clearly identified as EPR signals of the CyPBN adduct according to the literature data.[10] EPR spectra obtained in MeCN at 298 K in the presence of PBN. Line I: A solution of PBN (70.9 mg, 0.4 mmol) in MeCN (2.0 mL). Line II: A solution of **2ad** (39.7 mg, 0.2 mmol), PPh_3 (63.0 mg, 0.24 mmol), $[Ir(ppy)_2dtbbpy](PF_6)$ (1.8 mg, 1.0 mol%), **3a** (64.9 mg, 0.4 mmol), H_2O (25.2 mg, 1.4 mmol), PBN (70.9 mg, 0.4 mmol) in MeCN (2.0 mL).

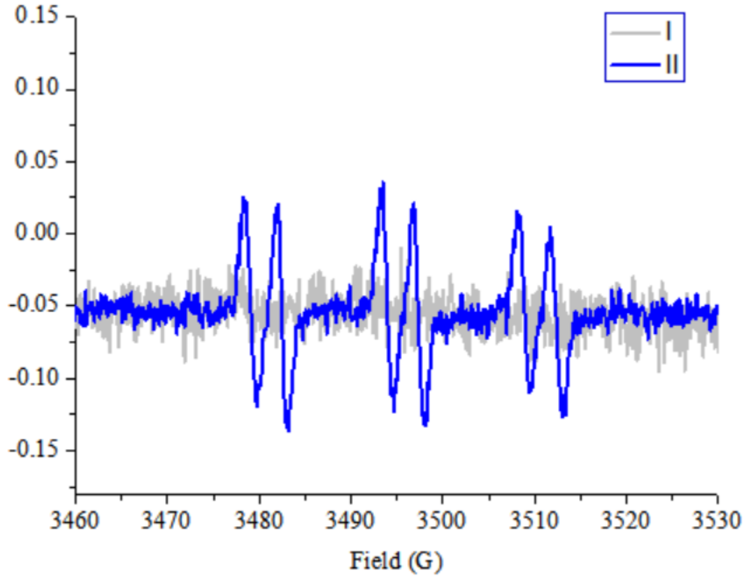

Supplementary Figure 6. EPR spectra

The X-band EPR spectrum of trapped cyclohexyl radical (Cy = cyclohexyl) and corresponding simulated spectrum based on hyperfine coupling constants of A_N = 14.9123 G, A_H = 3.41209 G (g-factor = 2.00619).

Experiment parameters:

Center-Field: 3508.95 G

Width: 200.0 G

Modulation Frequency: 9.815572 GHz

Microwave Power: 2.0 mW

Time constant: 1.28 ms

6.8 Deuterium-Labeling Experiment

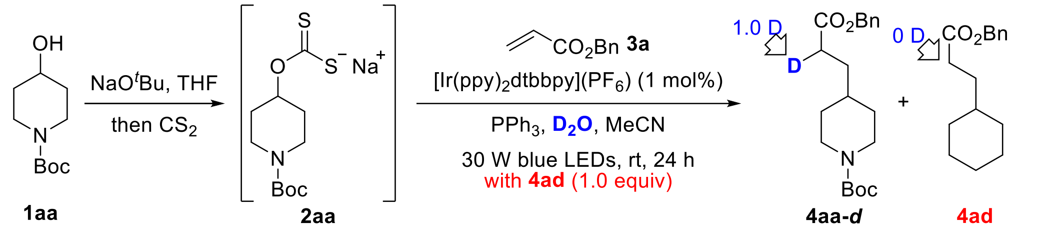

1aa **2aa** (solvent removed *in vacuo*) **4aa-*d*** 84% yield **4ad** 97% recovered

In a nitrogen-filled glovebox, an oven-dried 5 mL quartz tube equipped with a magnetic stir bar was charged sequentially with **1aa** (40.2 mg, 0.20 mmol), NaO^{t}Bu (19.2 mg, 0.20 mmol), followed by addition of dry THF (1.2 mL). The quartz tube was sealed with a septum cap and transferred out of the glovebox. Then stirred at room temperature for 30 min. The resulting solution was cooled to 0 °C followed by addition of CS_2 (22.8 mg, 19.0 µL, 0.30 mmol) via microsyringe, then stirred at 0 °C for 3 h before removing the solvent *in vacuo*. The system is transferred into the glovebox, and PPh_3 (63.0 mg, 0.24 mmol), [Ir(ppy)$_2$dtbbpy](PF$_6$) (1.8 mg, 1.0 mol%), **3a** (64.8 mg, 0.40 mmol), **4ad** (49.2 mg, 0.20 mmol), D_2O (28.0 mg, 1.4 mmol) in MeCN (2.0 mL) was added. The quartz tube was sealed with a septum cap and transferred out of the glovebox. The reaction mixture was irradiated with a 30 W blue LEDs lamp, maintained at a temperature of 26 °C, and stirred for 24 hours. The solvent was removed *in vacuo* and the residue was purified by column chromatography to afford **4aa-*d*** (58.5 mg, 84% yield). Meanwhile, no deuterium incorporation was observed in recovered **4ad**, indicating no H/D exchange between ester and deuterium oxide via keto–enol tautomerism occurred under the reaction conditions. These results support the intermediacy of *a*-acyl carbon anion **V** in the catalytic cycle.

6.9 Carbonyl Sulfide (COS) Detecting Experiment

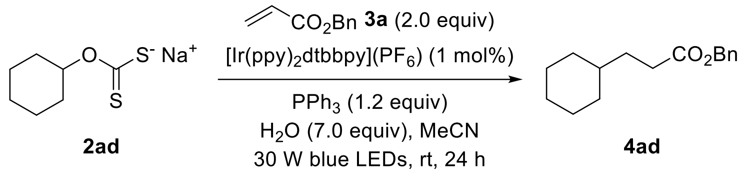

In a nitrogen-filled glovebox, an oven-dried 5 mL quartz tube equipped with a magnetic stirrer bar was charged sequentially with **2ad** (39.7 mg, 0.20 mmol), PPh_3 (63.0 mg, 0.24 mmol), $[Ir(ppy)_2dtbbpy](PF_6)$ (1.8 mg, 1.0 mol%), **3a** (64.9 mg, 0.40 mmol), and H_2O (25.2 mg, 1.4 mmol) in MeCN (2.0 mL). Then, the quartz tube was sealed with a septum cap and transferred out of the glovebox. The reaction mixture was irradiated with a 30 W blue LEDs lamp, maintained at a temperature of 26 ℃, and stirred for 24 hours. Then, a 10 μL sample of the reaction's headspace was taken with a GASTIGHT-Hamilton and injected into a GC-MS (Agilent gas chromatograph-mass spectrometer 7890A/5975C). The COS was detected compared to standard gas.

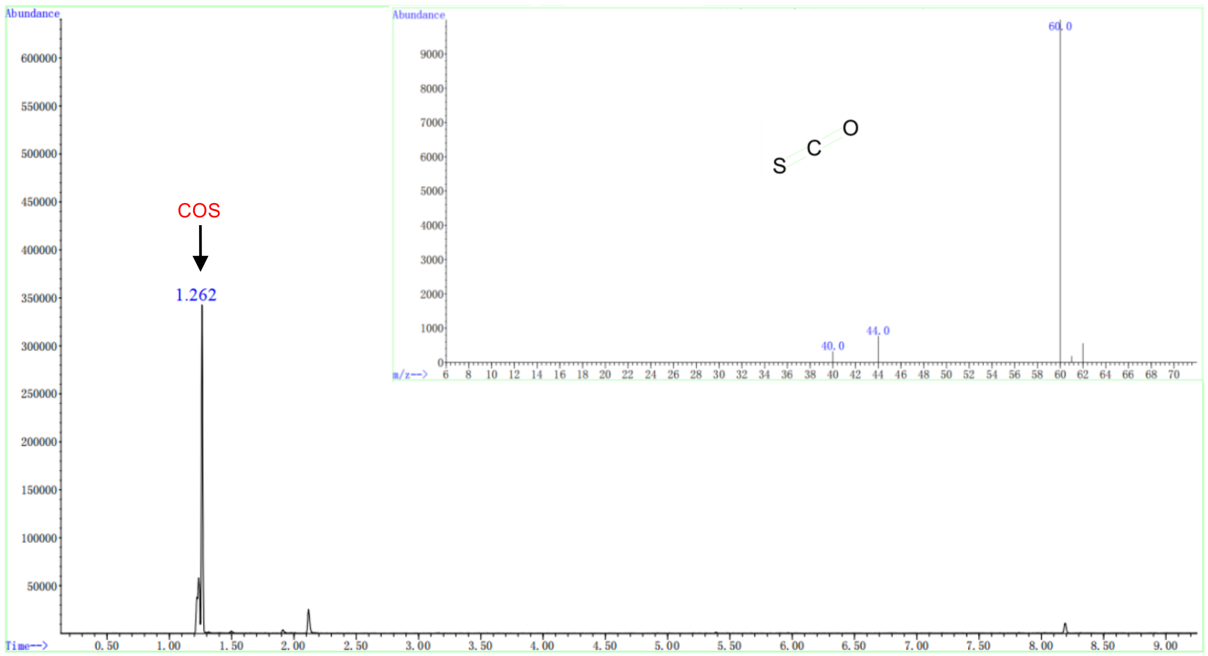

Supplementary Figure 7. GC-MS of standard gas

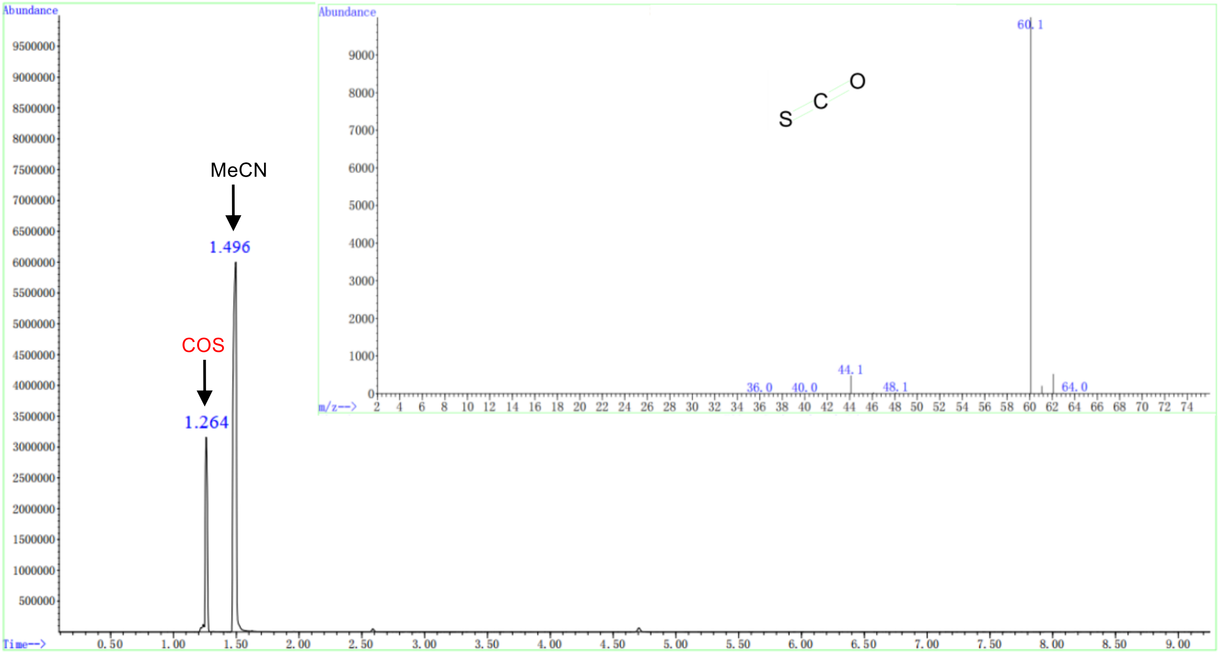

Supplementary Figure 8. GC-MS of gas in the headspace of the reaction

7. NMR Spectra

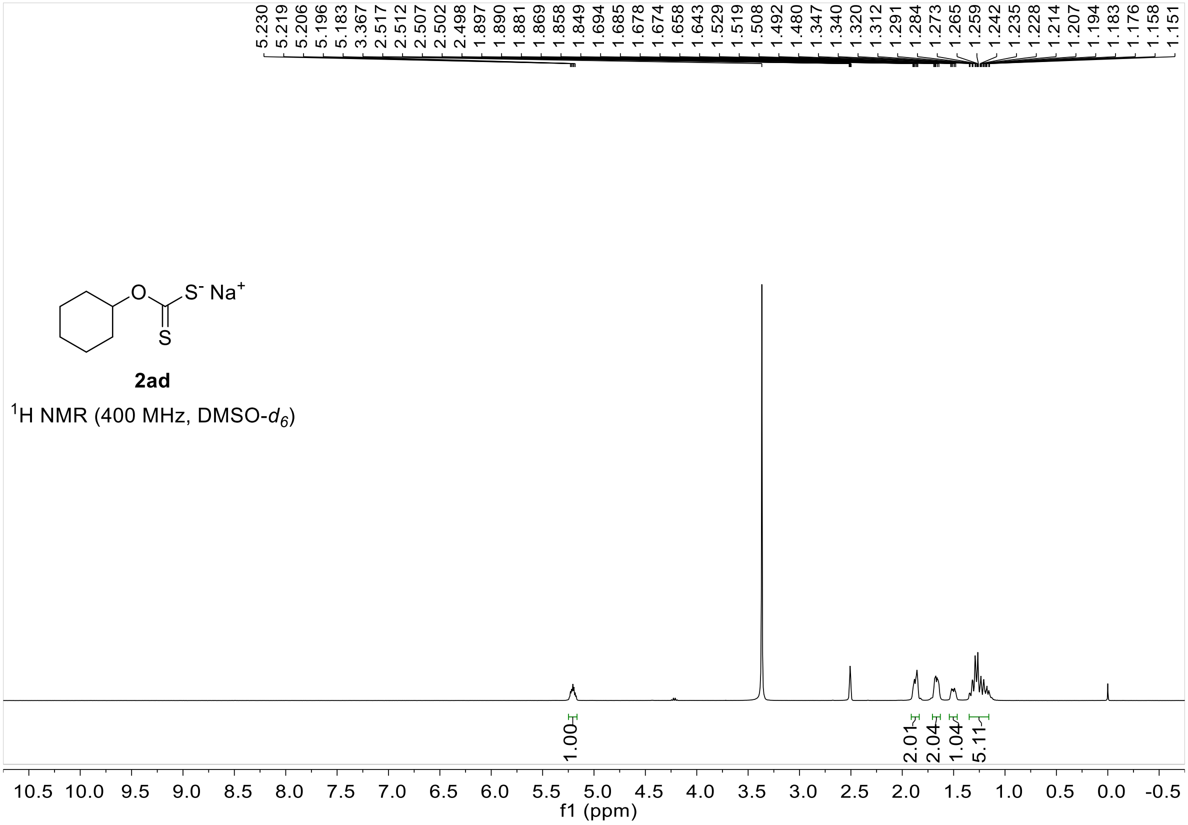

Supplementary Figure 9. ^{1}H NMR spectra of compound **2ad** (400 MHz, DMSO-d_6)

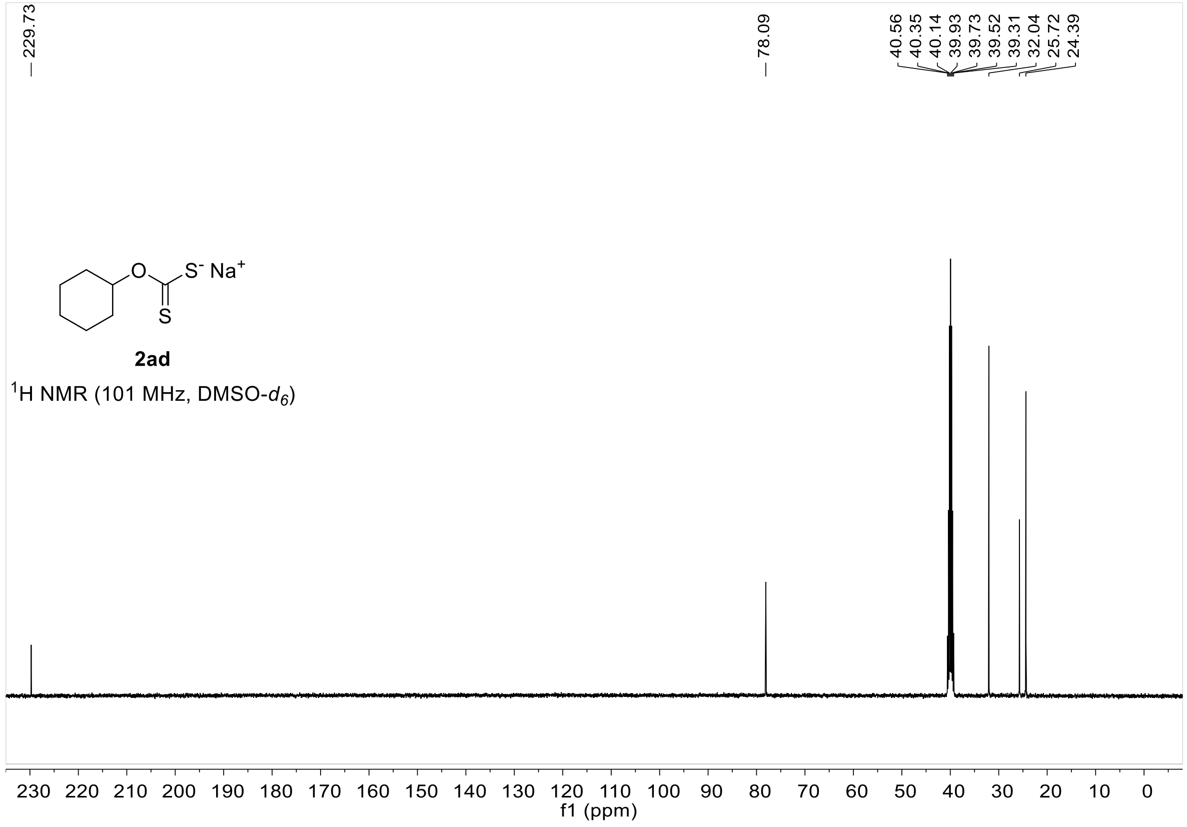

Supplementary Figure 10. ^{13}C NMR spectra of compound **2ad** (101 MHz, DMSO-d_6)

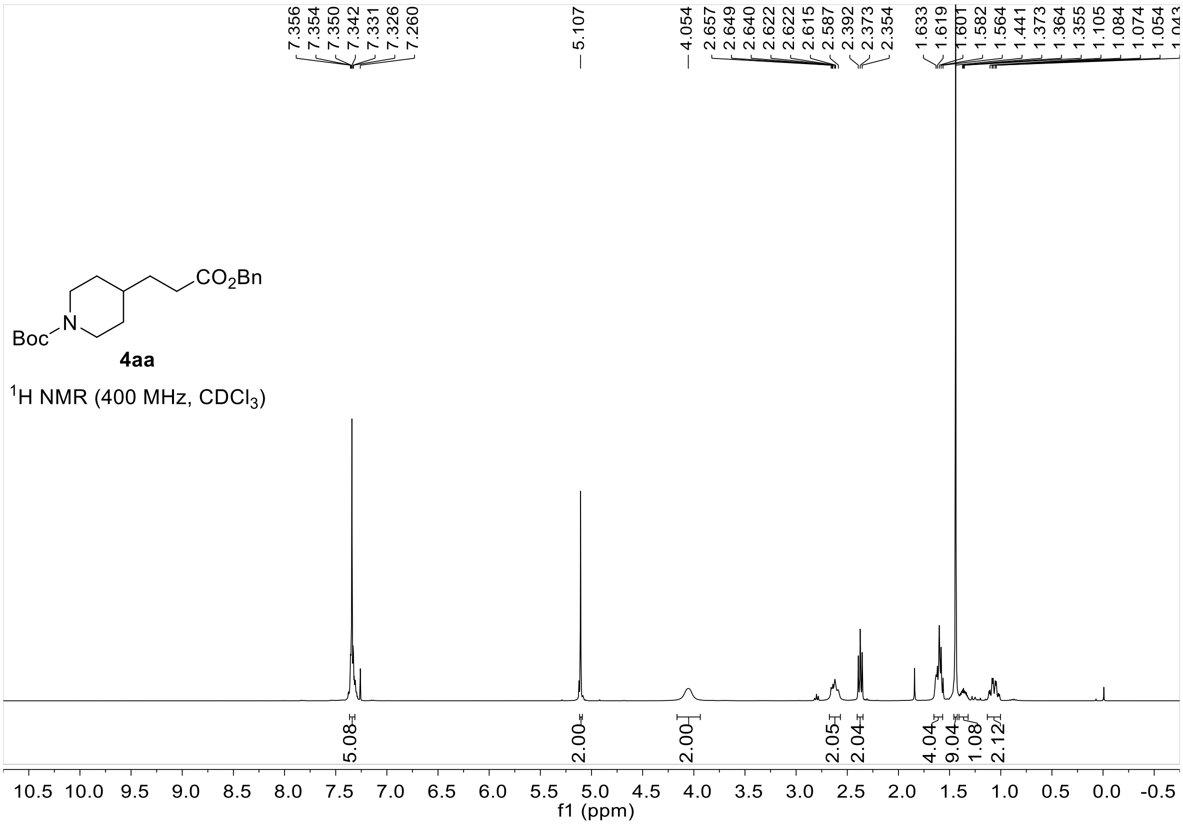

Supplementary Figure 11. ^{1}H NMR spectra of compound **4aa** (400 MHz, $CDCl_3$)

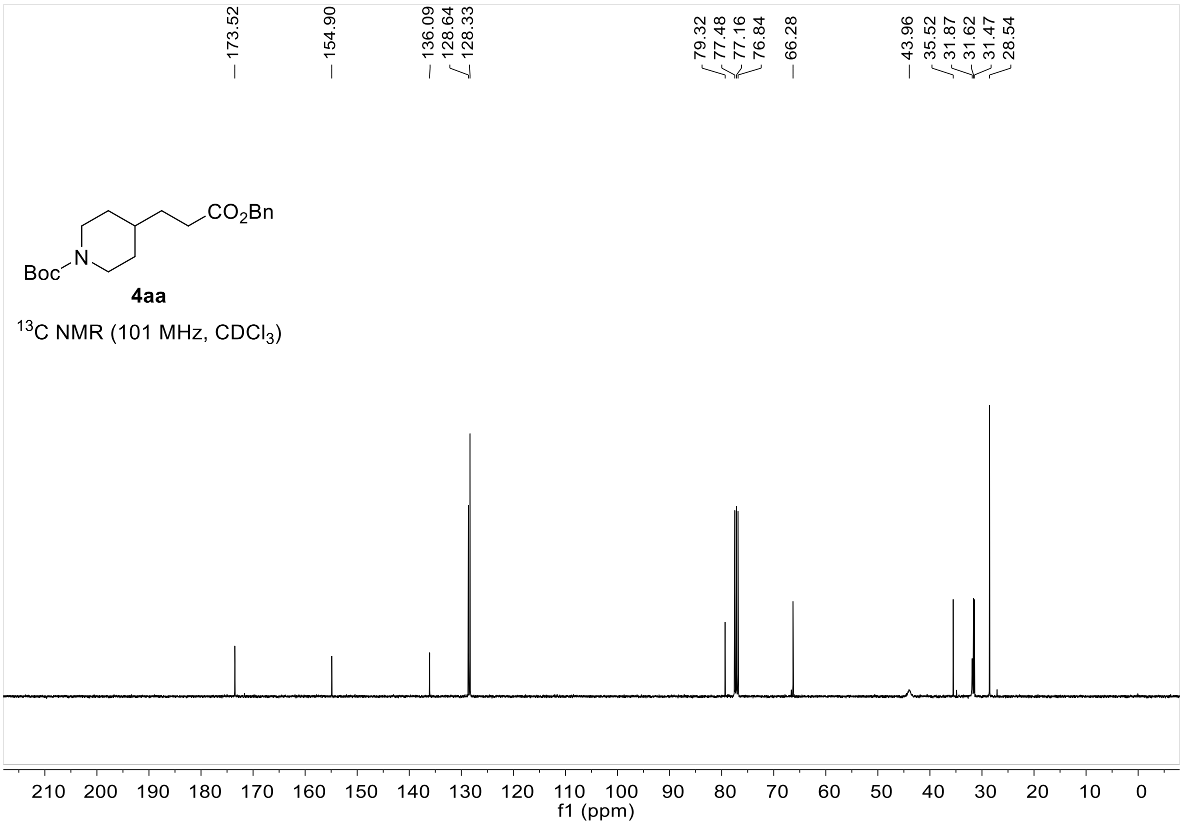

Supplementary Figure 12. ^{13}C NMR spectra of compound **4aa** (101 MHz, $CDCl_3$)

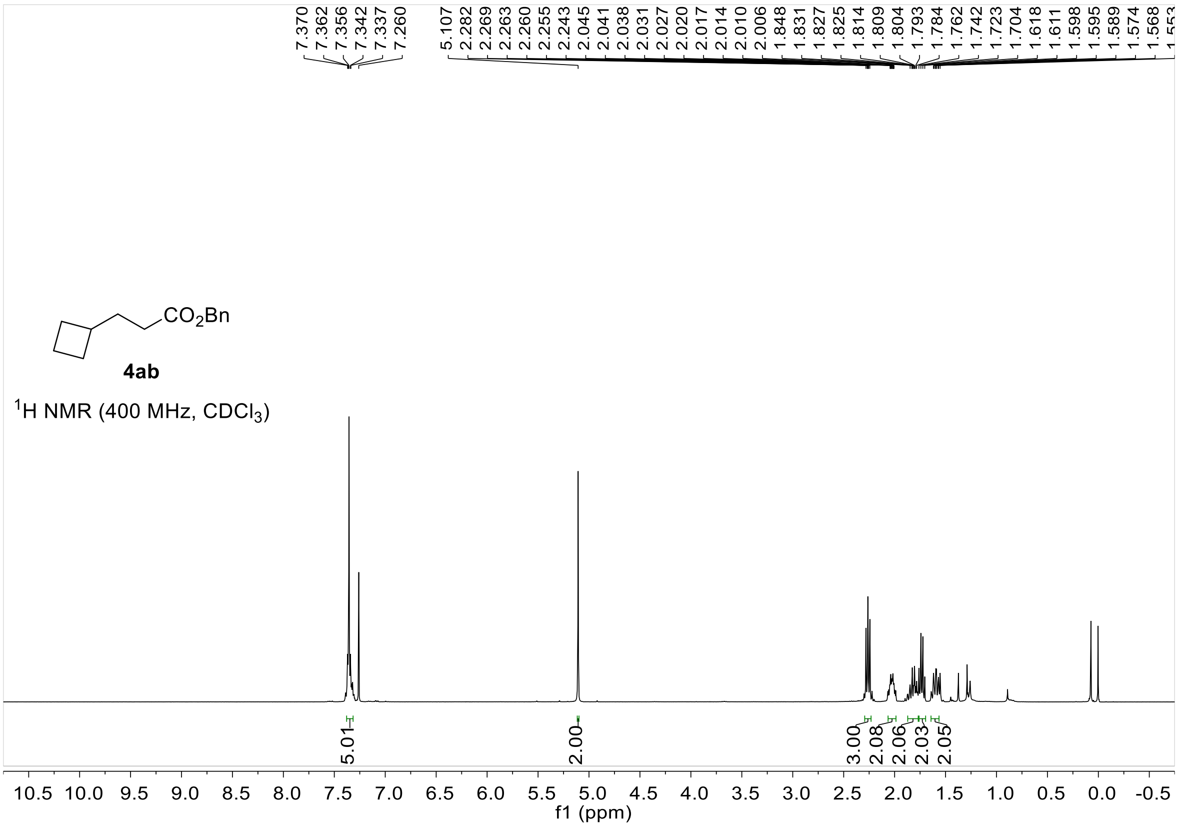

Supplementary Figure 13. ^{1}H NMR spectra of compound **4ab** (400 MHz, $CDCl_3$)

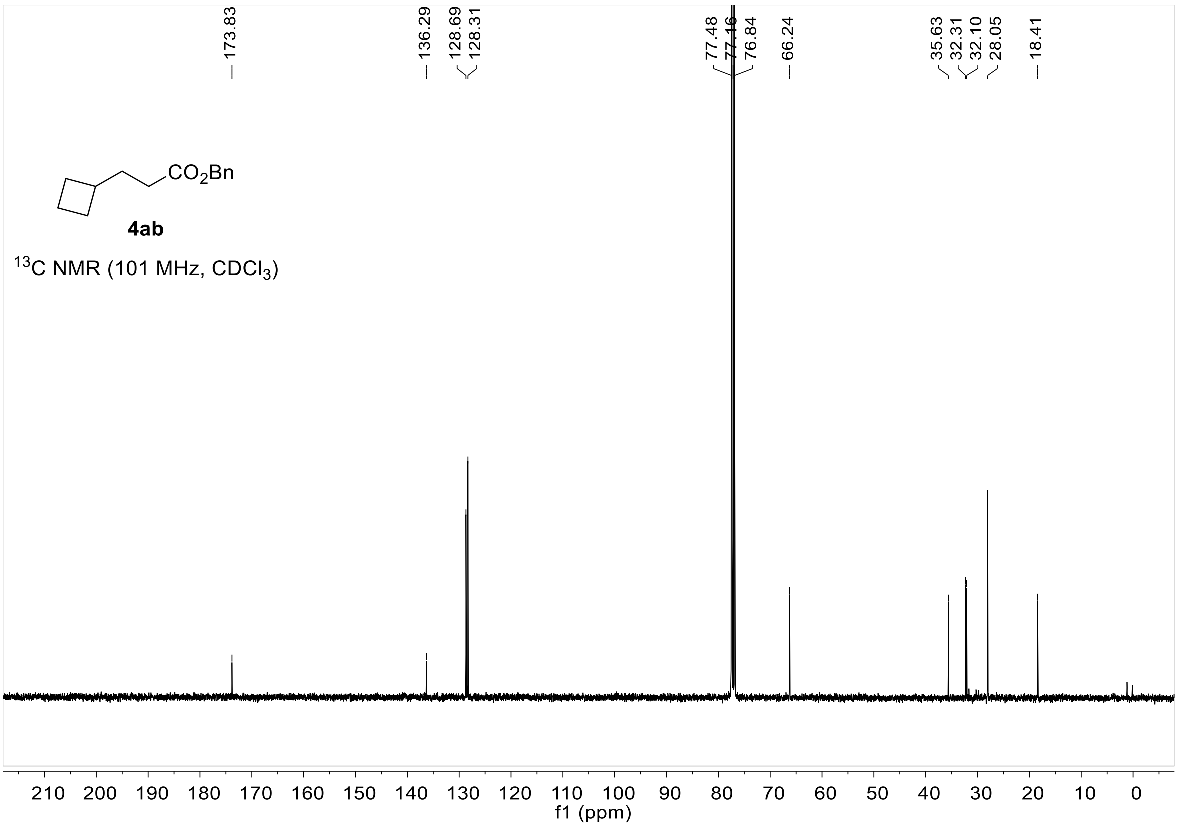

Supplementary Figure 14. ^{13}C NMR spectra of compound **4ab** (101 MHz, $CDCl_3$)

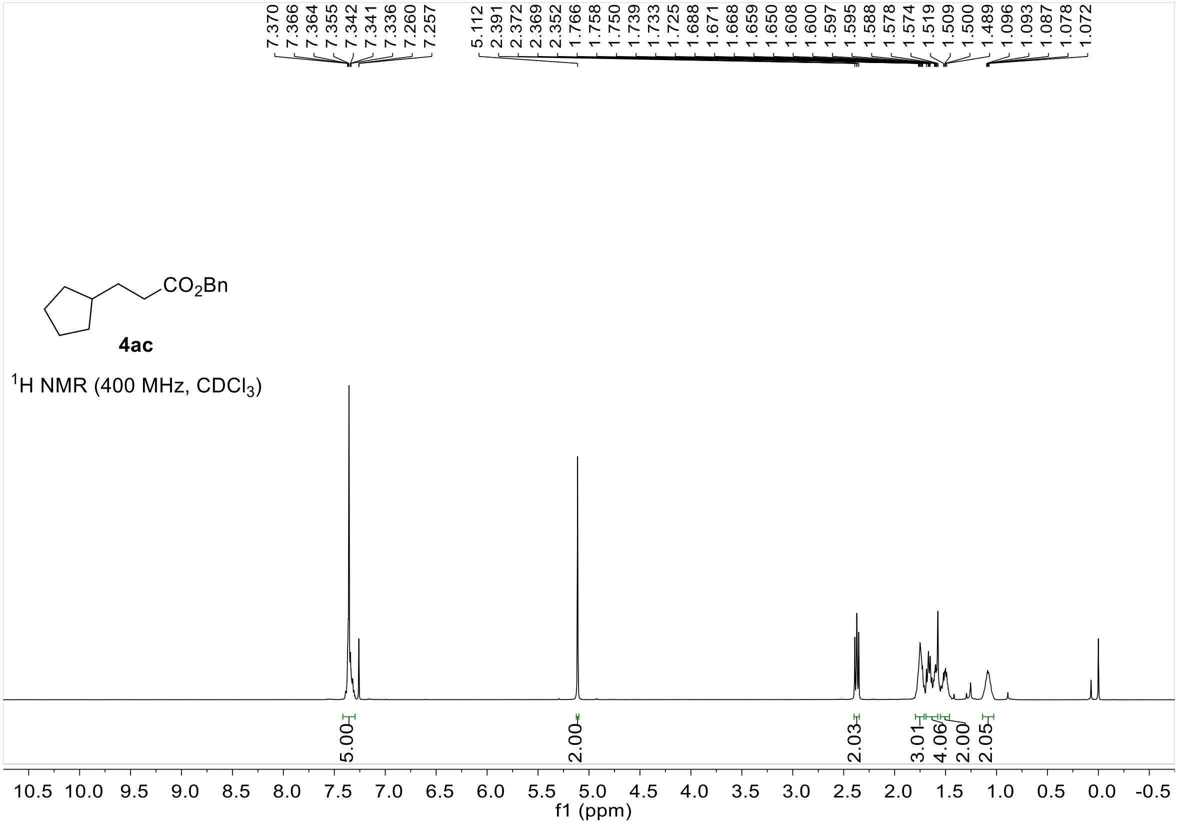

Supplementary Figure 15. ^{1}H NMR spectra of compound **4ac** (400 MHz, $CDCl_3$)

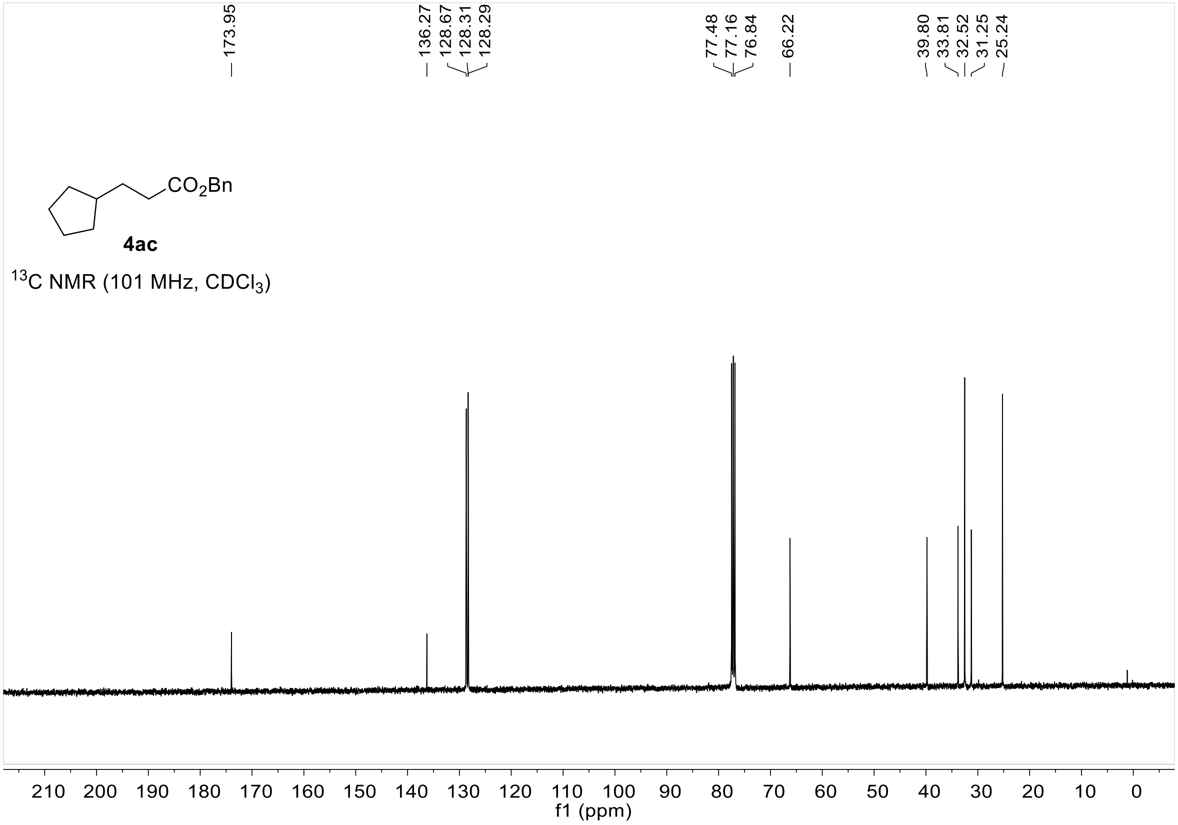

Supplementary Figure 16. ^{13}C NMR spectra of compound **4ac** (101 MHz, $CDCl_3$)

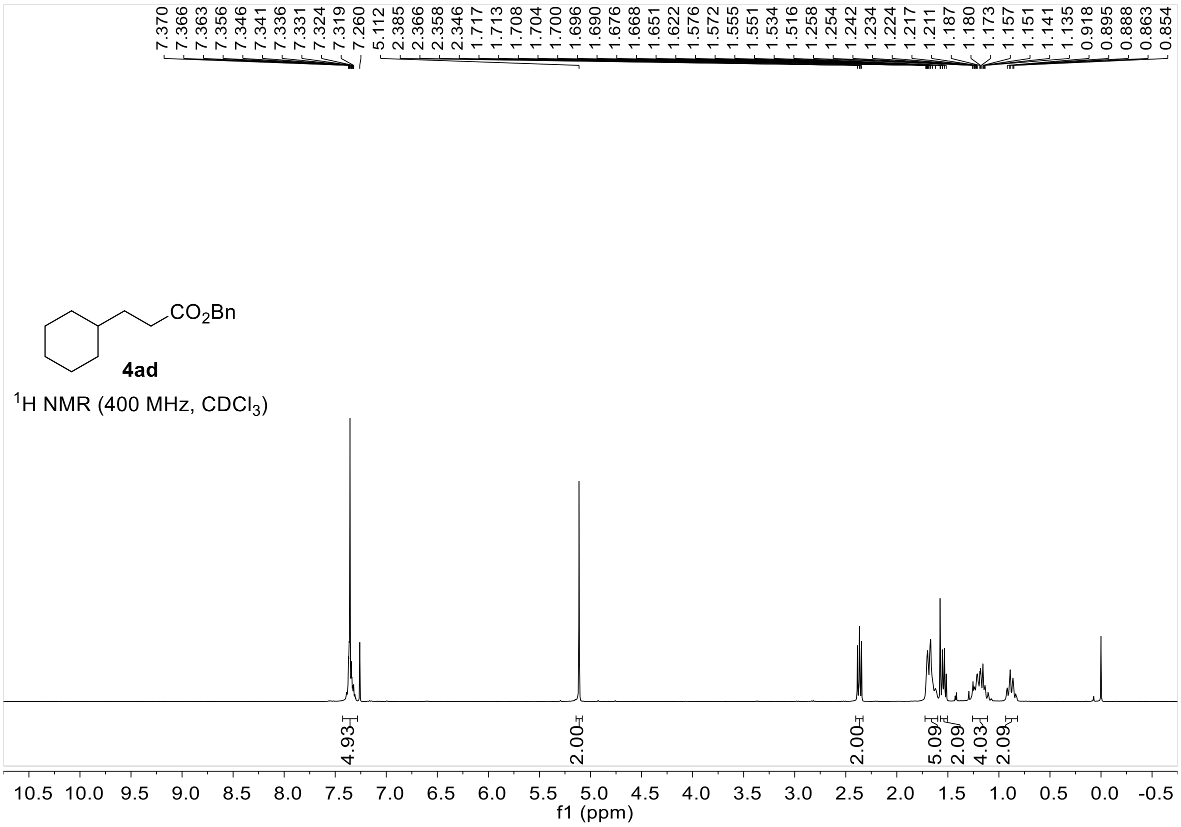

Supplementary Figure 17. ^{1}H NMR spectra of compound **4ad** (400 MHz, $CDCl_3$)

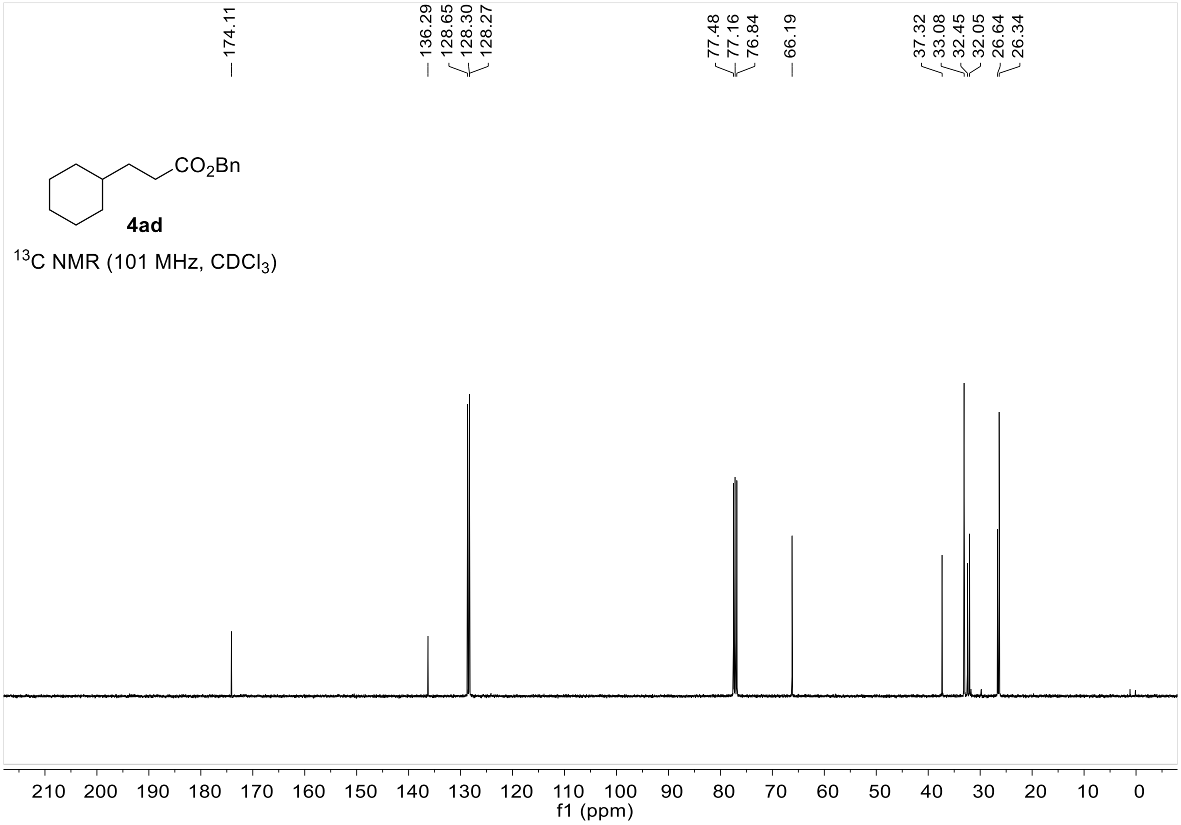

Supplementary Figure 18. ^{13}C NMR spectra of compound **4ad** (101 MHz, $CDCl_3$)

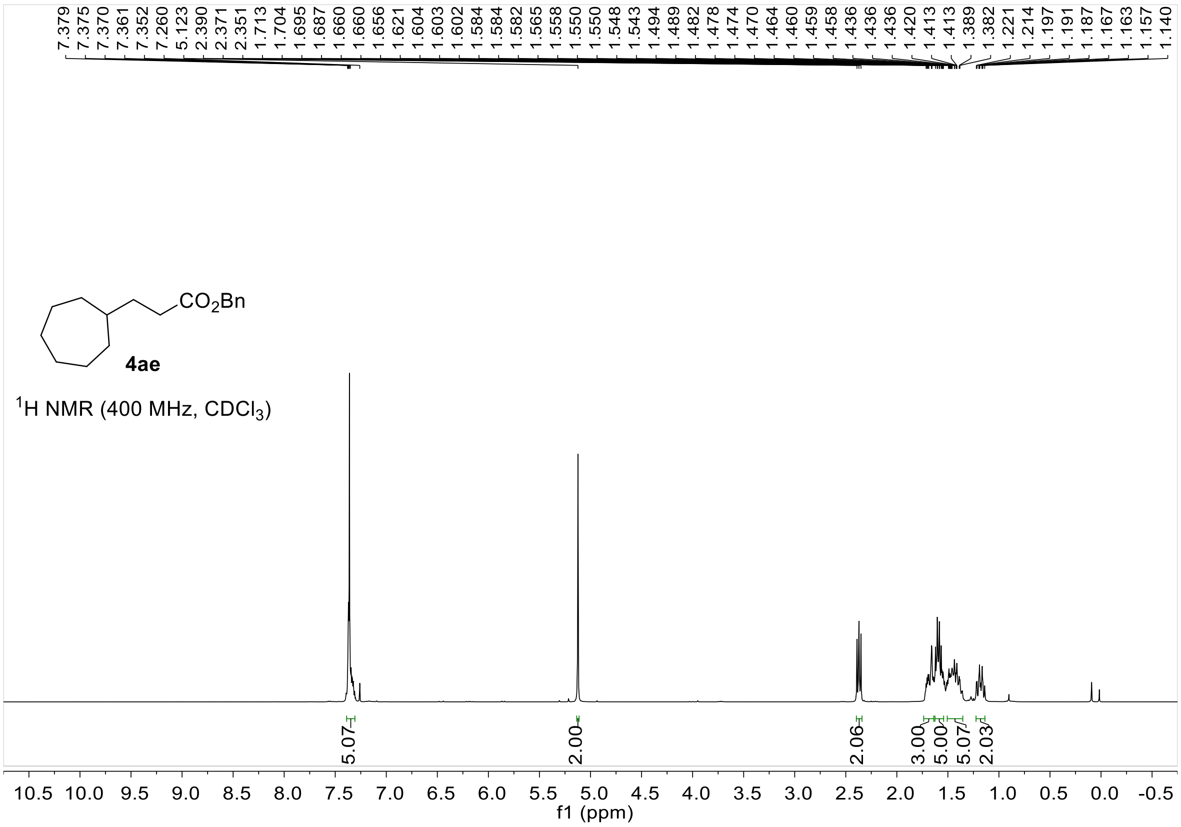

Supplementary Figure 19. ^{1}H NMR spectra of compound **4ae** (400 MHz, $CDCl_3$)

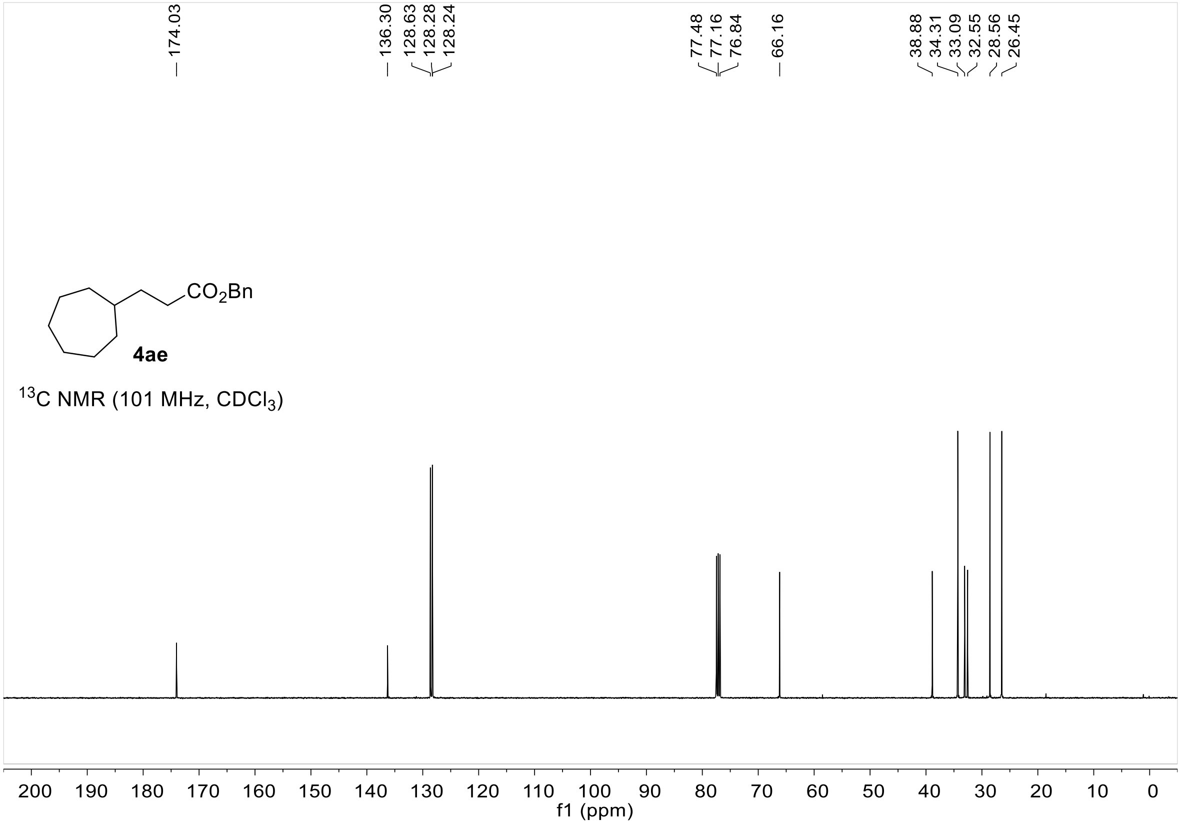

Supplementary Figure 20. ^{13}C NMR spectra of compound **4ae** (101 MHz, $CDCl_3$)

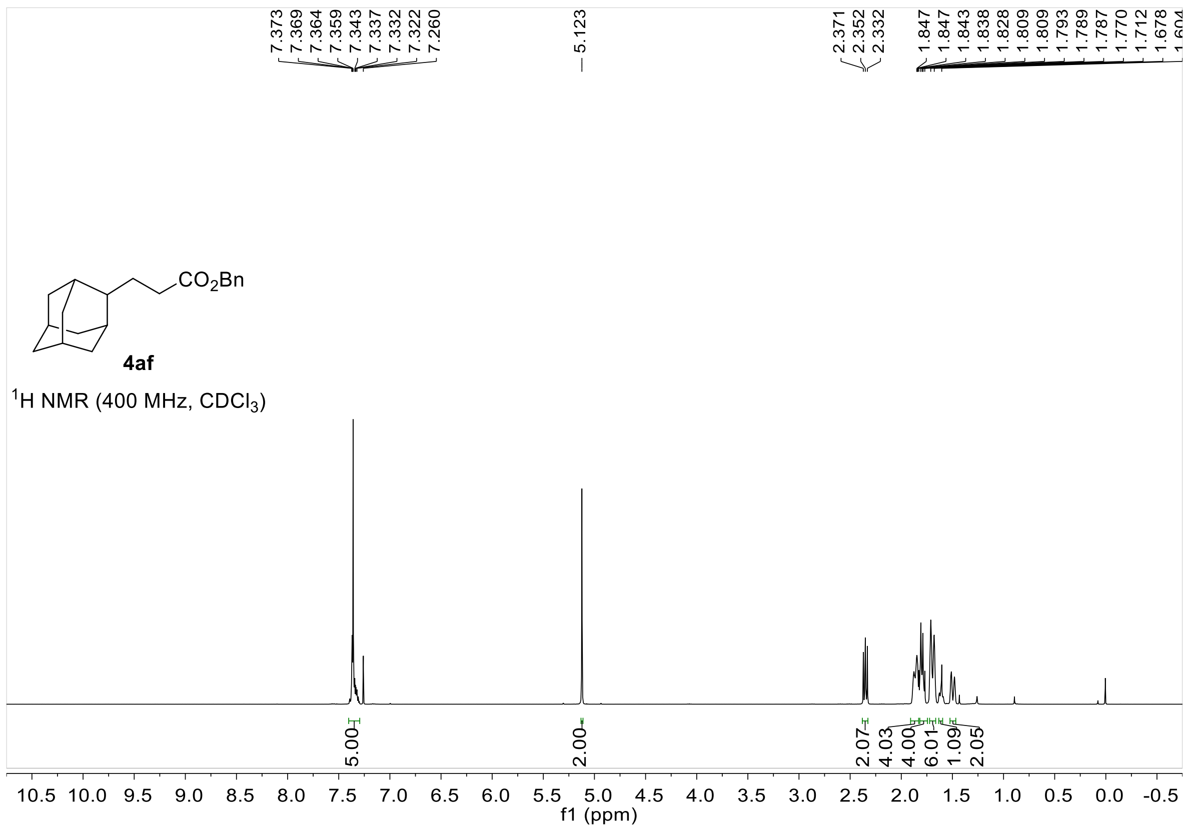

Supplementary Figure 21. ^{1}H NMR spectra of compound **4af** (400 MHz, $CDCl_3$)

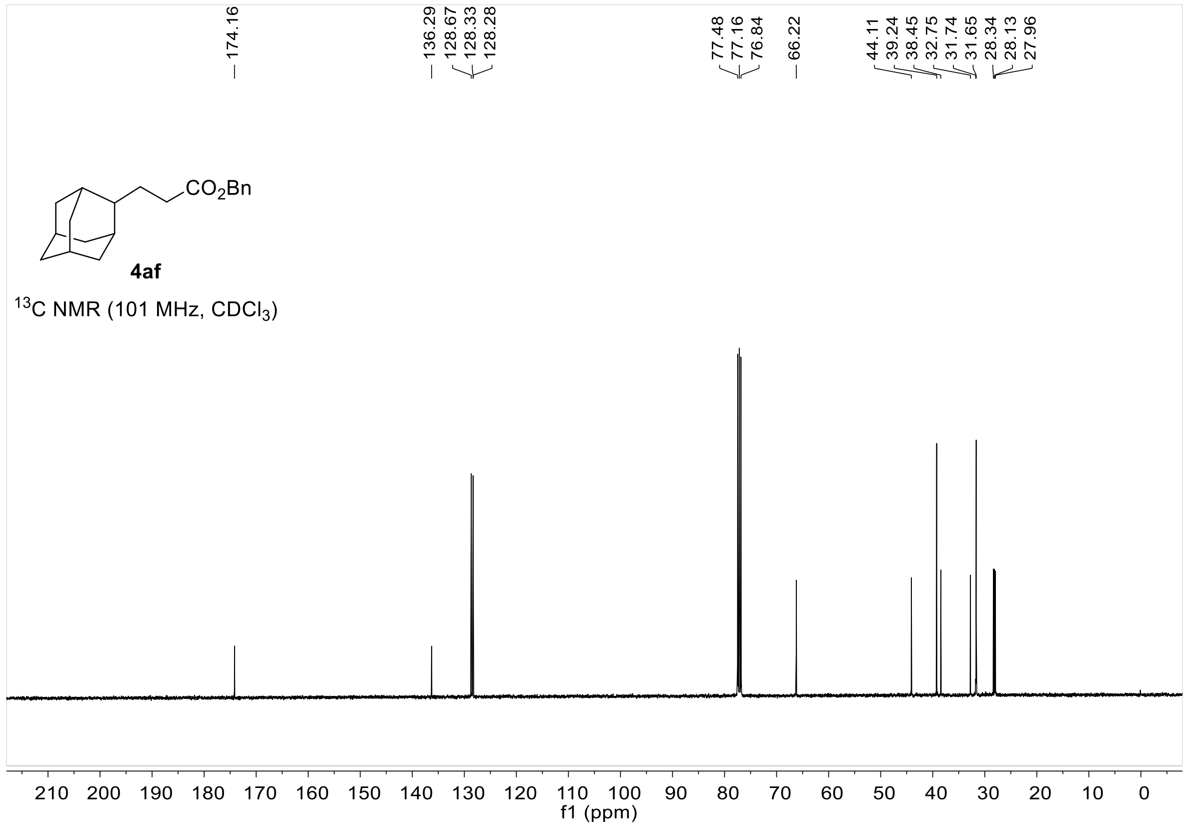

Supplementary Figure 22. ^{13}C NMR spectra of compound **4af** (101 MHz, $CDCl_3$)

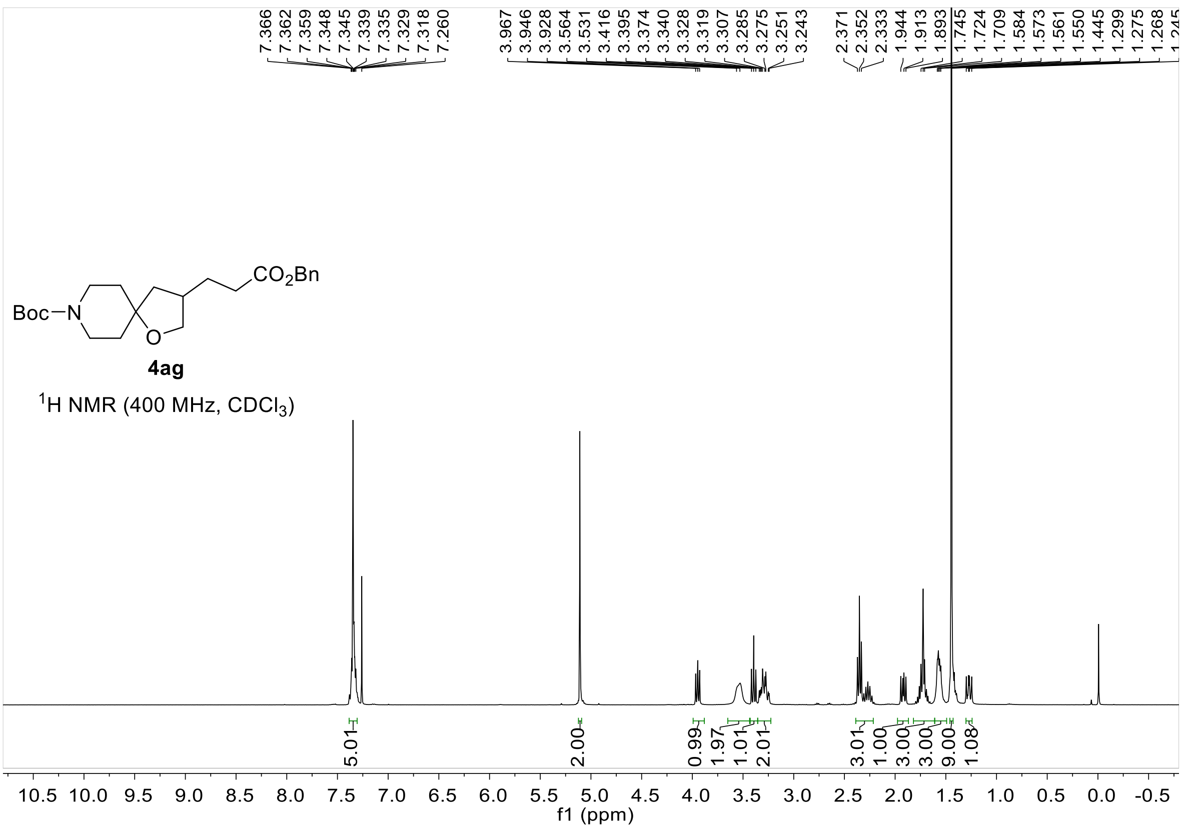

Supplementary Figure 23. ^{1}H NMR spectra of compound **4ag** (400 MHz, $CDCl_3$)

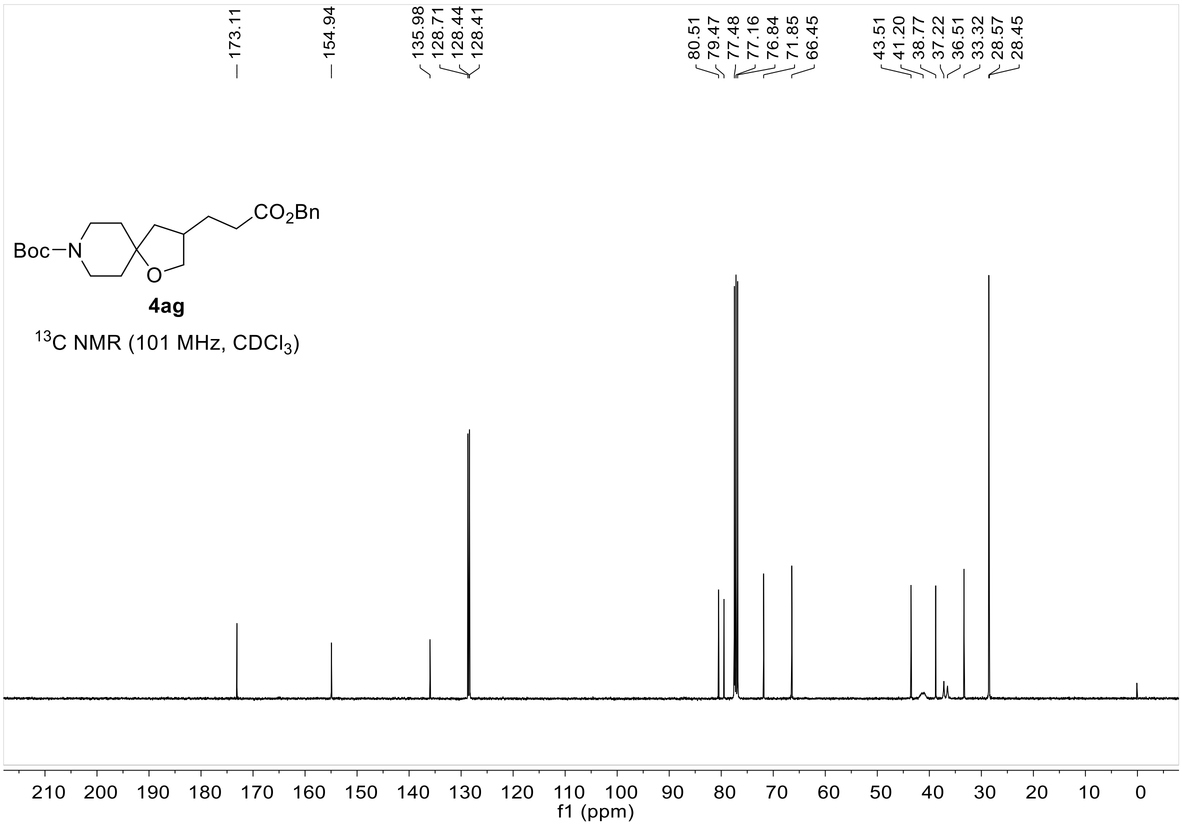

Supplementary Figure 24. ^{13}C NMR spectra of compound **4ag** (101 MHz, $CDCl_3$)

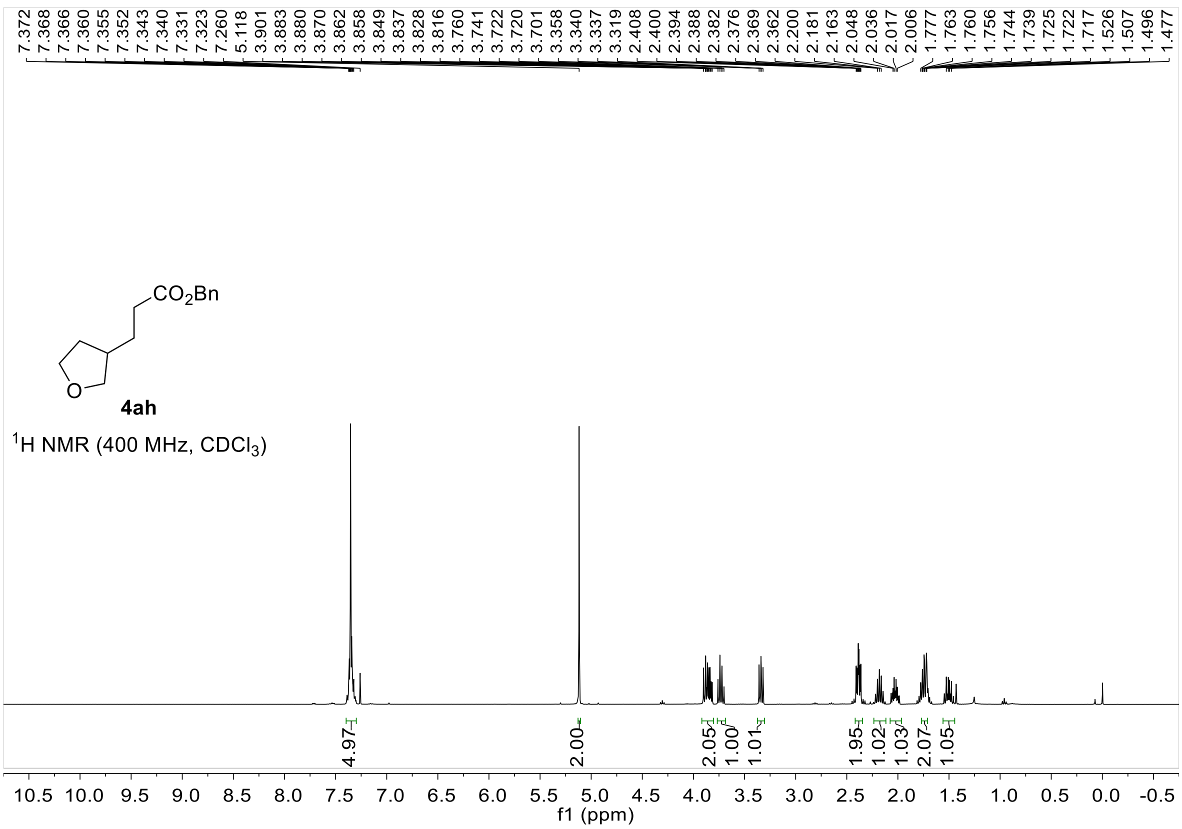

Supplementary Figure 25. ^{1}H NMR spectra of compound **4ah** (400 MHz, $CDCl_3$)

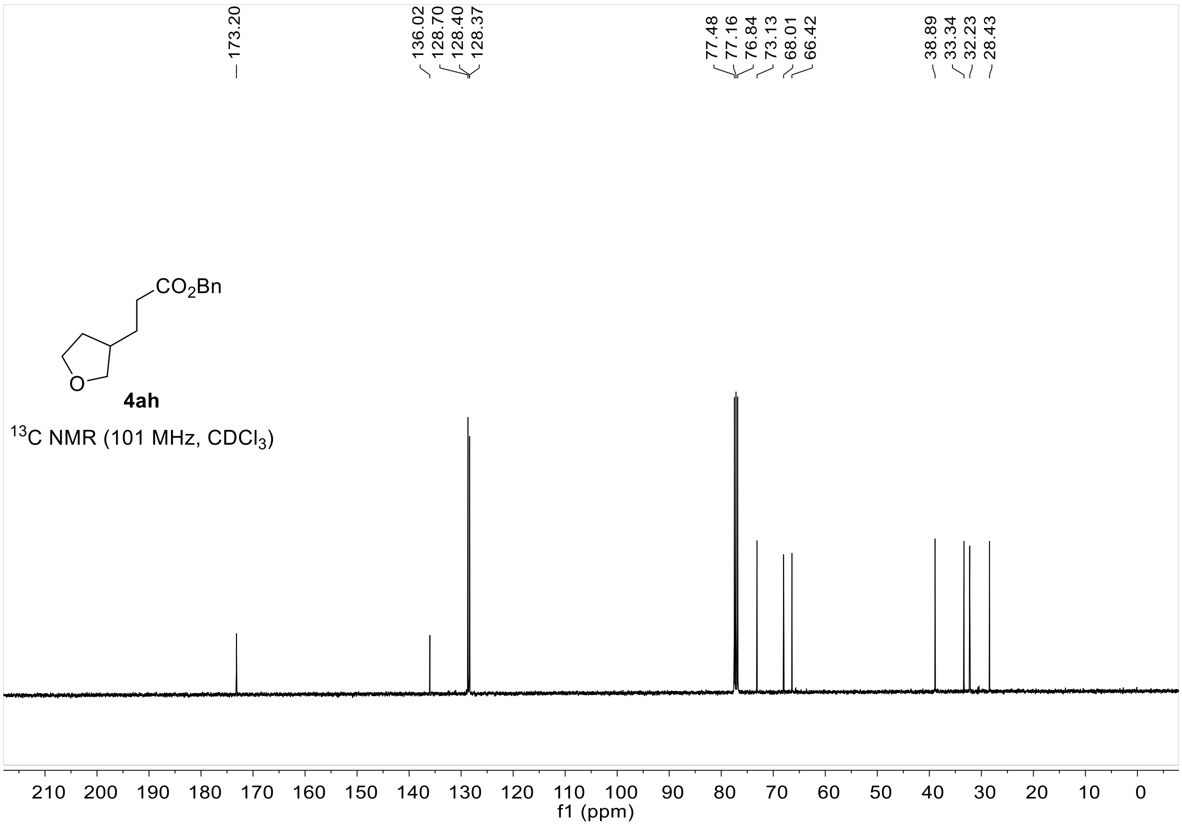

Supplementary Figure 26. ^{13}C NMR spectra of compound **4ah** (101 MHz, $CDCl_3$)

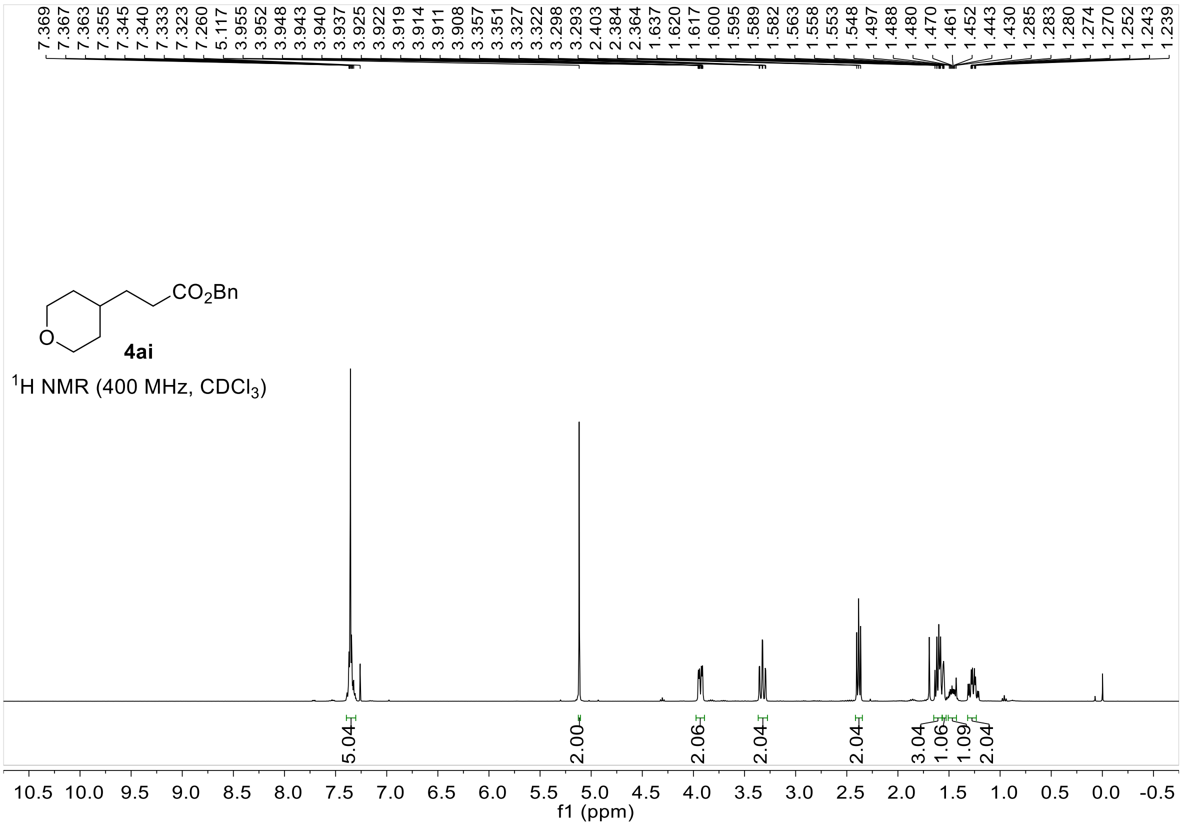

Supplementary Figure 27. ^{1}H NMR spectra of compound **4ai** (400 MHz, $CDCl_3$)

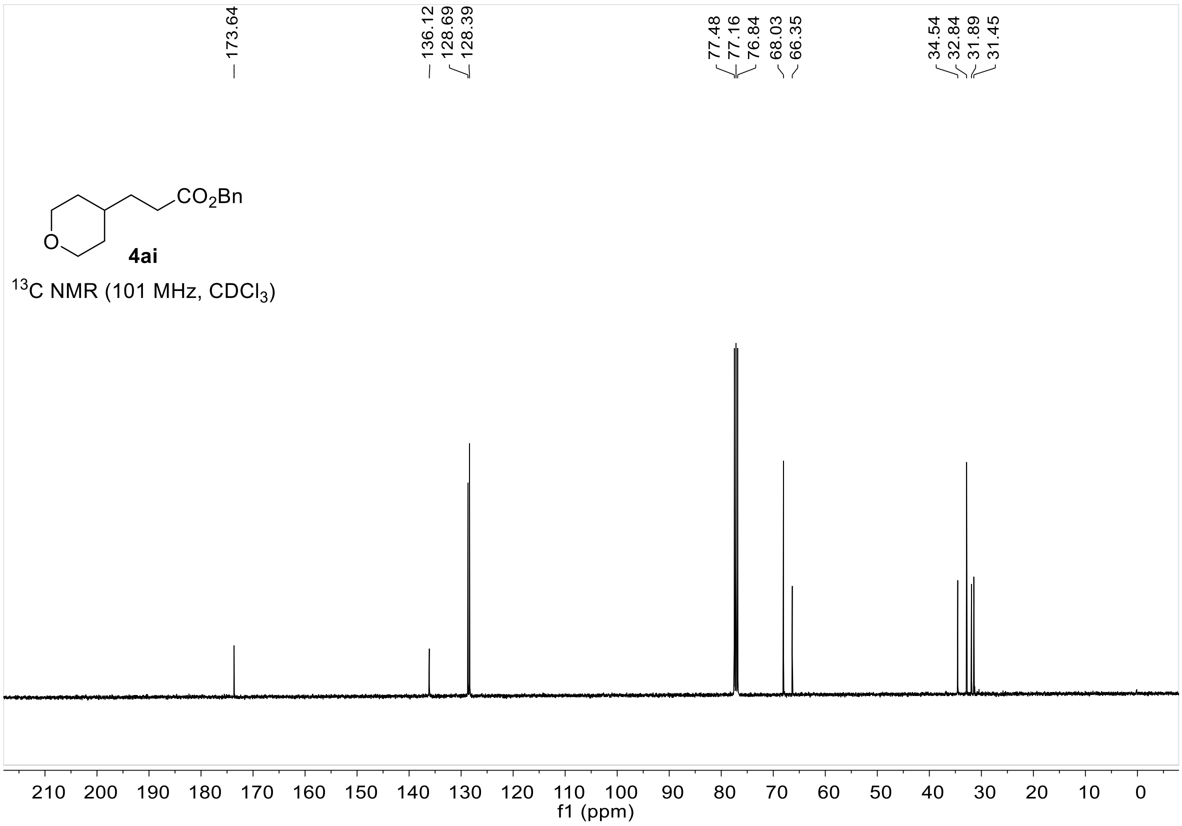

Supplementary Figure 28. ^{13}C NMR spectra of compound **4ai** (101 MHz, $CDCl_3$)

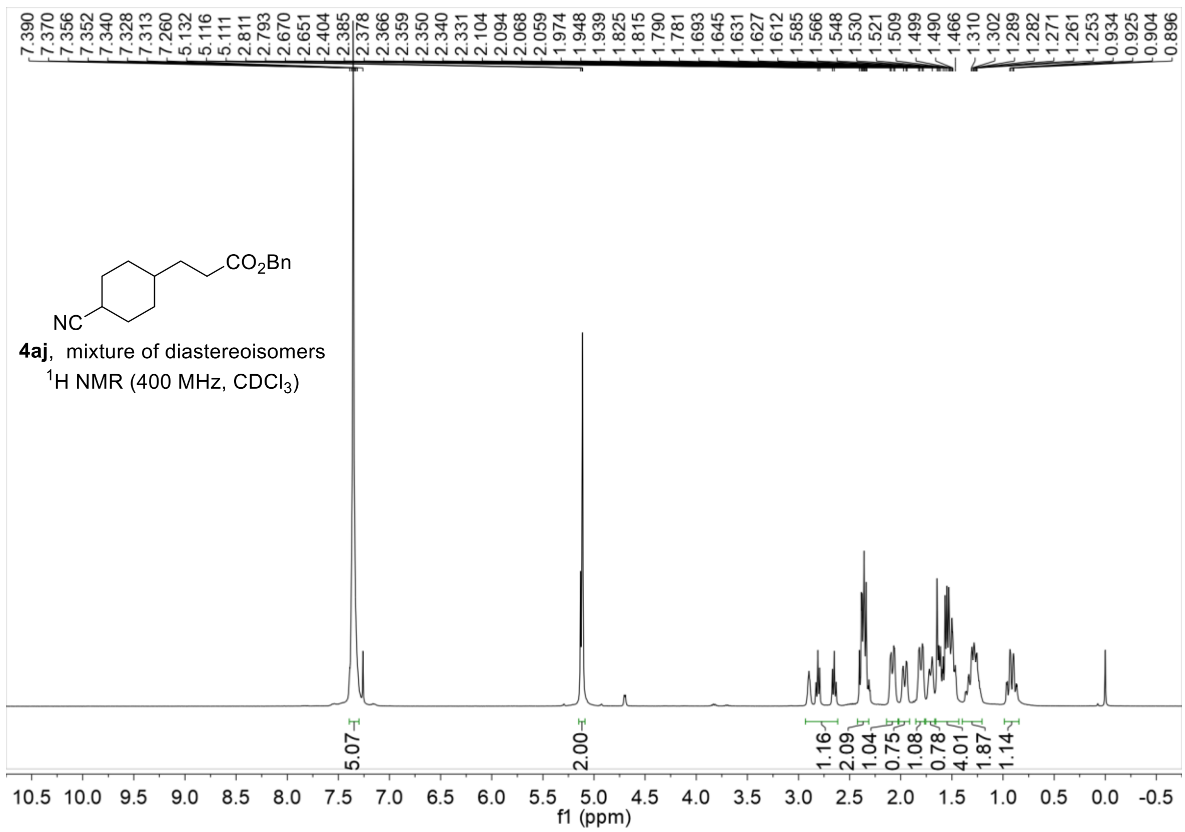

Supplementary Figure 29. ^{1}H NMR spectra of compound **4aj** (400 MHz, $CDCl_3$)

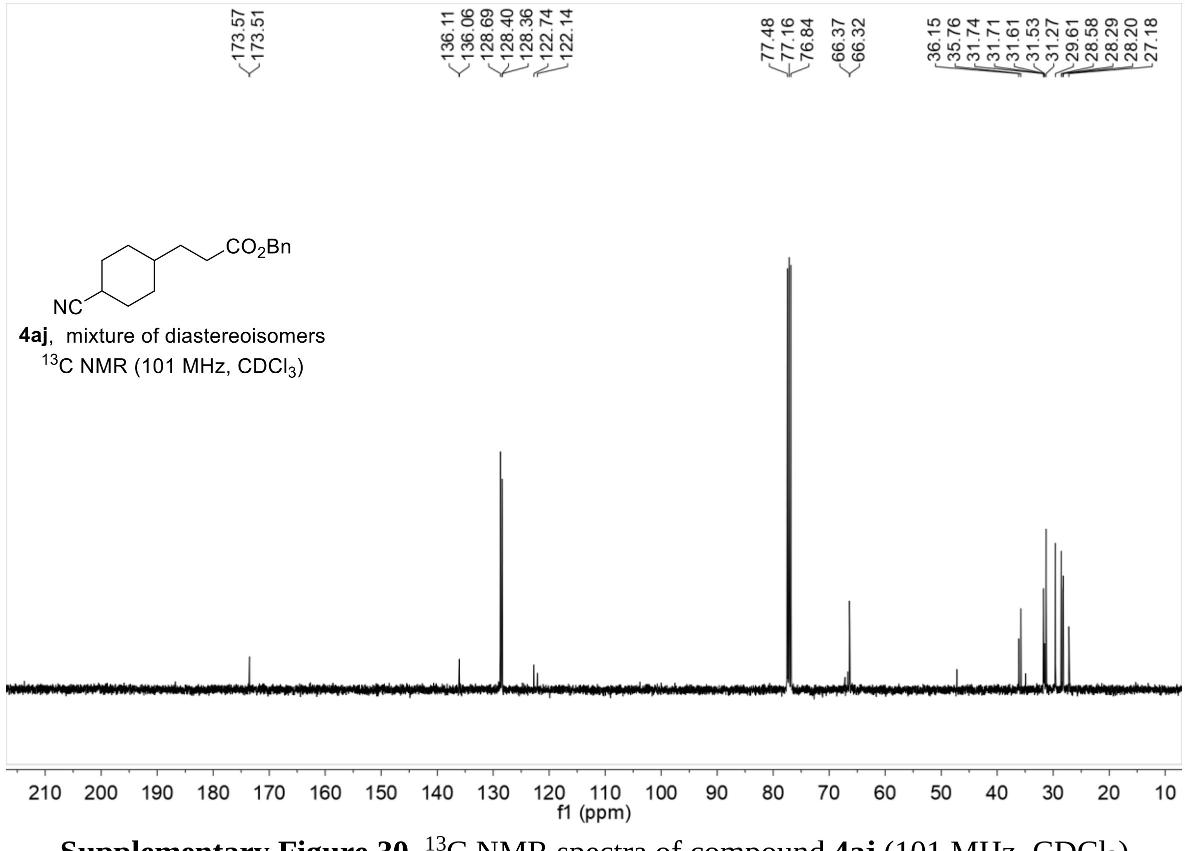

Supplementary Figure 30. ^{13}C NMR spectra of compound **4aj** (101 MHz, $CDCl_3$)

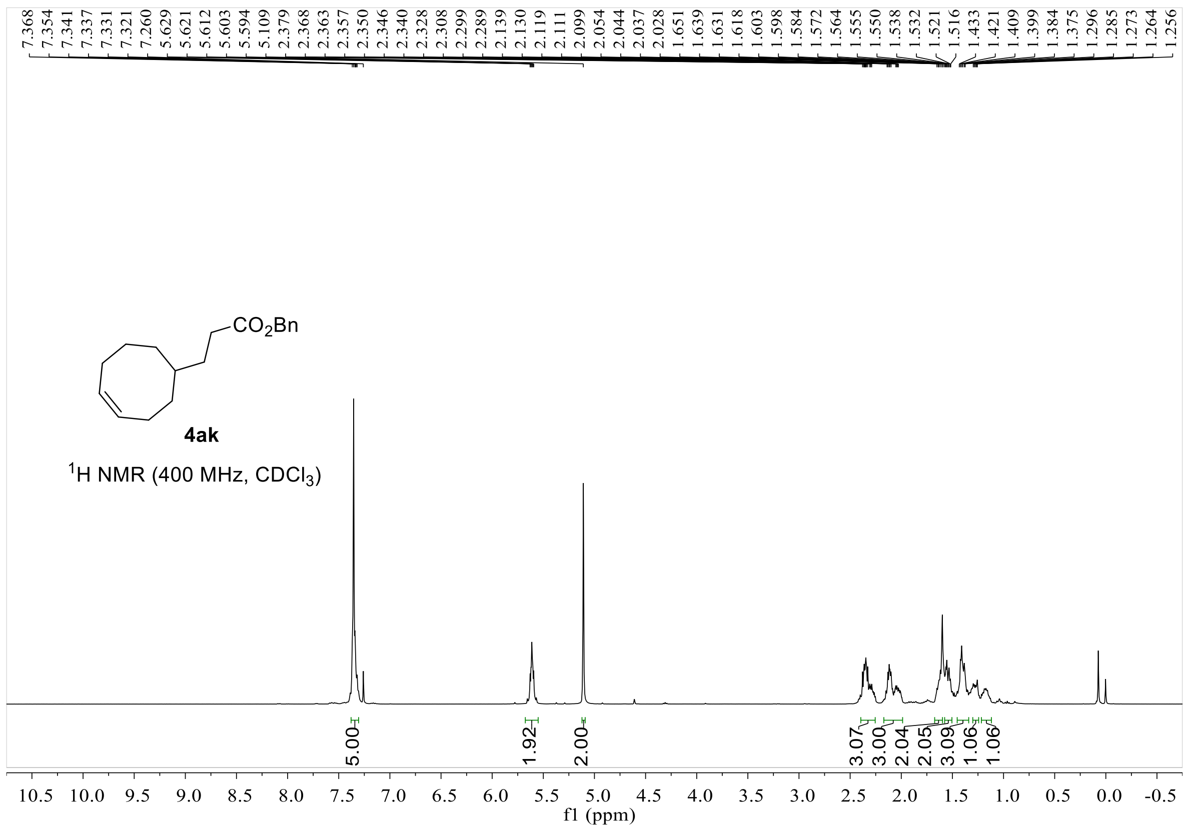

Supplementary Figure 31. ^{1}H NMR spectra of compound **4ak** (400 MHz, $CDCl_3$)

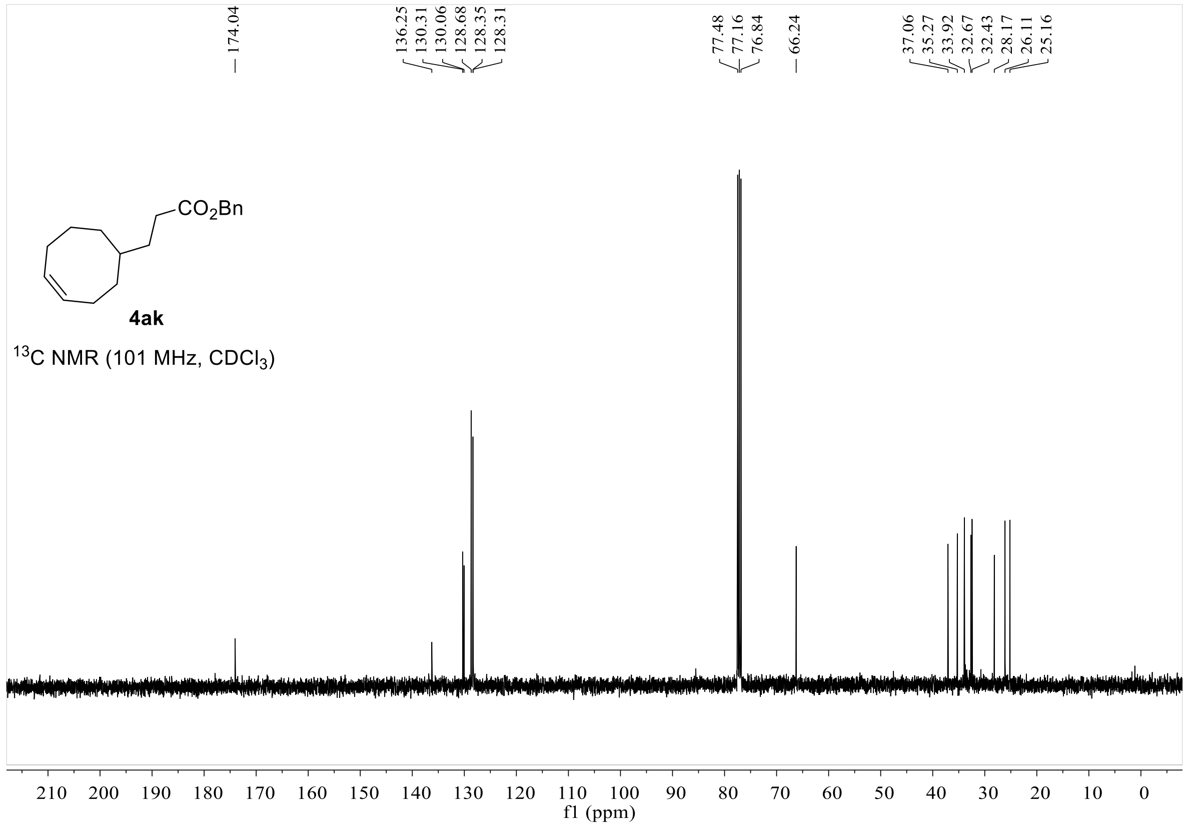

Supplementary Figure 32. ^{13}C NMR spectra of compound **4ak** (101 MHz, $CDCl_3$)

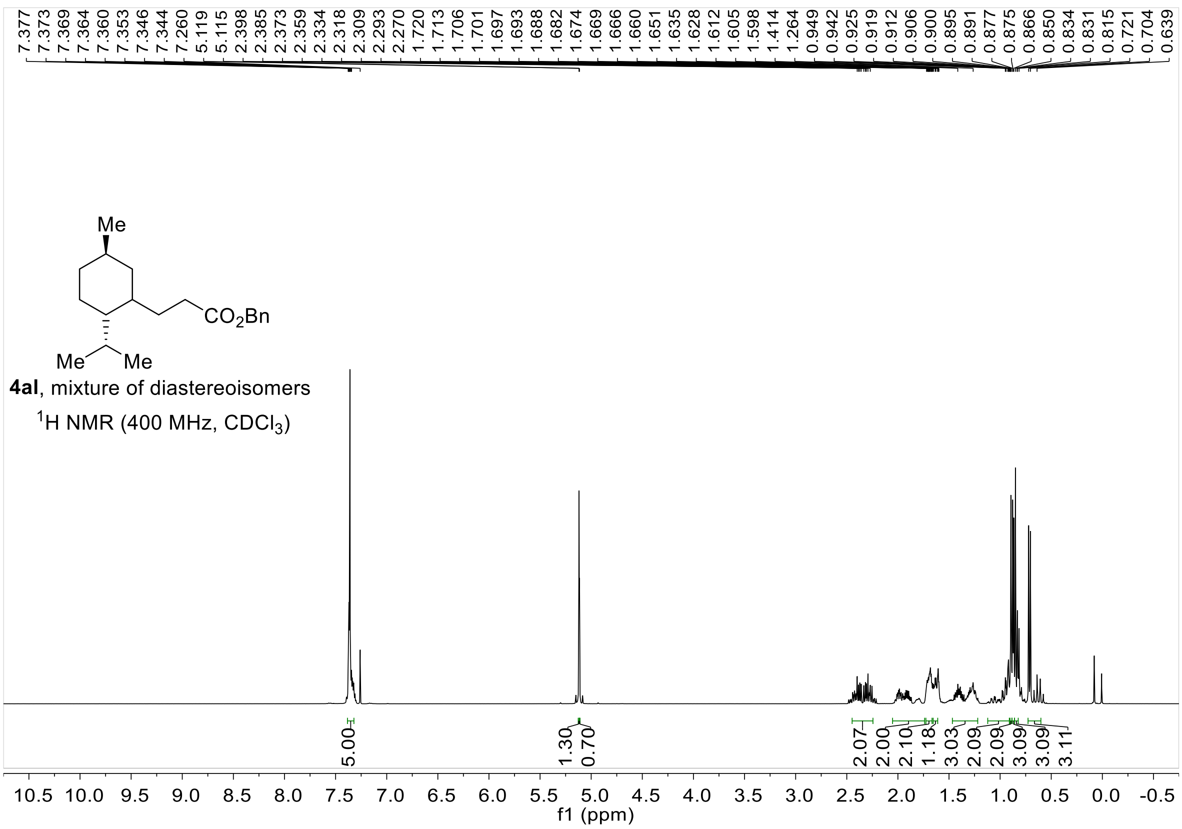

Supplementary Figure 33. ^{1}H NMR spectra of compound **4al** (400 MHz, $CDCl_3$)

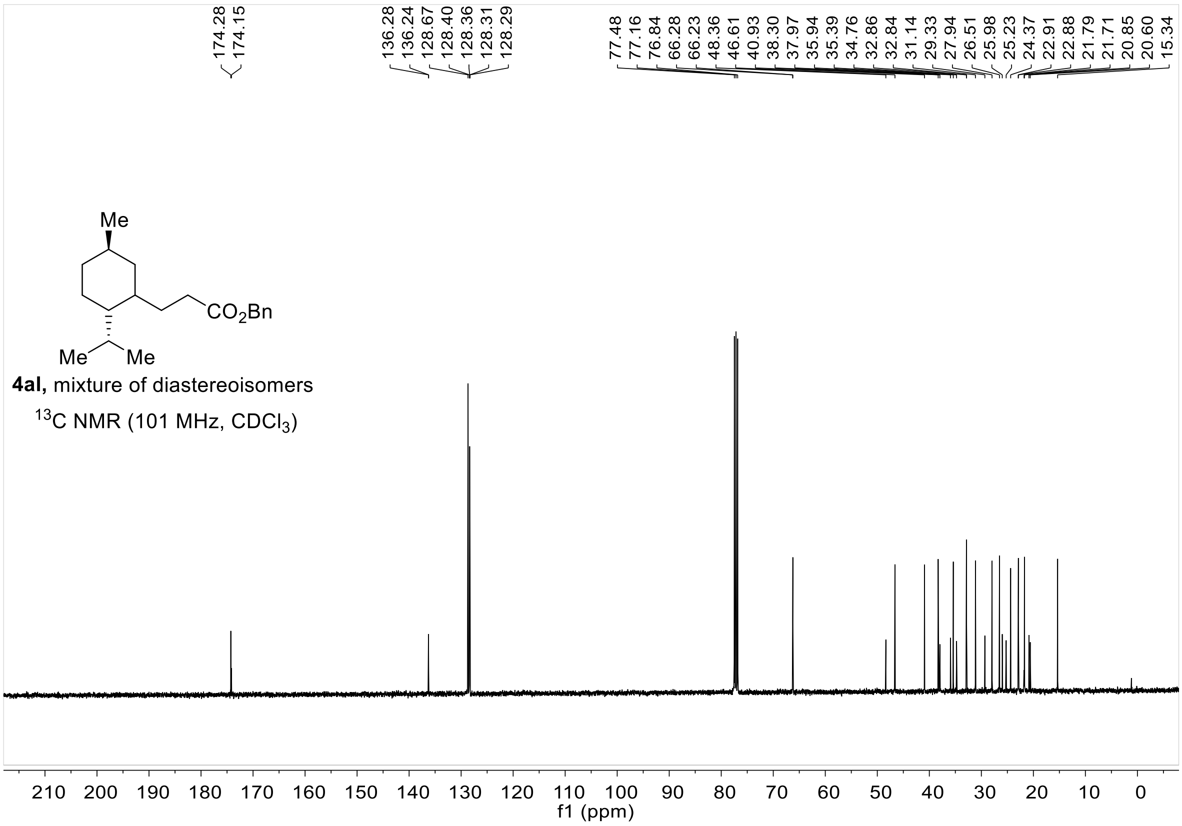

Supplementary Figure 34. ^{13}C NMR spectra of compound **4al** (101 MHz, $CDCl_3$)

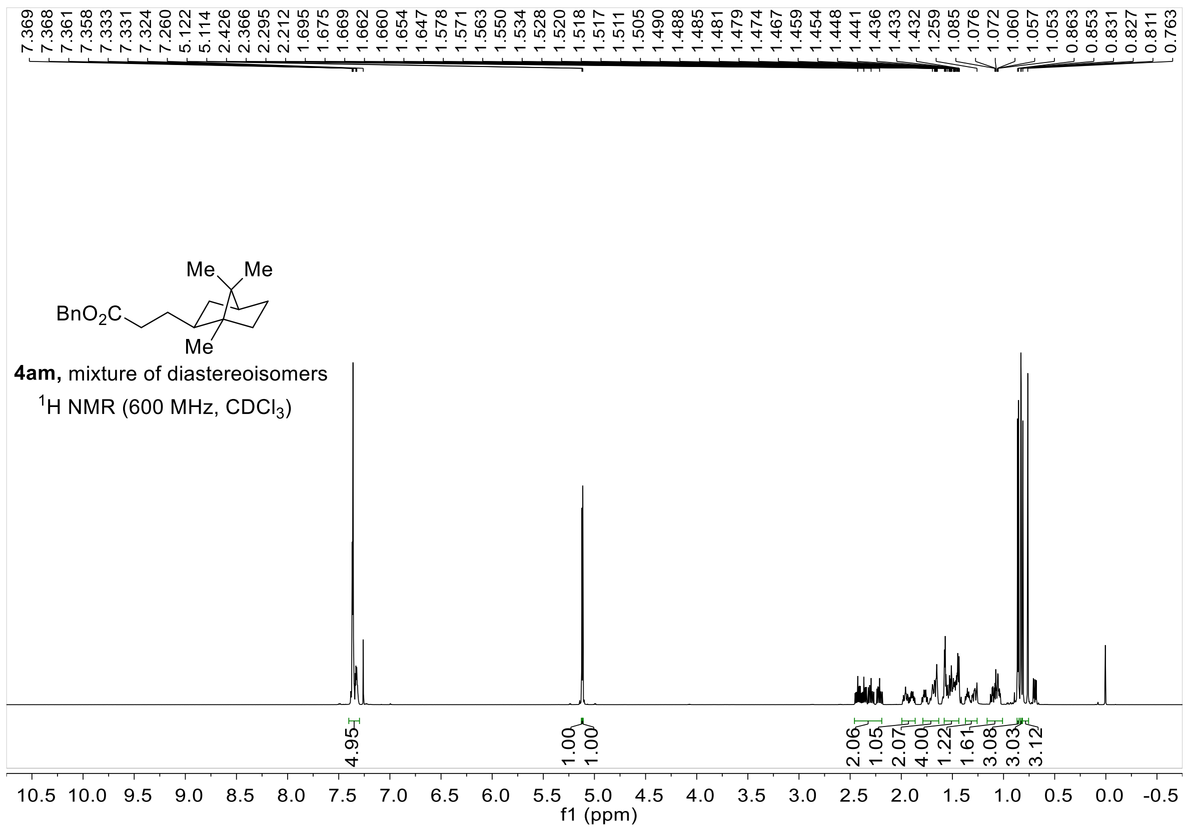

Supplementary Figure 35. ^{1}H NMR spectra of compound **4am** (600 MHz, $CDCl_3$)

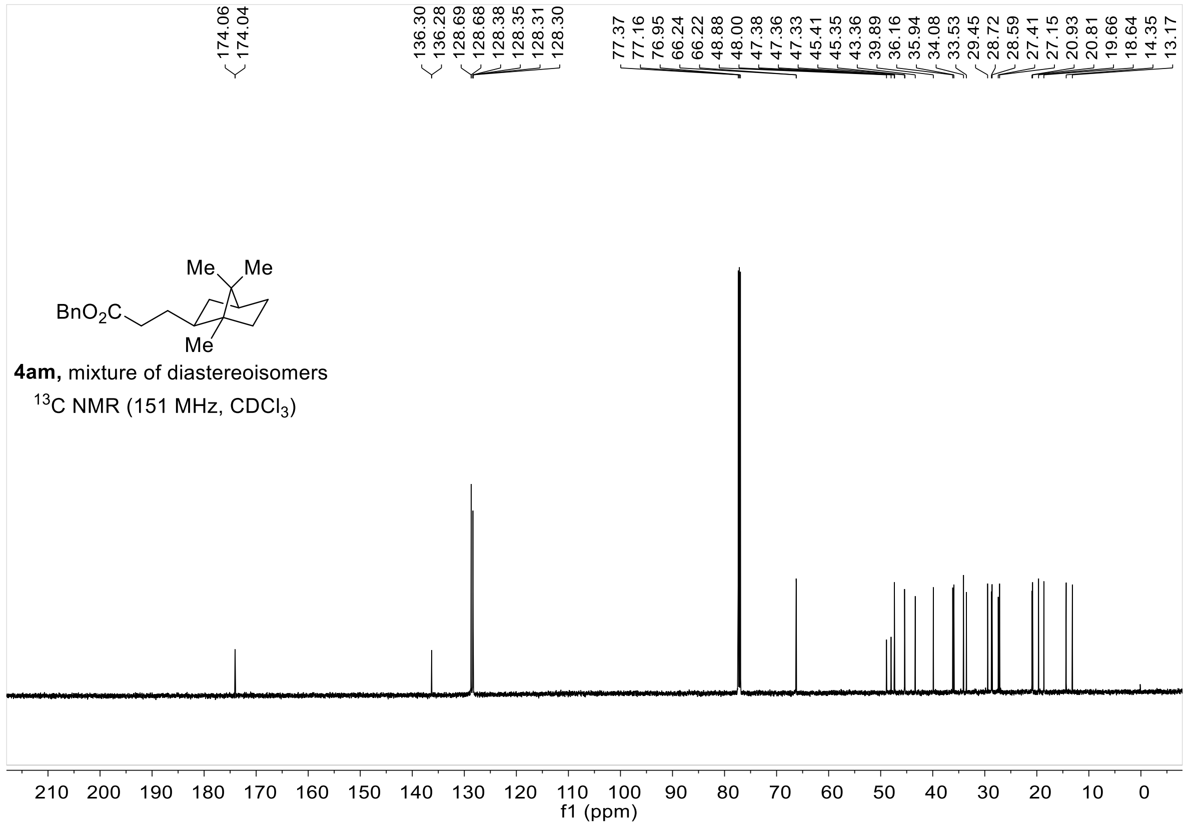

Supplementary Figure 36. ^{13}C NMR spectra of compound **4am** (151 MHz, $CDCl_3$)

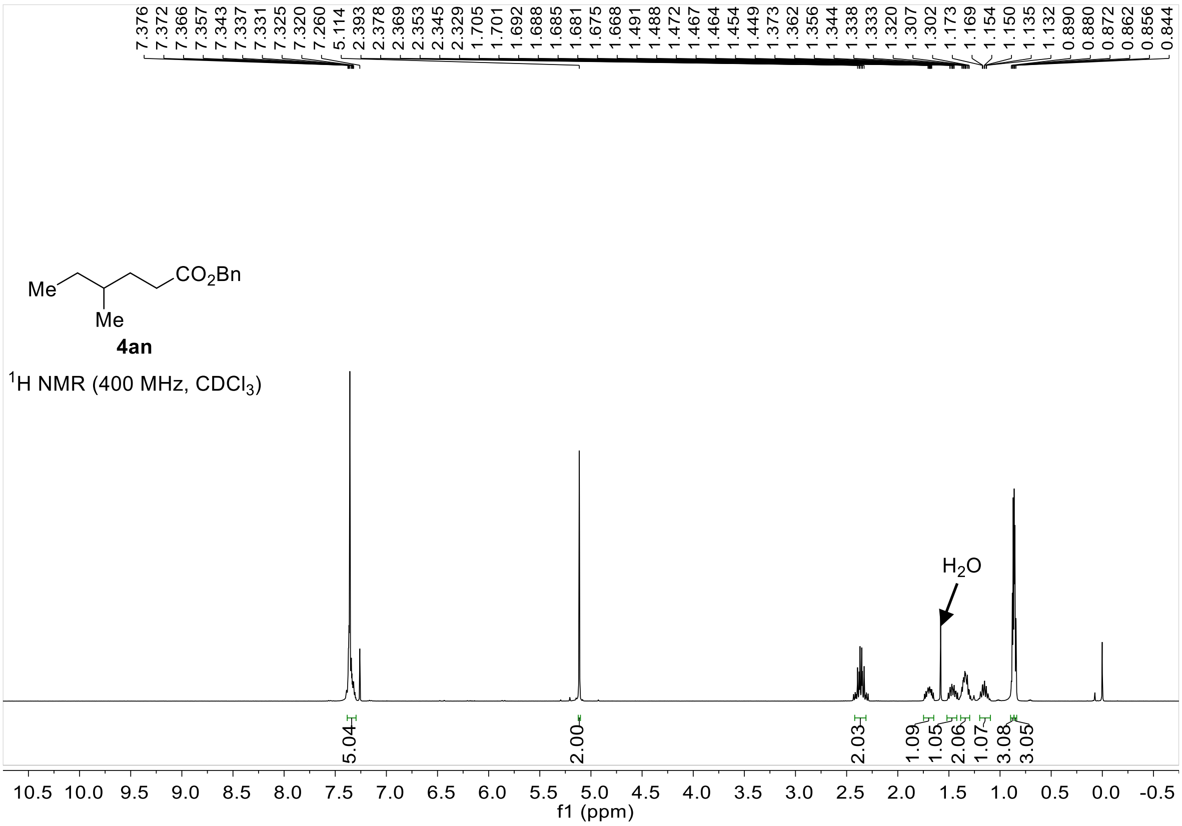

Supplementary Figure 37. ^{1}H NMR spectra of compound **4an** (400 MHz, $CDCl_3$)

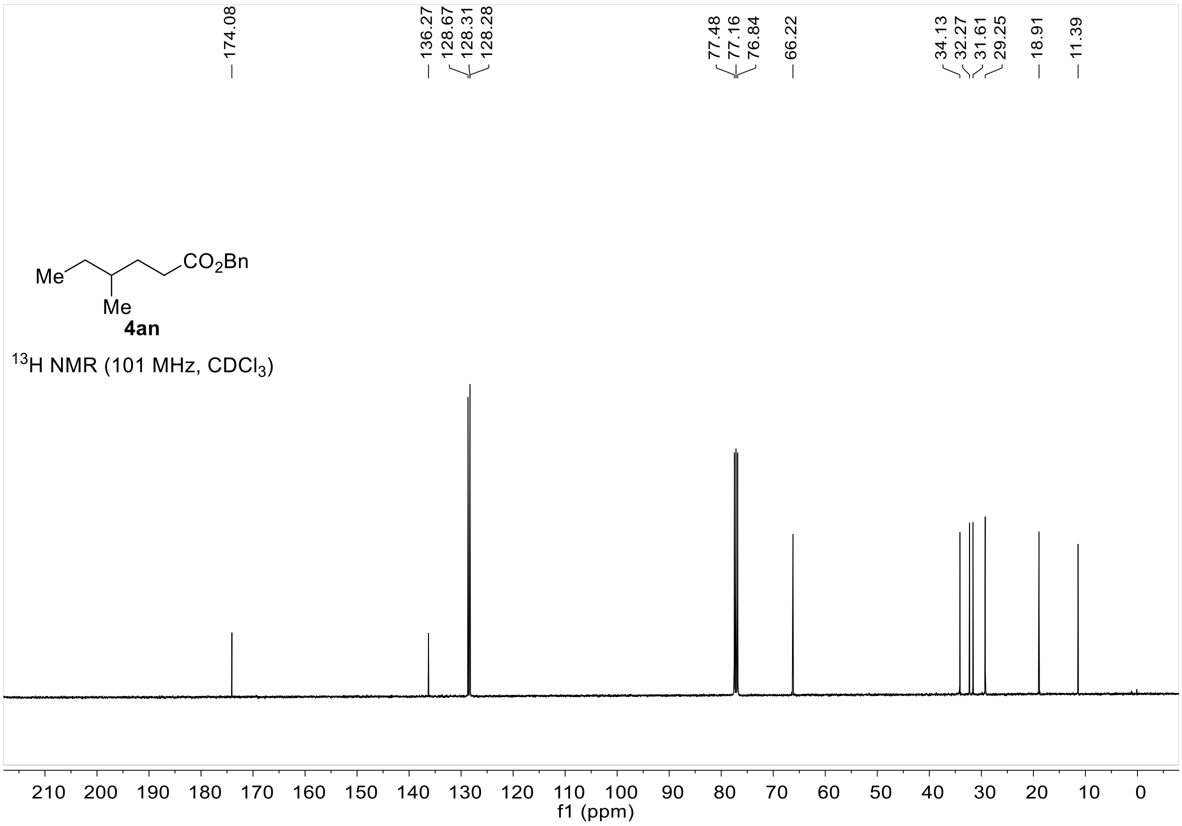

Supplementary Figure 38. ^{13}C NMR spectra of compound **4an** (101 MHz, $CDCl_3$)

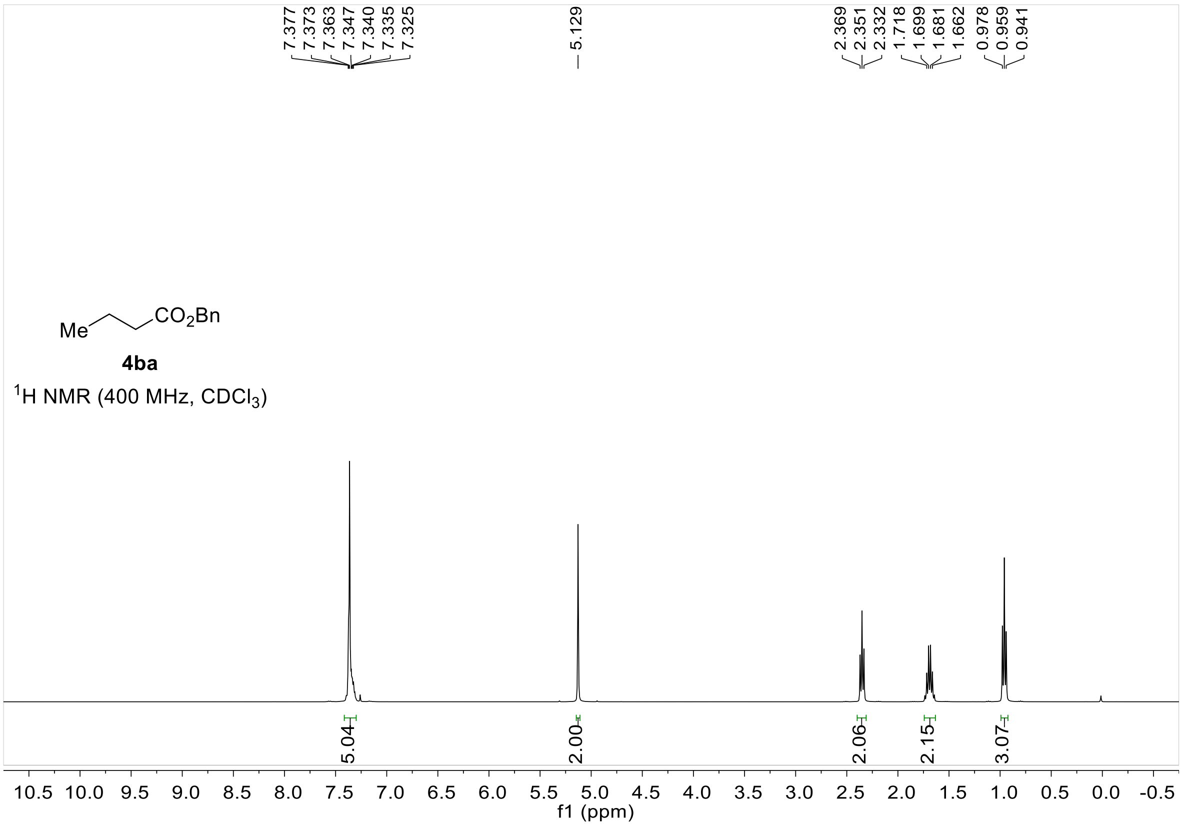

Supplementary Figure 39. ^{1}H NMR spectra of compound **4ba** (400 MHz, $CDCl_3$)

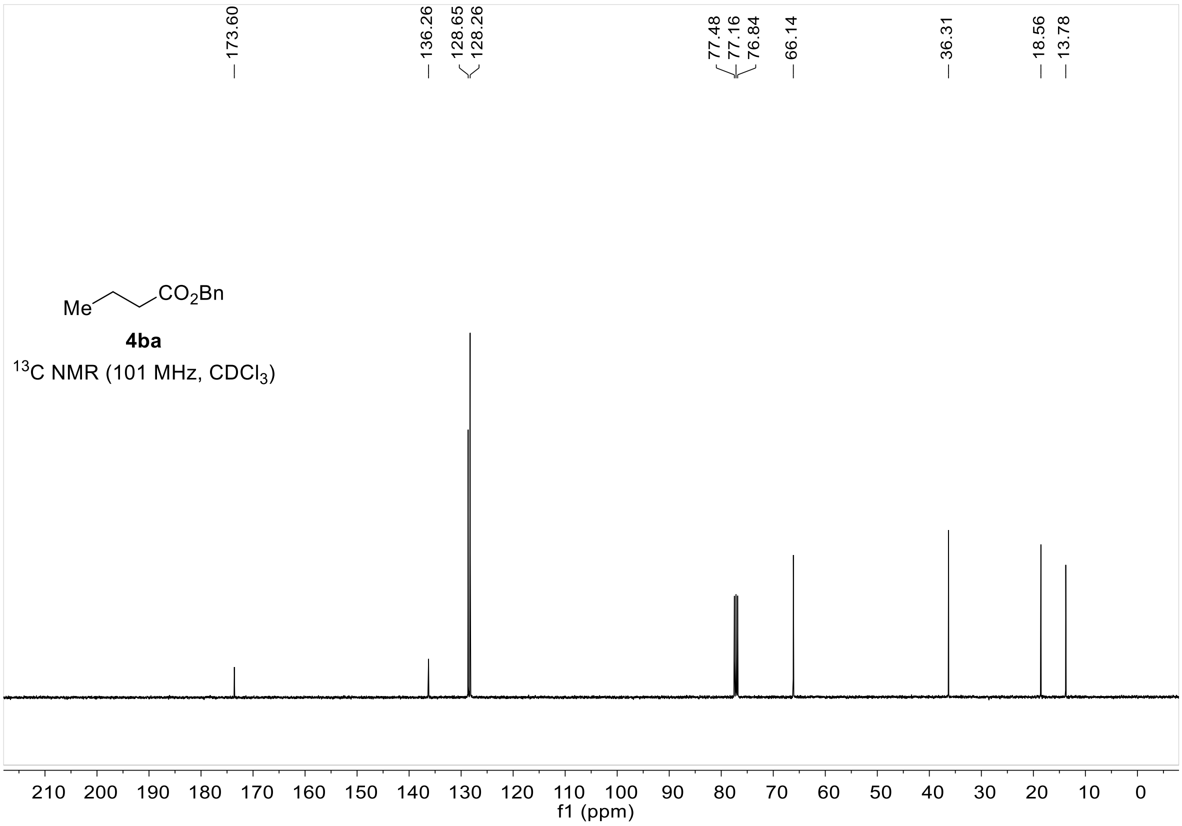

Supplementary Figure 40. ^{13}C NMR spectra of compound **4ba** (101 MHz, $CDCl_3$)

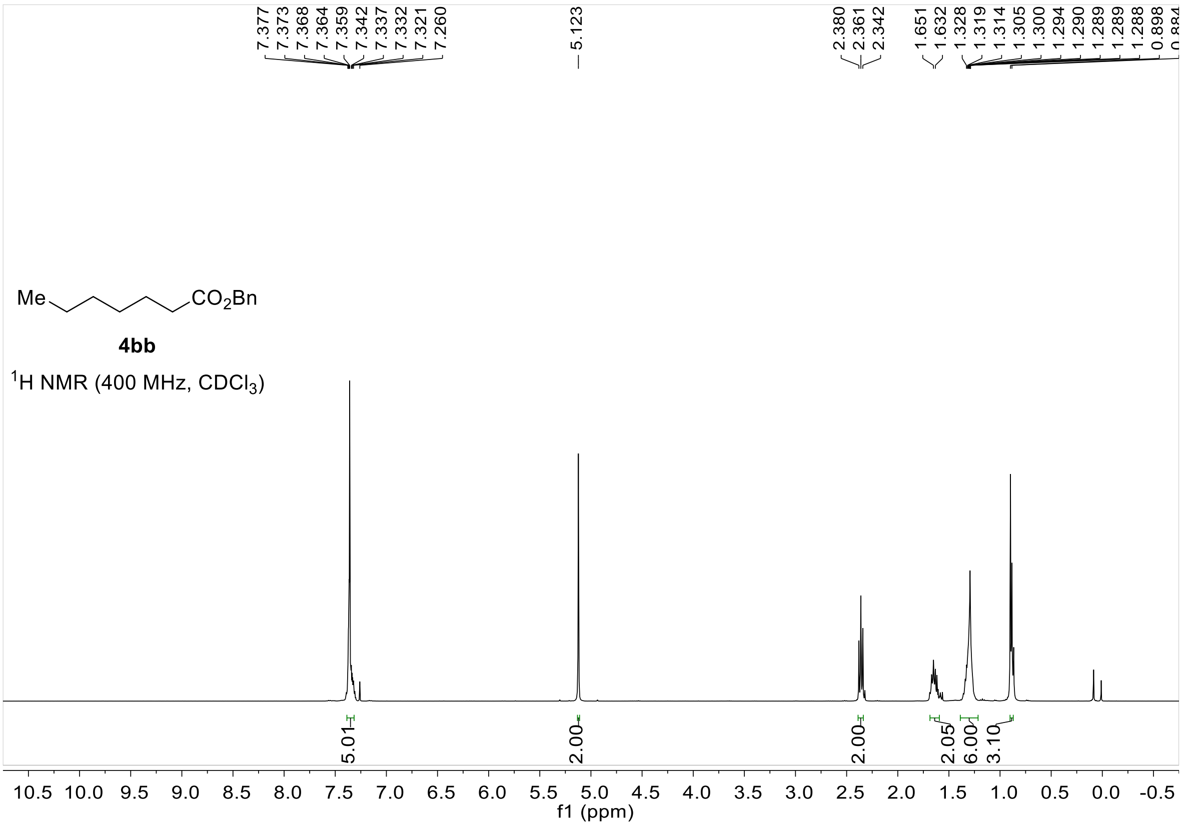

Supplementary Figure 41. ^{1}H NMR spectra of compound **4bb** (400 MHz, $CDCl_3$)

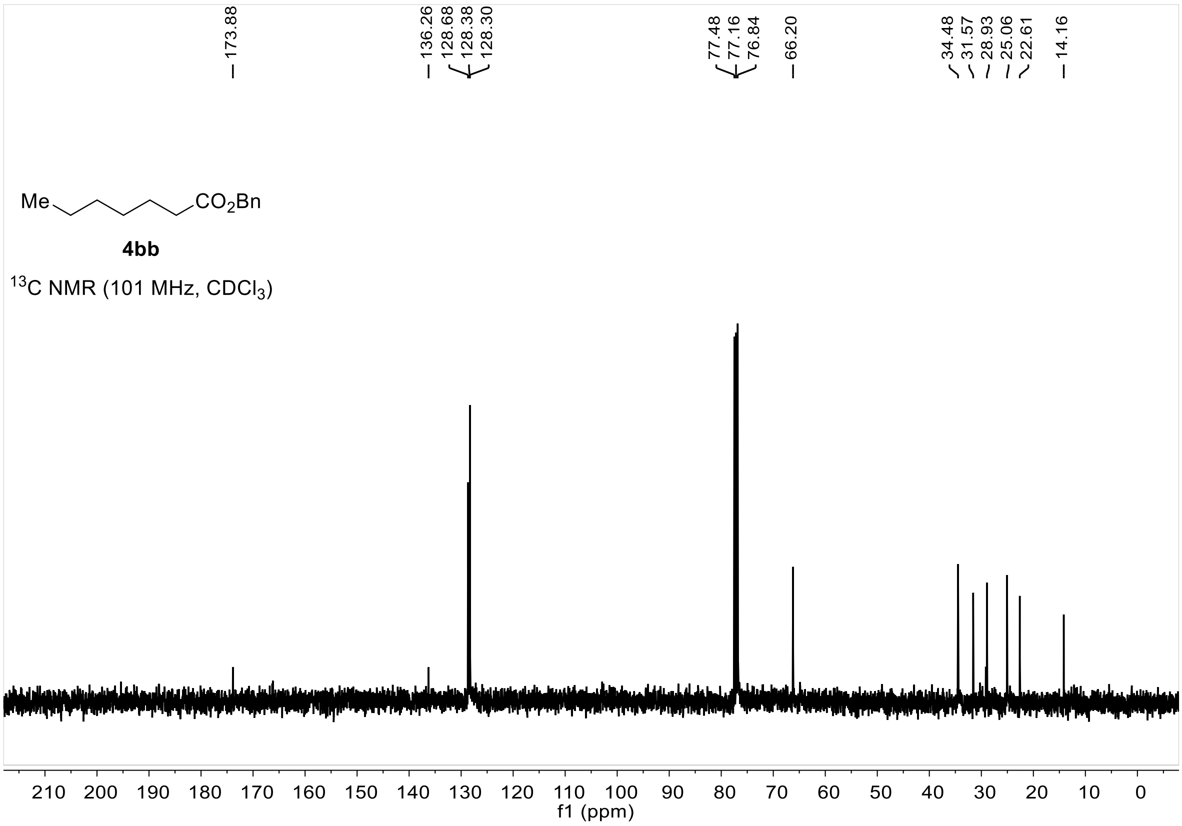

Supplementary Figure 42. ^{13}C NMR spectra of compound **4bb** (101 MHz, $CDCl_3$)

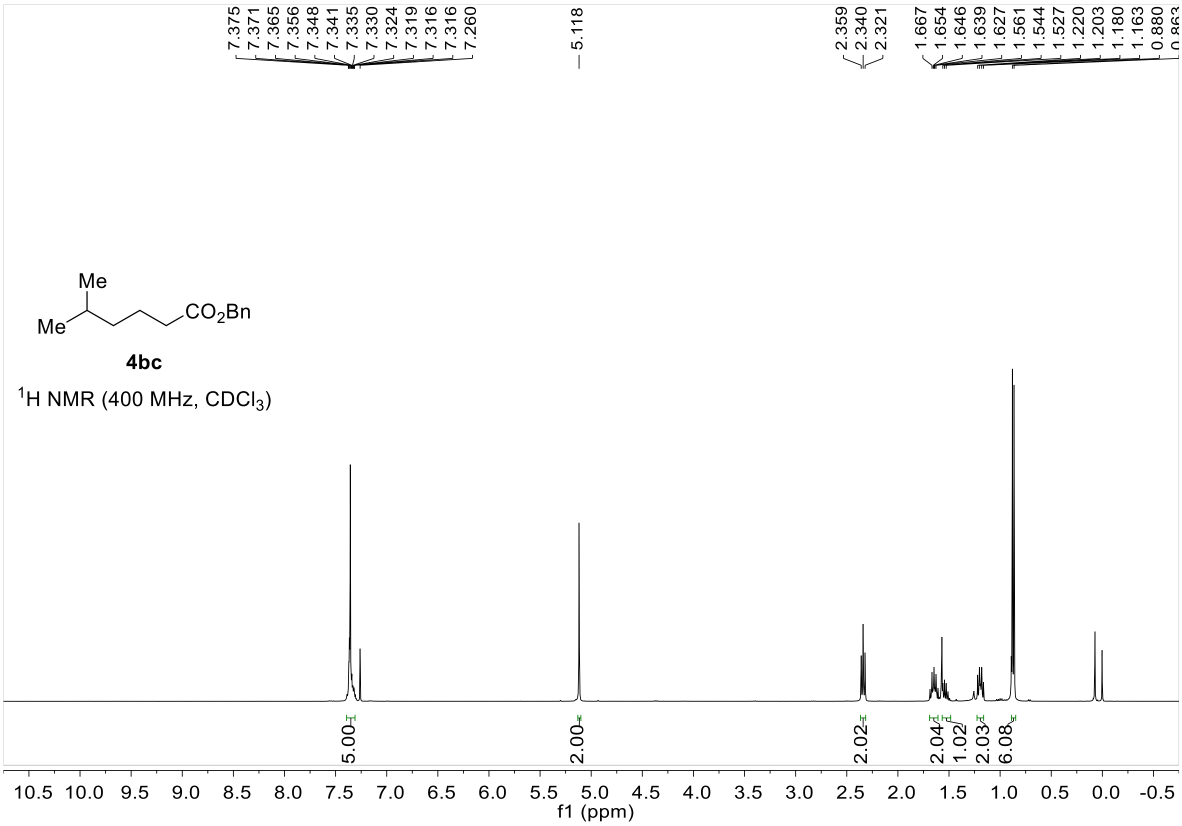

Supplementary Figure 43. ^{1}H NMR spectra of compound **4bc** (400 MHz, $CDCl_3$)

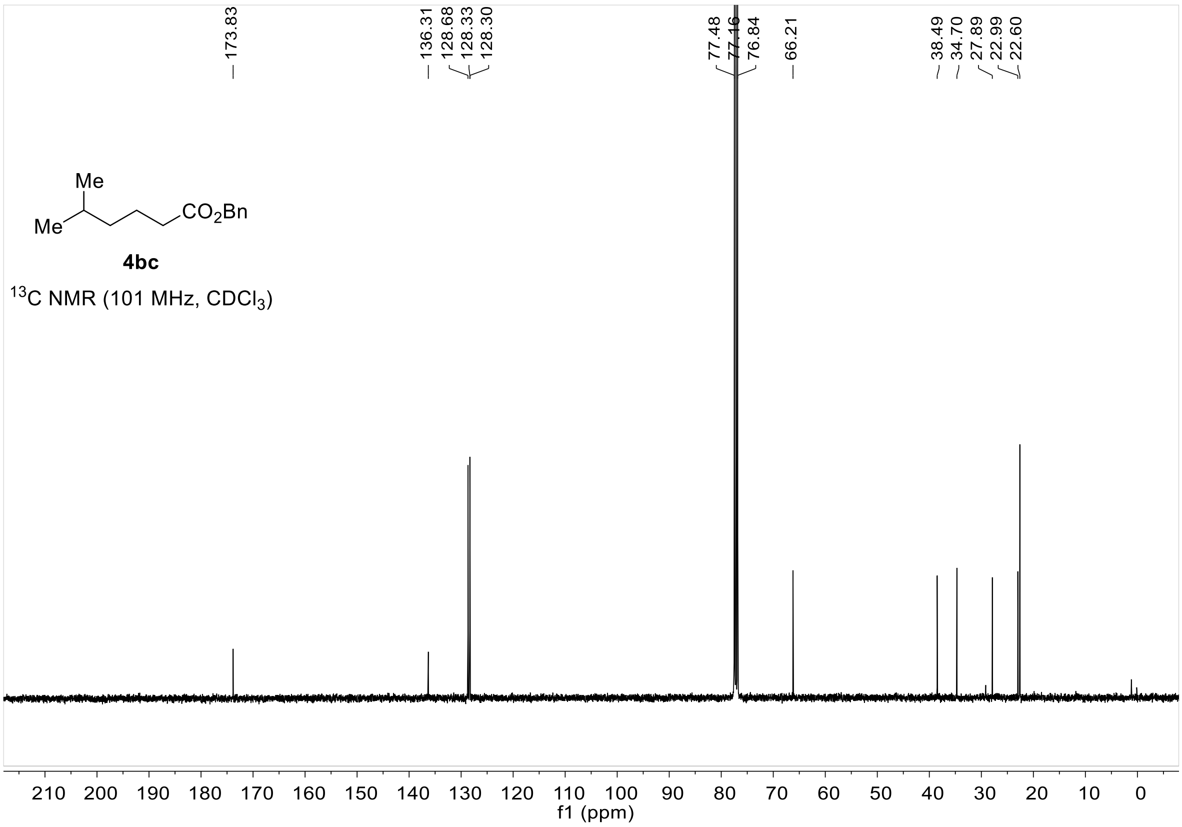

Supplementary Figure 44. ^{13}C NMR spectra of compound **4bc** (101 MHz, $CDCl_3$)

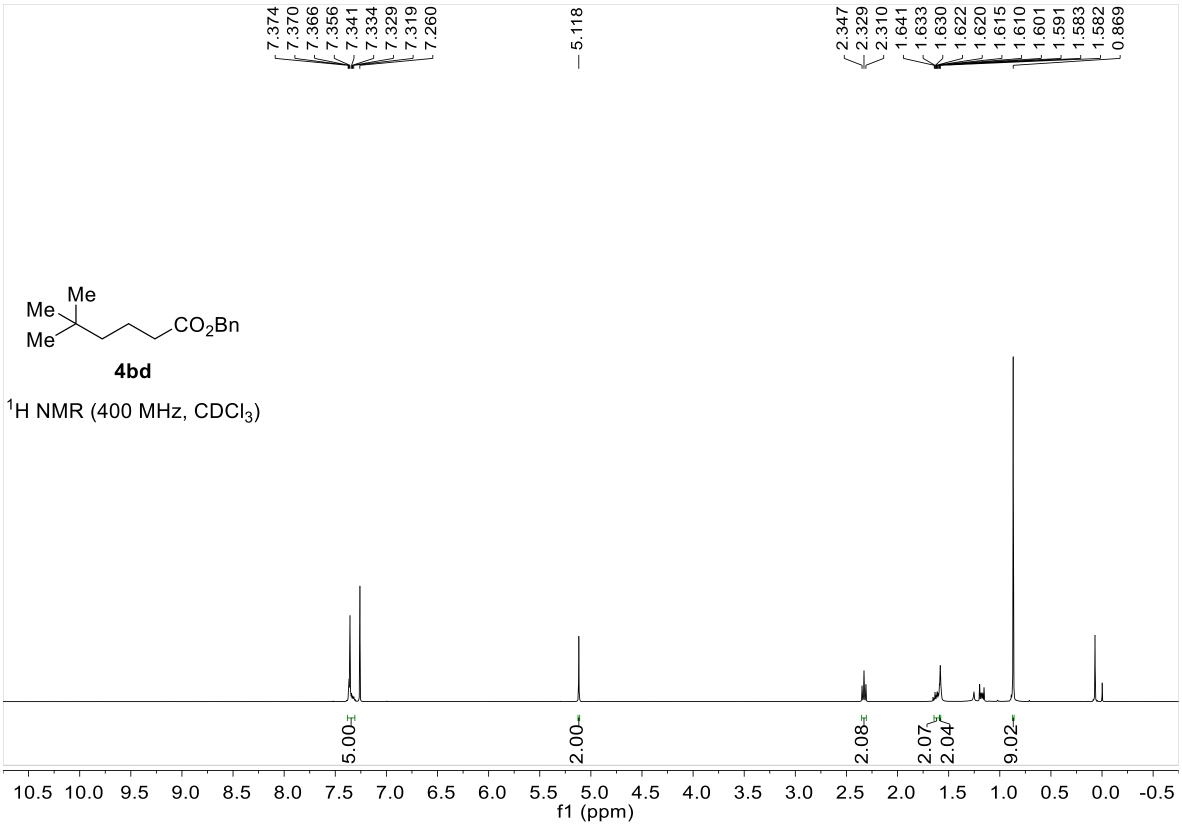

Supplementary Figure 45. ^{1}H NMR spectra of compound **4bd** (400 MHz, $CDCl_3$)

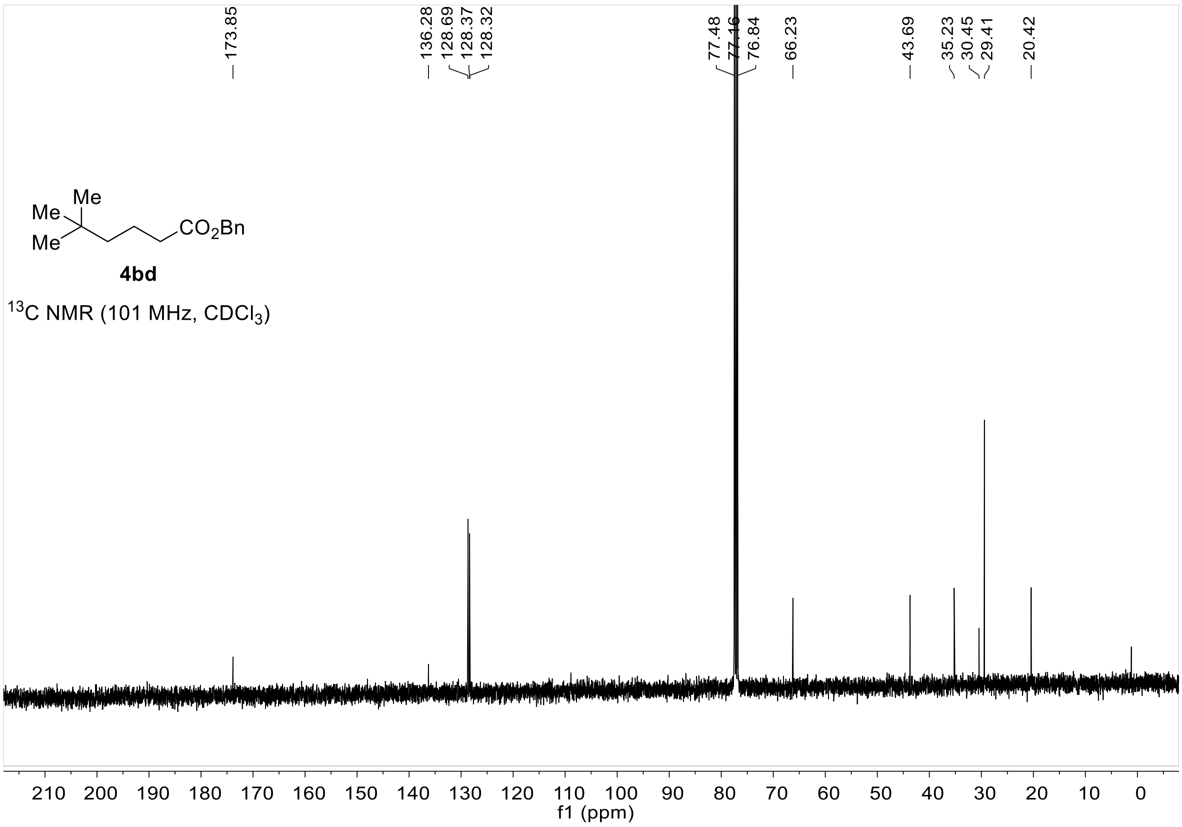

Supplementary Figure 46. ^{13}C NMR spectra of compound **4bd** (101 MHz, $CDCl_3$)

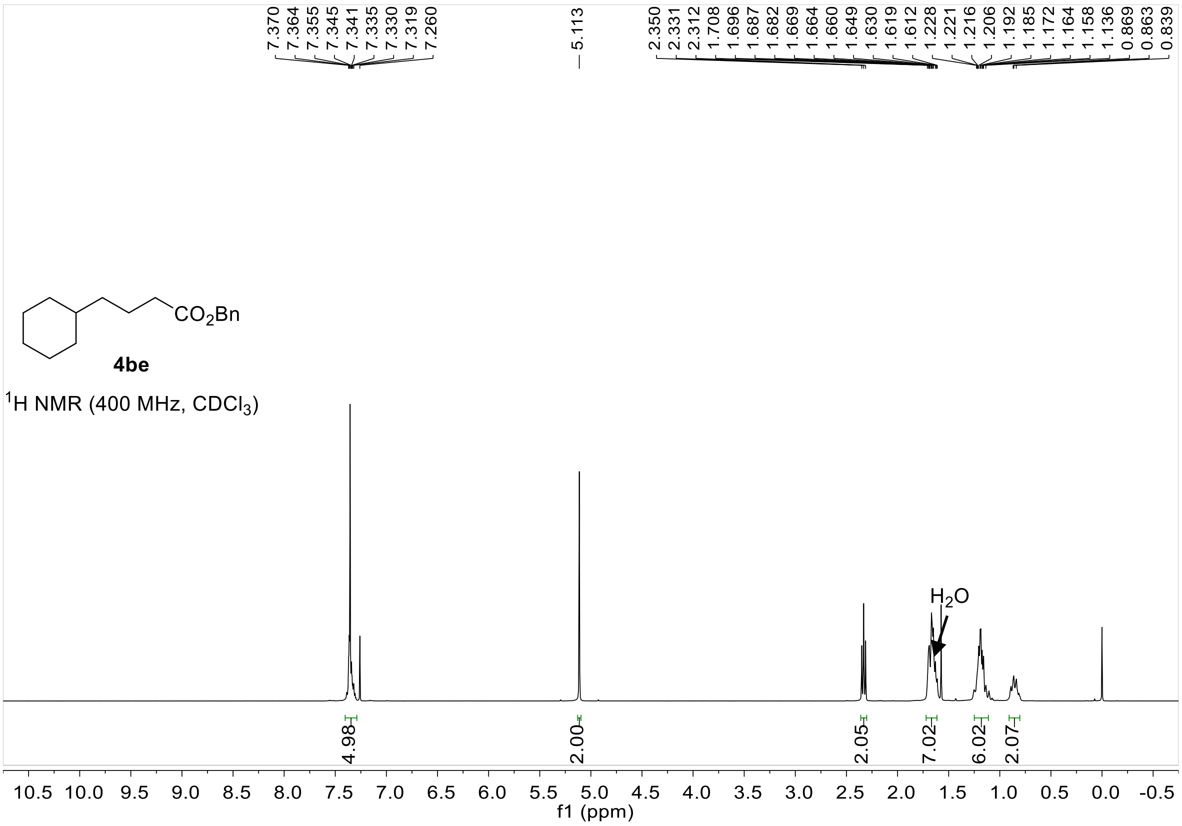

Supplementary Figure 47. ^{1}H NMR spectra of compound **4be** (400 MHz, $CDCl_3$)

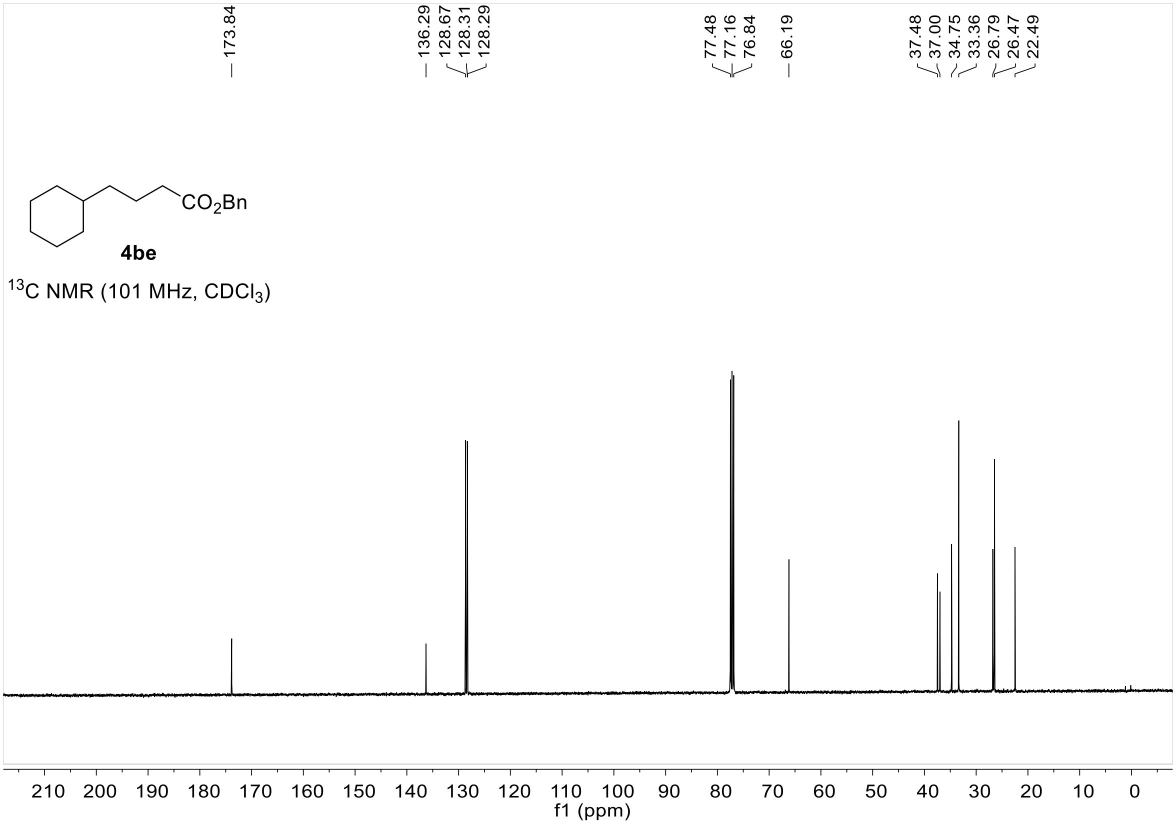

Supplementary Figure 48. ^{13}C NMR spectra of compound **4be** (101 MHz, $CDCl_3$)

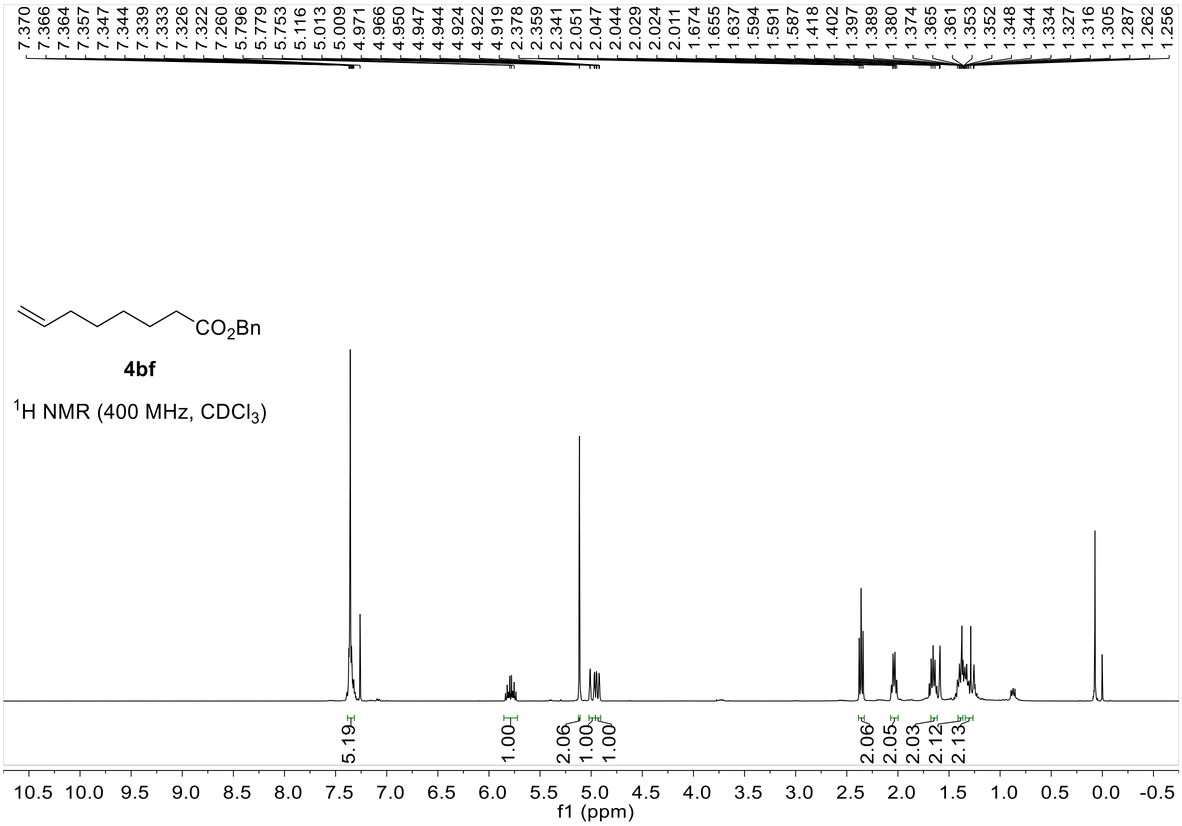

Supplementary Figure 49. ^{1}H NMR spectra of compound **4bf** (400 MHz, $CDCl_3$)

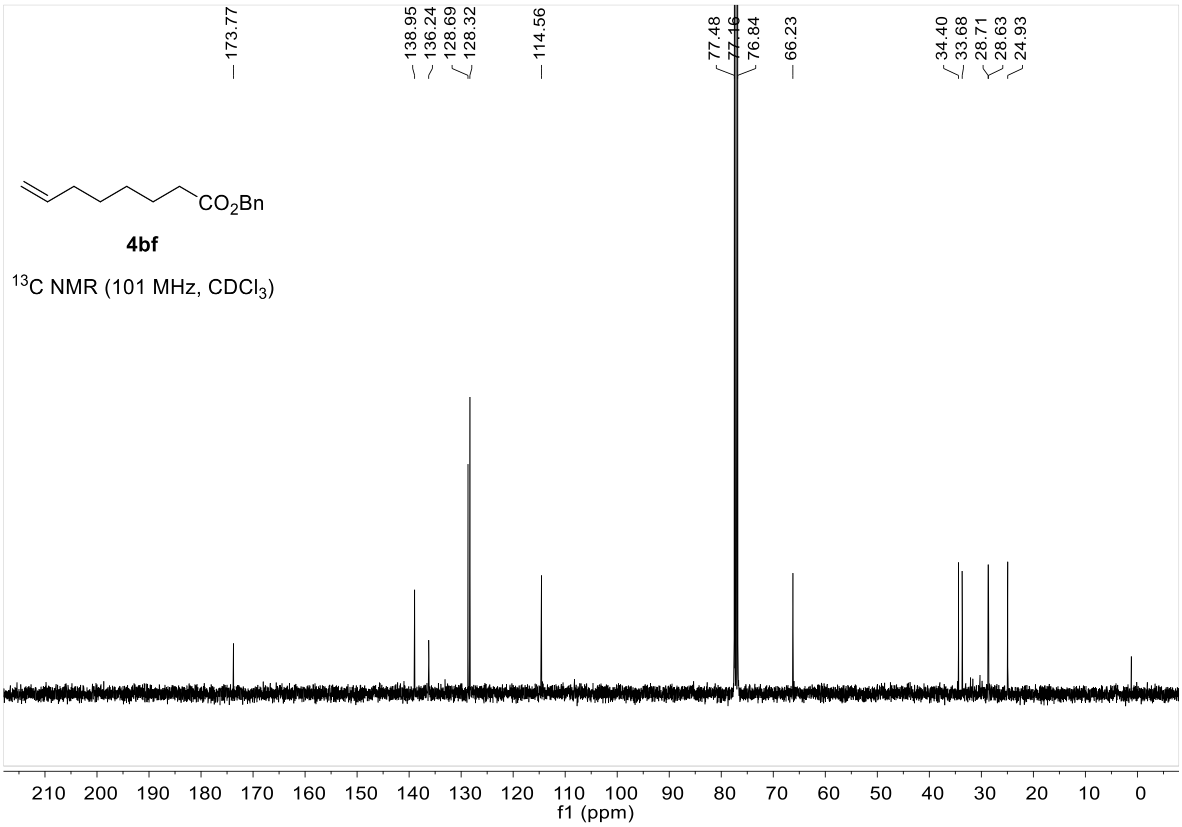

Supplementary Figure 50. ^{13}C NMR spectra of compound **4bf** (101 MHz, $CDCl_3$)

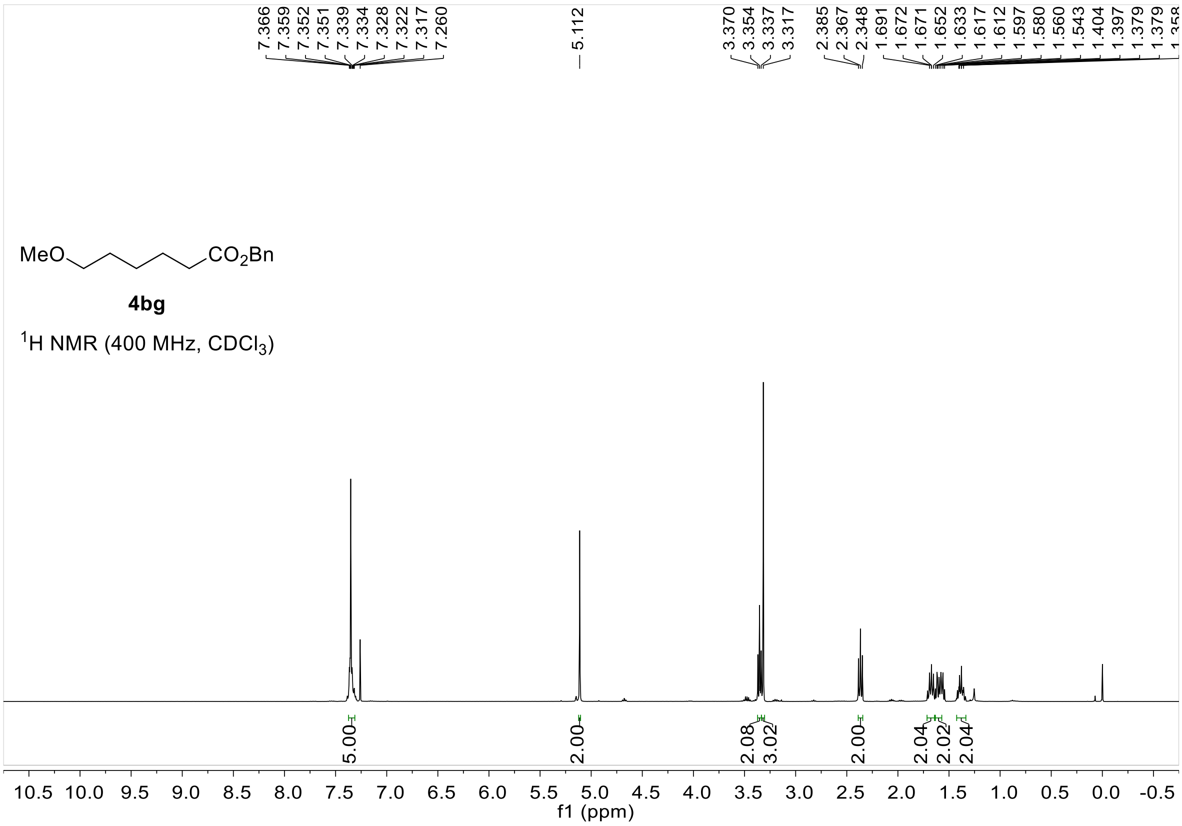

Supplementary Figure 51. ^{1}H NMR spectra of compound **4bg** (400 MHz, $CDCl_3$)

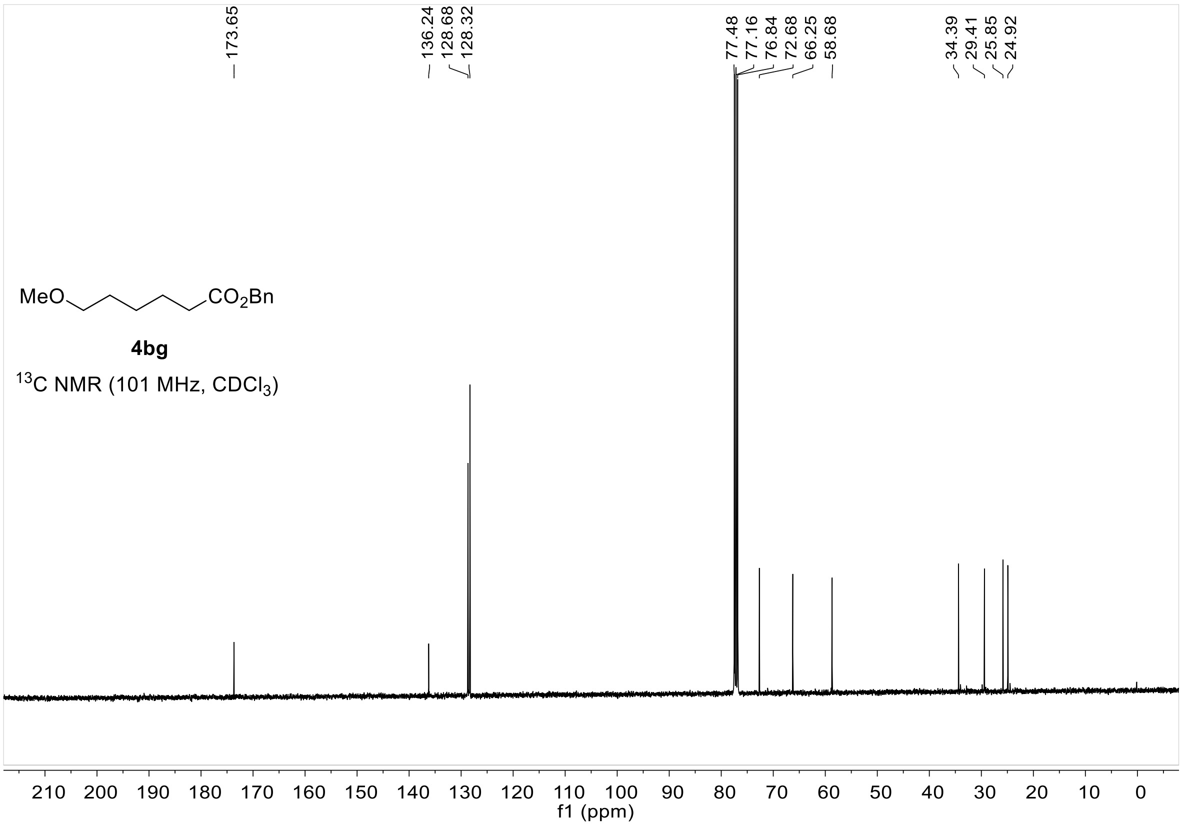

Supplementary Figure 52. ^{13}C NMR spectra of compound **4bg** (101 MHz, $CDCl_3$)

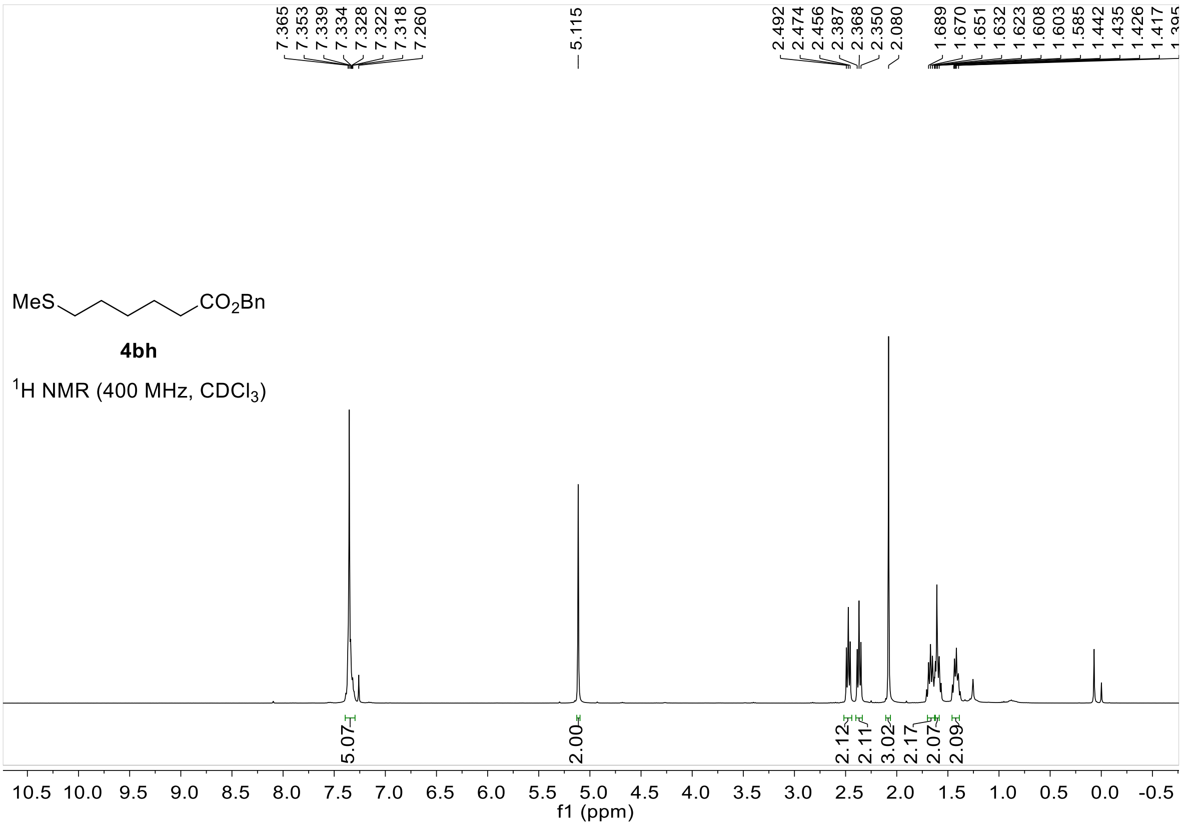

Supplementary Figure 53. ^{1}H NMR spectra of compound **4bh** (400 MHz, $CDCl_3$)

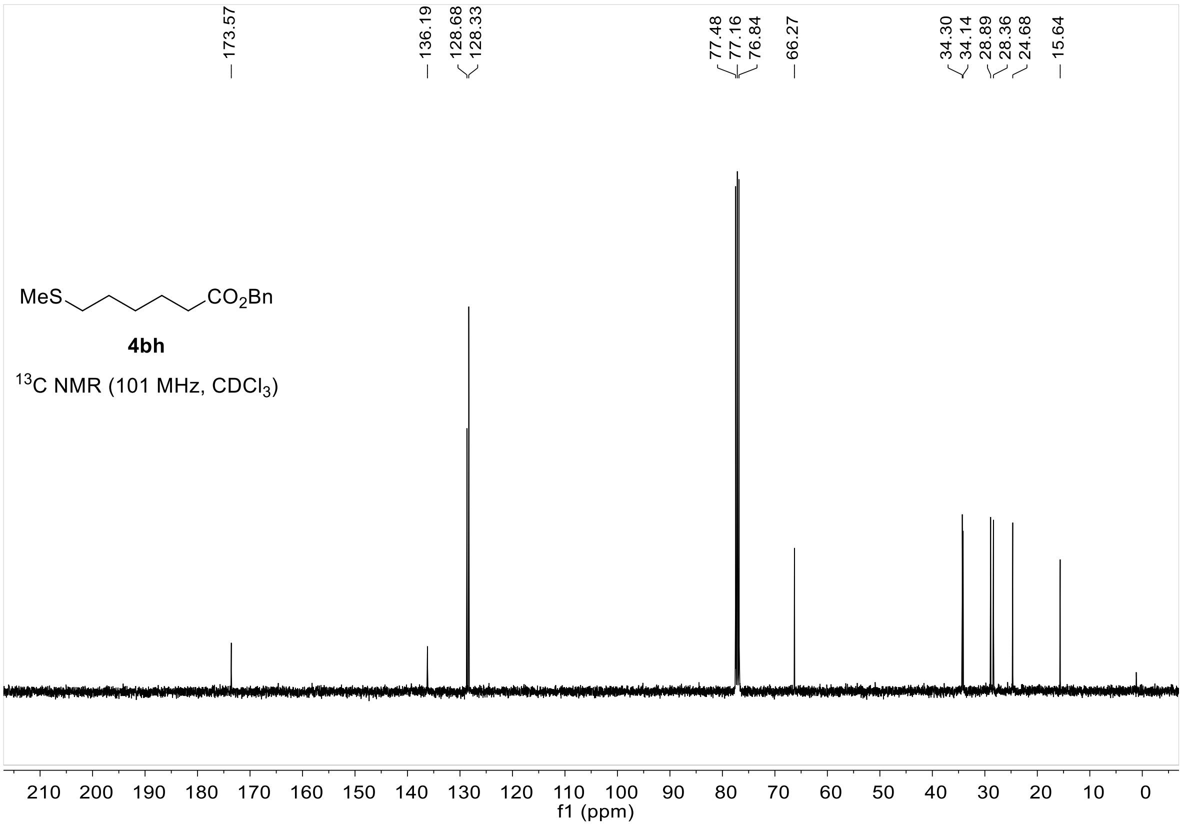

Supplementary Figure 54. ^{13}C NMR spectra of compound **4bh** (101 MHz, $CDCl_3$)

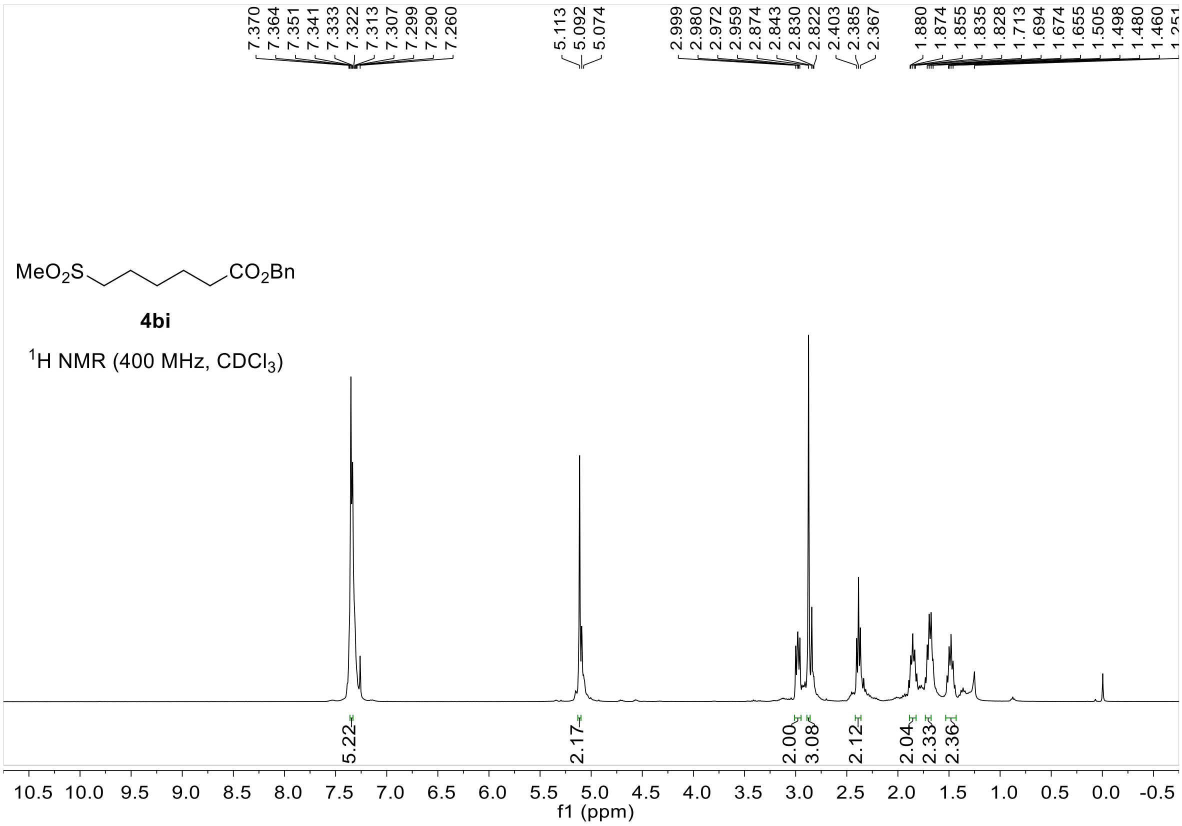

Supplementary Figure 55. ^{1}H NMR spectra of compound **4bi** (400 MHz, $CDCl_3$)

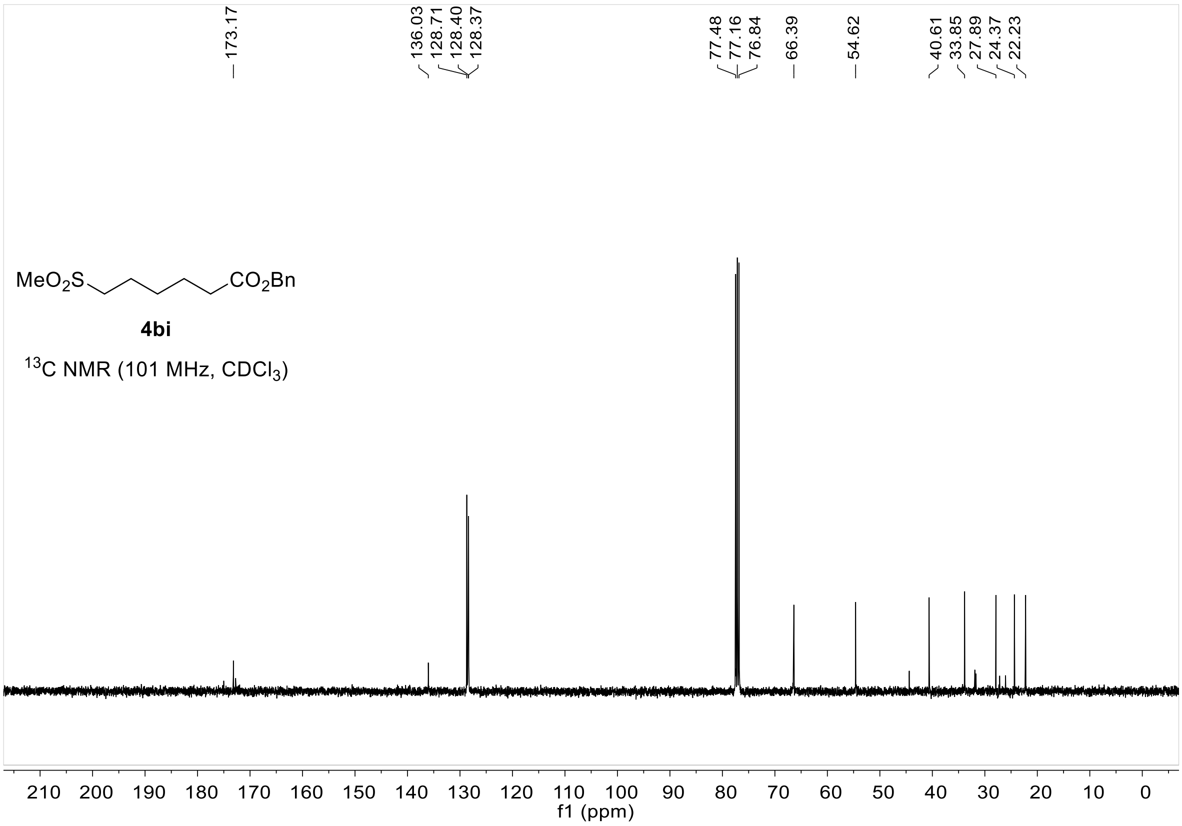

Supplementary Figure 56. ^{13}C NMR spectra of compound **4bi** (101 MHz, $CDCl_3$)

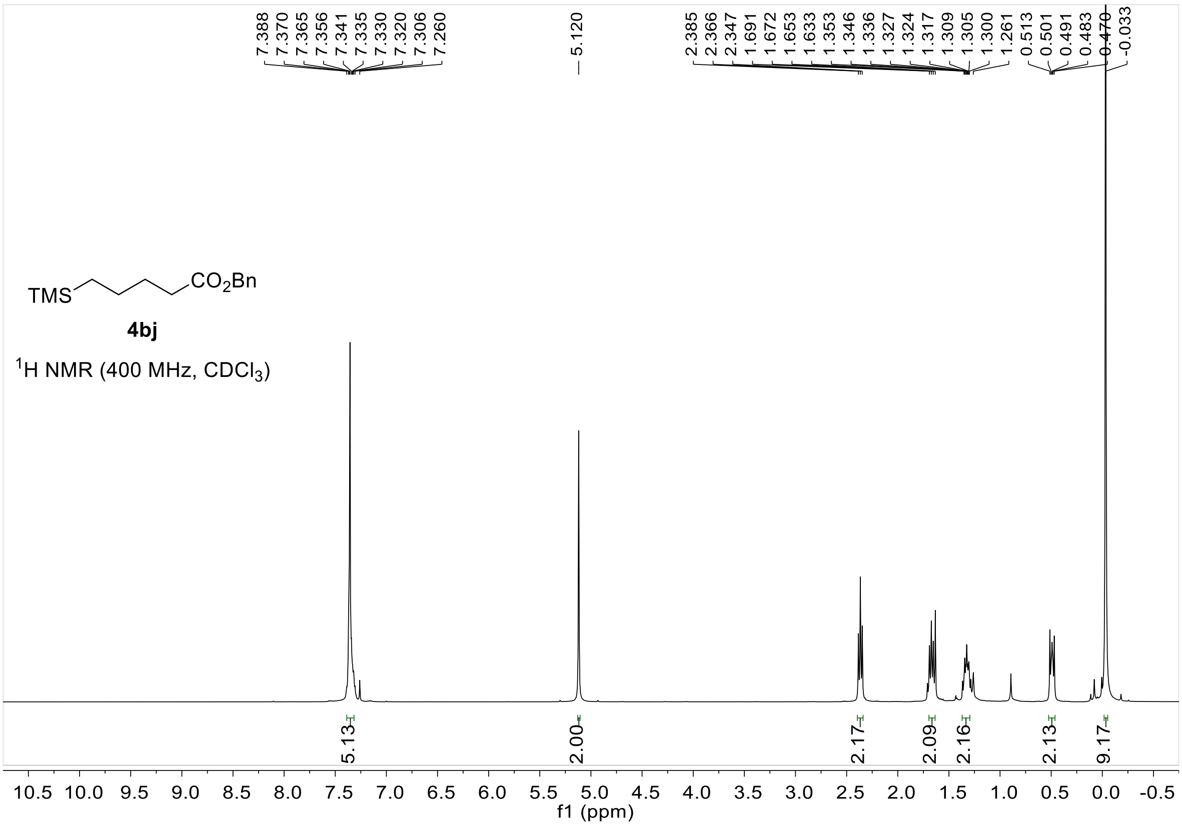

Supplementary Figure 57. ^{1}H NMR spectra of compound **4bj** (400 MHz, $CDCl_3$)

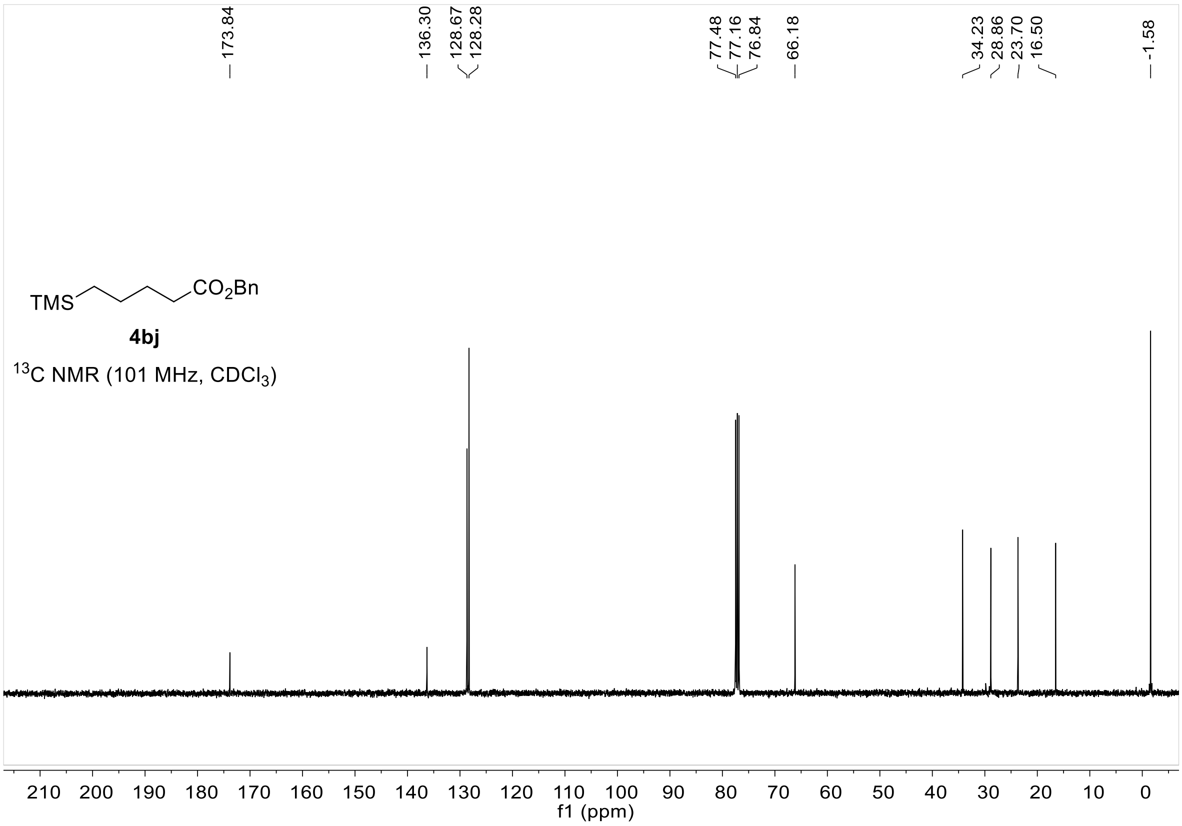

Supplementary Figure 58. ^{13}C NMR spectra of compound **4bj** (101 MHz, $CDCl_3$)

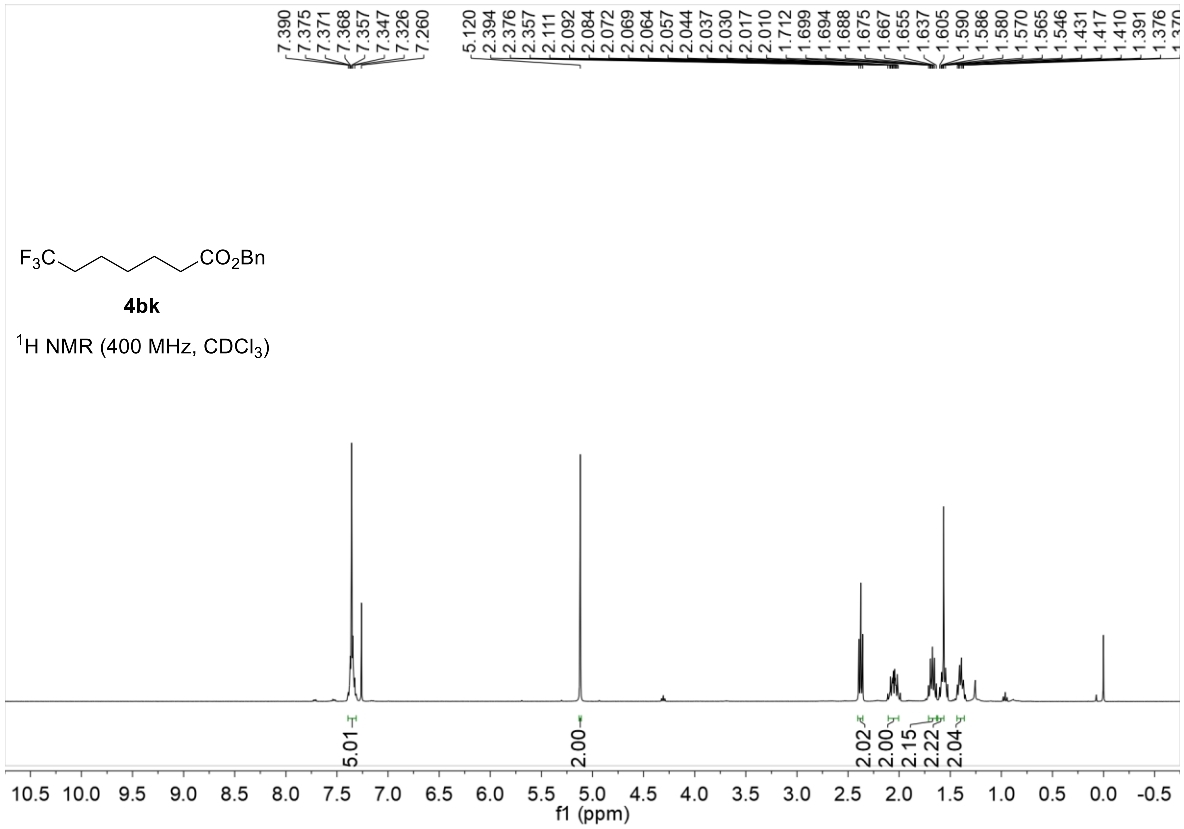

Supplementary Figure 59. ^{1}H NMR spectra of compound **4bk** (400 MHz, $CDCl_3$)

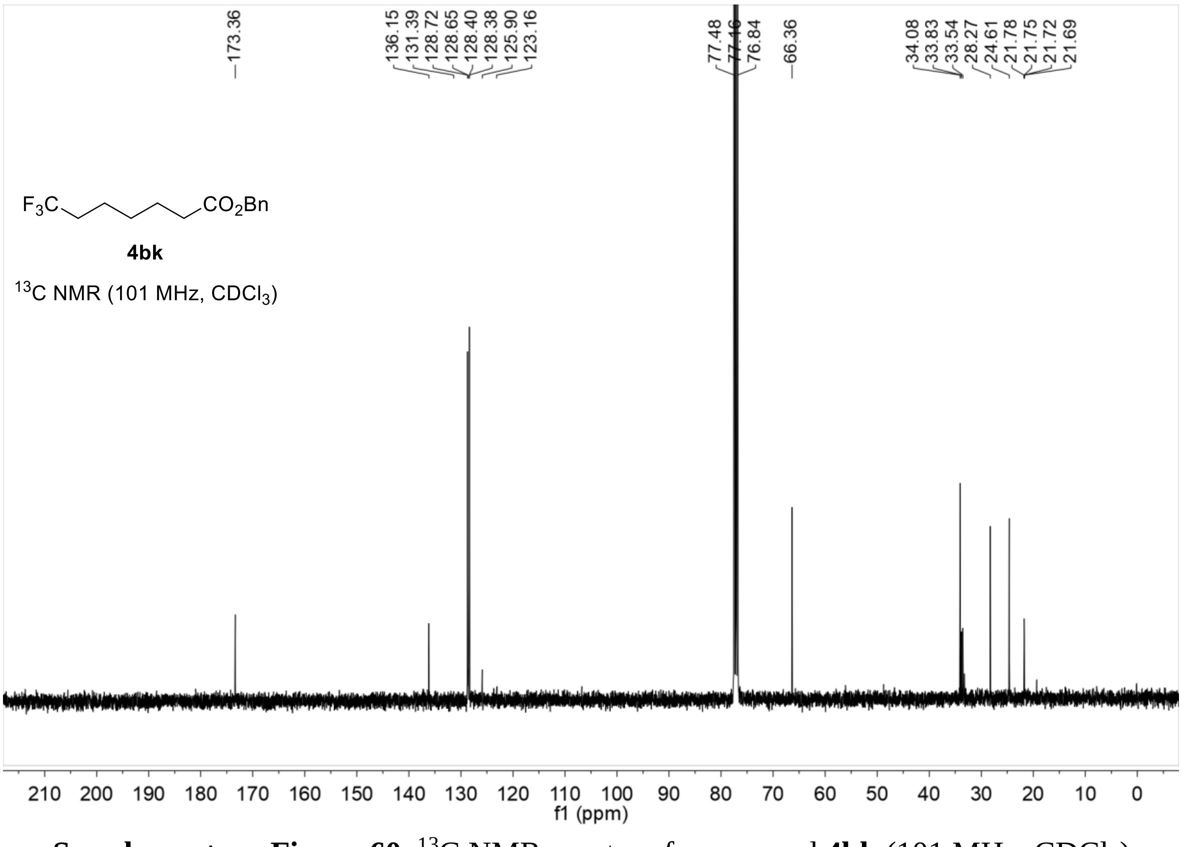

Supplementary Figure 60. ^{13}C NMR spectra of compound **4bk** (101 MHz, $CDCl_3$)

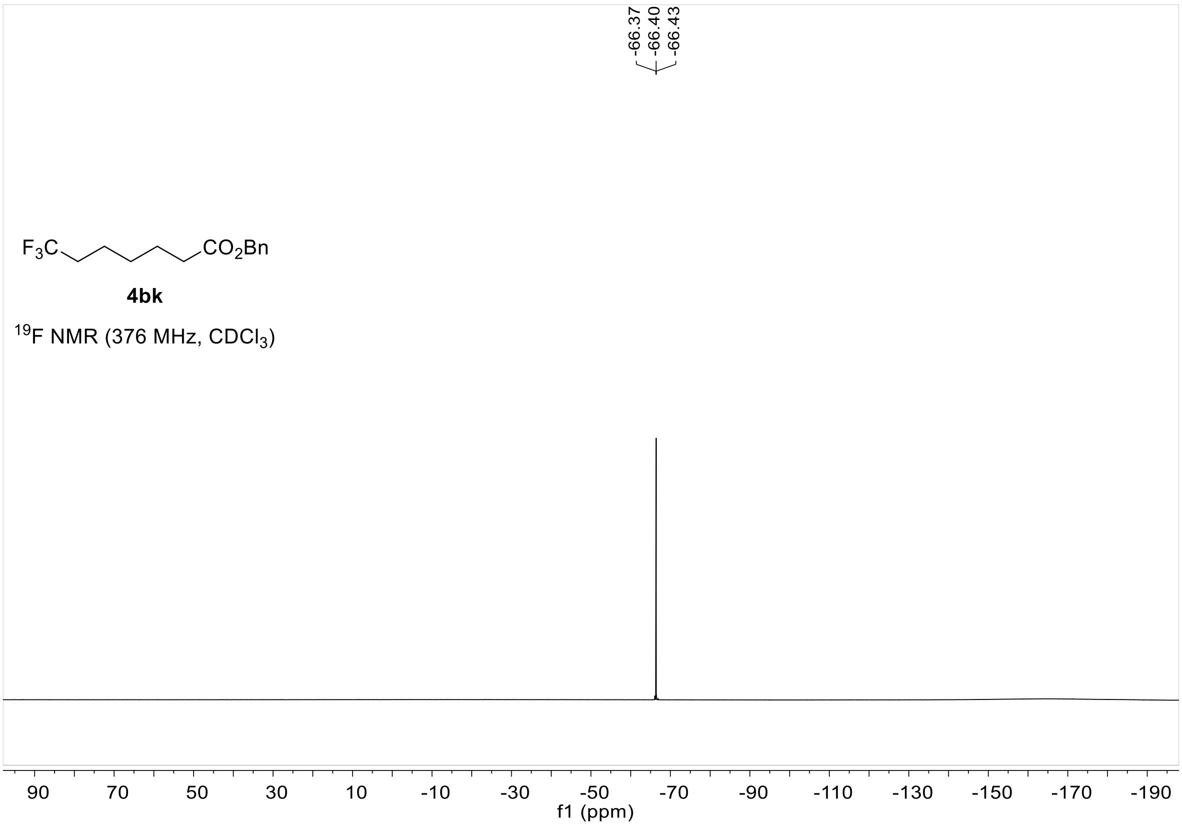

Supplementary Figure 61. ^{19}F NMR spectra of compound **4bk** (376 MHz, $CDCl_3$)

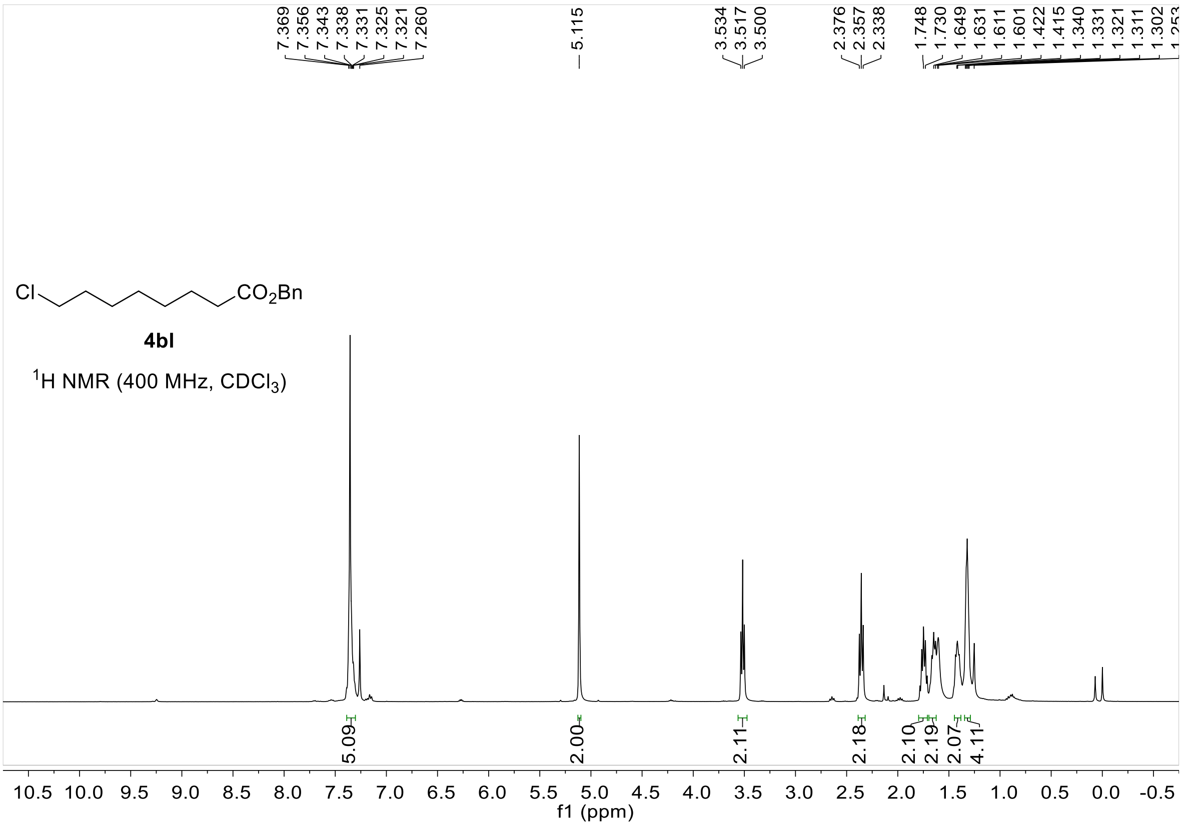

Supplementary Figure 62. ^{1}H NMR spectra of compound **4bl** (400 MHz, $CDCl_3$)

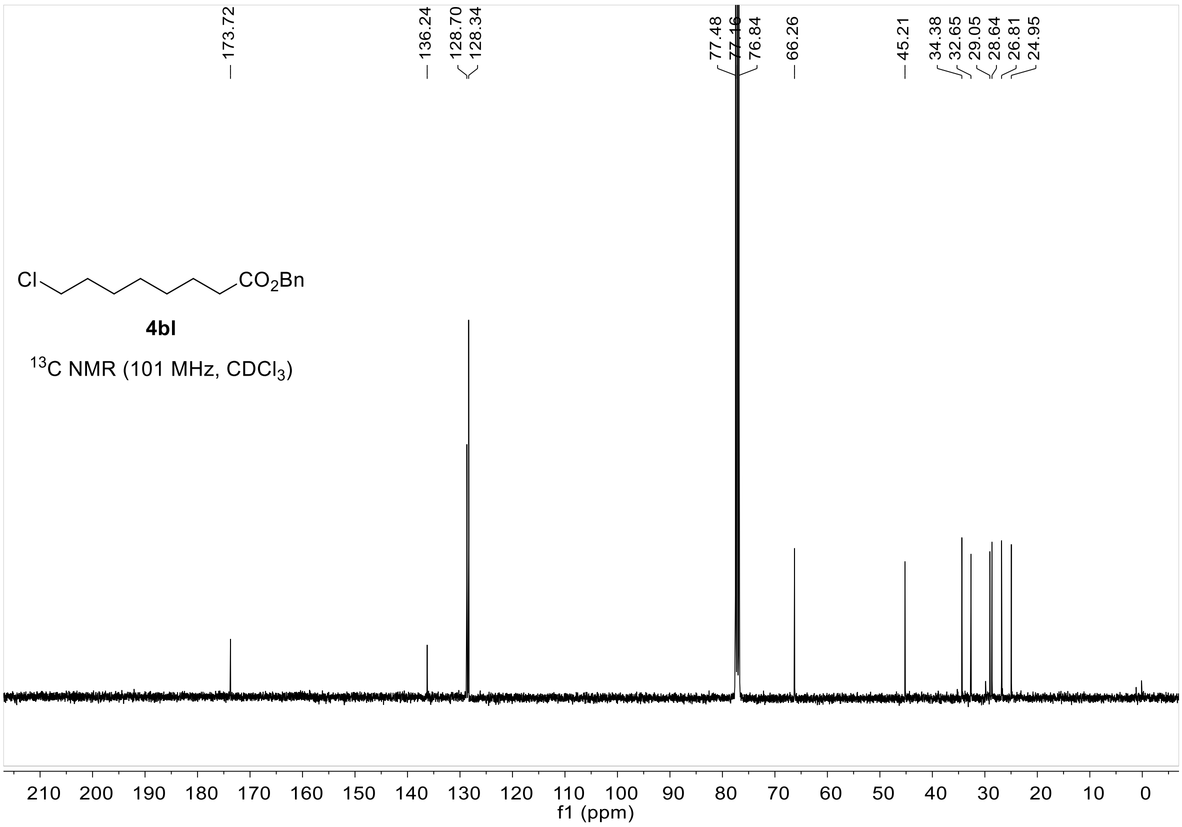

Supplementary Figure 63. ^{13}C NMR spectra of compound **4bl** (101 MHz, $CDCl_3$)

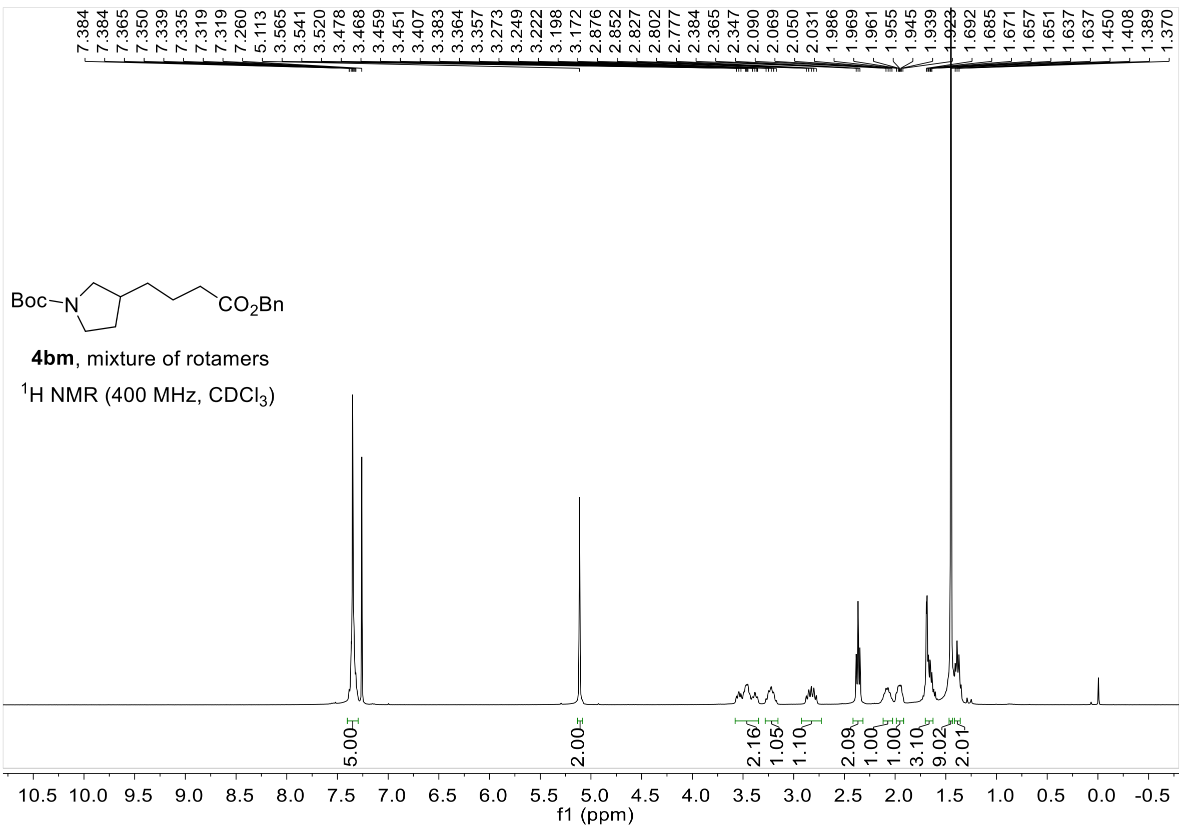

Supplementary Figure 64. ^{1}H NMR spectra of compound **4bm** (400 MHz, $CDCl_3$)

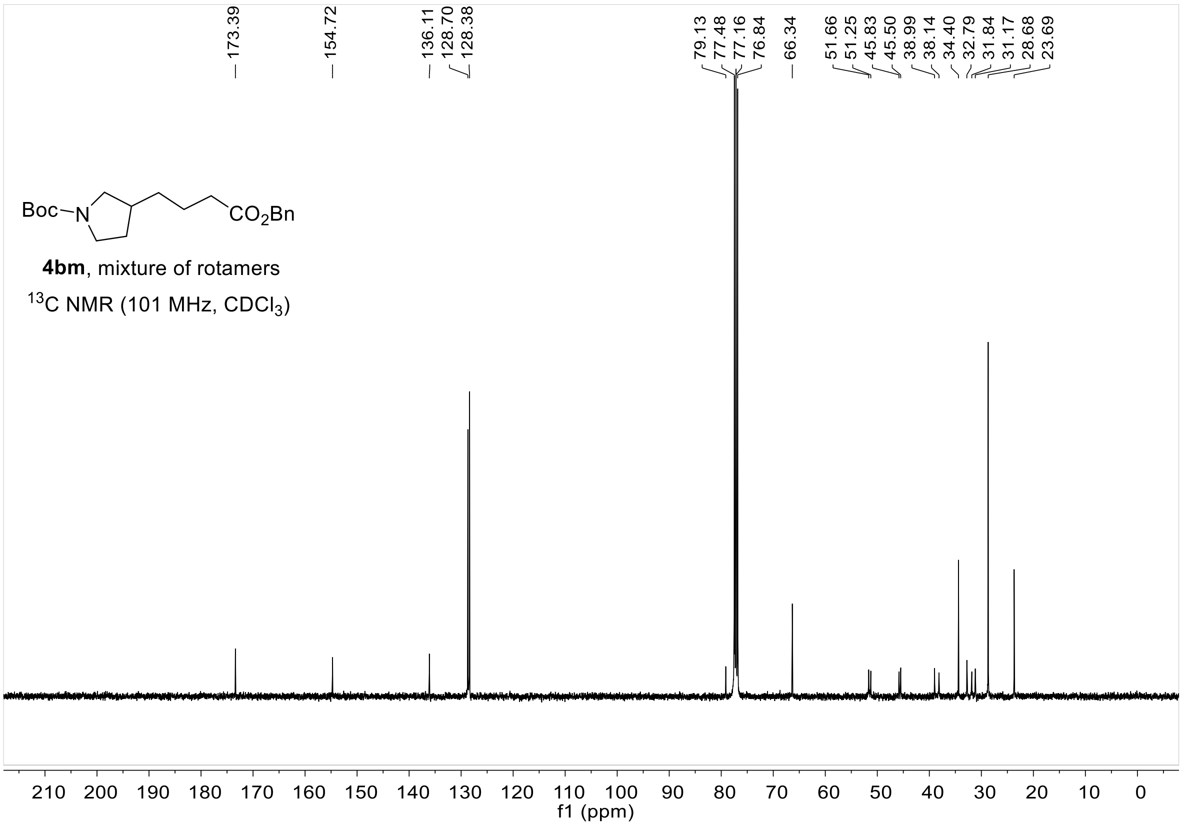

Supplementary Figure 65. ^{13}C NMR spectra of compound **4bm** (101 MHz, $CDCl_3$)

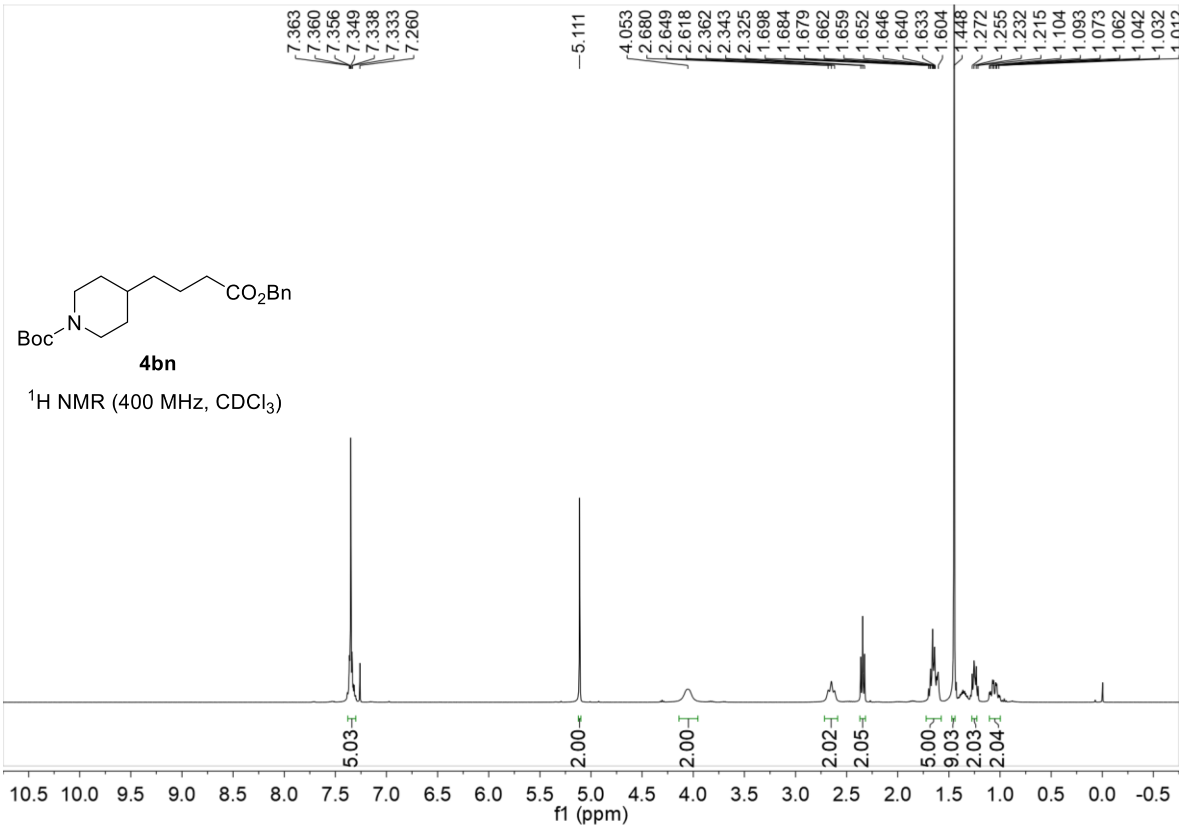

Supplementary Figure 66. ^{1}H NMR spectra of compound **4bn** (400 MHz, $CDCl_3$)

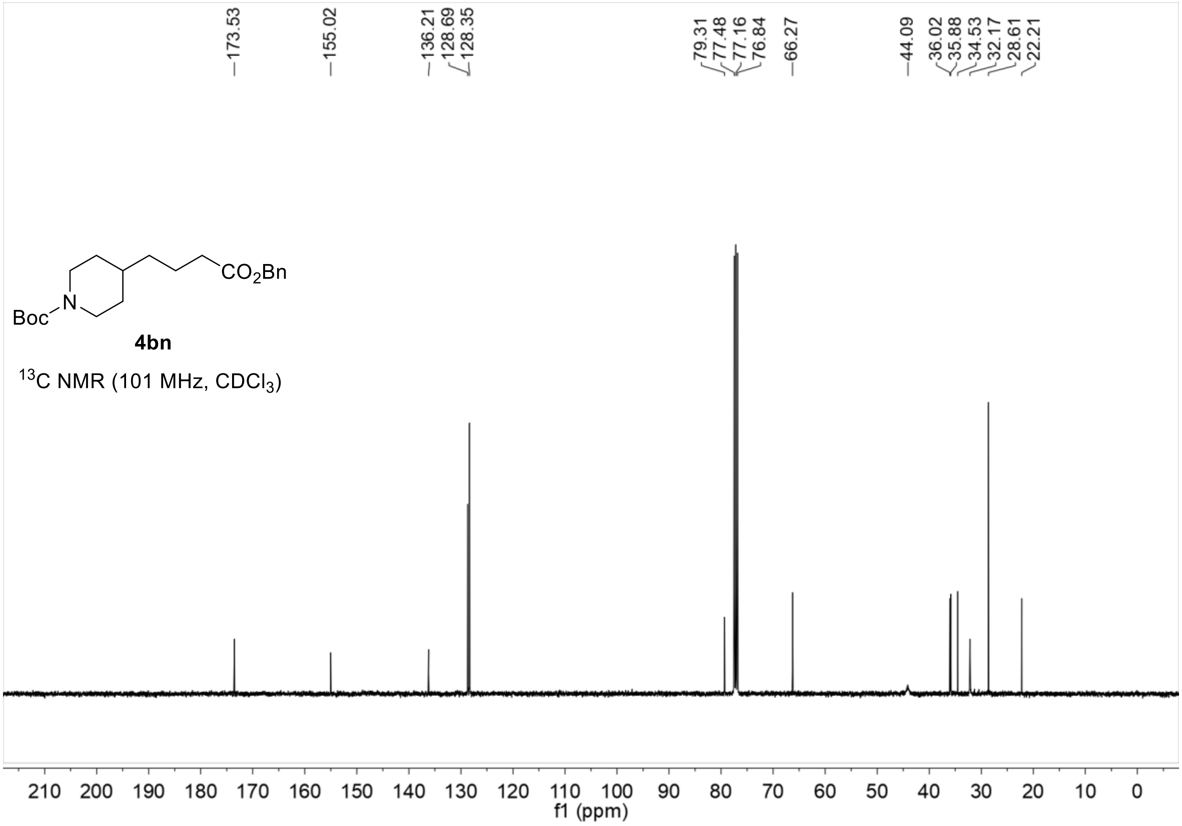

Supplementary Figure 67. ^{13}C NMR spectra of compound **4bn** (101 MHz, $CDCl_3$)

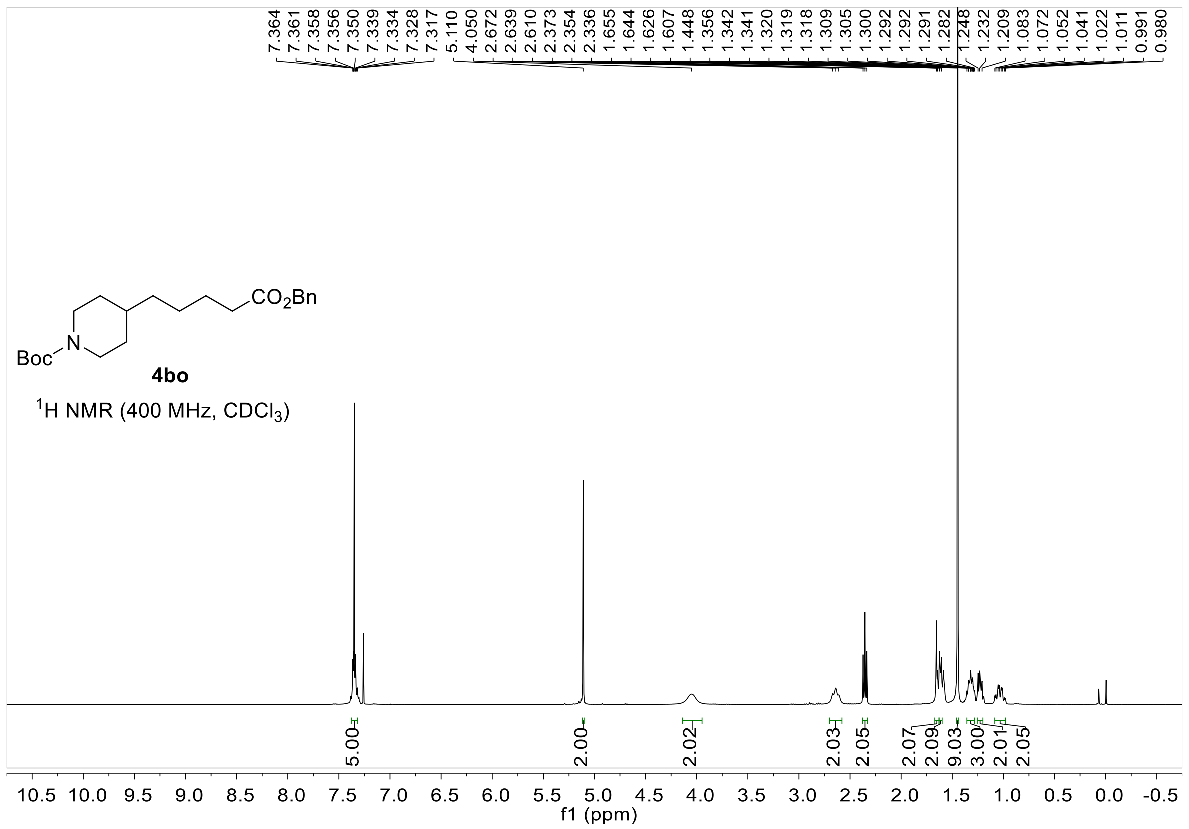

Supplementary Figure 68. ^{1}H NMR spectra of compound **4bo** (400 MHz, $CDCl_3$)

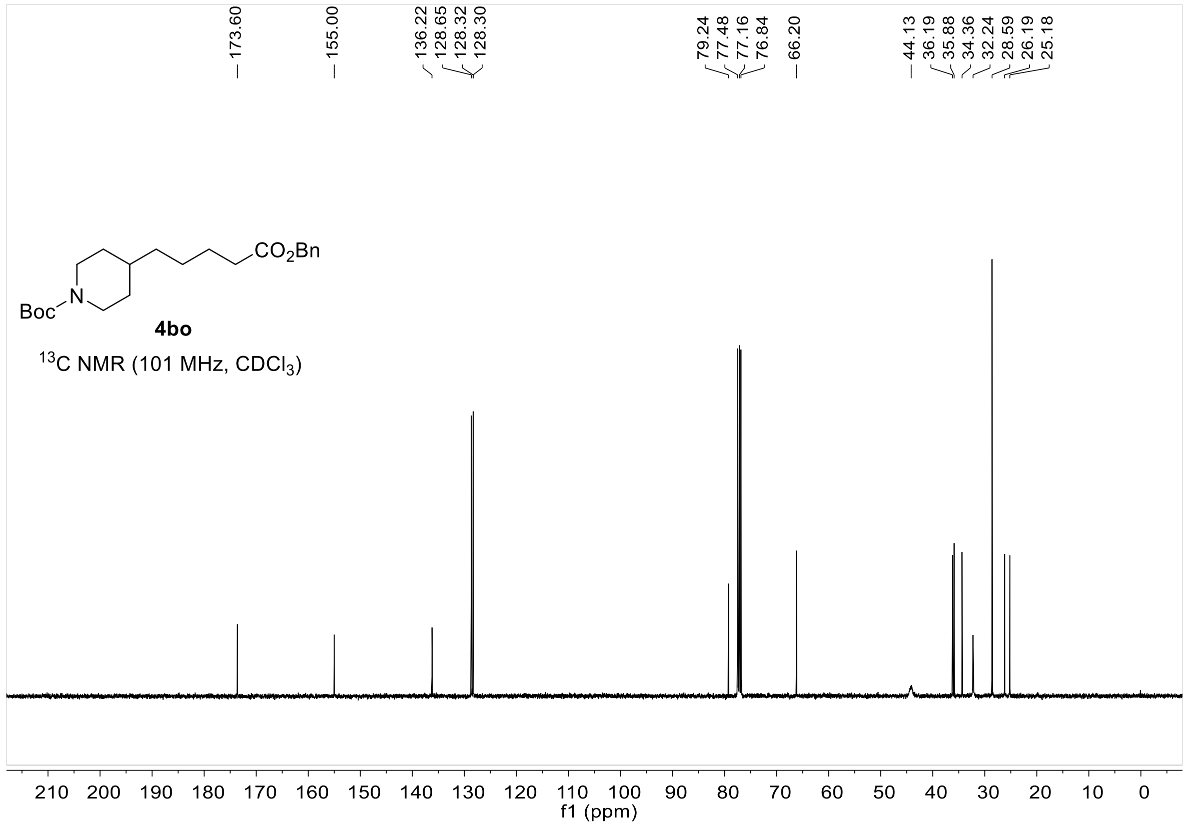

Supplementary Figure 69. ^{13}C NMR spectra of compound **4bo** (101 MHz, $CDCl_3$)

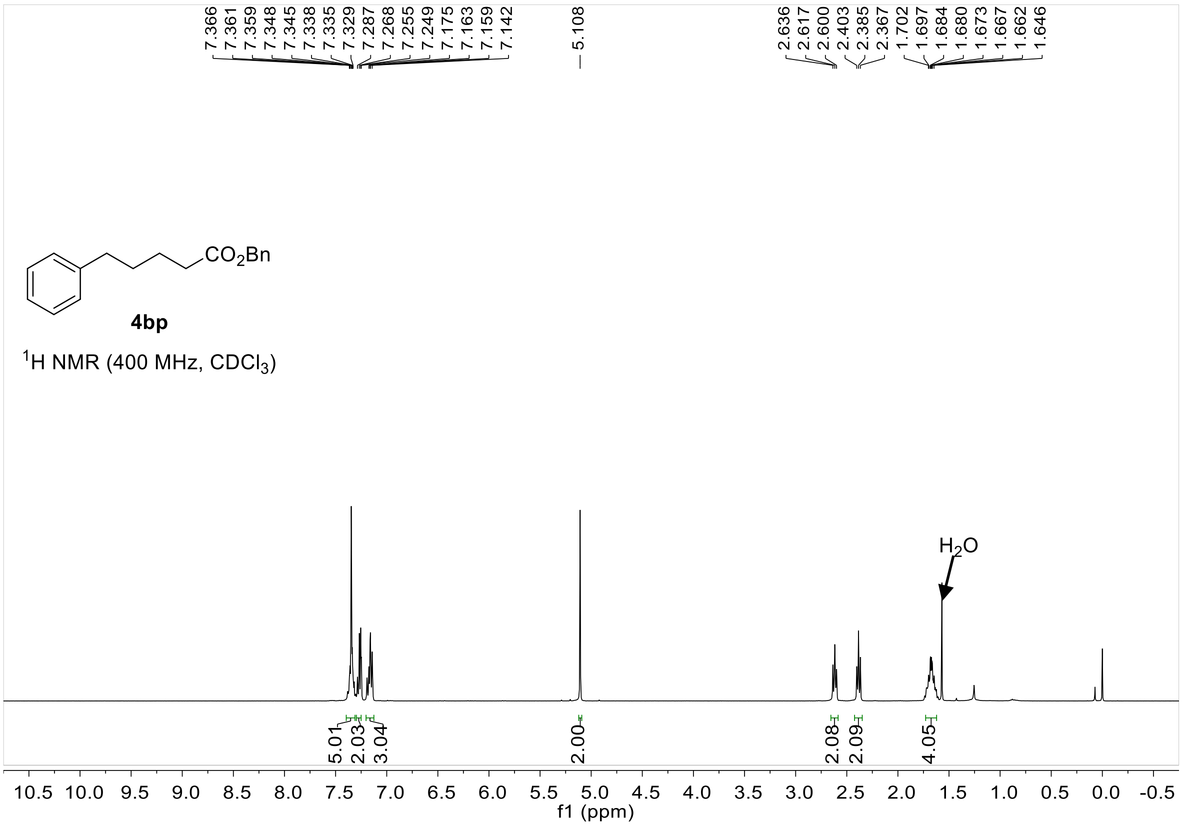

Supplementary Figure 70. ^{1}H NMR spectra of compound **4bp** (400 MHz, $CDCl_3$)

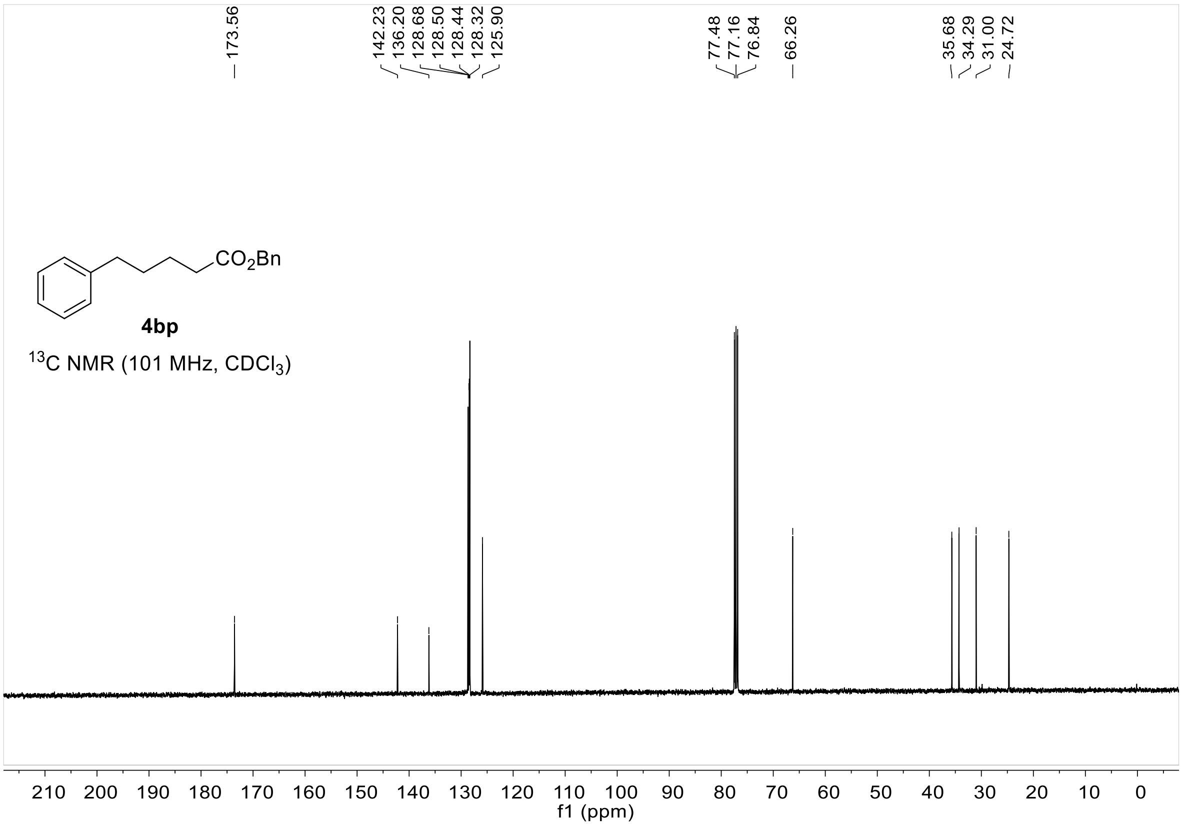

Supplementary Figure 71. ^{13}C NMR spectra of compound **4bp** (101 MHz, $CDCl_3$)

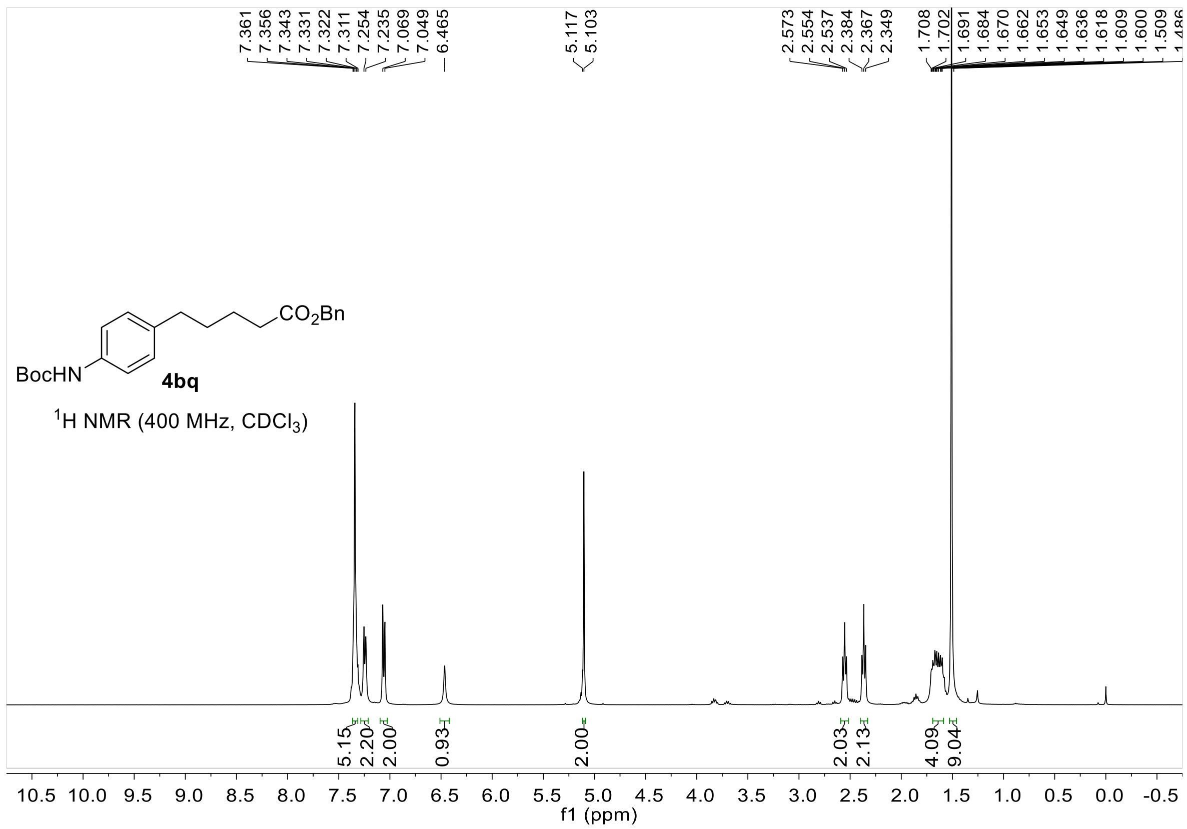

Supplementary Figure 72. ^{1}H NMR spectra of compound **4bq** (400 MHz, $CDCl_3$)

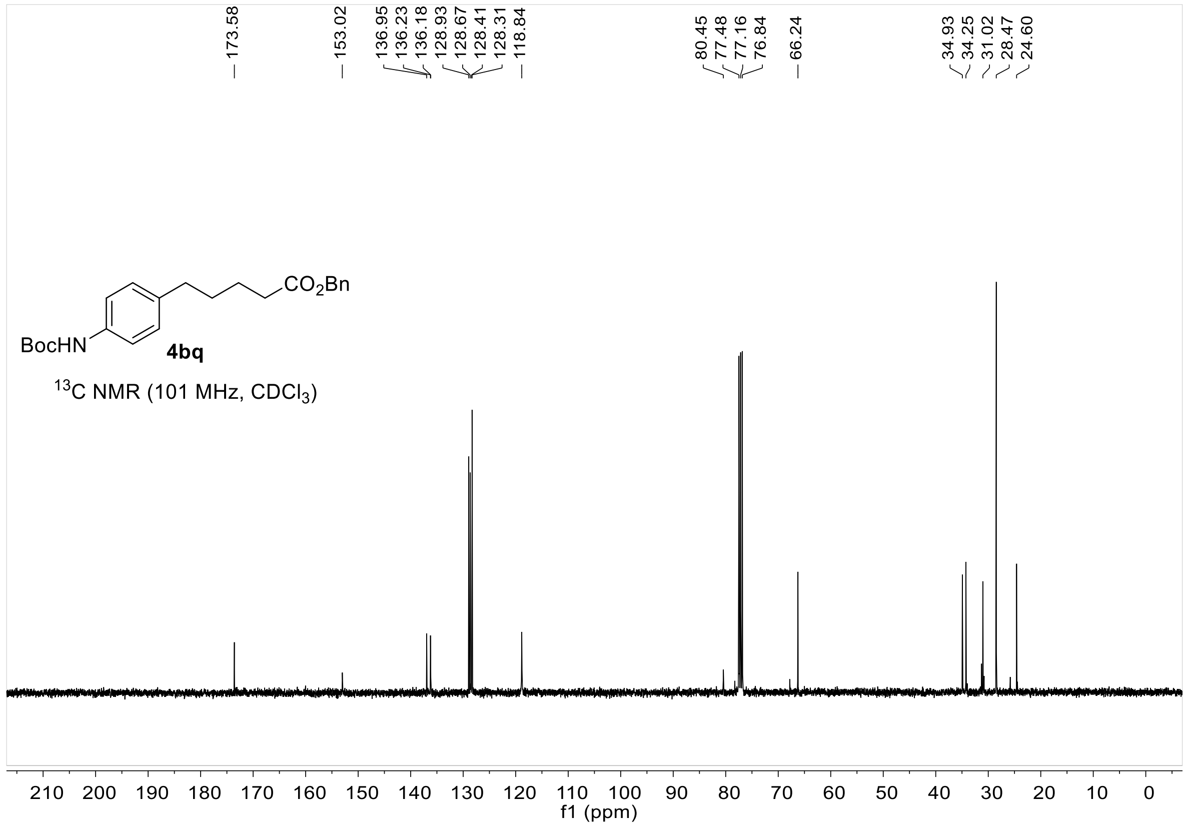

Supplementary Figure 73. ^{13}C NMR spectra of compound **4bq** (101 MHz, $CDCl_3$)

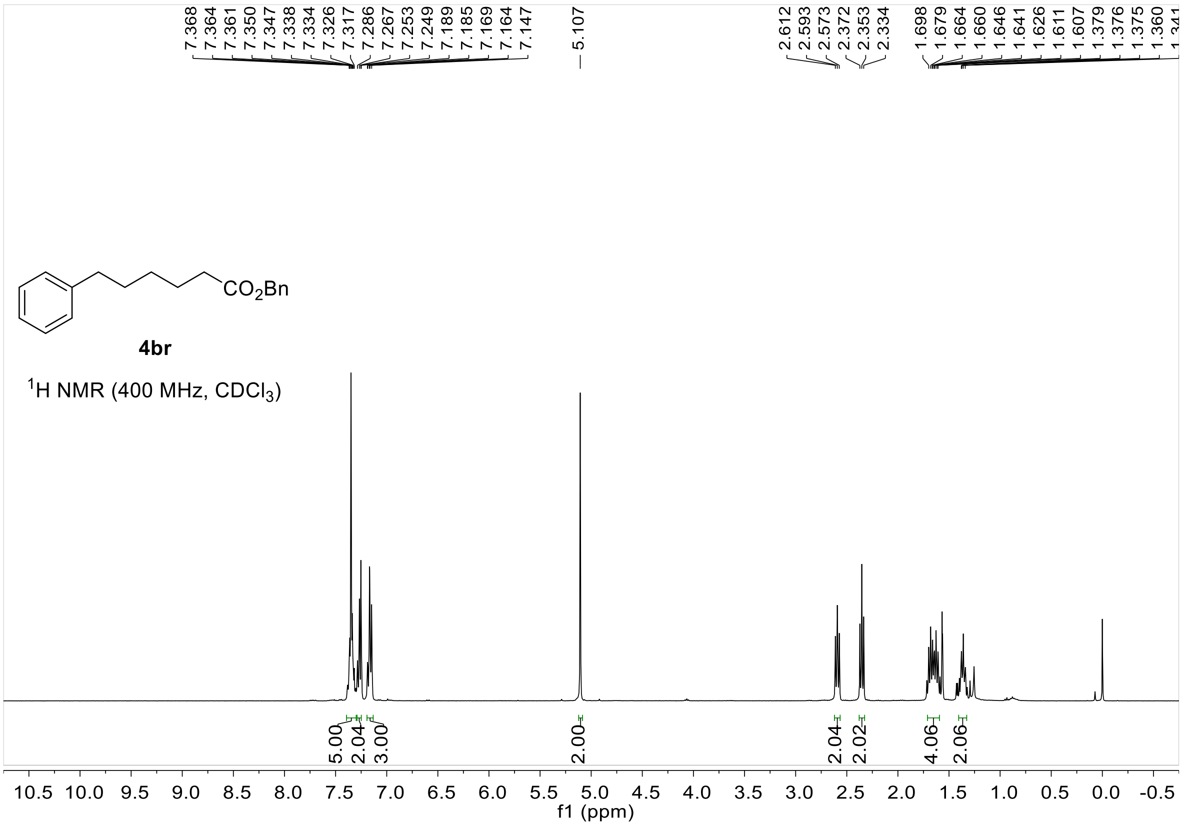

Supplementary Figure 74. ^{1}H NMR spectra of compound **4br** (400 MHz, $CDCl_3$)

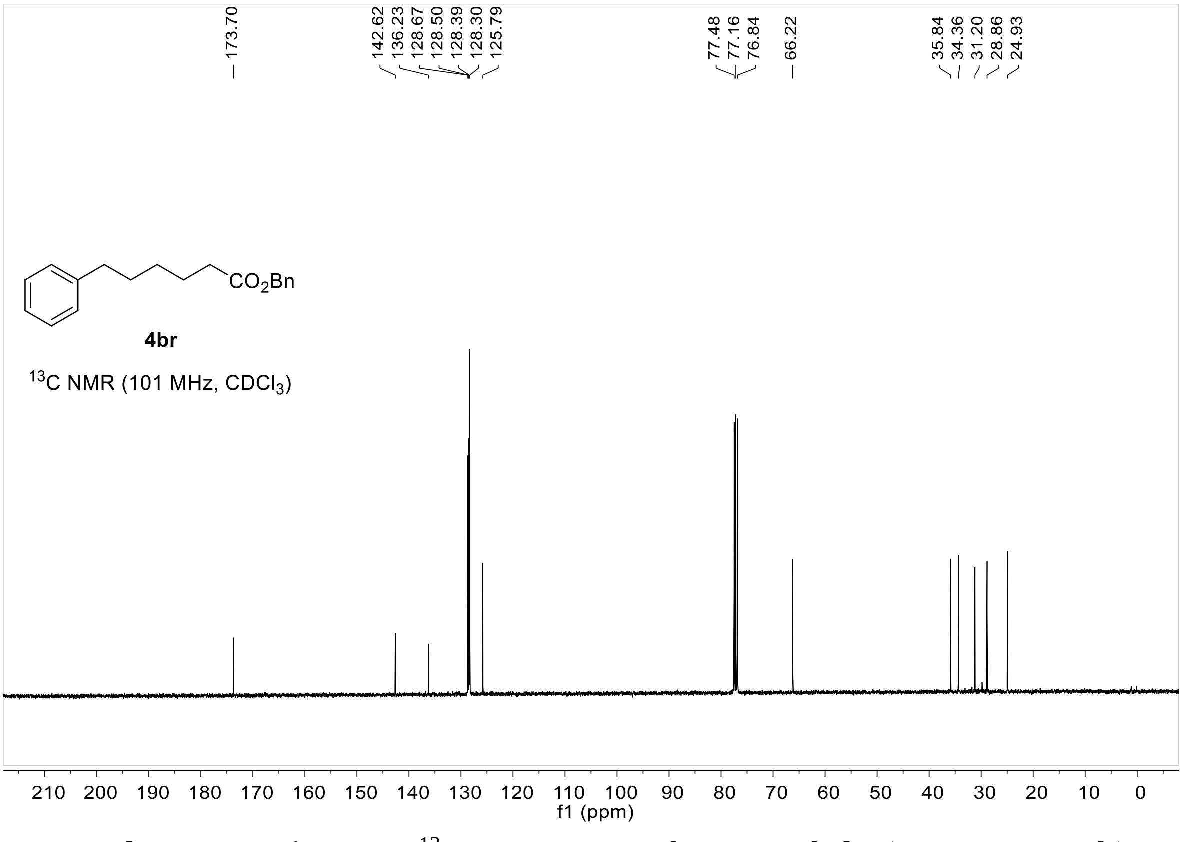

Supplementary Figure 75. ^{13}C NMR spectra of compound **4br** (101 MHz, $CDCl_3$)

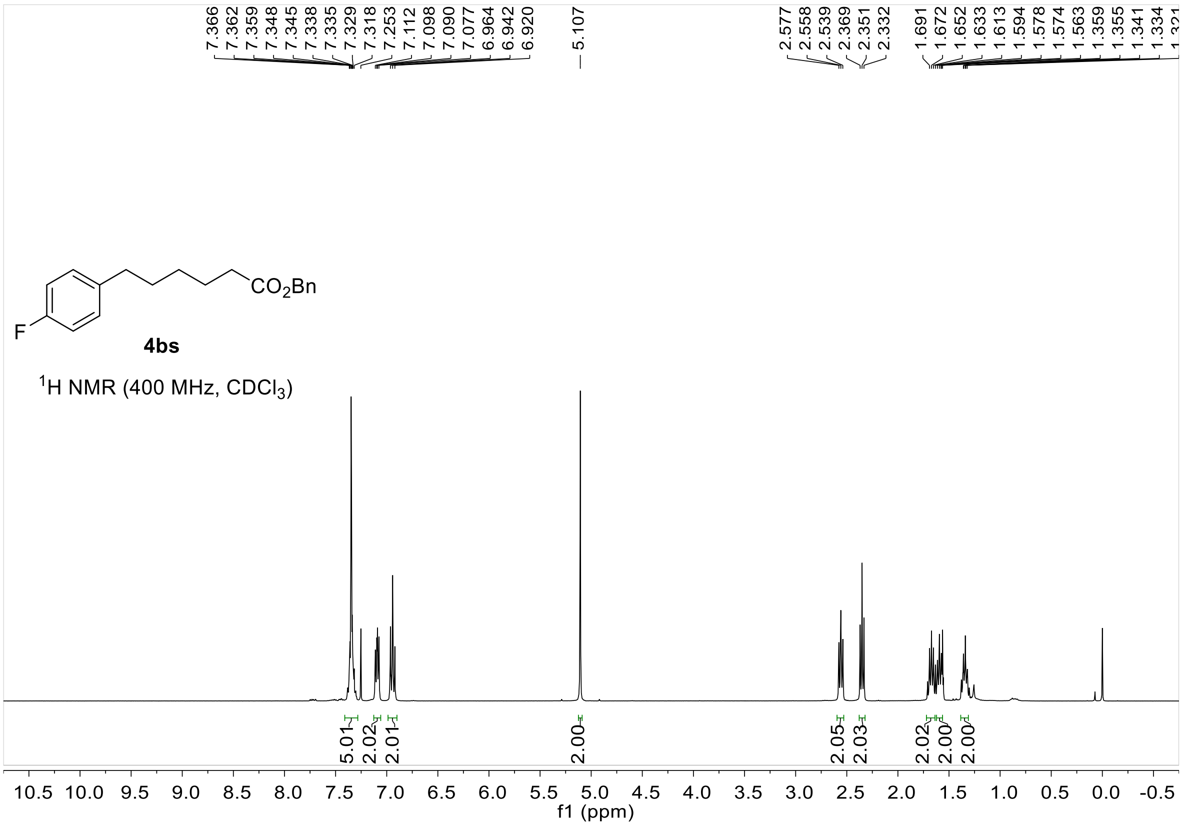

Supplementary Figure 76. ^{1}H NMR spectra of compound **4bs** (400 MHz, $CDCl_3$)

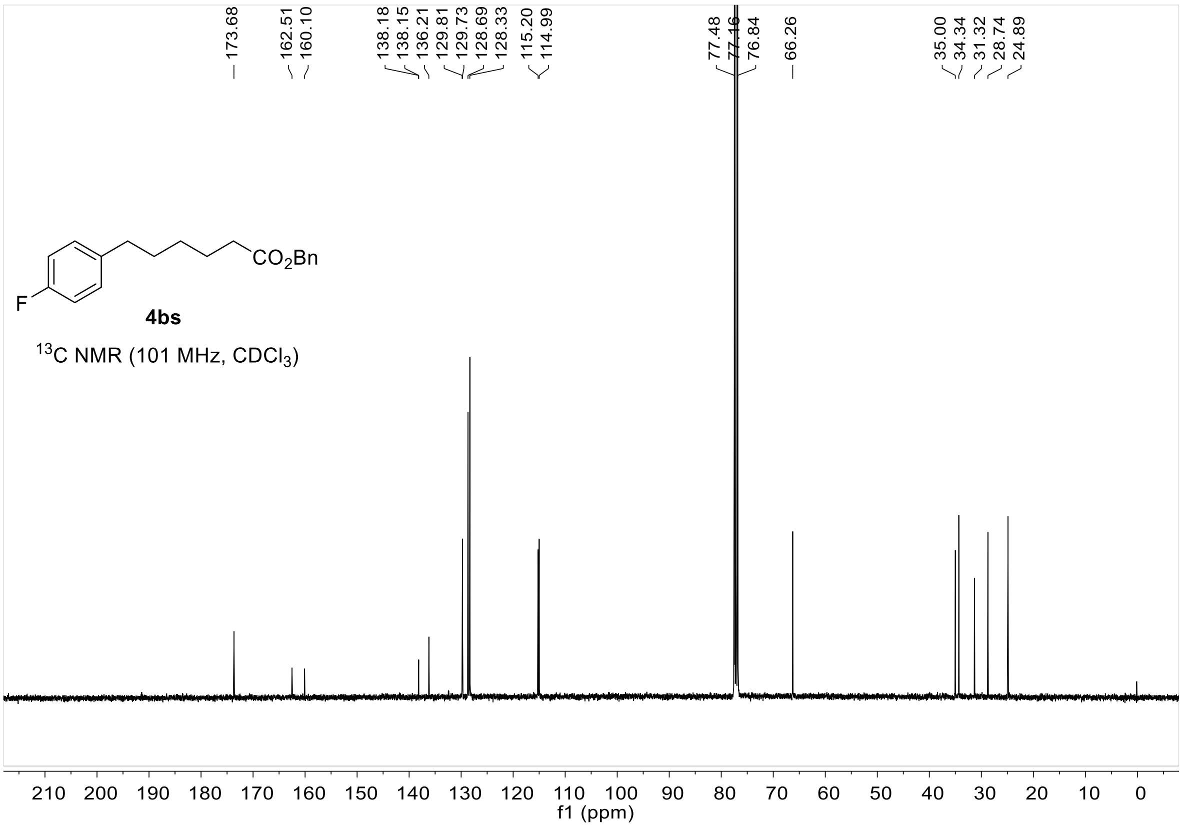

Supplementary Figure 77. ^{13}C NMR spectra of compound **4bs** (101 MHz, $CDCl_3$)

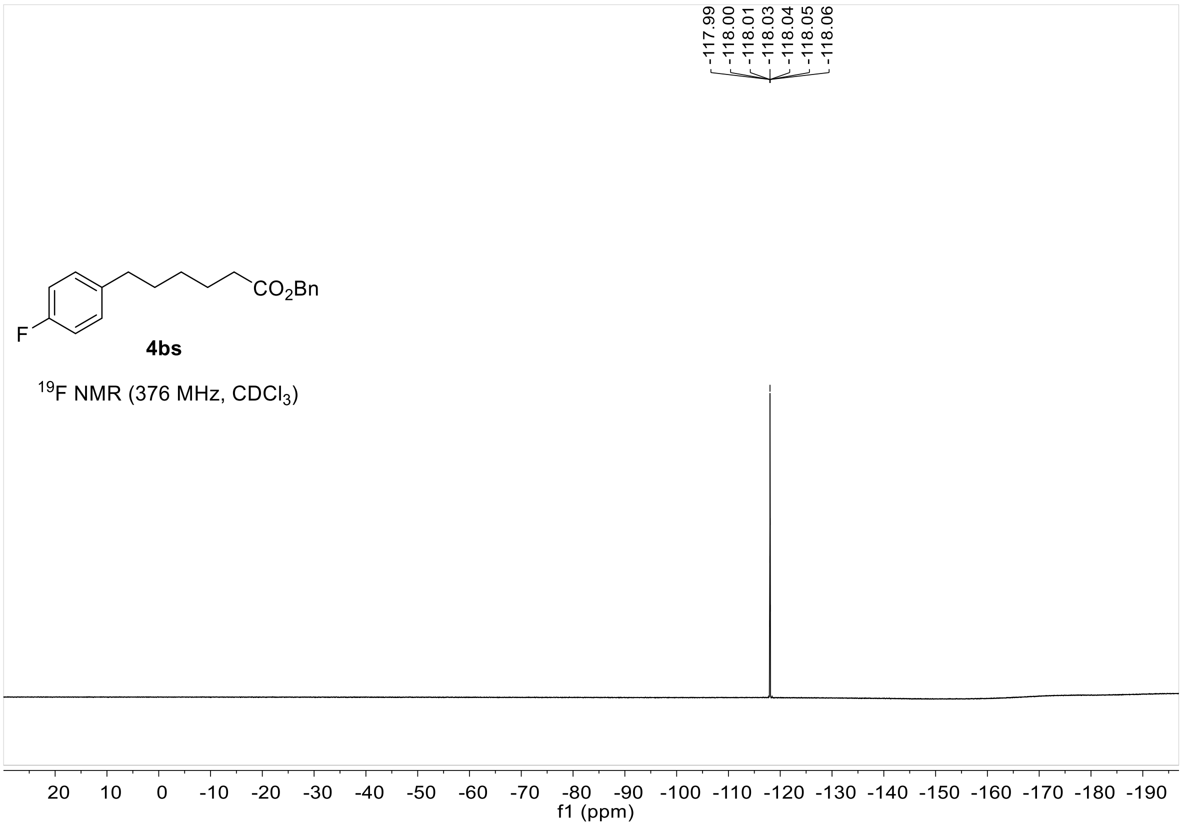

Supplementary Figure 78. ^{19}F NMR spectra of compound **4bs** (376 MHz, $CDCl_3$)

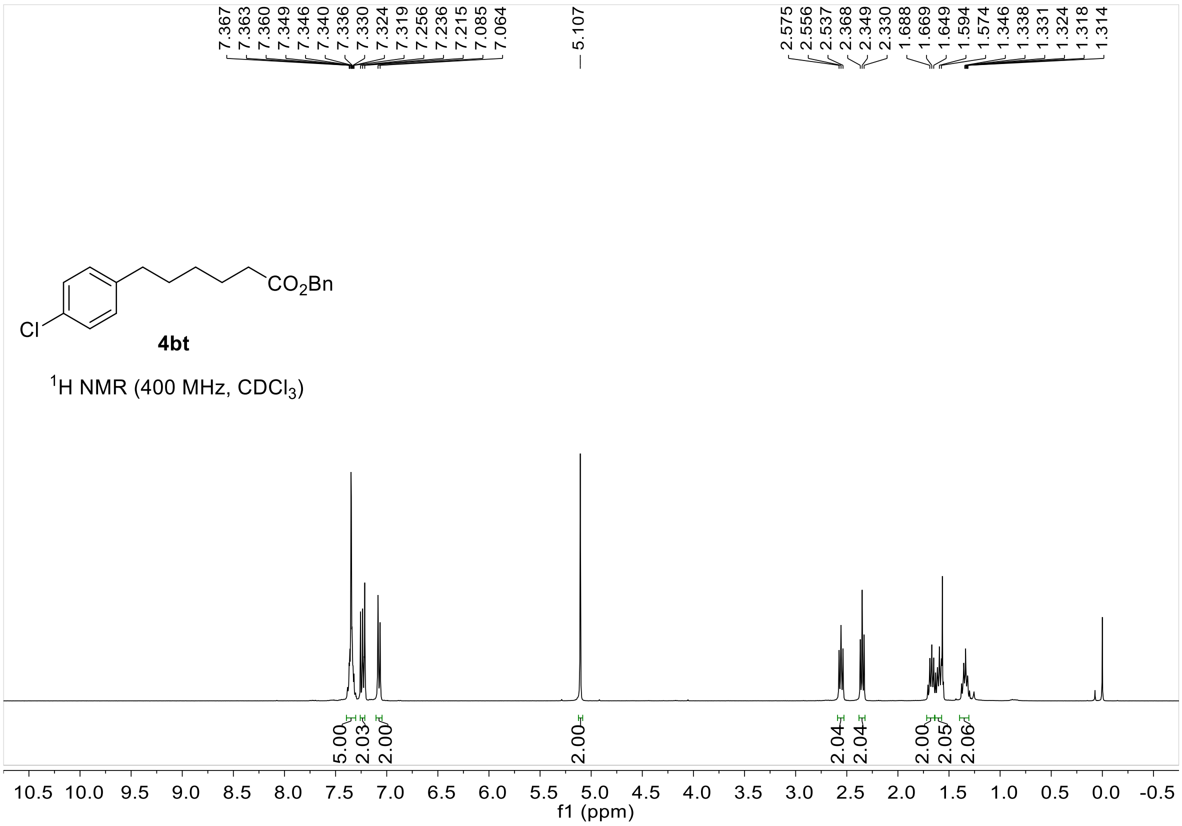

Supplementary Figure 79. ^{1}H NMR spectra of compound **4bt** (400 MHz, $CDCl_3$)

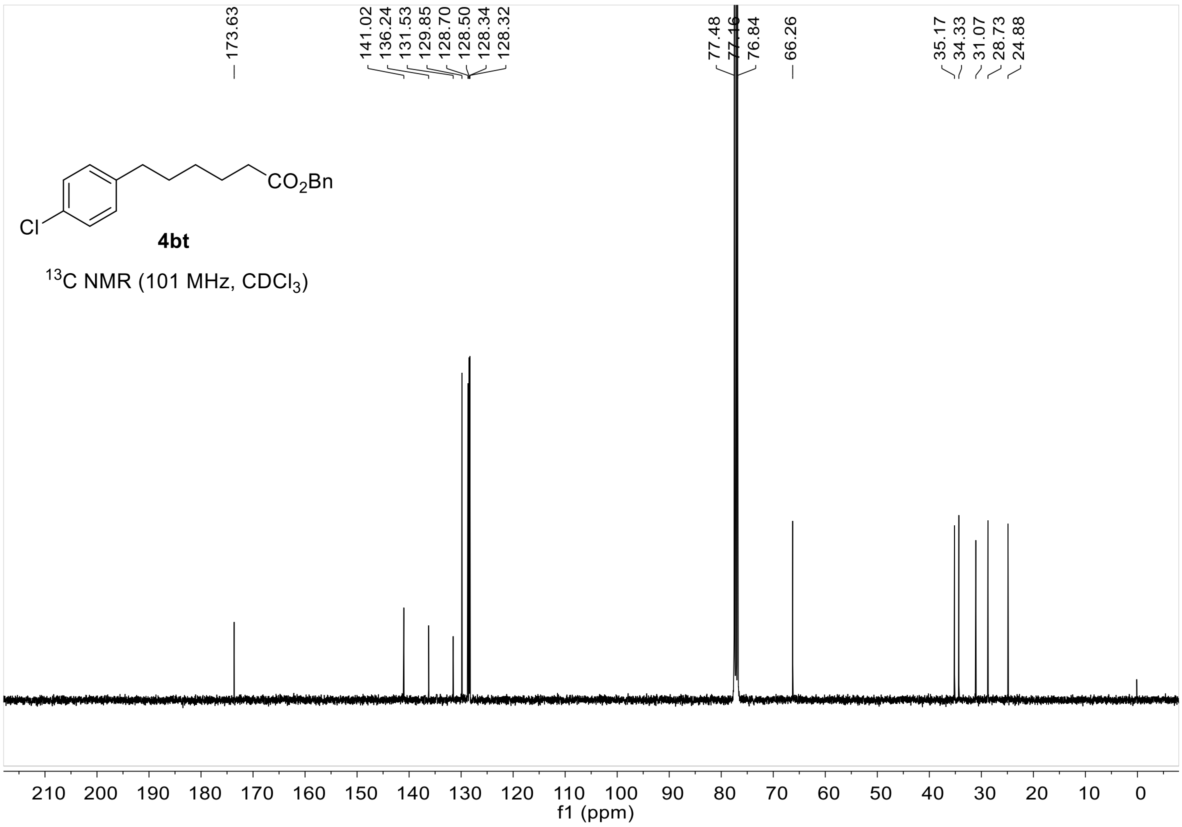

Supplementary Figure 80. ^{13}C NMR spectra of compound **4bt** (101 MHz, $CDCl_3$)

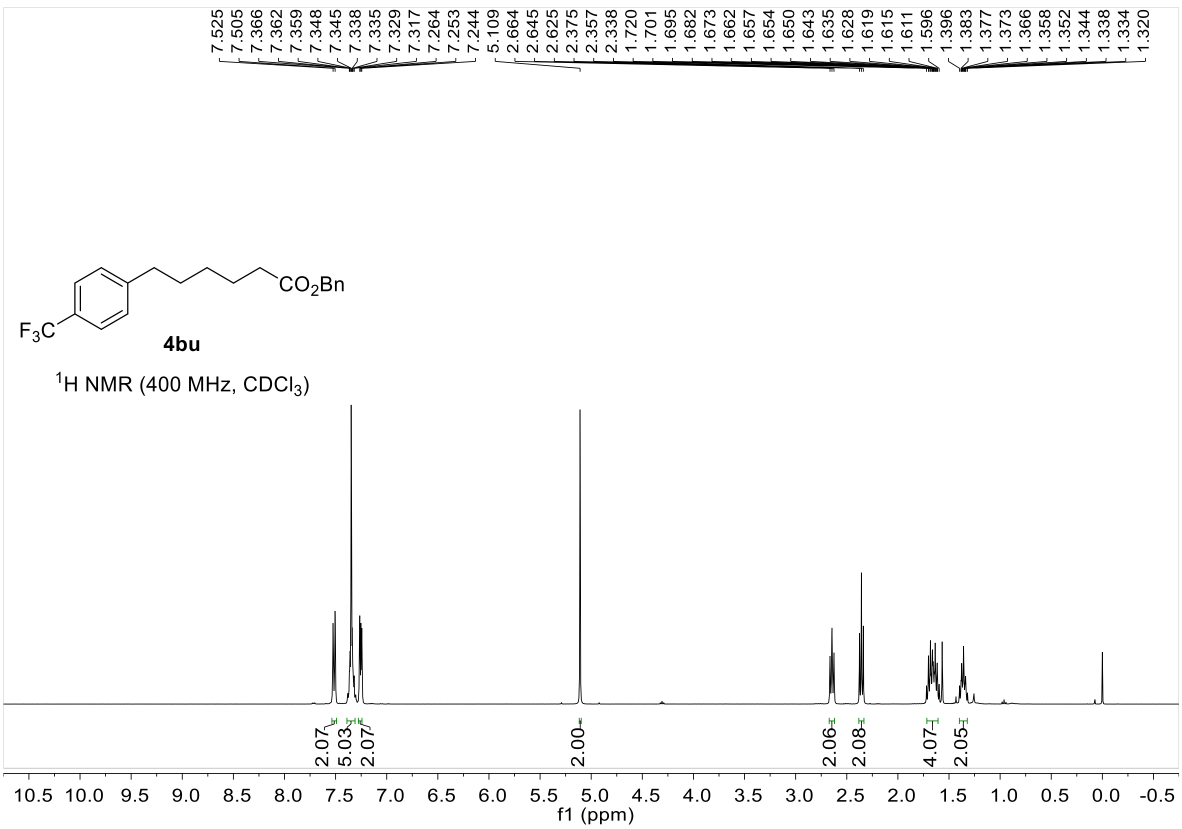

Supplementary Figure 81. ^{1}H NMR spectra of compound **4bu** (400 MHz, $CDCl_3$)

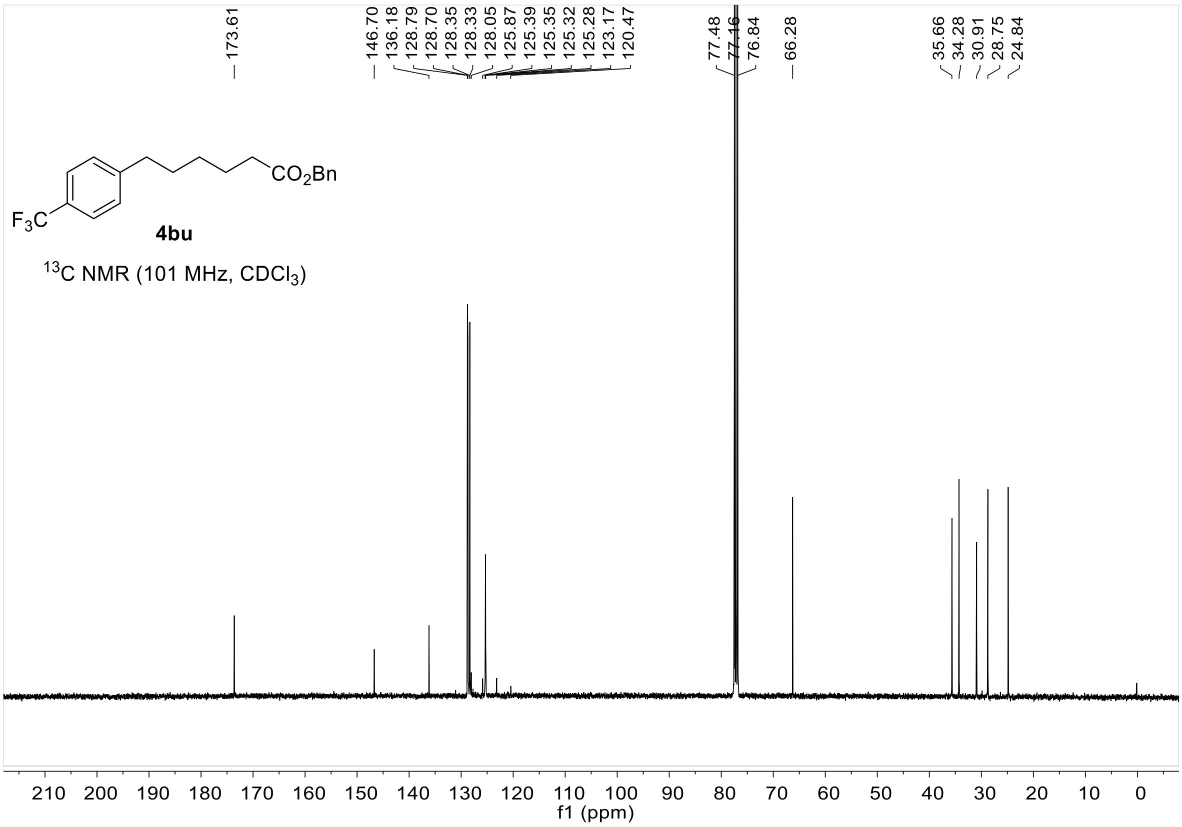

Supplementary Figure 82. ^{13}C NMR spectra of compound **4bu** (101 MHz, $CDCl_3$)

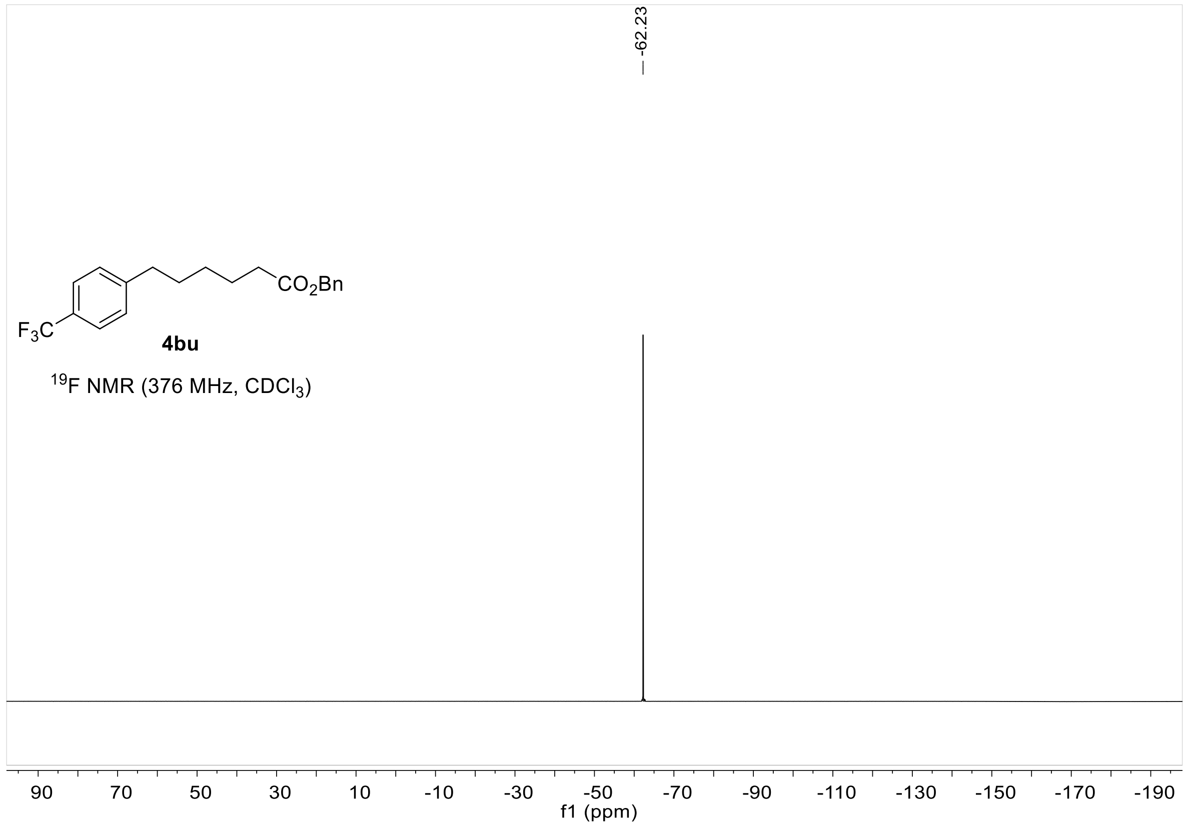

Supplementary Figure 83. ^{19}F NMR spectra of compound **4bu** (376 MHz, $CDCl_3$)

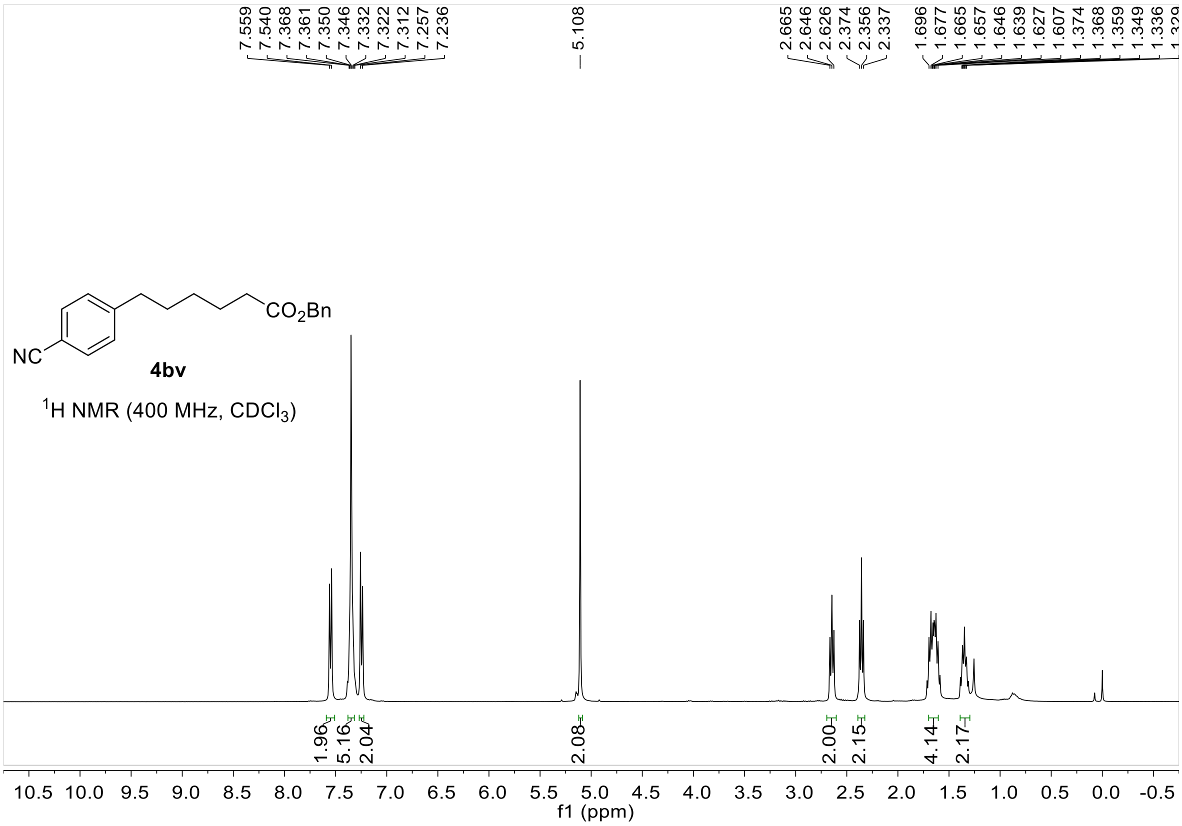

Supplementary Figure 84. ^{1}H NMR spectra of compound **4bv** (400 MHz, $CDCl_3$)

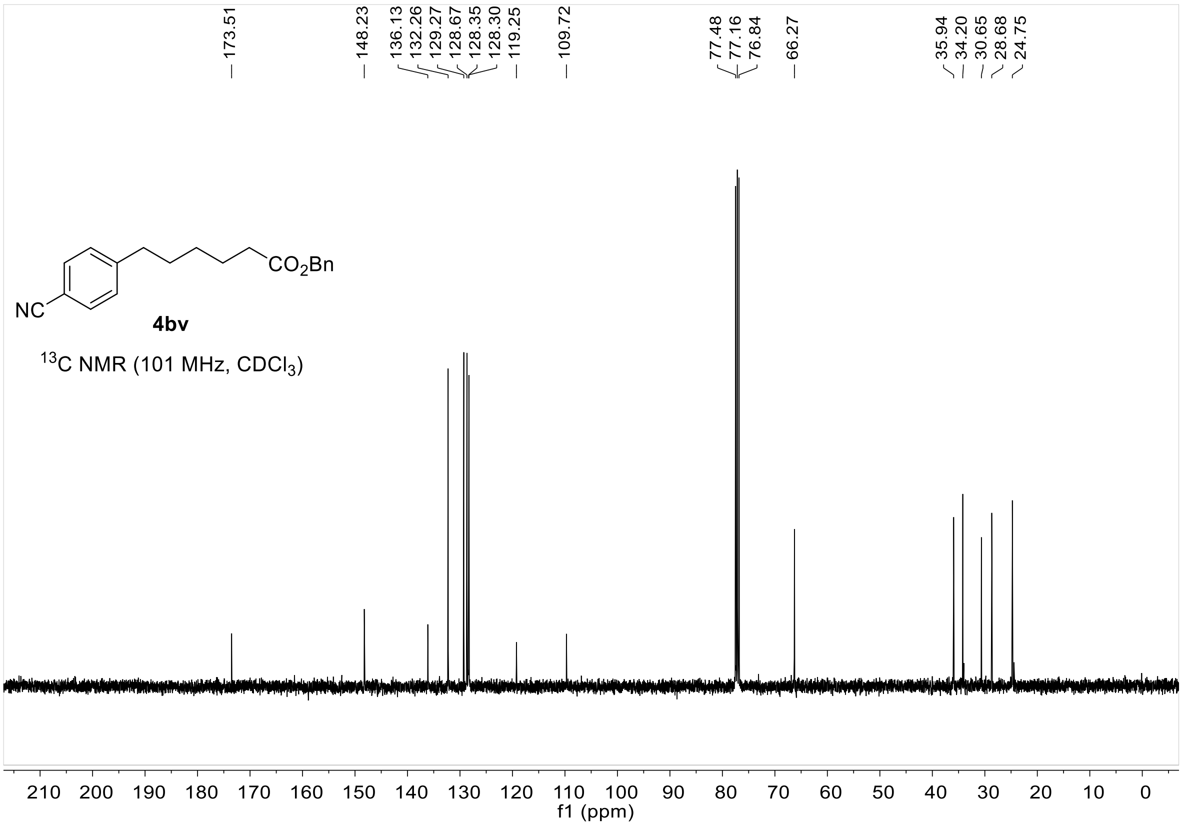

Supplementary Figure 85. ^{13}C NMR spectra of compound **4bv** (101 MHz, $CDCl_3$)

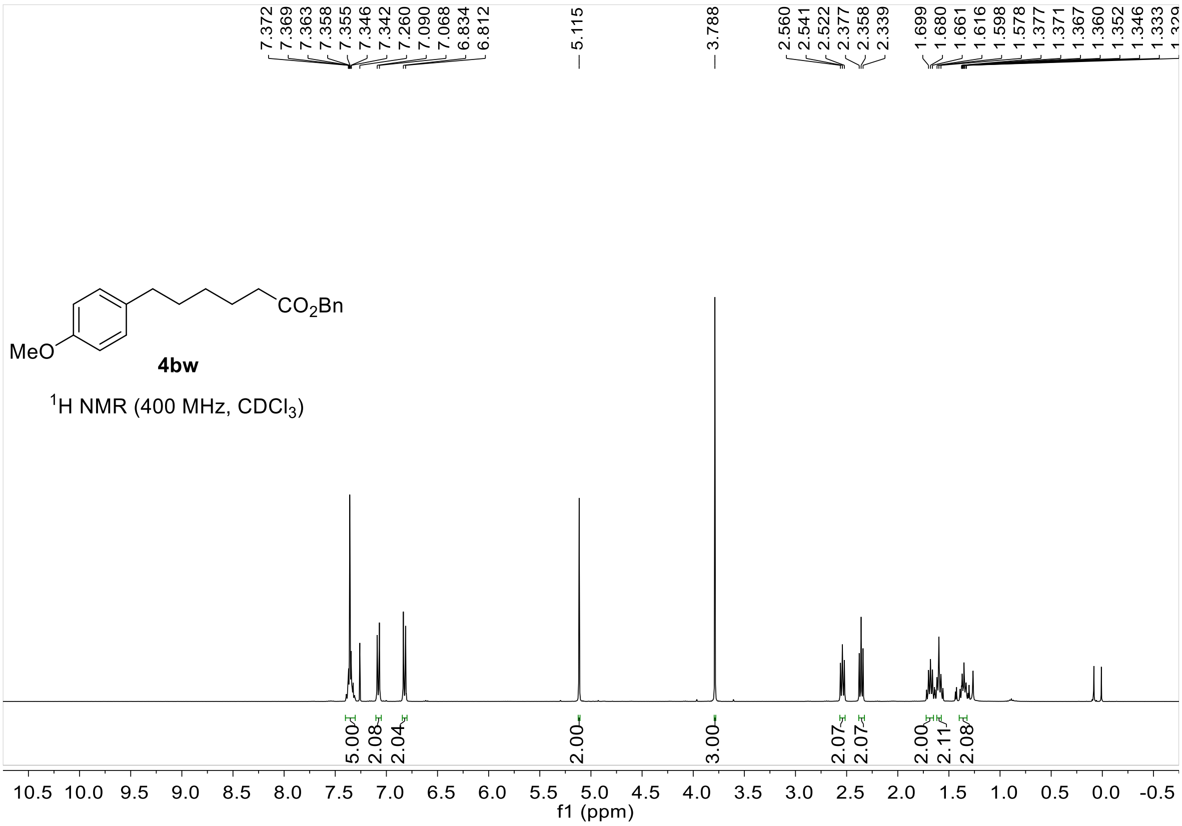

Supplementary Figure 86. ^{1}H NMR spectra of compound **4bw** (400 MHz, $CDCl_3$)

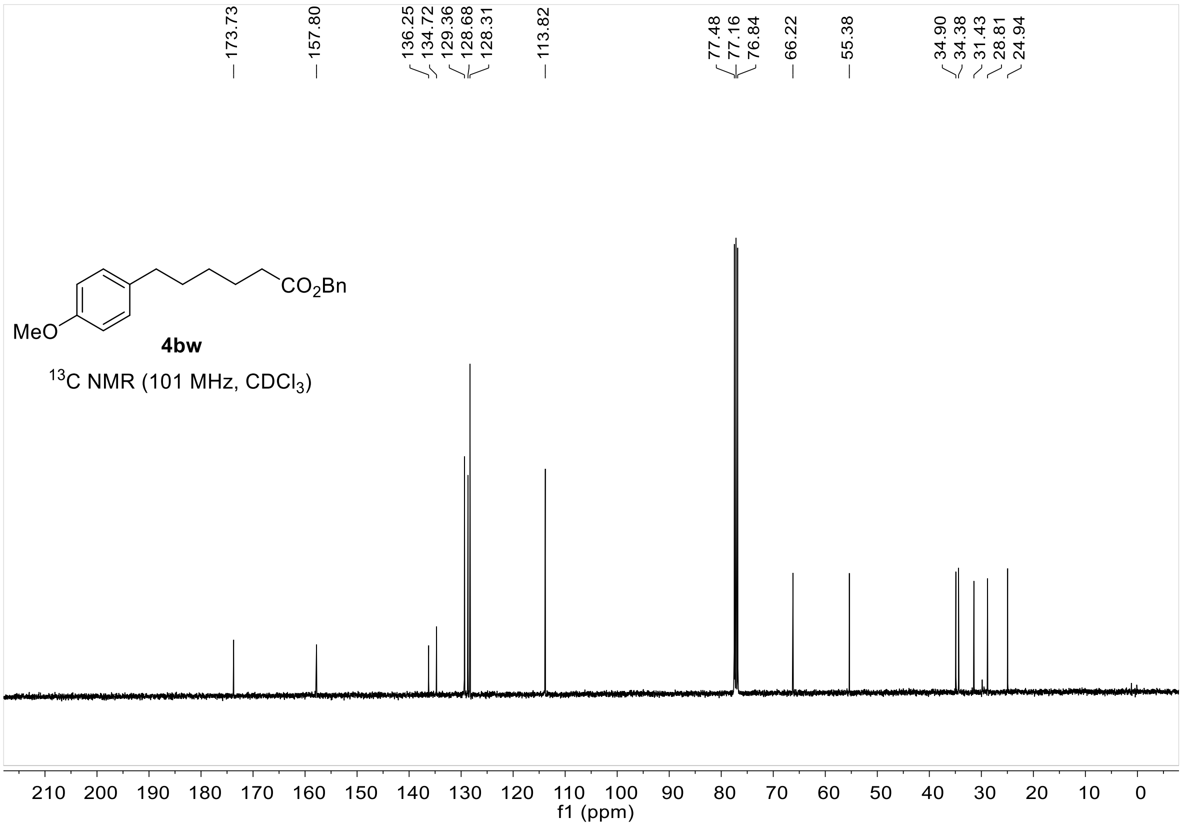

Supplementary Figure 87. ^{13}C NMR spectra of compound **4bw** (101 MHz, $CDCl_3$)

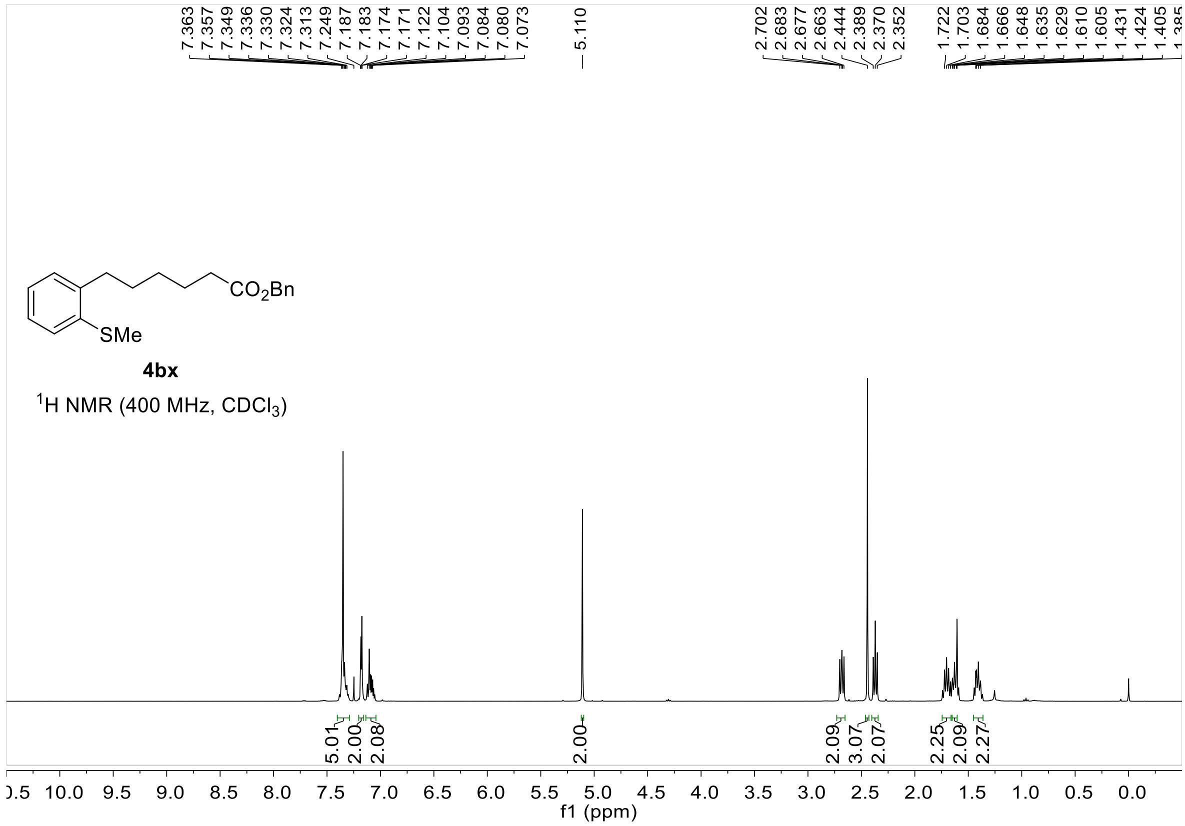

Supplementary Figure 88. ^{1}H NMR spectra of compound **4bx** (400 MHz, $CDCl_3$)

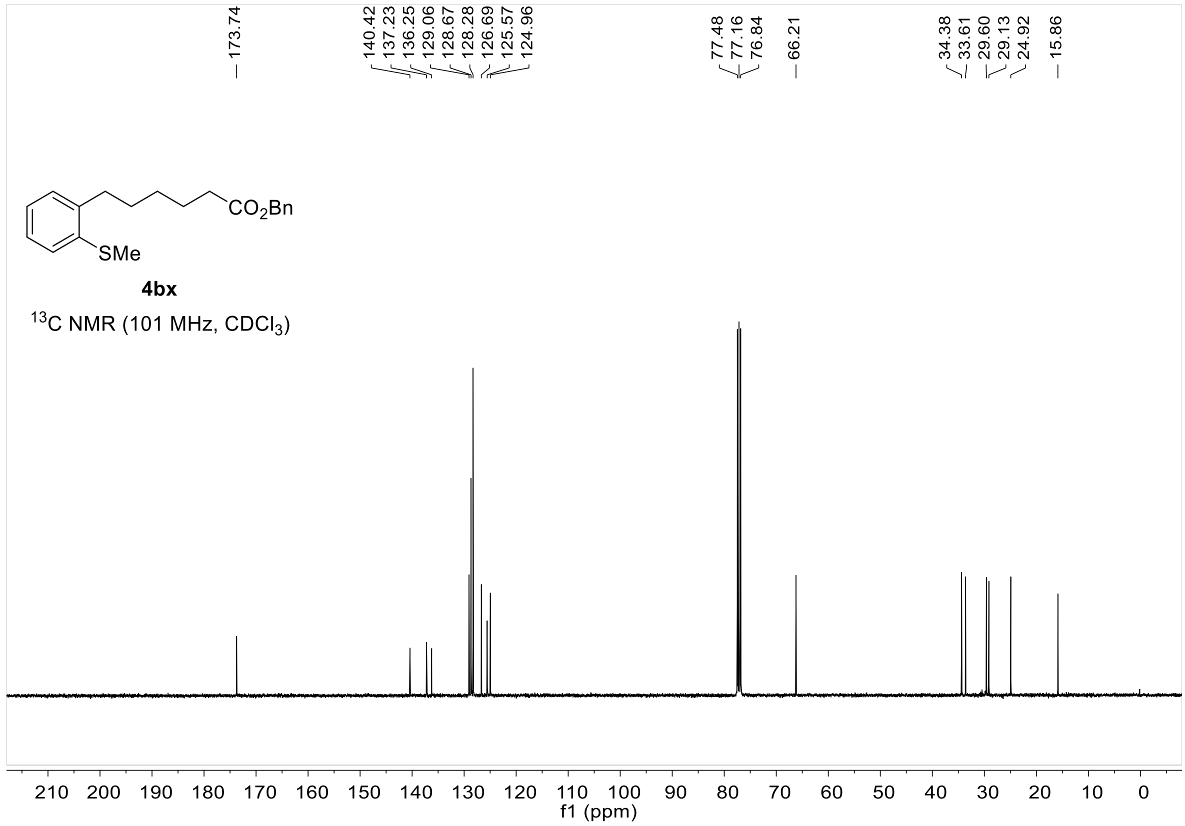

Supplementary Figure 89. ^{13}C NMR spectra of compound **4bx** (101 MHz, $CDCl_3$)

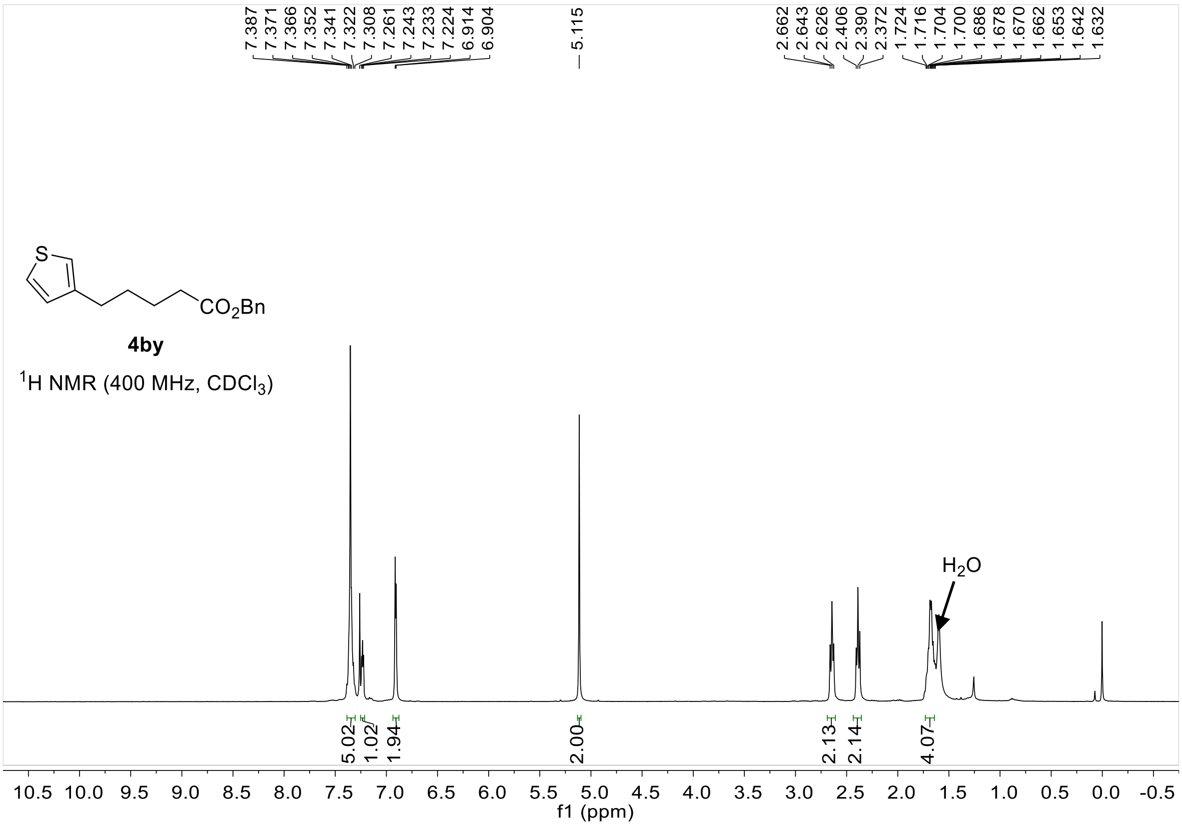

Supplementary Figure 90. ^{1}H NMR spectra of compound **4by** (400 MHz, $CDCl_3$)

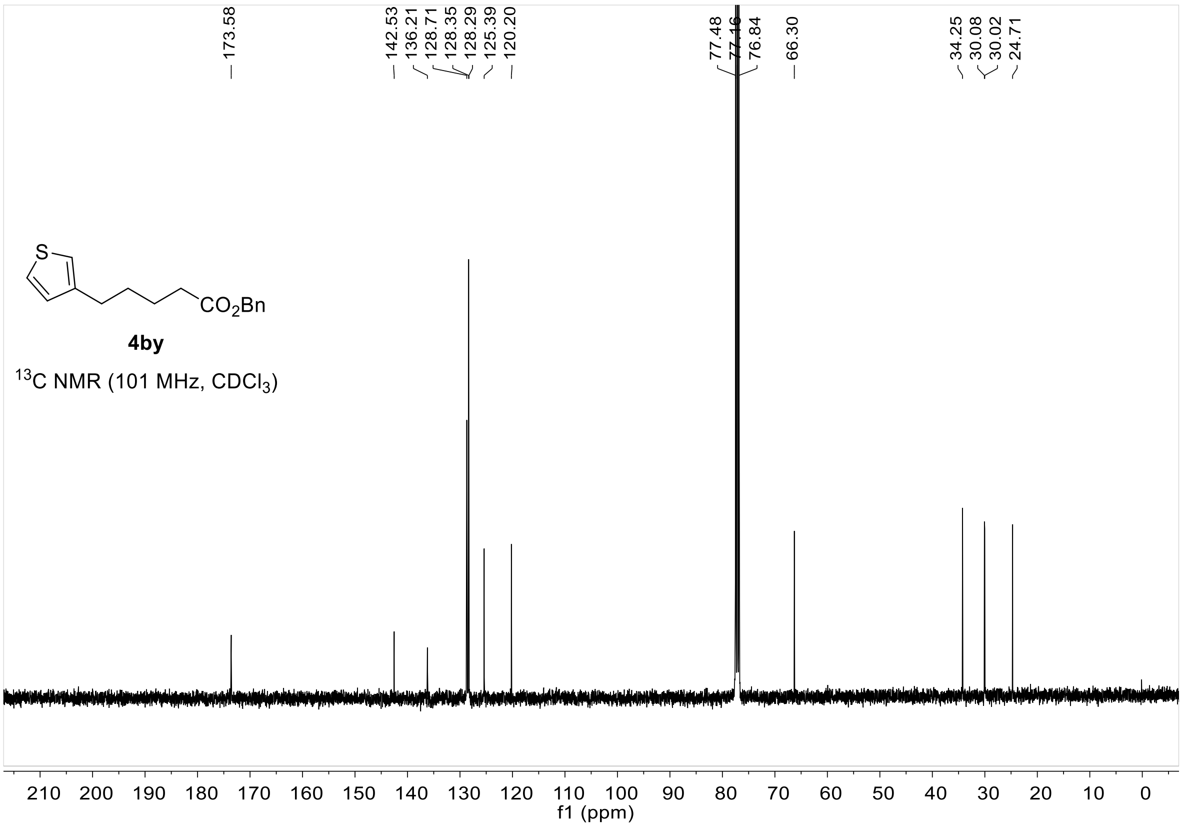

Supplementary Figure 91. ^{13}C NMR spectra of compound **4by** (101 MHz, $CDCl_3$)

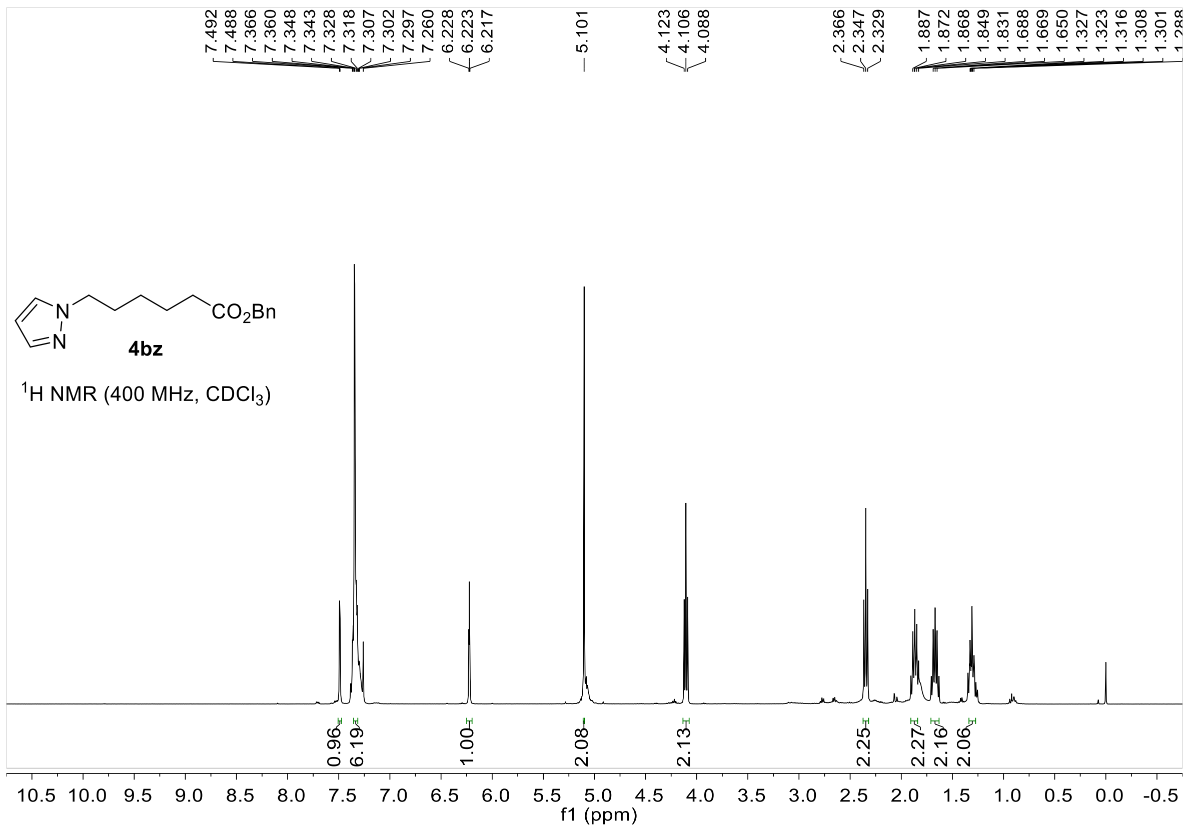

Supplementary Figure 92. ^{1}H NMR spectra of compound **4bz** (400 MHz, $CDCl_3$)

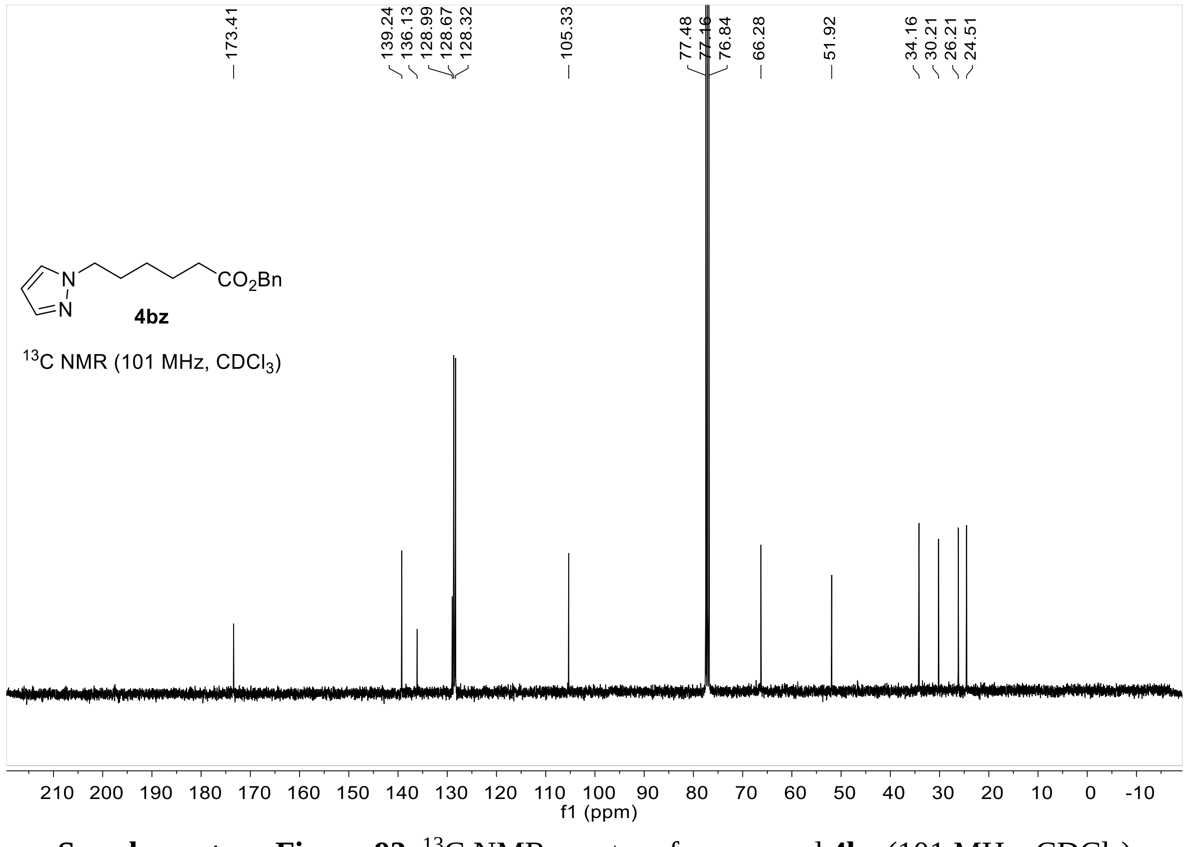

Supplementary Figure 93. ^{13}C NMR spectra of compound **4bz** (101 MHz, $CDCl_3$)

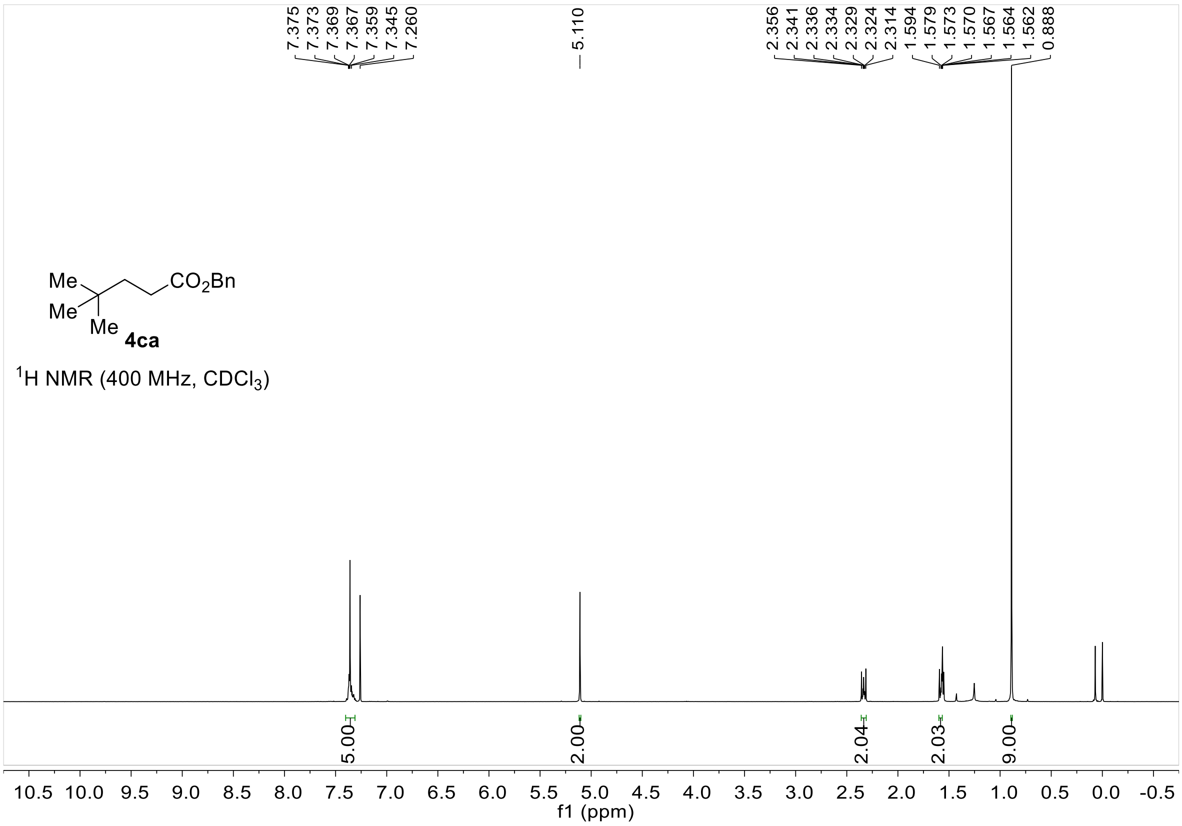

Supplementary Figure 94. ^{1}H NMR spectra of compound **4ca** (400 MHz, $CDCl_3$)

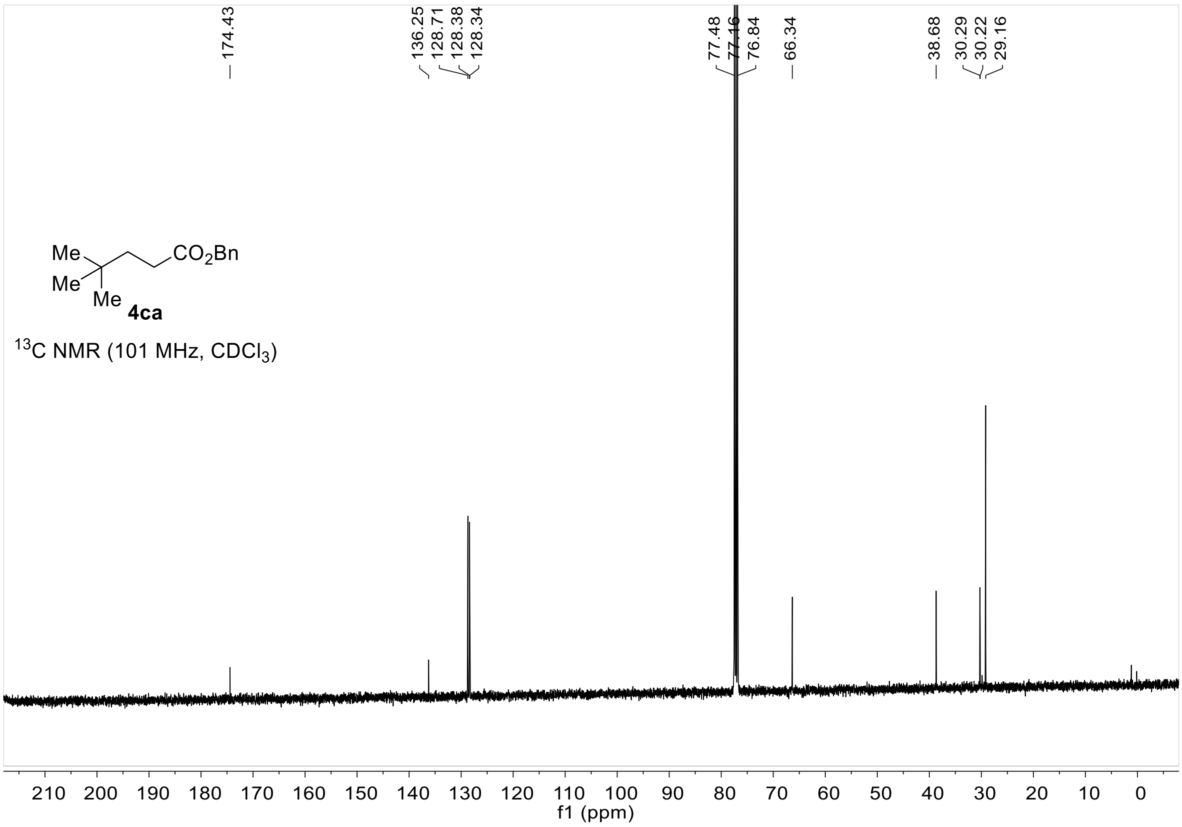

Supplementary Figure 95. ^{13}C NMR spectra of compound **4ca** (101 MHz, $CDCl_3$)

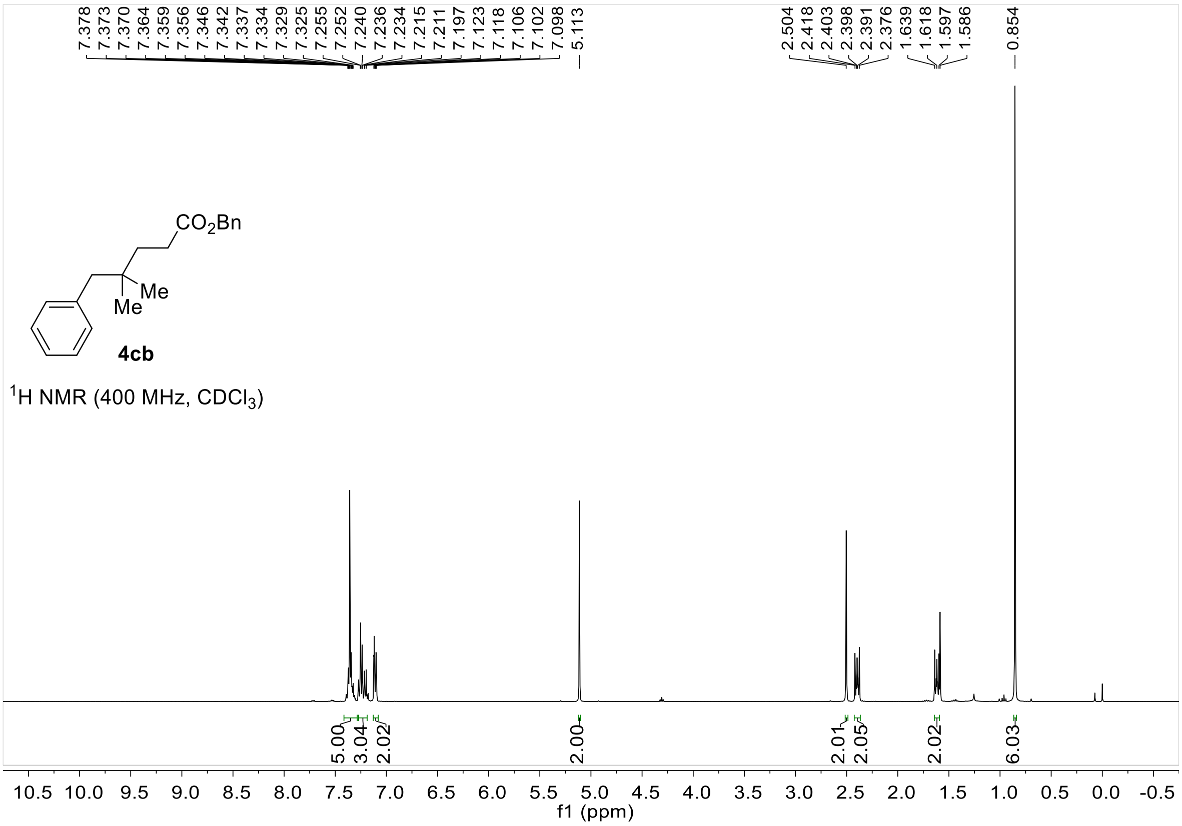

Supplementary Figure 96. ^{1}H NMR spectra of compound **4cb** (400 MHz, $CDCl_3$)

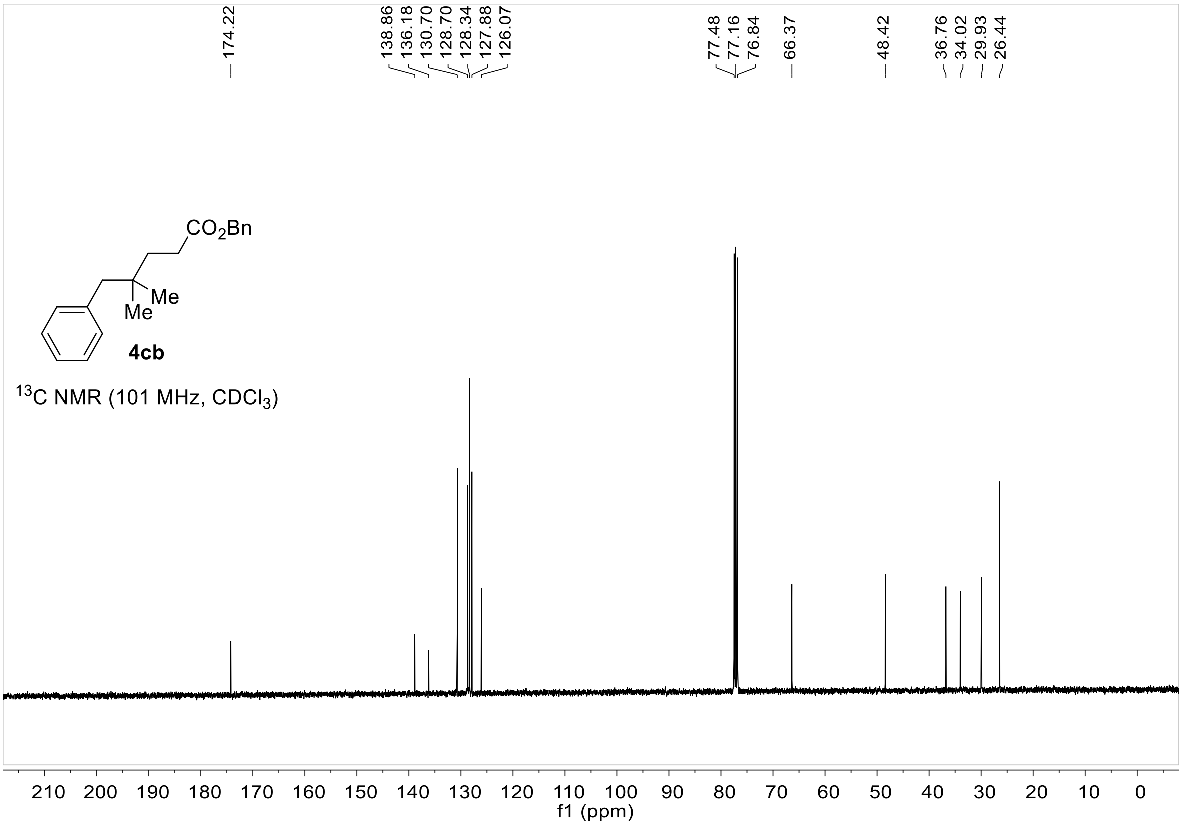

Supplementary Figure 97. ^{13}C NMR spectra of compound **4cb** (101 MHz, $CDCl_3$)

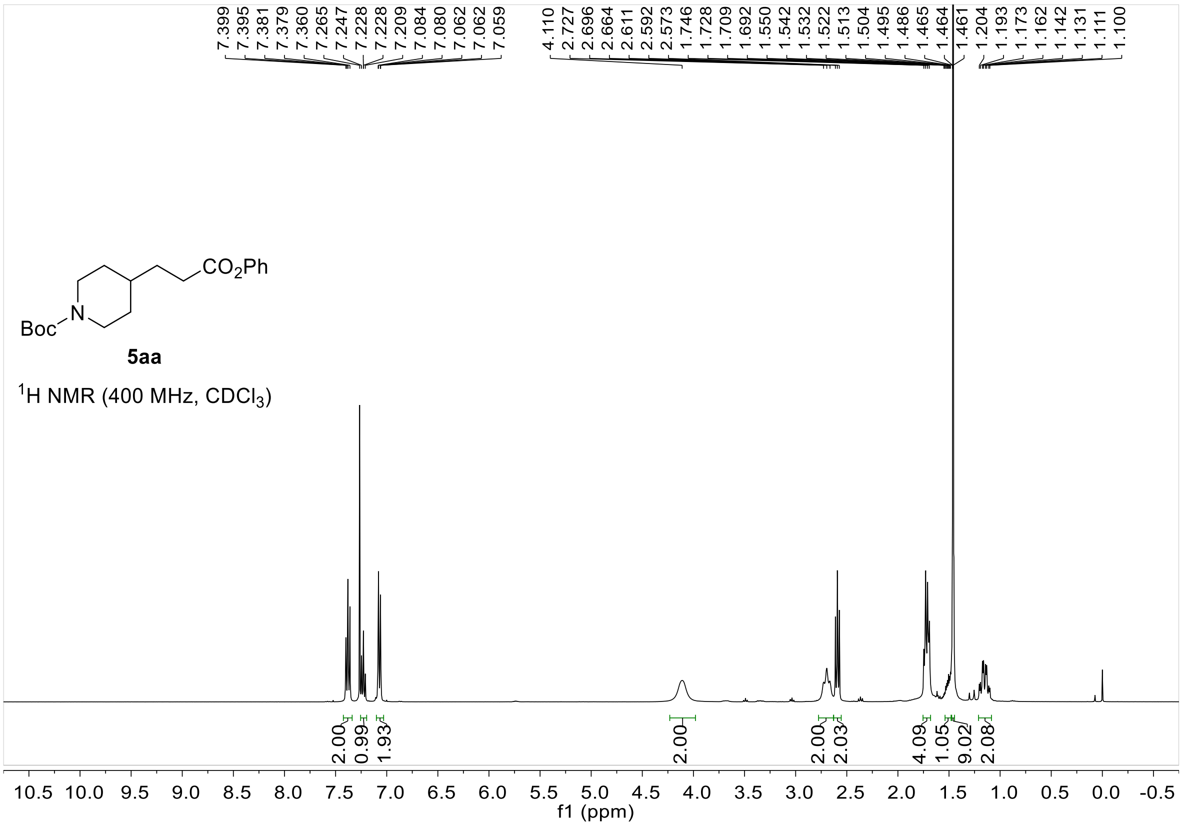

Supplementary Figure 98. ^{1}H NMR spectra of compound **5aa** (400 MHz, $CDCl_3$)

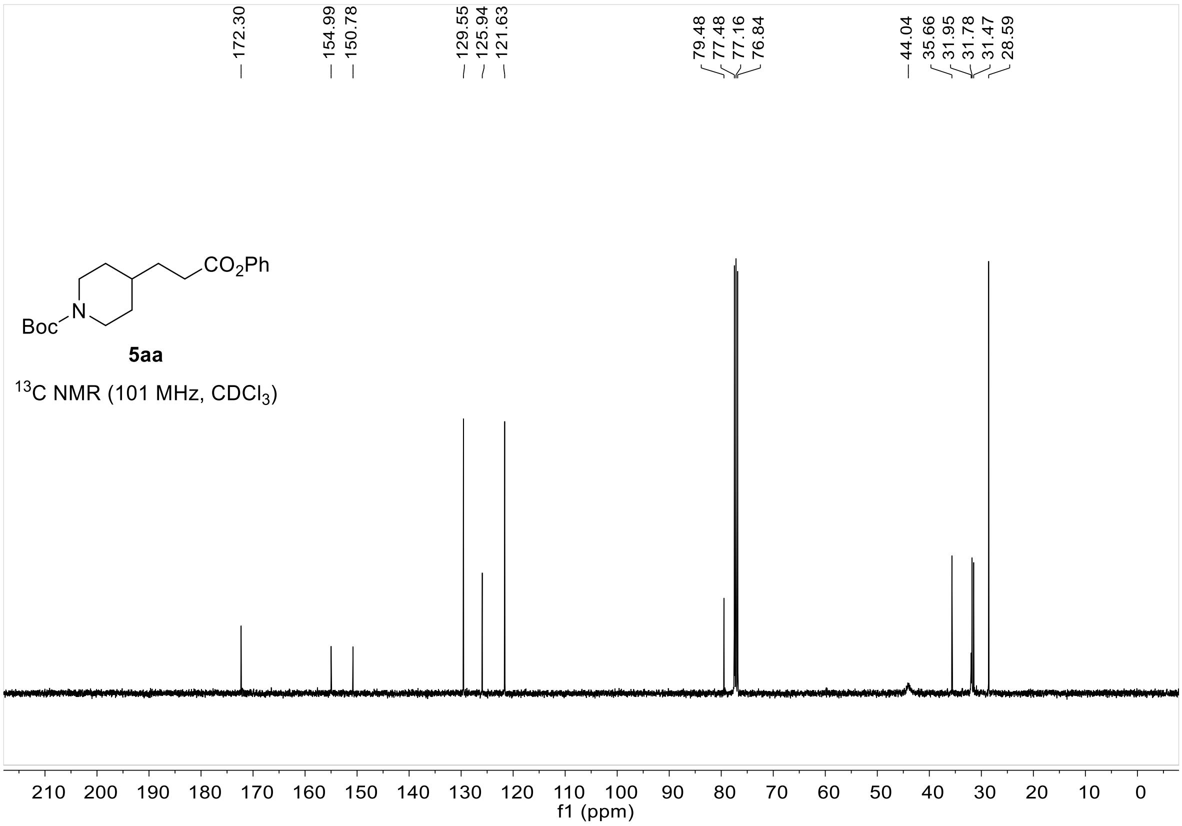

Supplementary Figure 99. ^{13}C NMR spectra of compound **5aa** (101 MHz, $CDCl_3$)

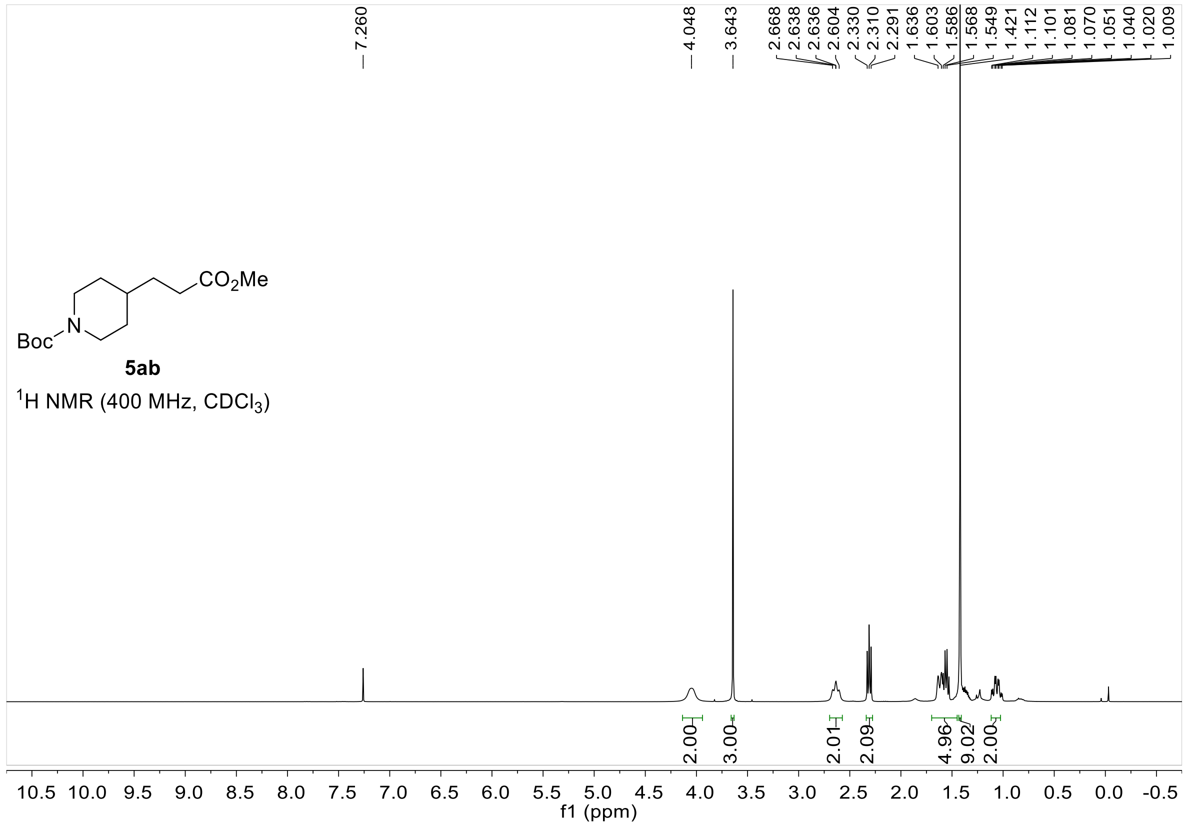

Supplementary Figure 100. ^{1}H NMR spectra of compound **5ab** (400 MHz, $CDCl_3$)

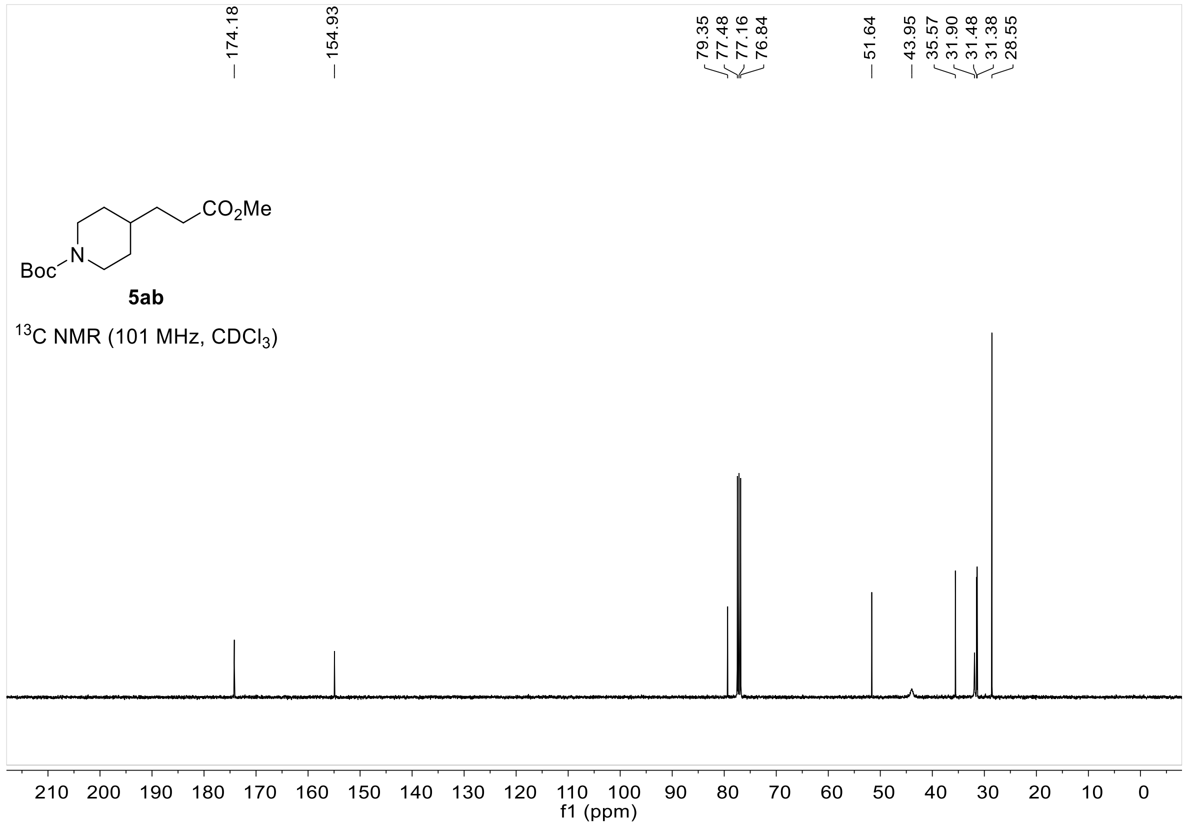

Supplementary Figure 101. ^{13}C NMR spectra of compound **5ab** (101 MHz, $CDCl_3$)

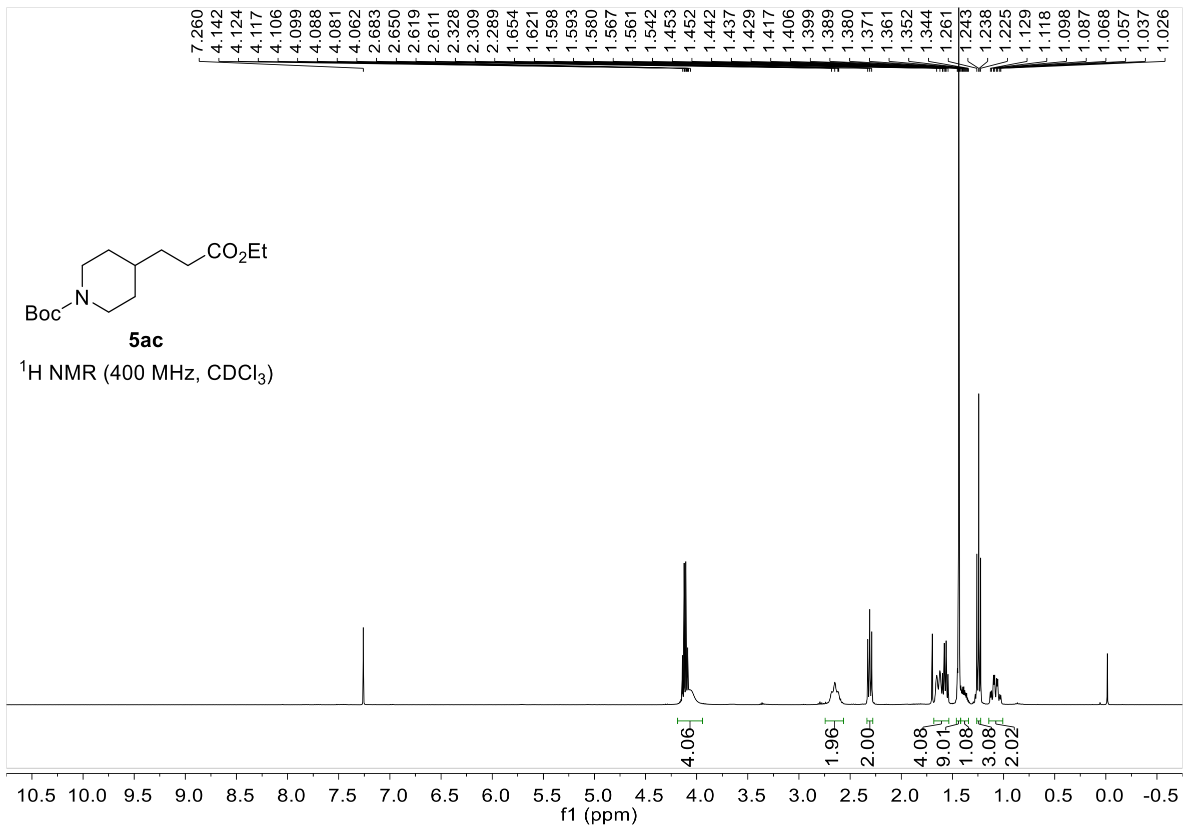

Supplementary Figure 102. ^{1}H NMR spectra of compound **5ac** (400 MHz, $CDCl_3$)

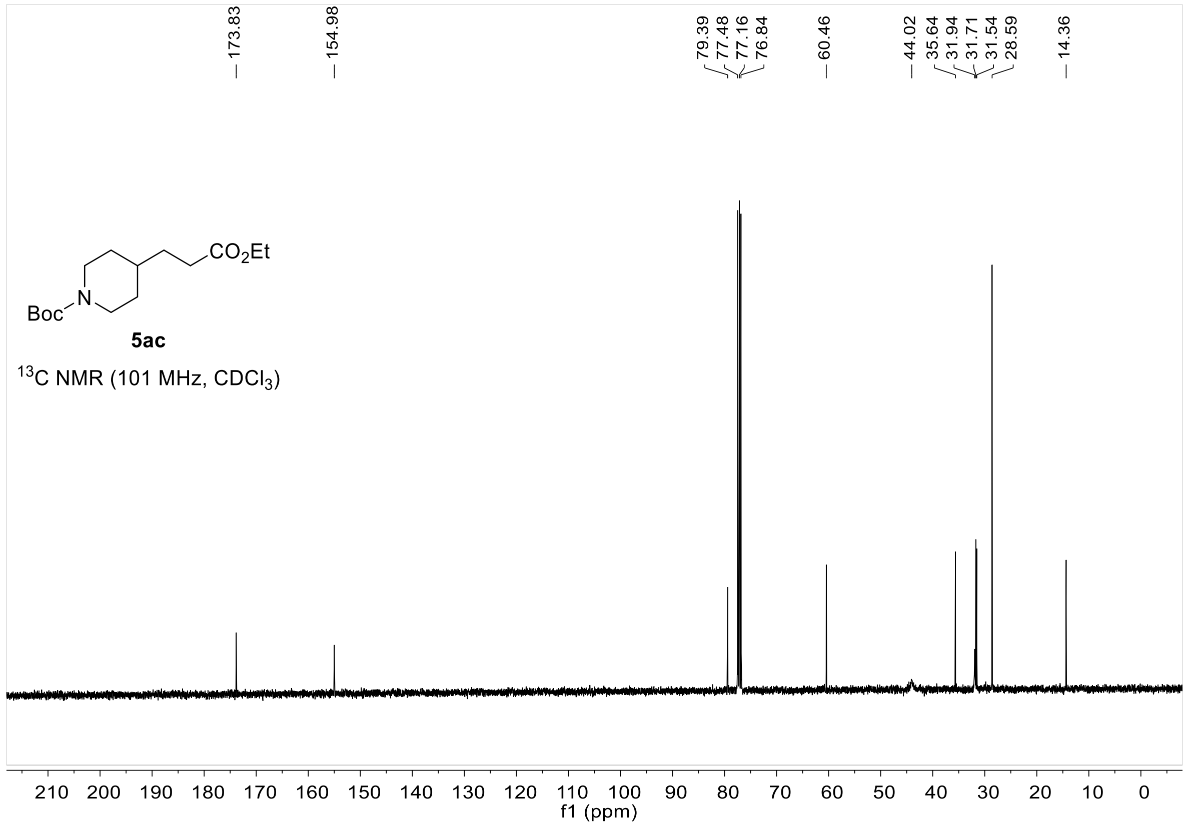

Supplementary Figure 103. ^{13}C NMR spectra of compound **5ac** (101 MHz, $CDCl_3$)

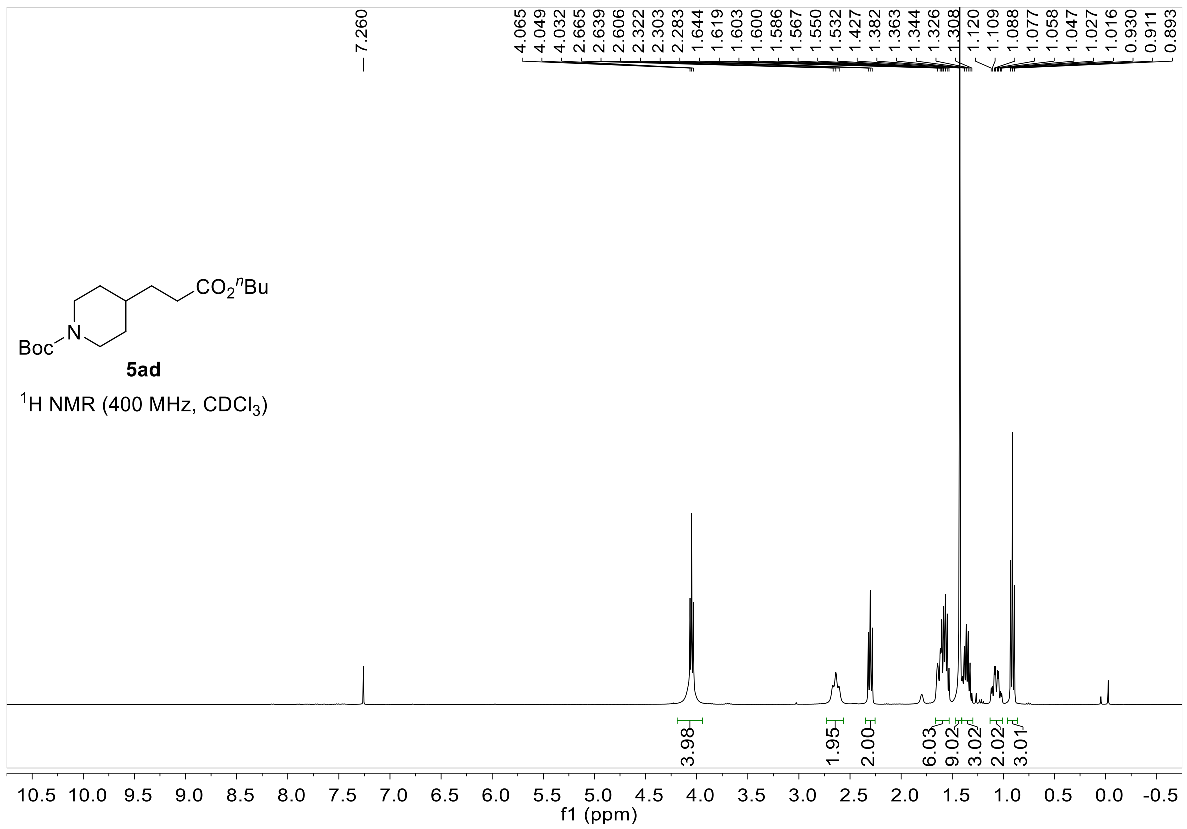

Supplementary Figure 104. ^{1}H NMR spectra of compound **5ad** (400 MHz, $CDCl_3$)

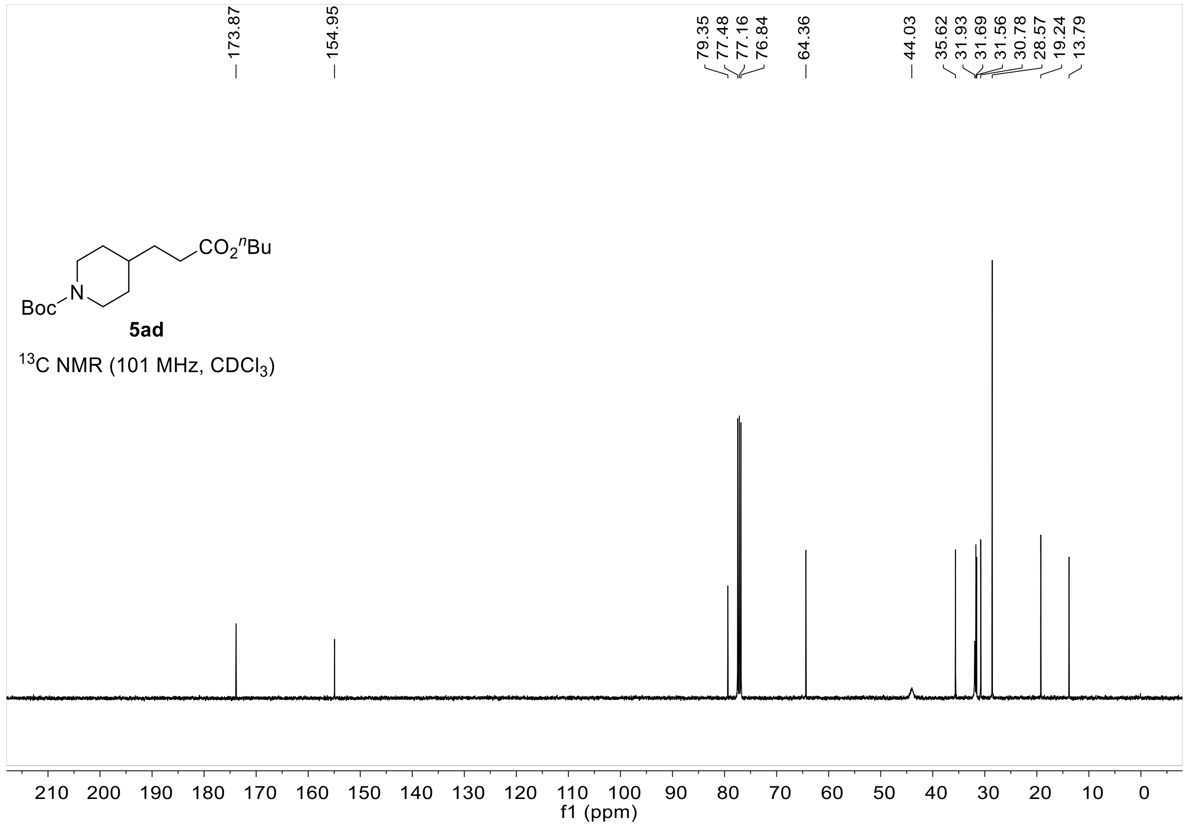

Supplementary Figure 105. ^{13}C NMR spectra of compound **5ad** (101 MHz, $CDCl_3$)

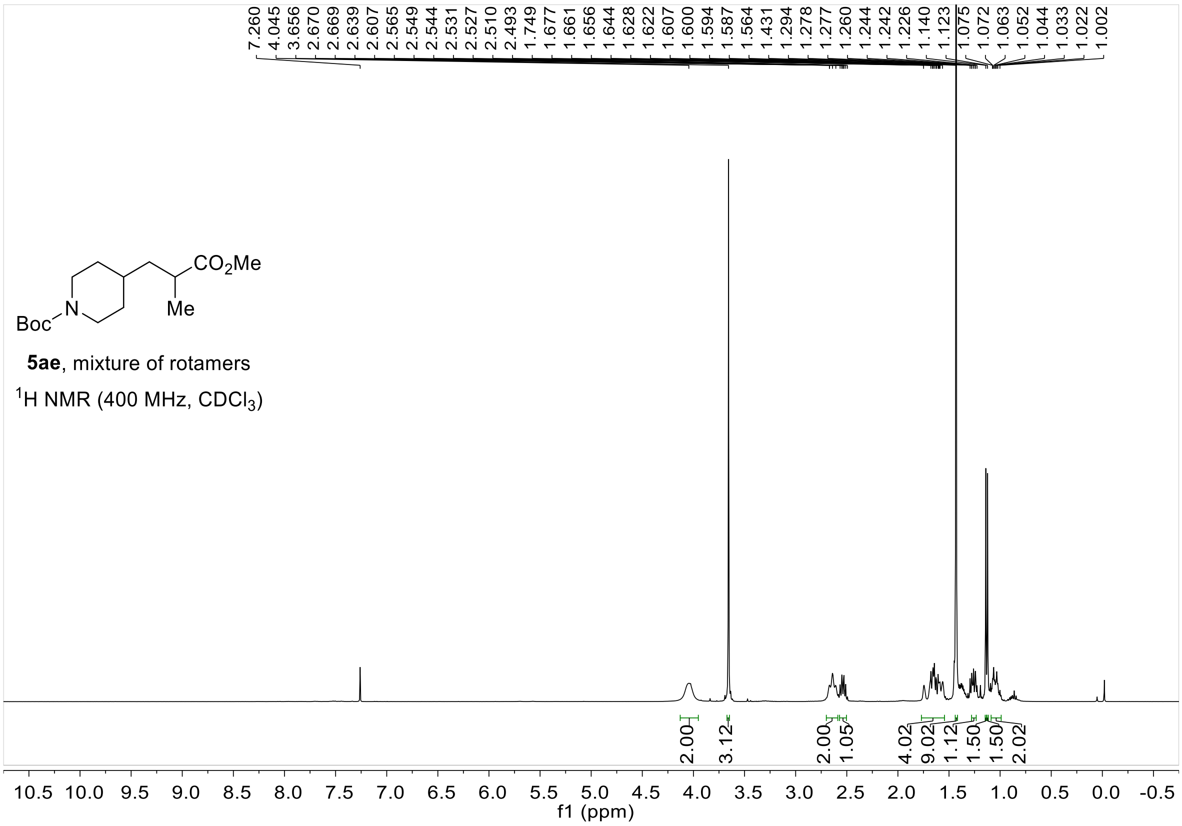

Supplementary Figure 106. ^{1}H NMR spectra of compound **5ae** (400 MHz, $CDCl_3$)

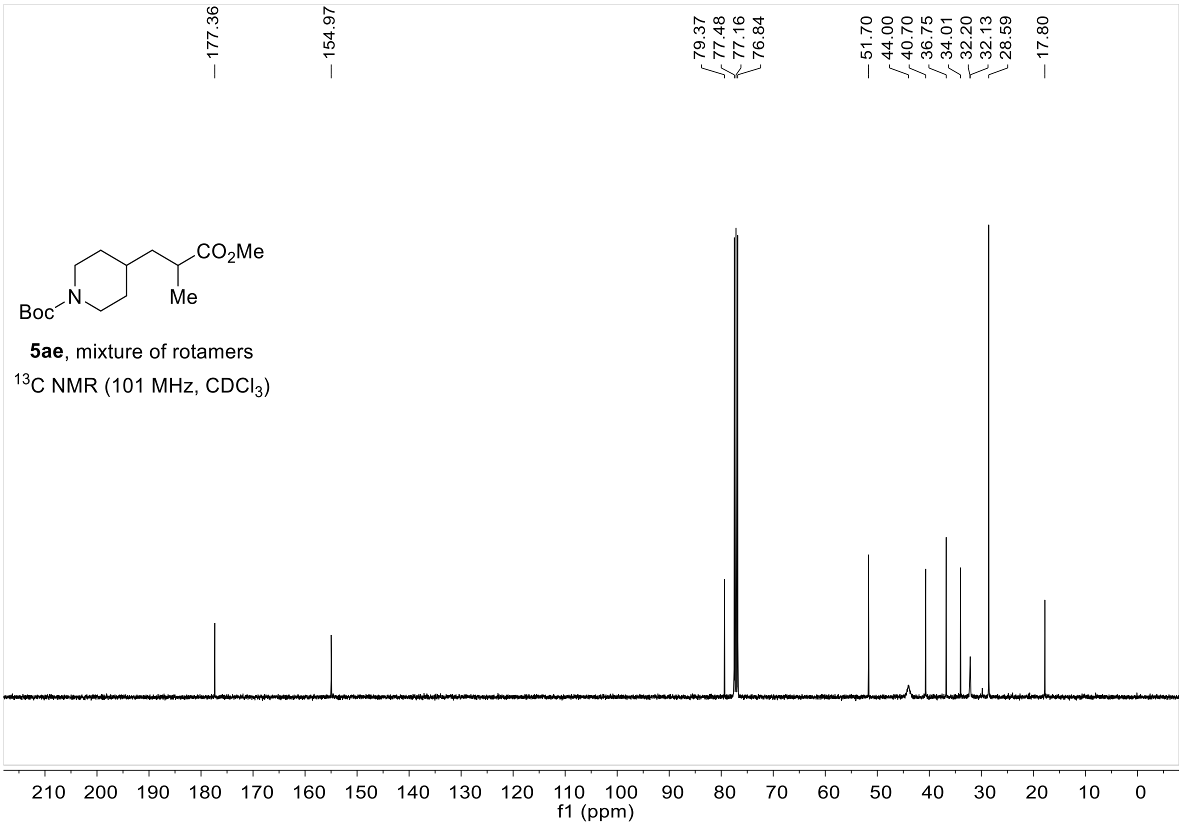

Supplementary Figure 107. ^{13}C NMR spectra of compound **5ae** (101 MHz, $CDCl_3$)

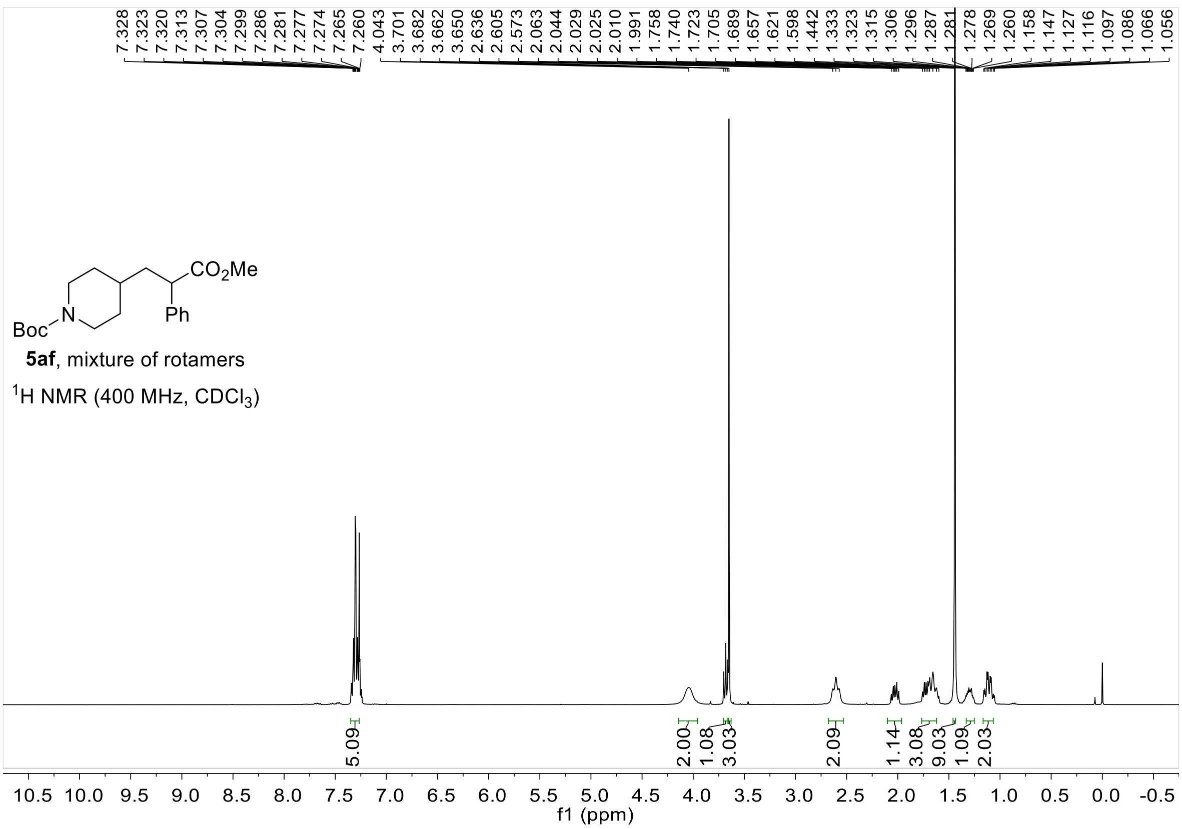

Supplementary Figure 108. ^{1}H NMR spectra of compound **5af** (400 MHz, $CDCl_3$)

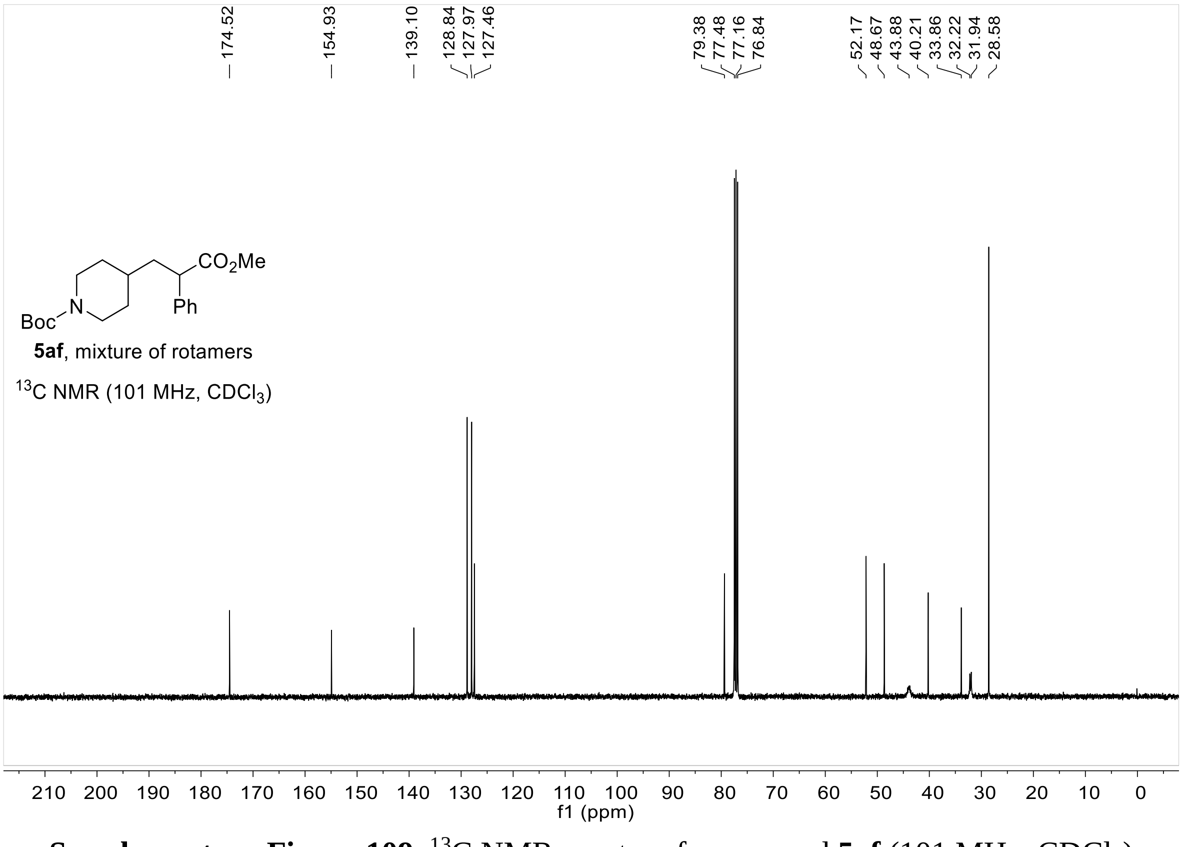

Supplementary Figure 109. ^{13}C NMR spectra of compound **5af** (101 MHz, $CDCl_3$)

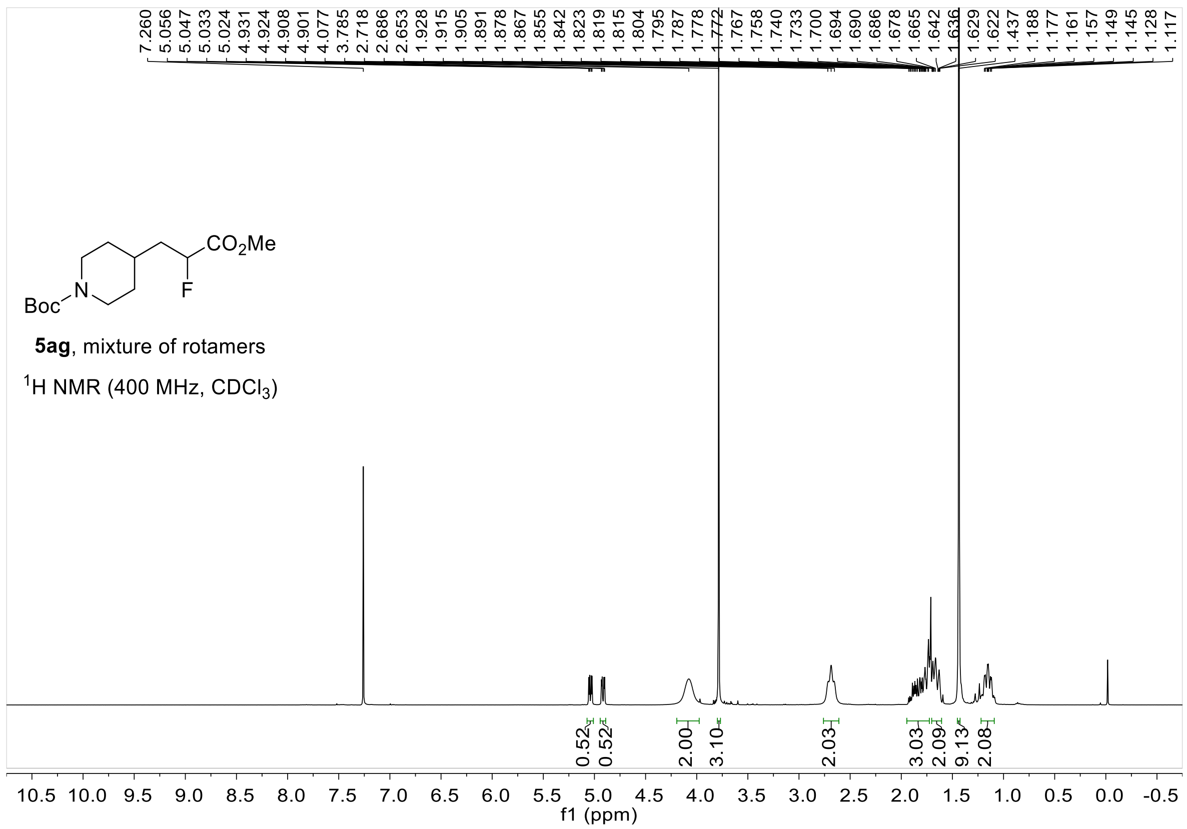

Supplementary Figure 110. ^{1}H NMR spectra of compound **5ag** (400 MHz, $CDCl_3$)

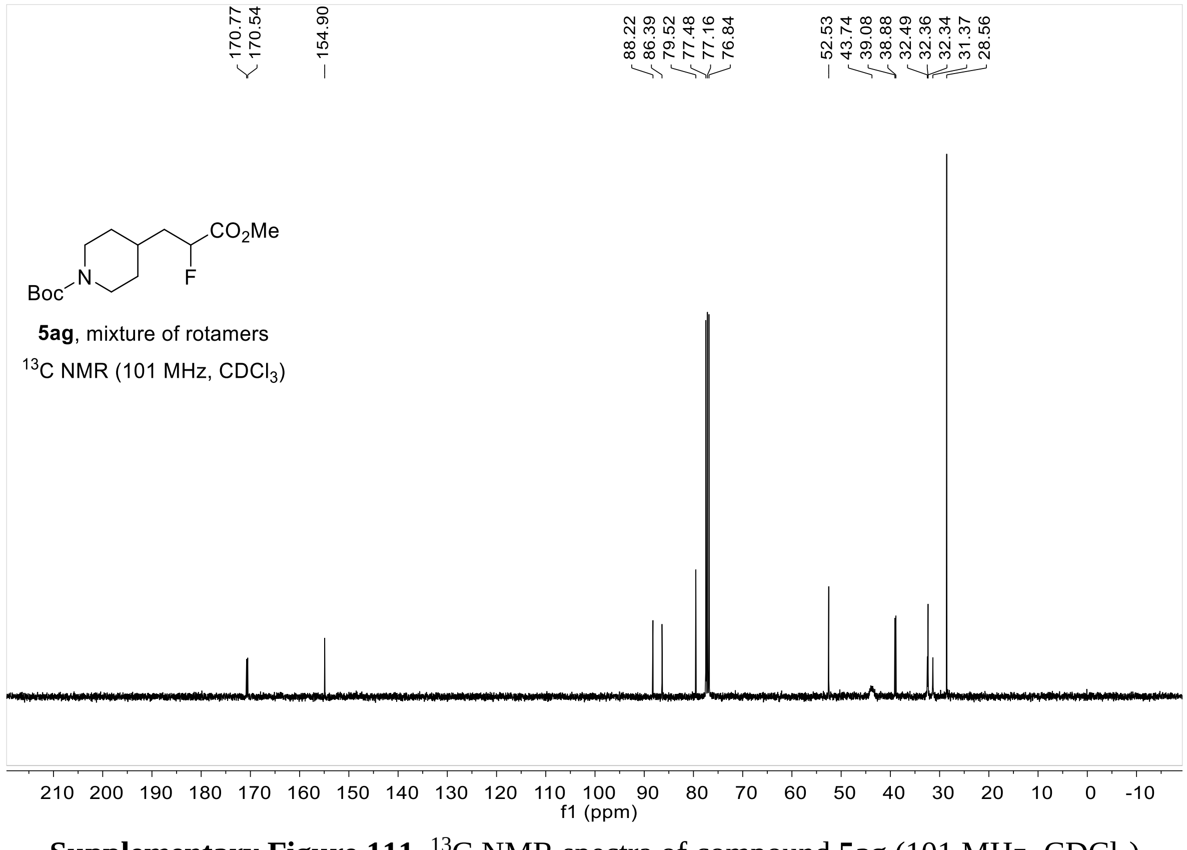

Supplementary Figure 111. ^{13}C NMR spectra of compound **5ag** (101 MHz, $CDCl_3$)

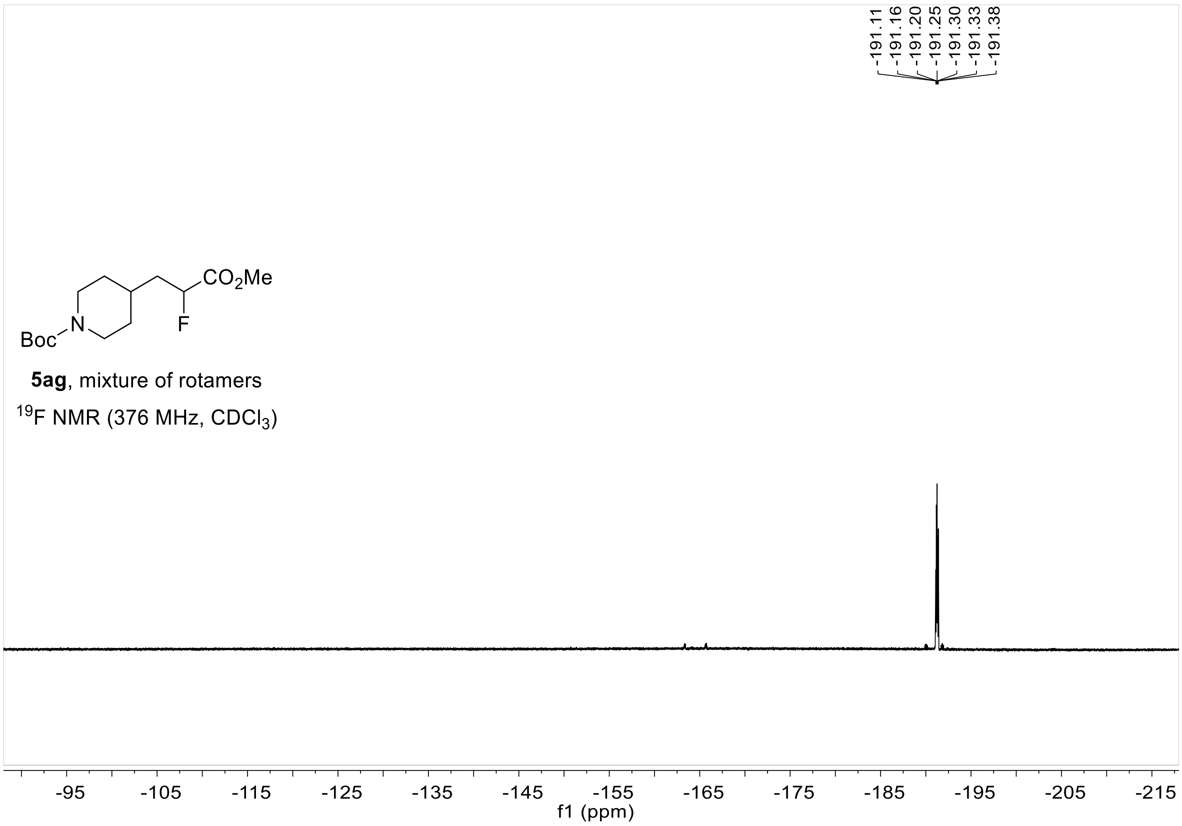

Supplementary Figure 112. ^{19}F NMR spectra of compound **5ag** (376 MHz, $CDCl_3$)

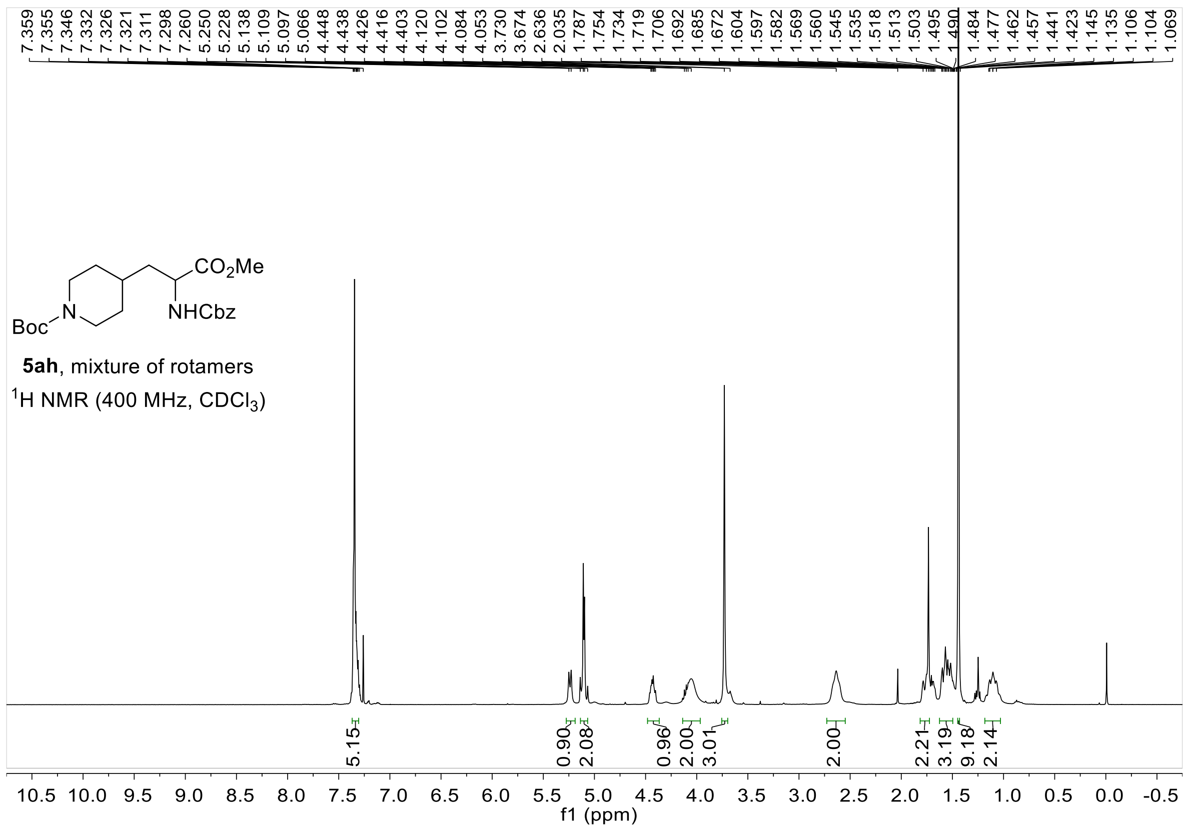

Supplementary Figure 113. ^{1}H NMR spectra of compound **5ah** (400 MHz, $CDCl_3$)

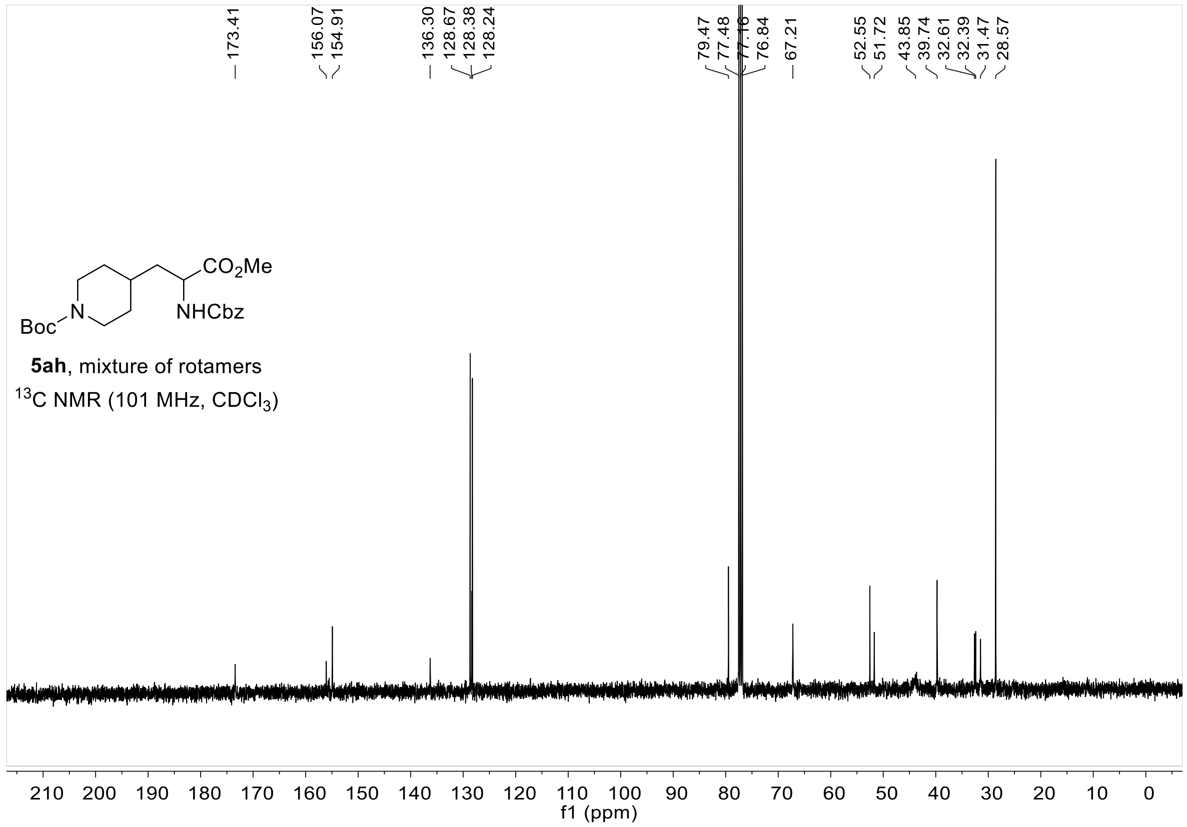

Supplementary Figure 114. ^{13}C NMR spectra of compound **5ah** (101 MHz, $CDCl_3$)

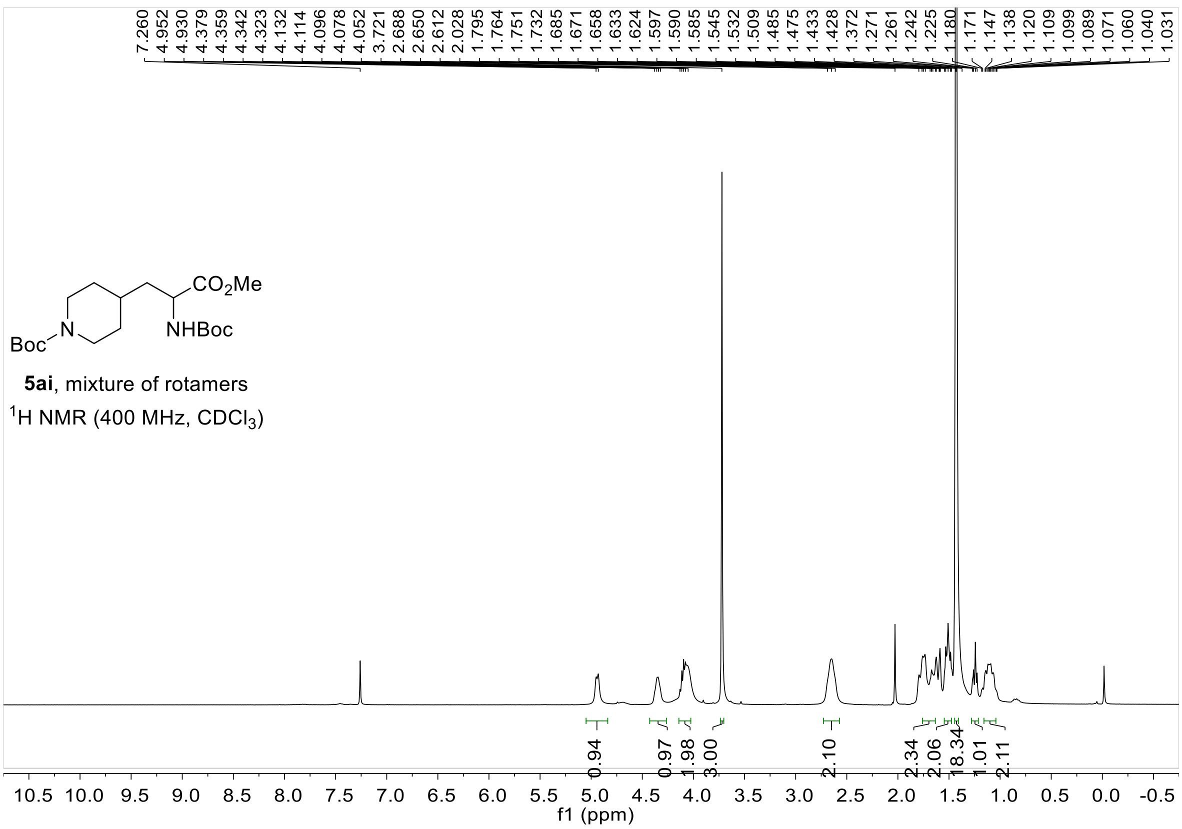

Supplementary Figure 115. ^{1}H NMR spectra of compound **5ai** (400 MHz, $CDCl_3$)

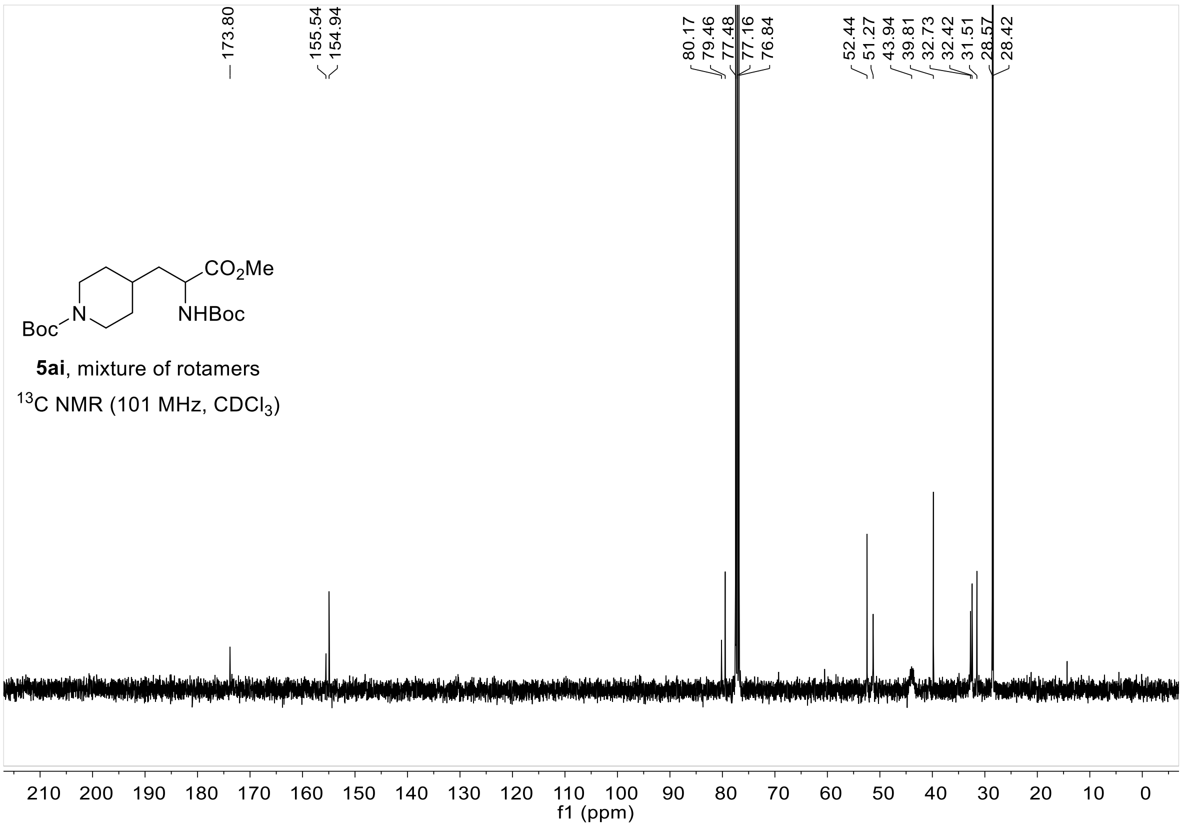

Supplementary Figure 116. ^{13}C NMR spectra of compound **5ai** (101 MHz, $CDCl_3$)

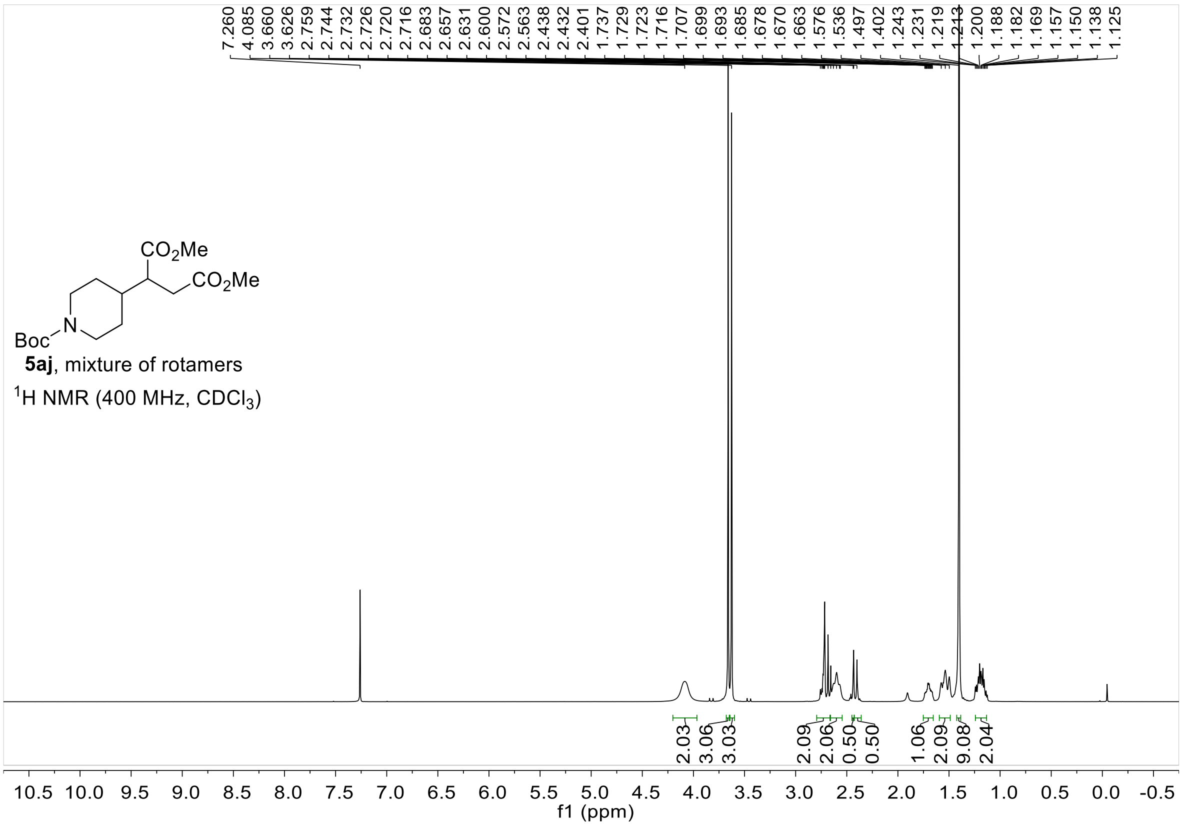

Supplementary Figure 117. ^{1}H NMR spectra of compound **5aj** (400 MHz, $CDCl_3$)

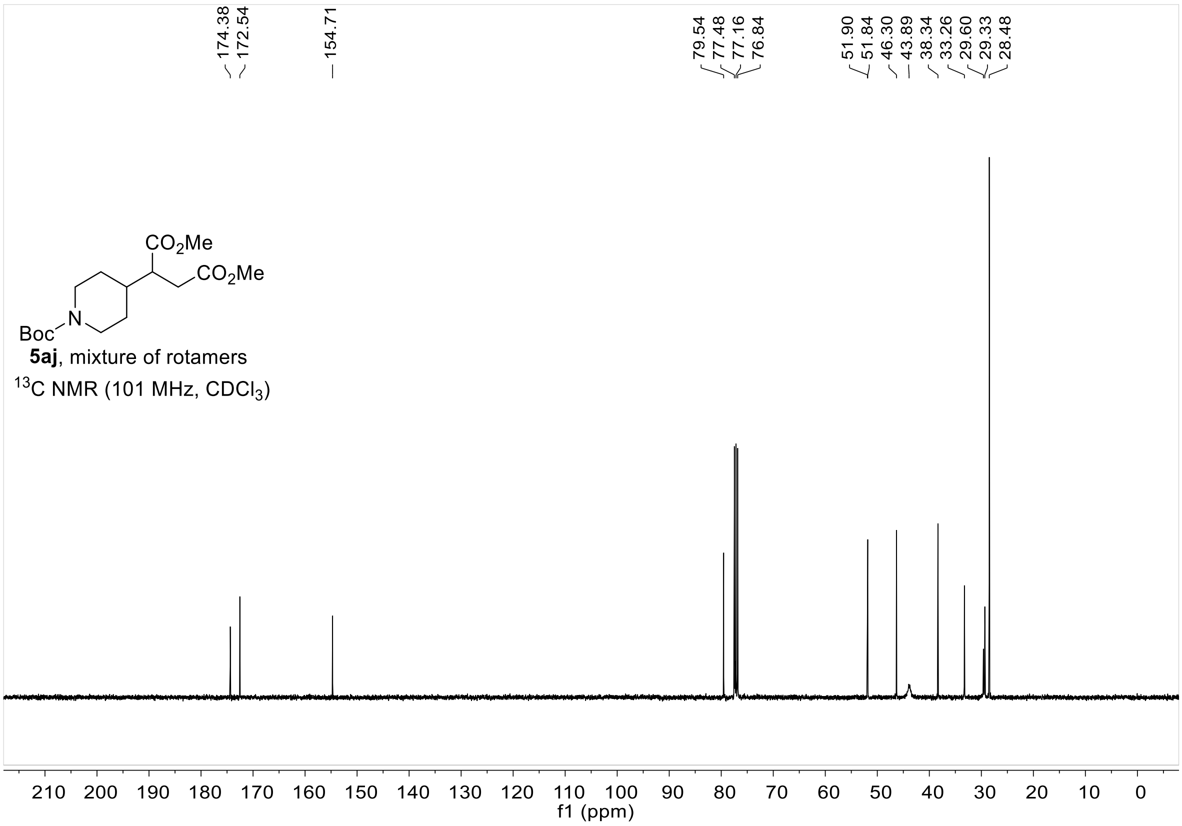

Supplementary Figure 118. ^{13}C NMR spectra of compound **5aj** (101 MHz, $CDCl_3$)

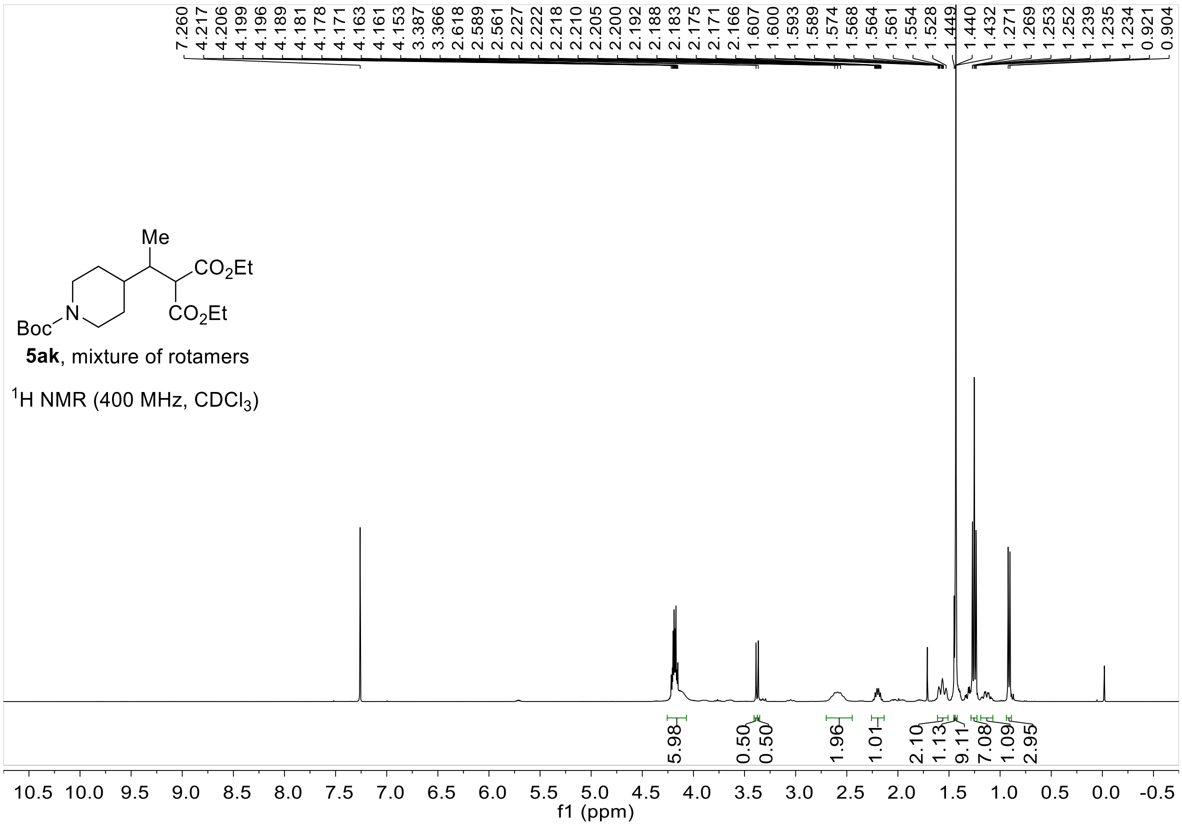

Supplementary Figure 119. ^1H NMR spectra of compound **5ak** (400 MHz, $CDCl_3$)

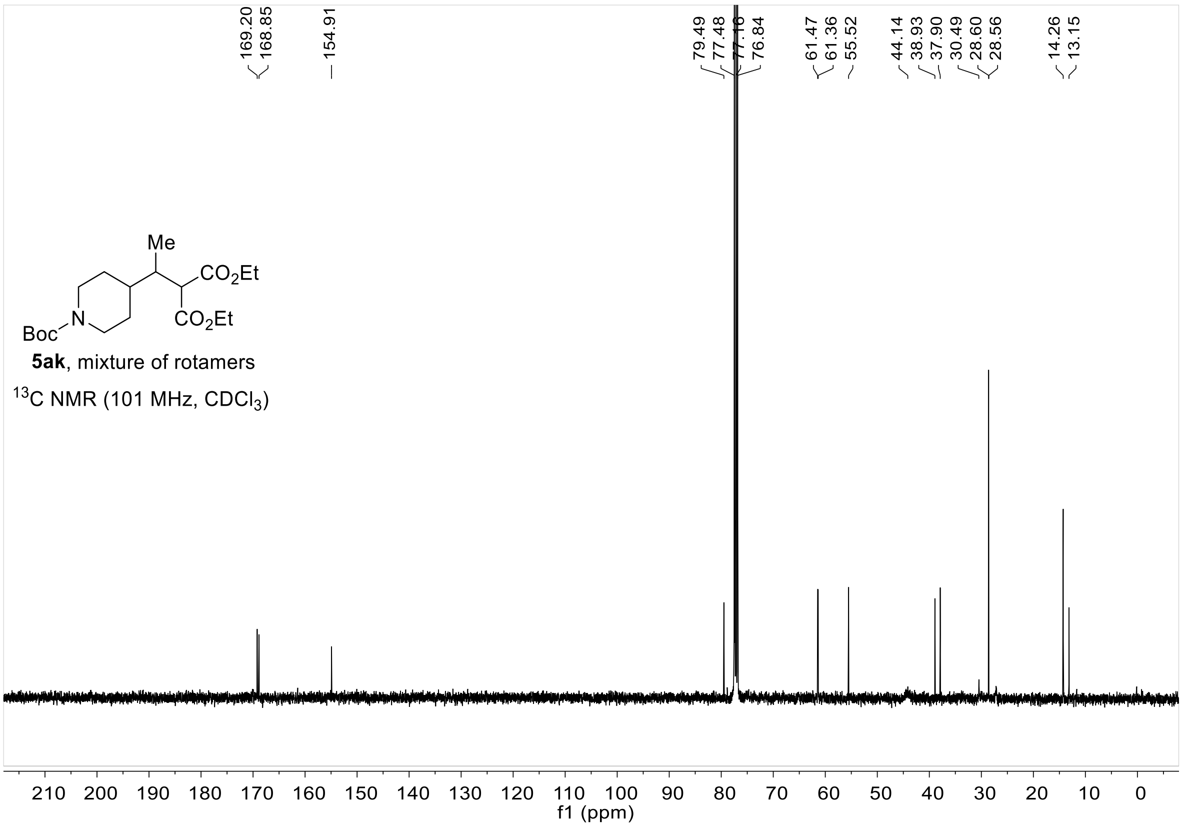

Supplementary Figure 120. ^{13}C NMR spectra of compound **5ak** (101 MHz, $CDCl_3$)

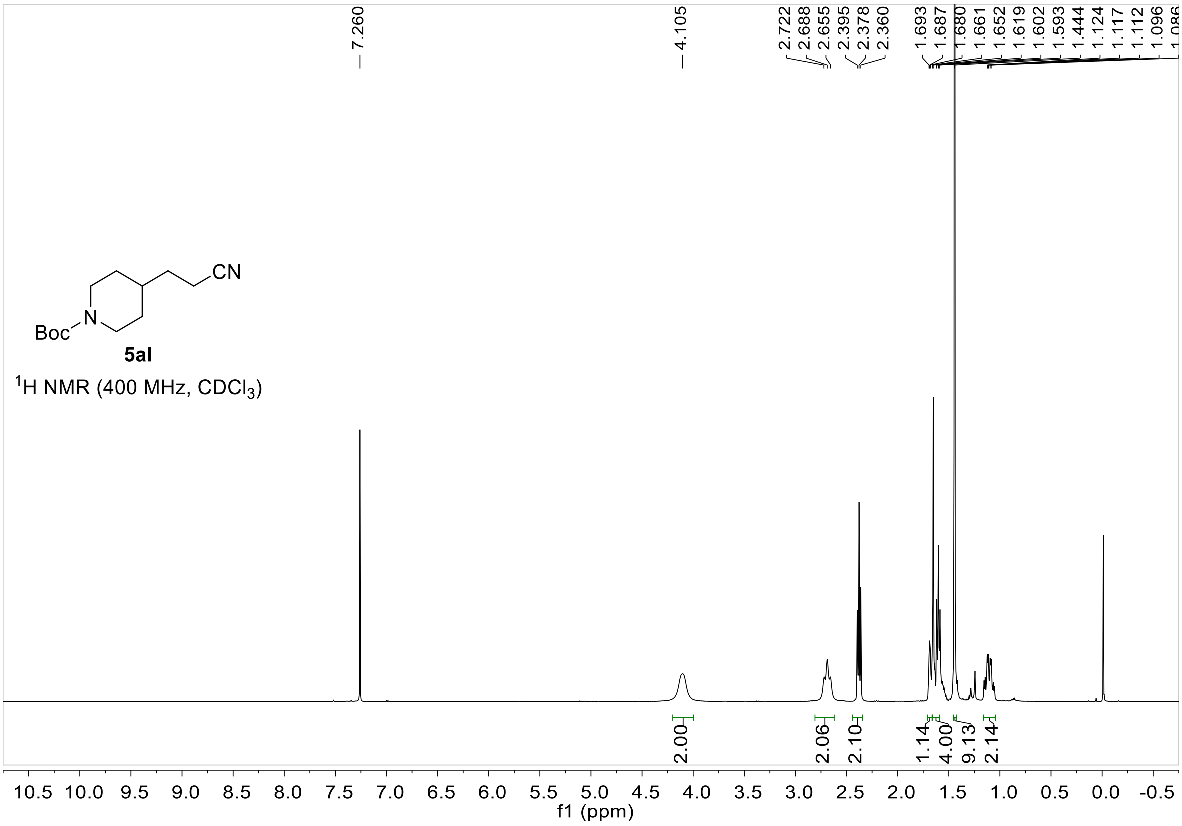

Supplementary Figure 121. ^{1}H NMR spectra of compound **5al** (400 MHz, $CDCl_3$)

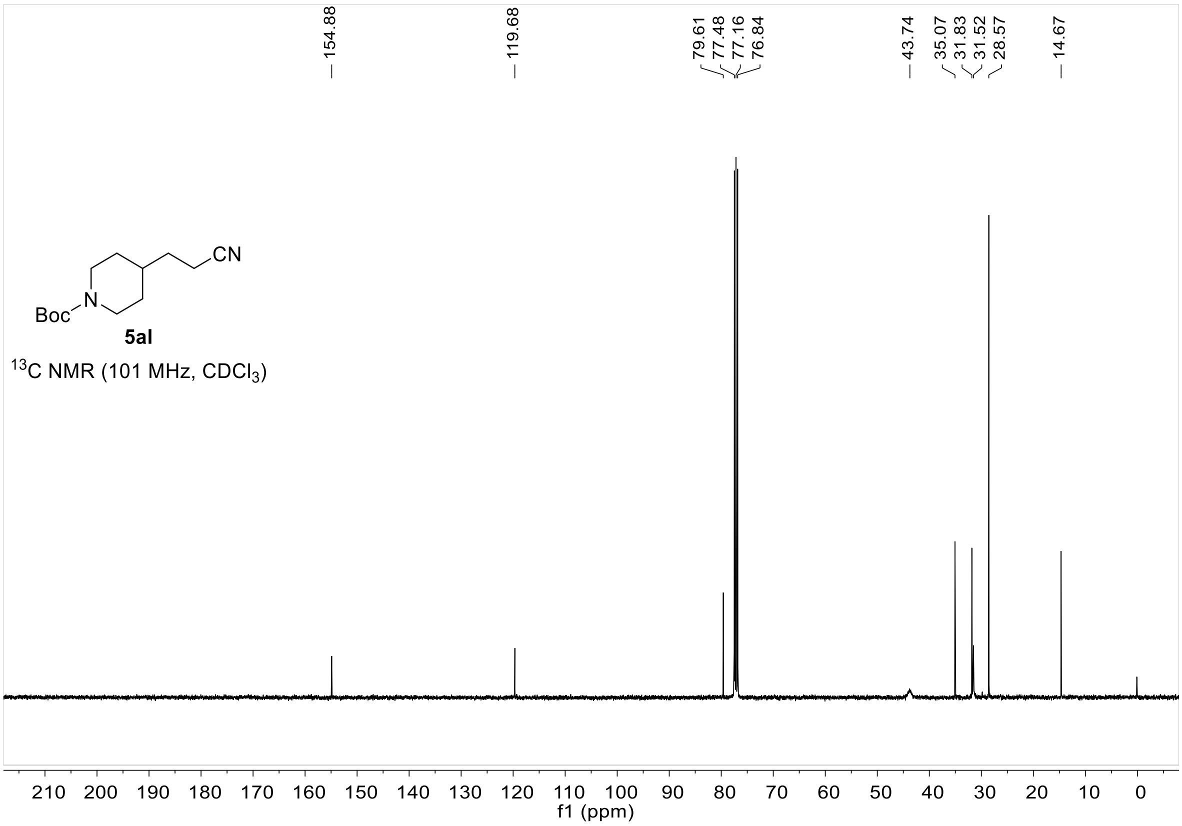

Supplementary Figure 122. ^{13}C NMR spectra of compound **5al** (101 MHz, $CDCl_3$)

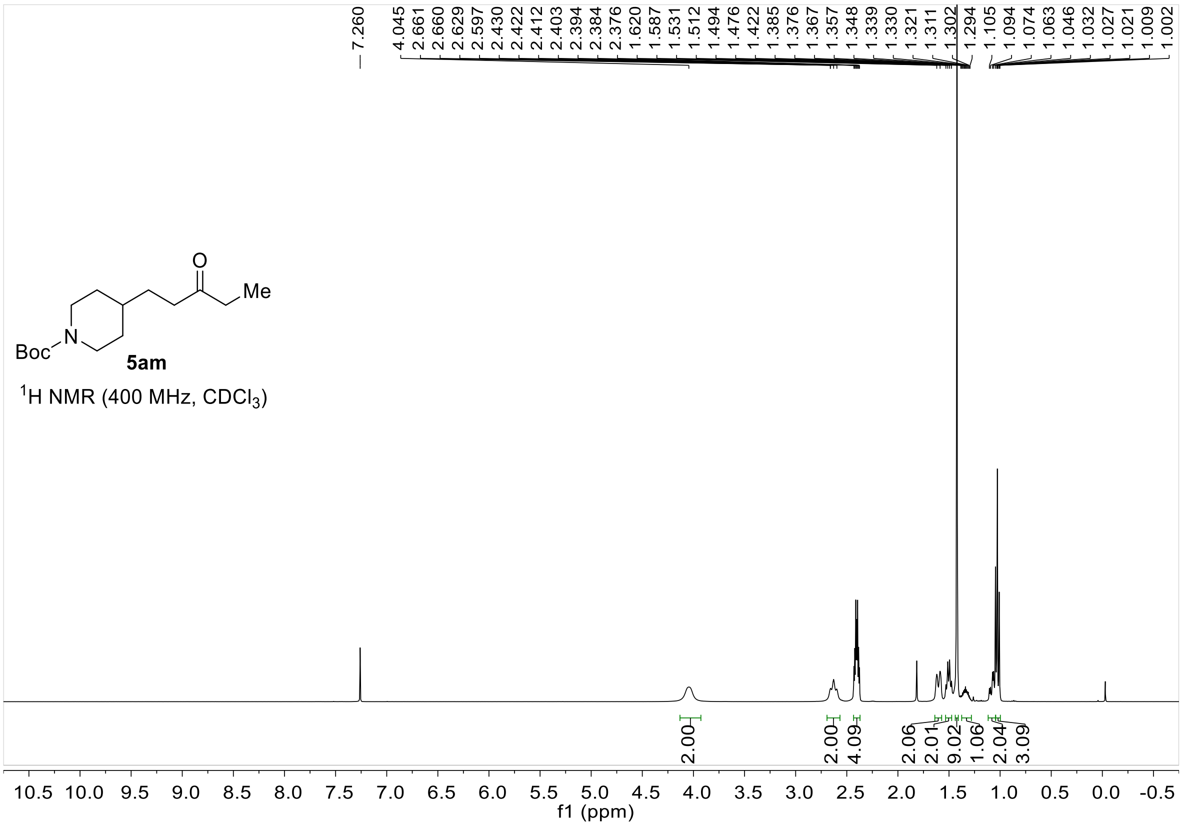

Supplementary Figure 123. ^{1}H NMR spectra of compound **5am** (400 MHz, $CDCl_3$)

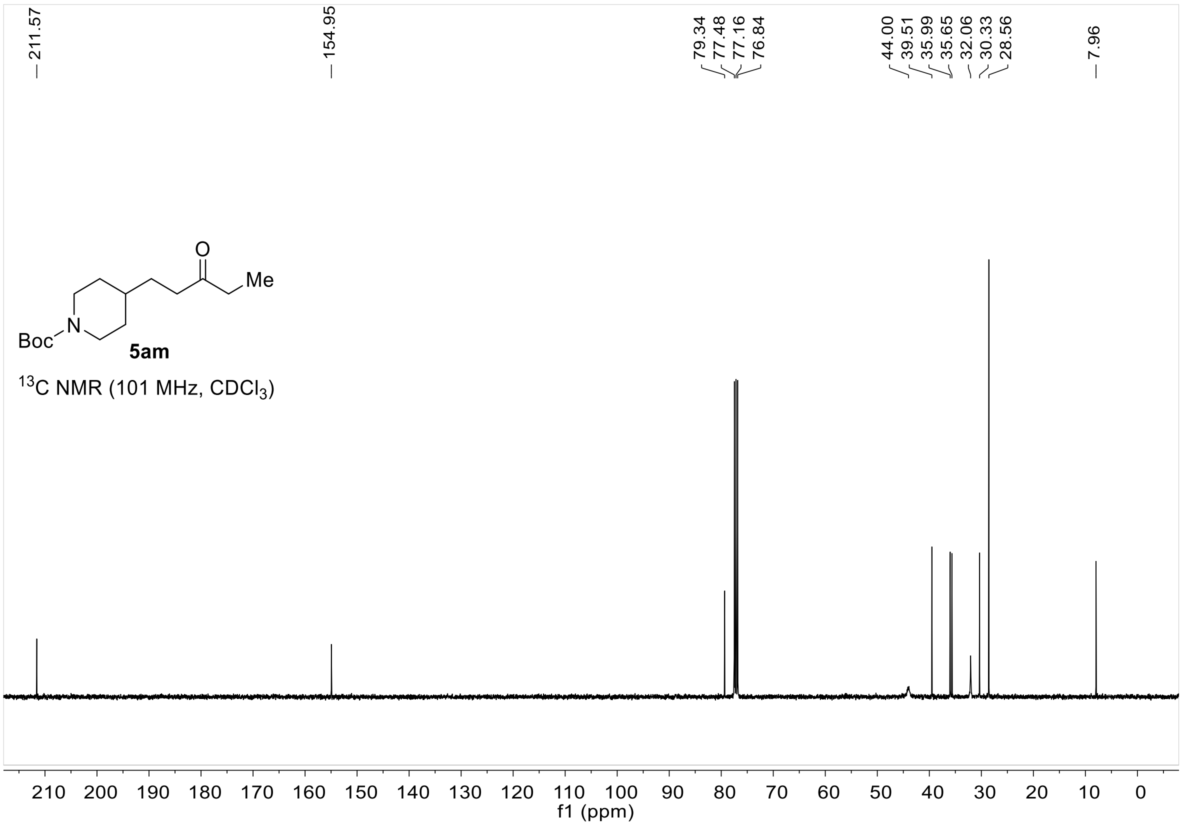

Supplementary Figure 124. ^{13}C NMR spectra of compound **5am** (101 MHz, $CDCl_3$)

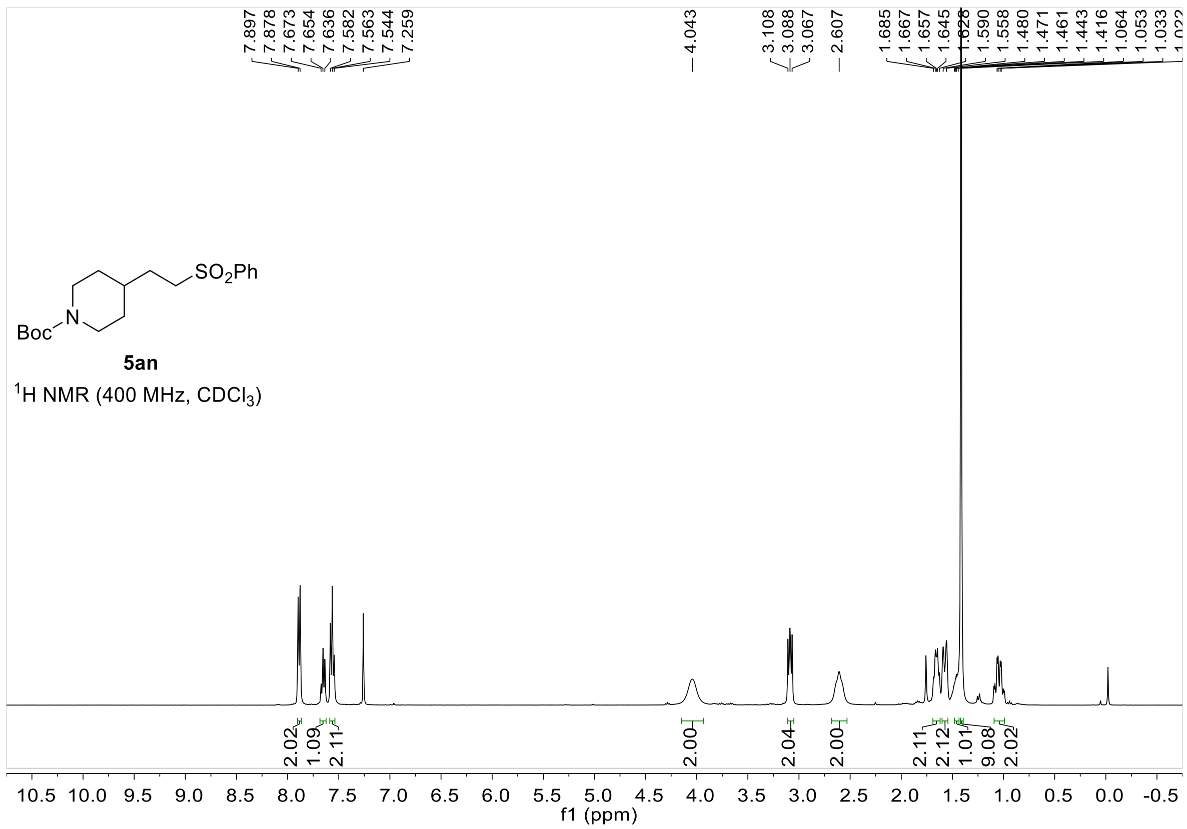

Supplementary Figure 125. ^{1}H NMR spectra of compound **5an** (400 MHz, $CDCl_3$)

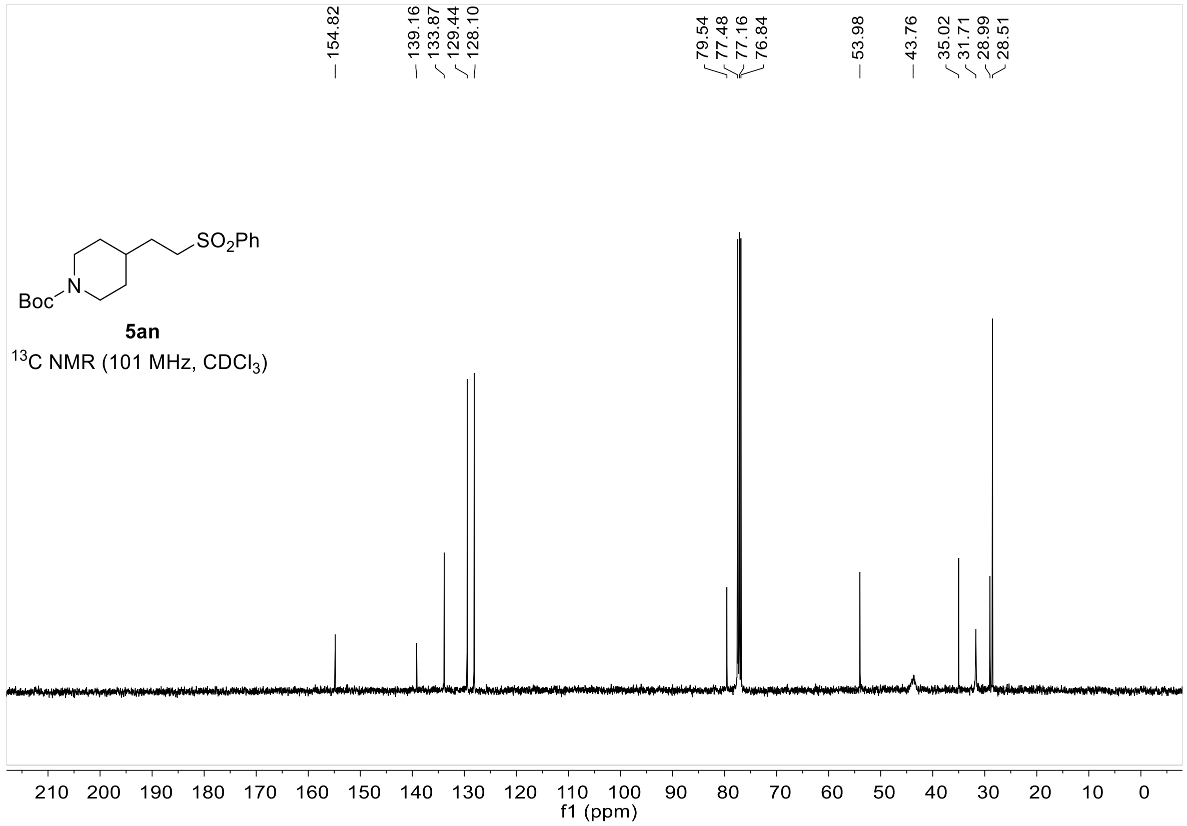

Supplementary Figure 126. ^{13}C NMR spectra of compound **5an** (101 MHz, $CDCl_3$)

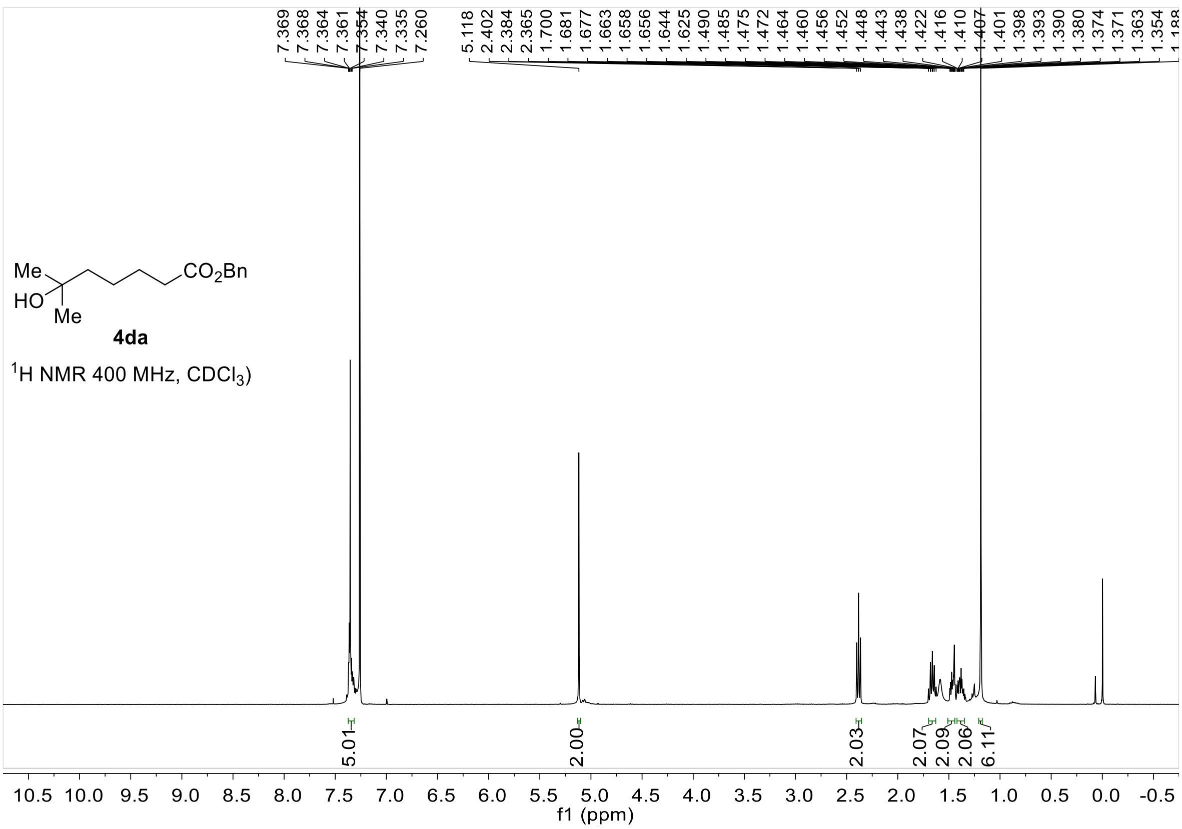

Supplementary Figure 127. ^{1}H NMR spectra of compound **4da** (400 MHz, $CDCl_3$)

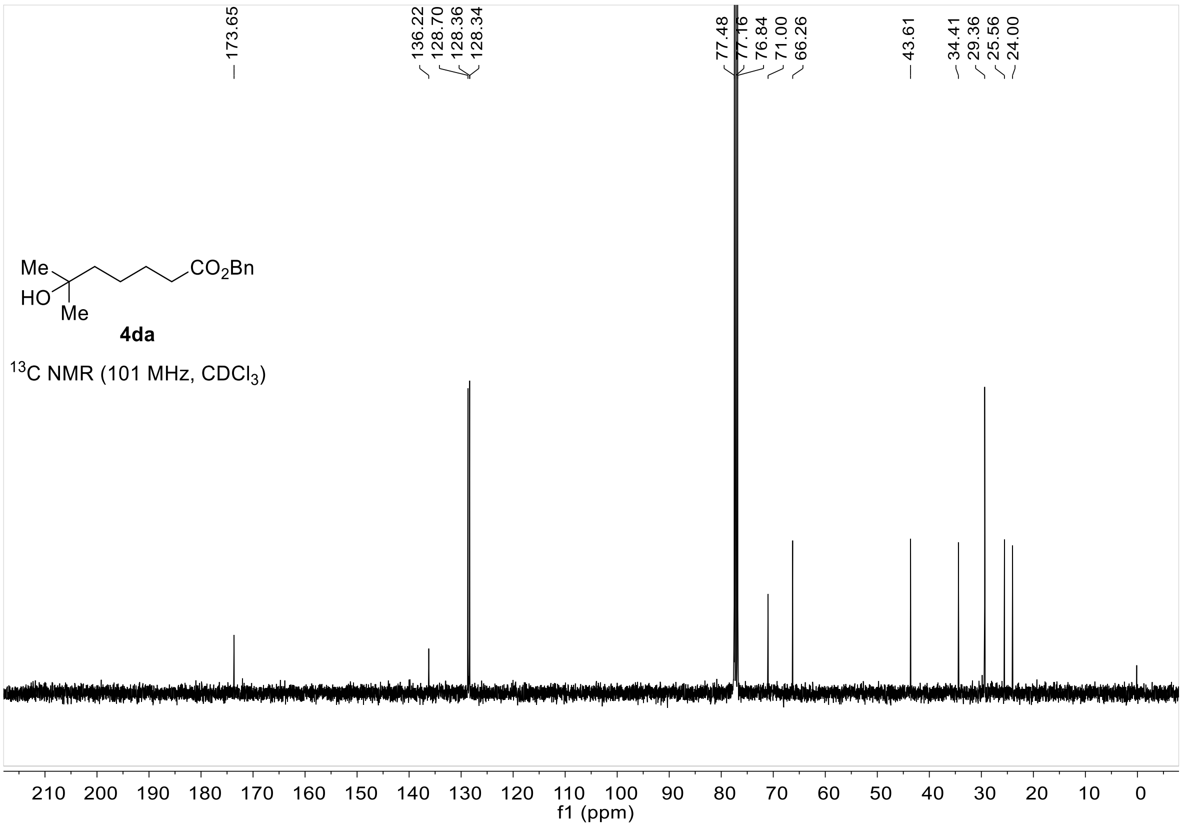

Supplementary Figure 128. ^{13}C NMR spectra of compound **4da** (101 MHz, $CDCl_3$)

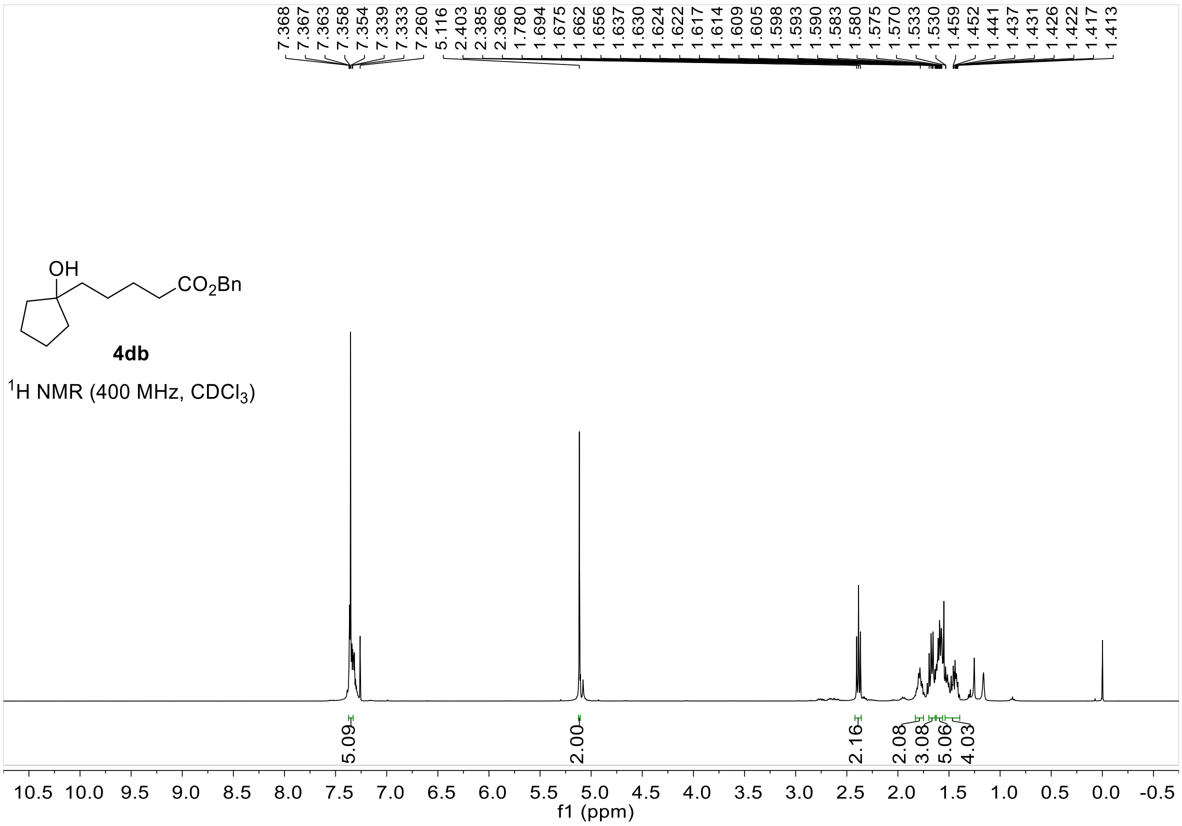

Supplementary Figure 129. ^{1}H NMR spectra of compound **4db** (400 MHz, $CDCl_3$)

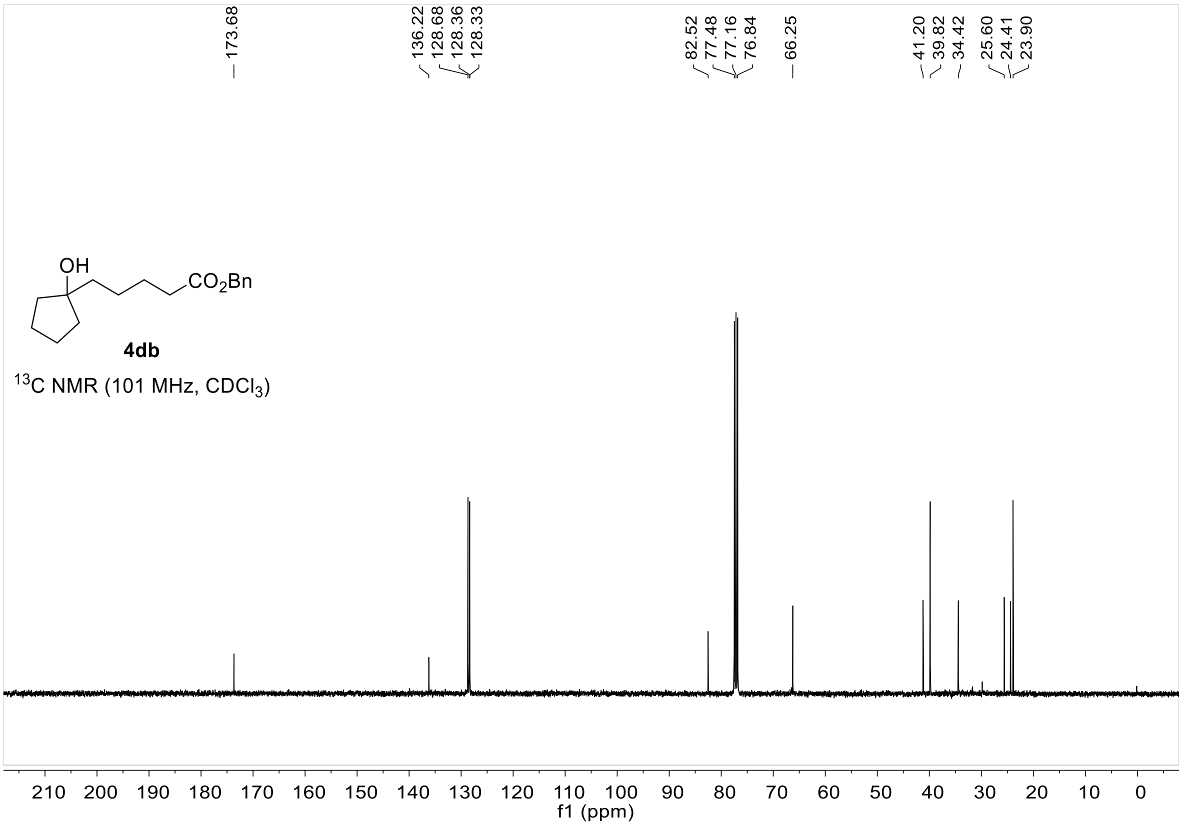

Supplementary Figure 130. ^{13}C NMR spectra of compound **4db** (101 MHz, $CDCl_3$)

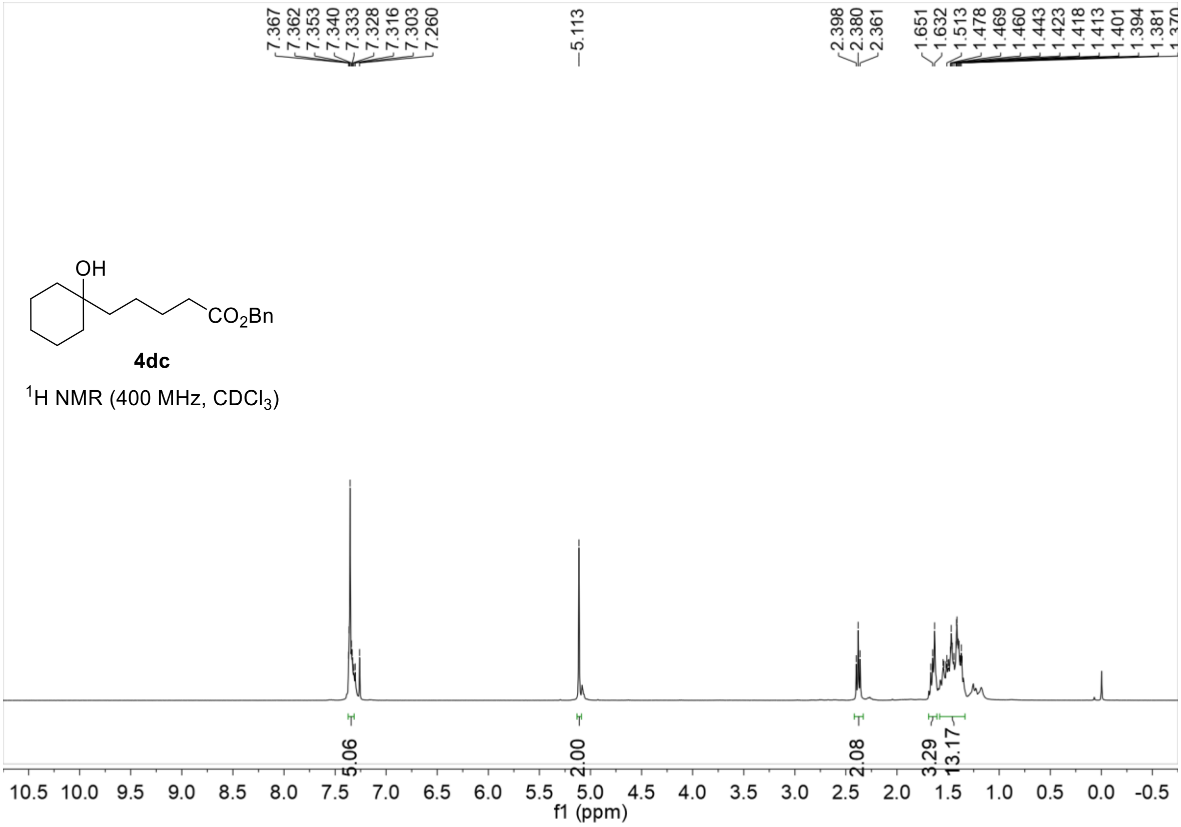

Supplementary Figure 131. ^{1}H NMR spectra of compound **4dc** (400 MHz, $CDCl_3$)

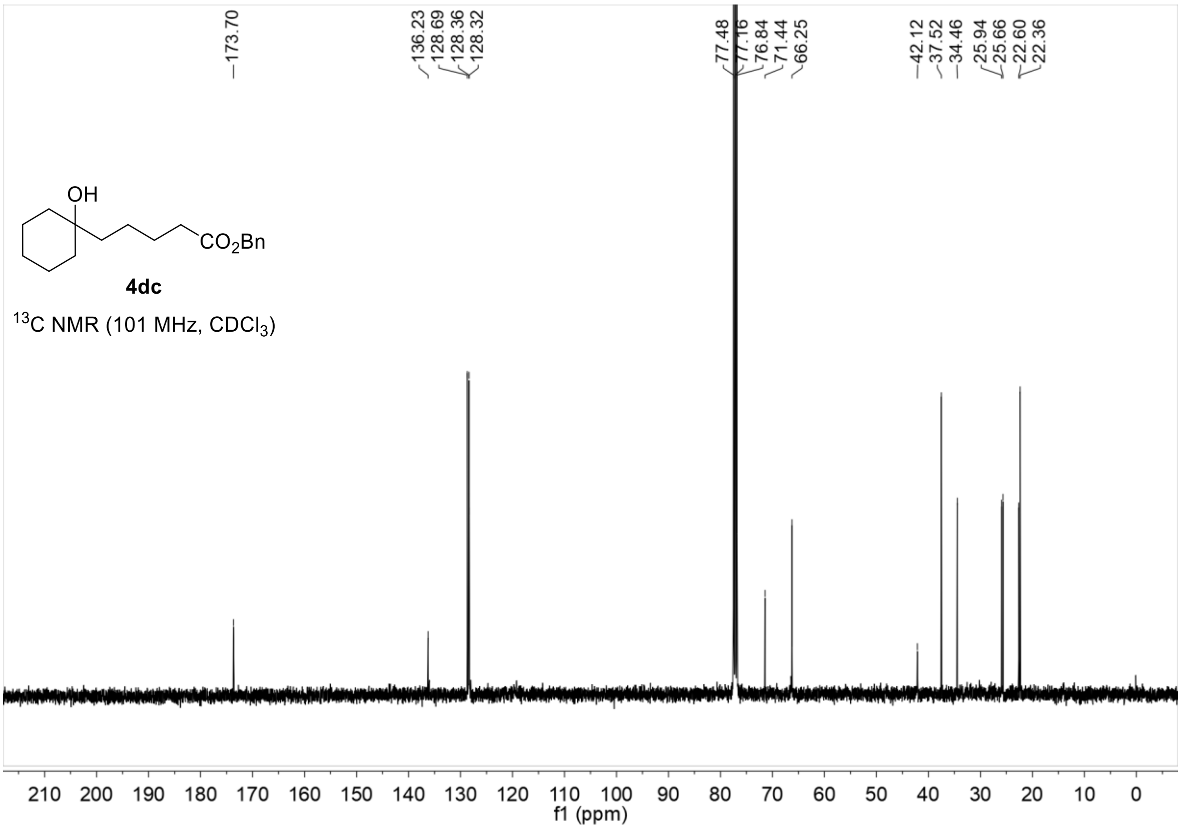

Supplementary Figure 132. ^{13}C NMR spectra of compound **4dc** (101 MHz, $CDCl_3$)

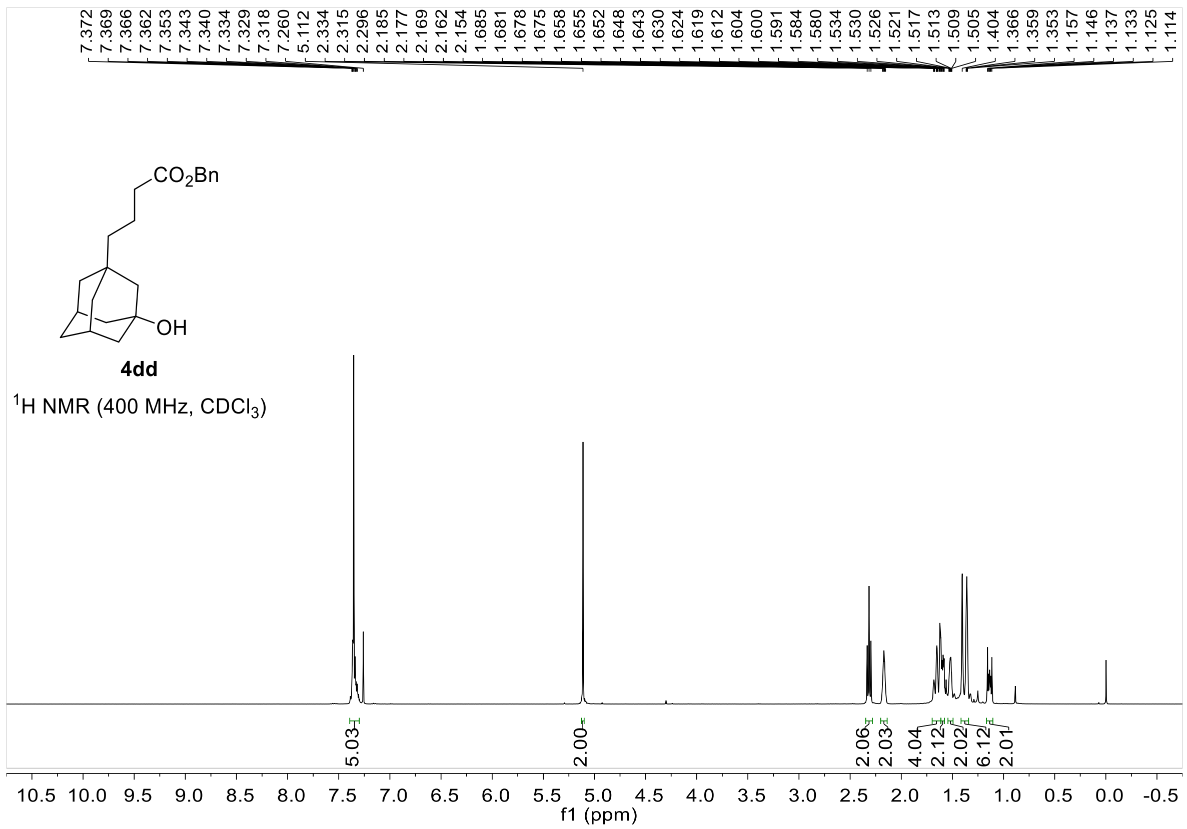

Supplementary Figure 133. ^{1}H NMR spectra of compound **4dd** (400 MHz, $CDCl_3$)

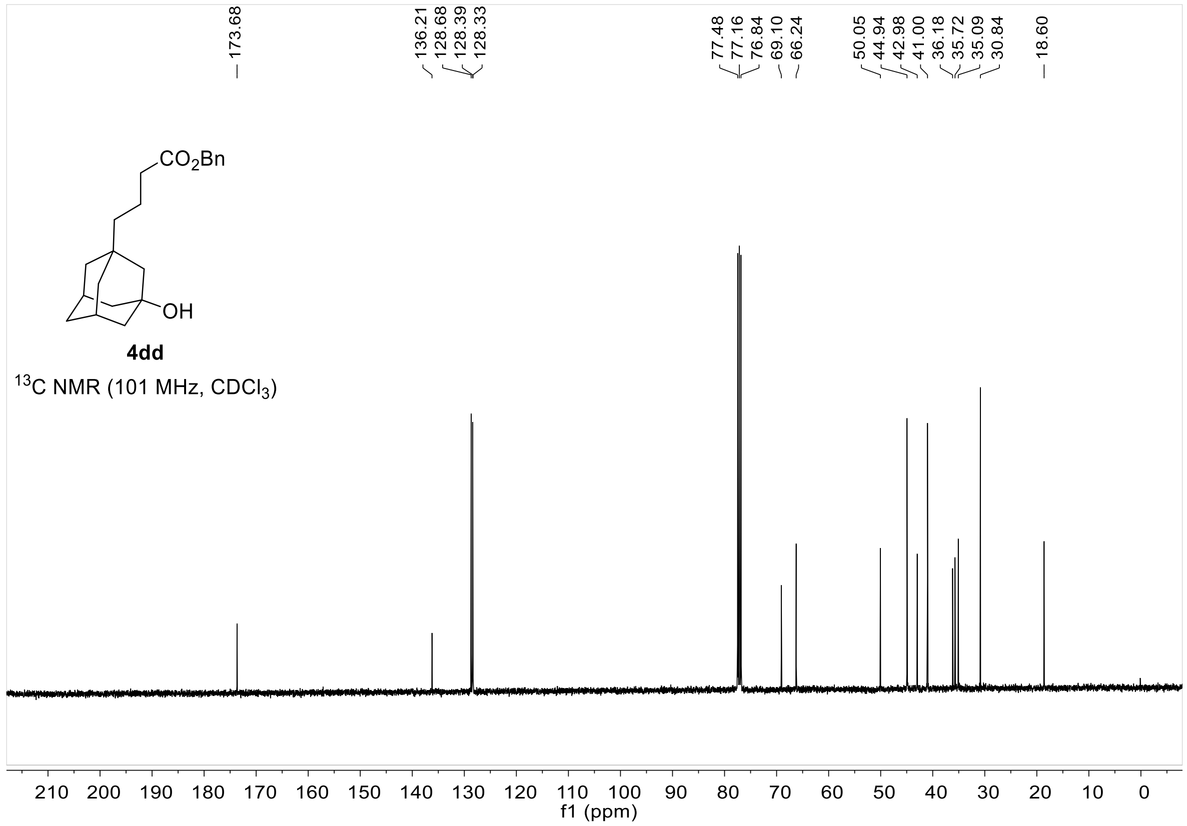

Supplementary Figure 134. ^{13}C NMR spectra of compound **4dd** (101 MHz, $CDCl_3$)

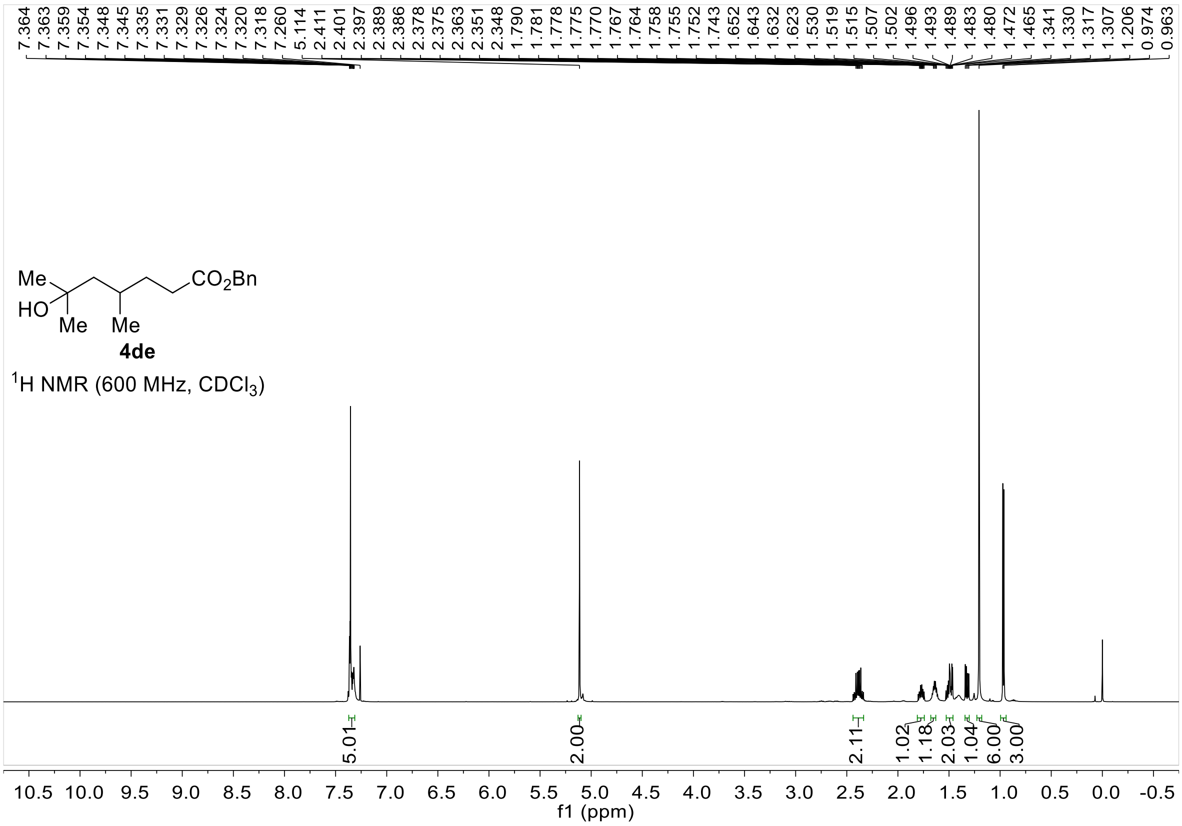

Supplementary Figure 135. ^{1}H NMR spectra of compound **4de** (600 MHz, $CDCl_3$)

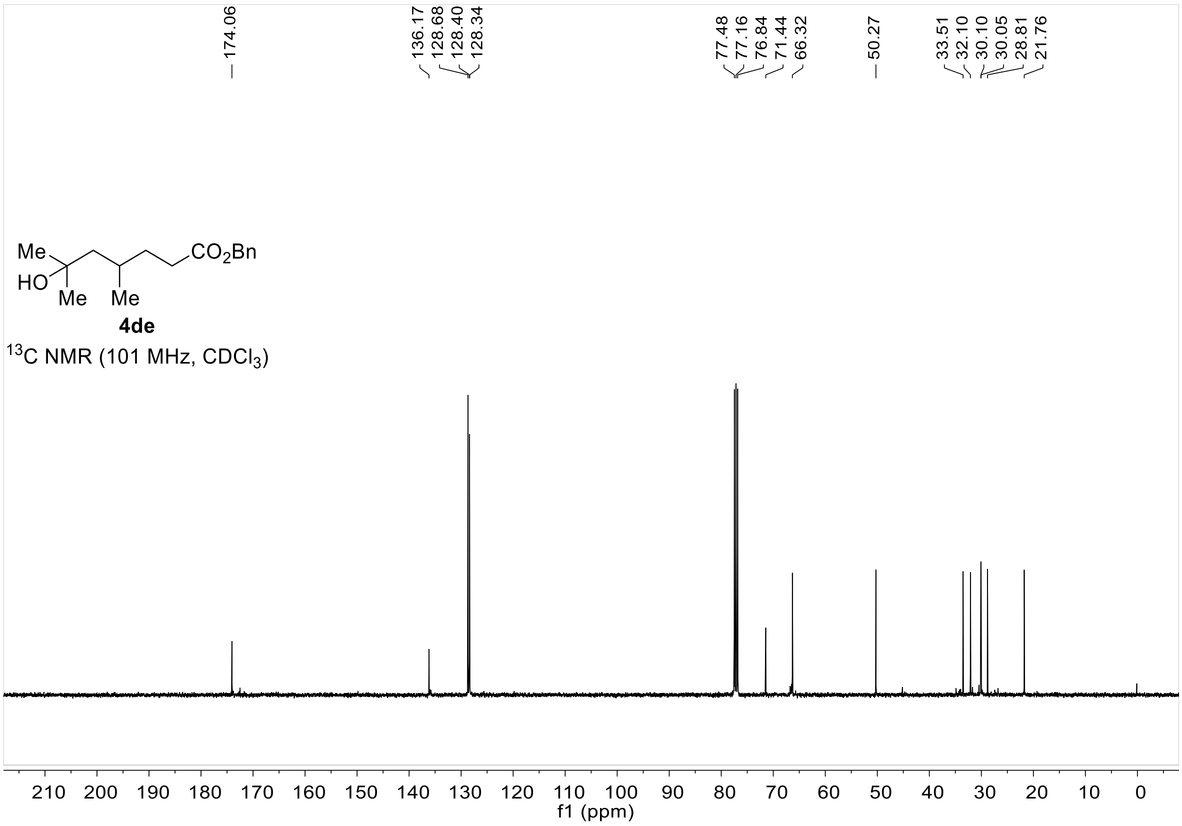

Supplementary Figure 136. ^{13}C NMR spectra of compound **4de** (101 MHz, $CDCl_3$)

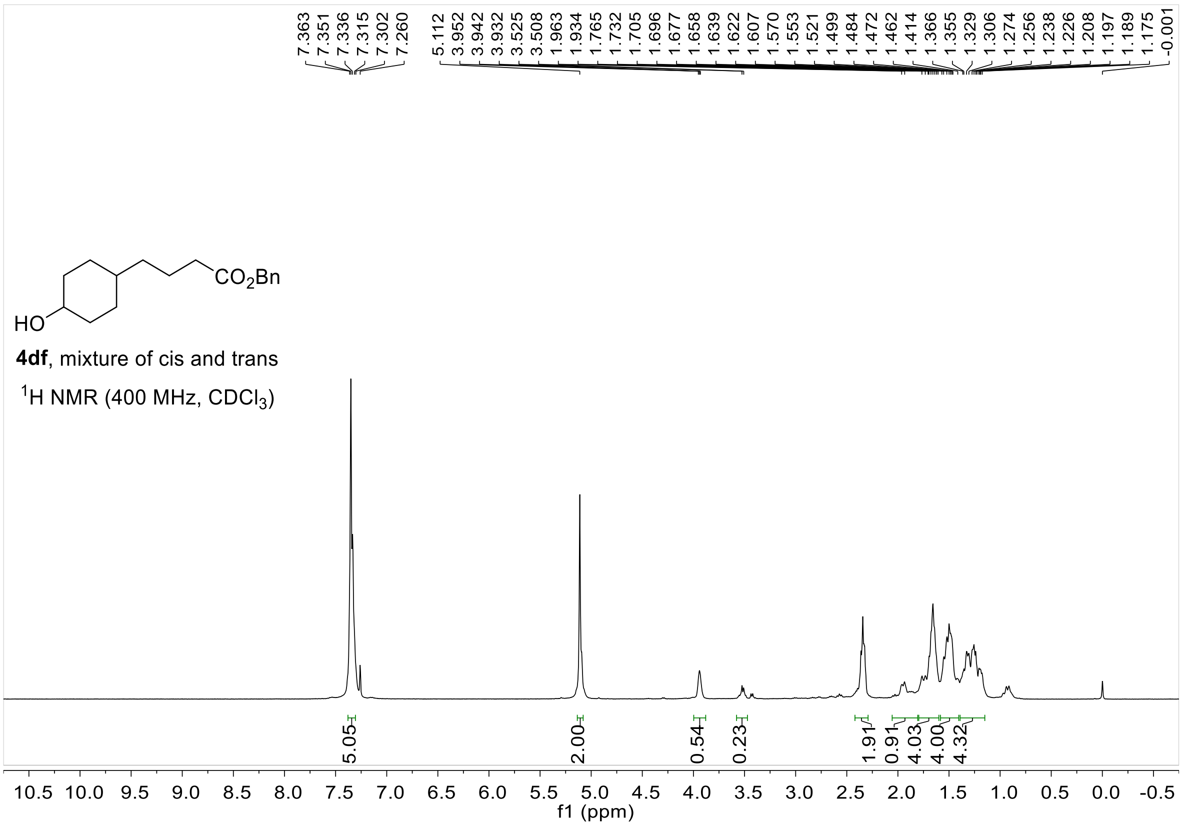

Supplementary Figure 137. ^{1}H NMR spectra of compound **4df** (400 MHz, $CDCl_3$)

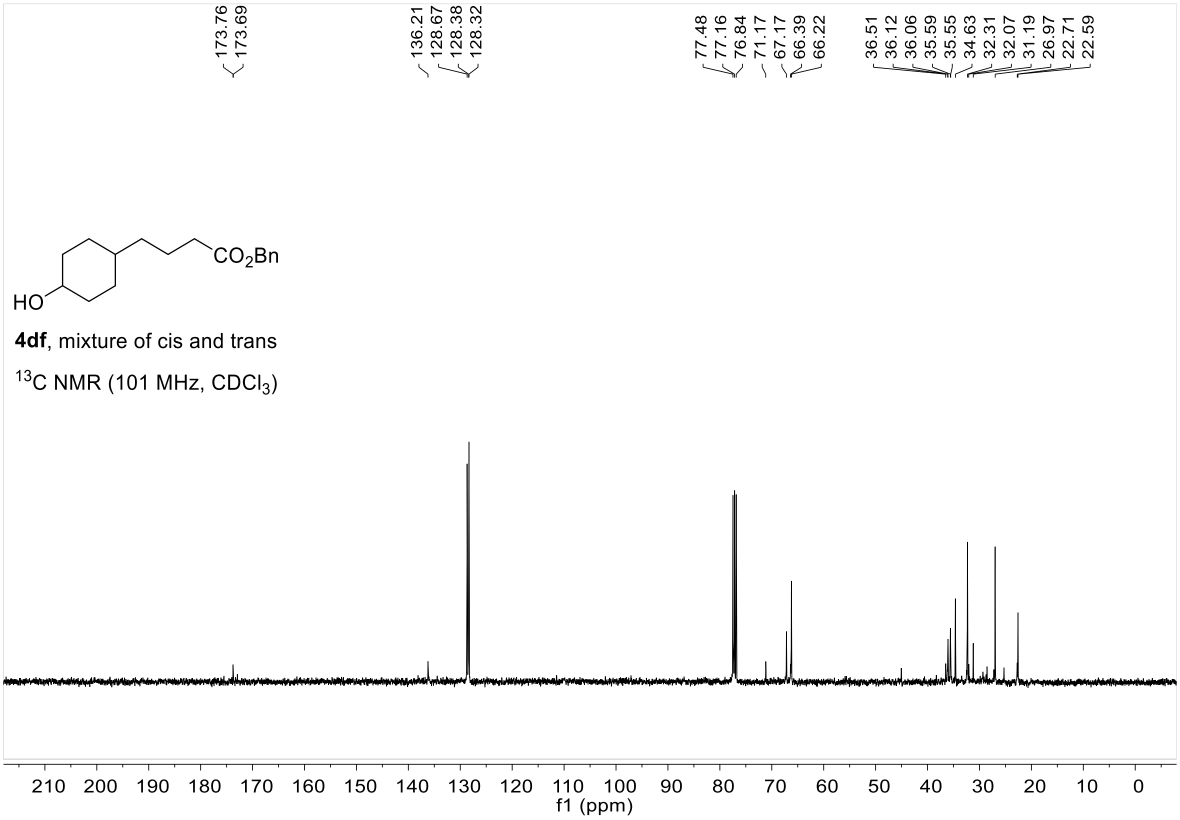

Supplementary Figure 138. ^{13}C NMR spectra of compound **4df** (101 MHz, $CDCl_3$)

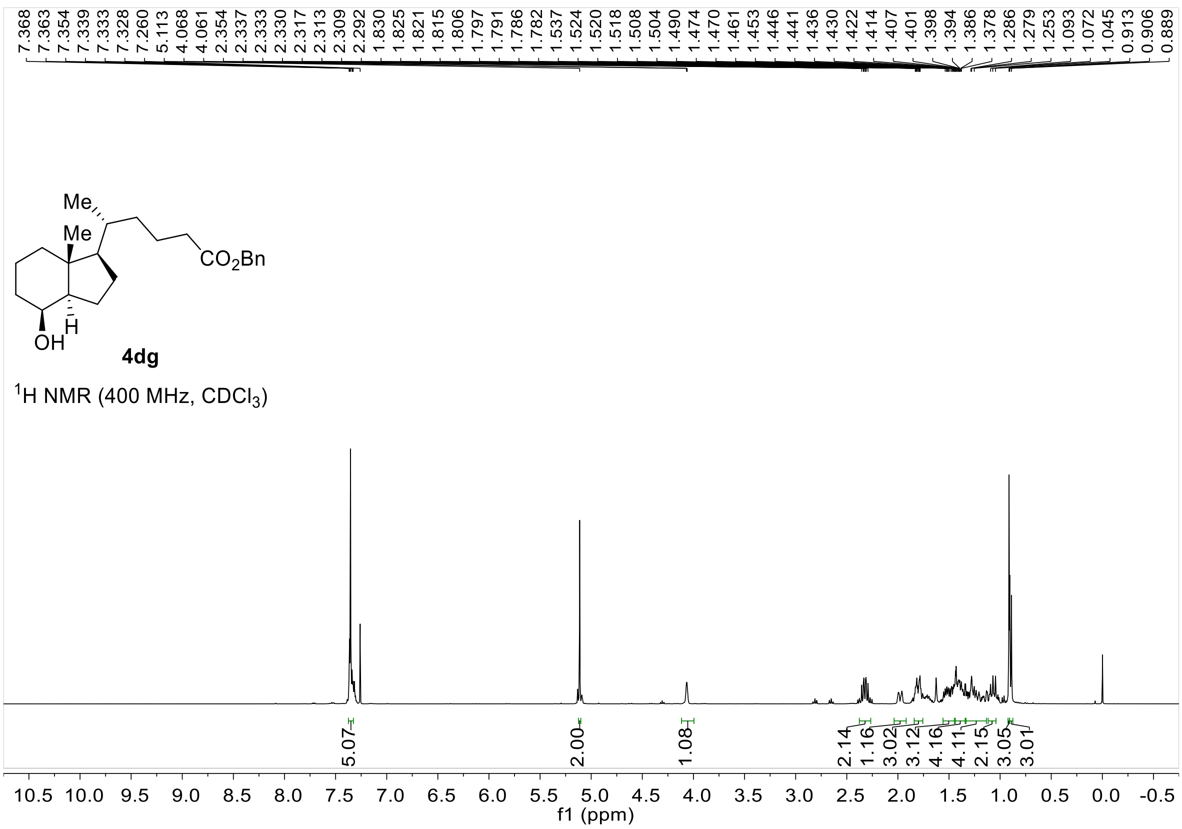

Supplementary Figure 139. ^{1}H NMR spectra of compound **4dg** (400 MHz, $CDCl_3$)

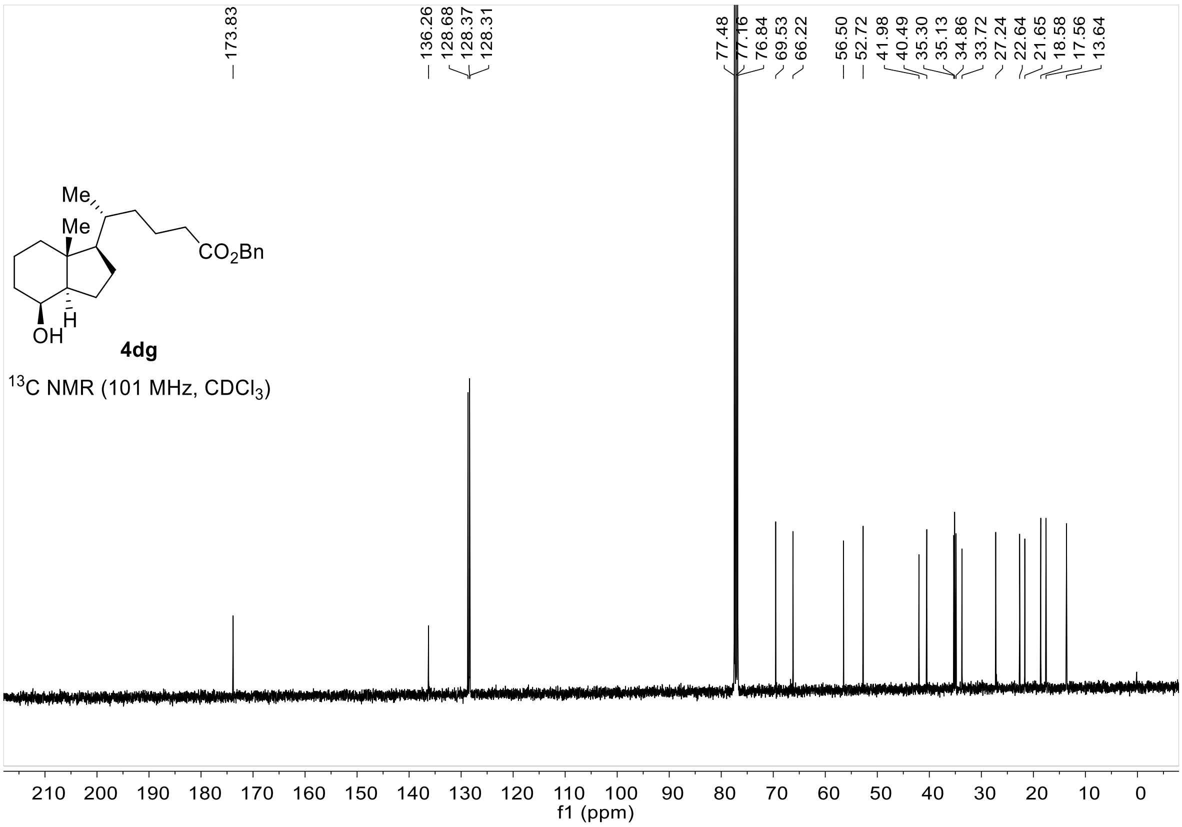

Supplementary Figure 140. ^{13}C NMR spectra of compound **4dg** (101 MHz, $CDCl_3$)

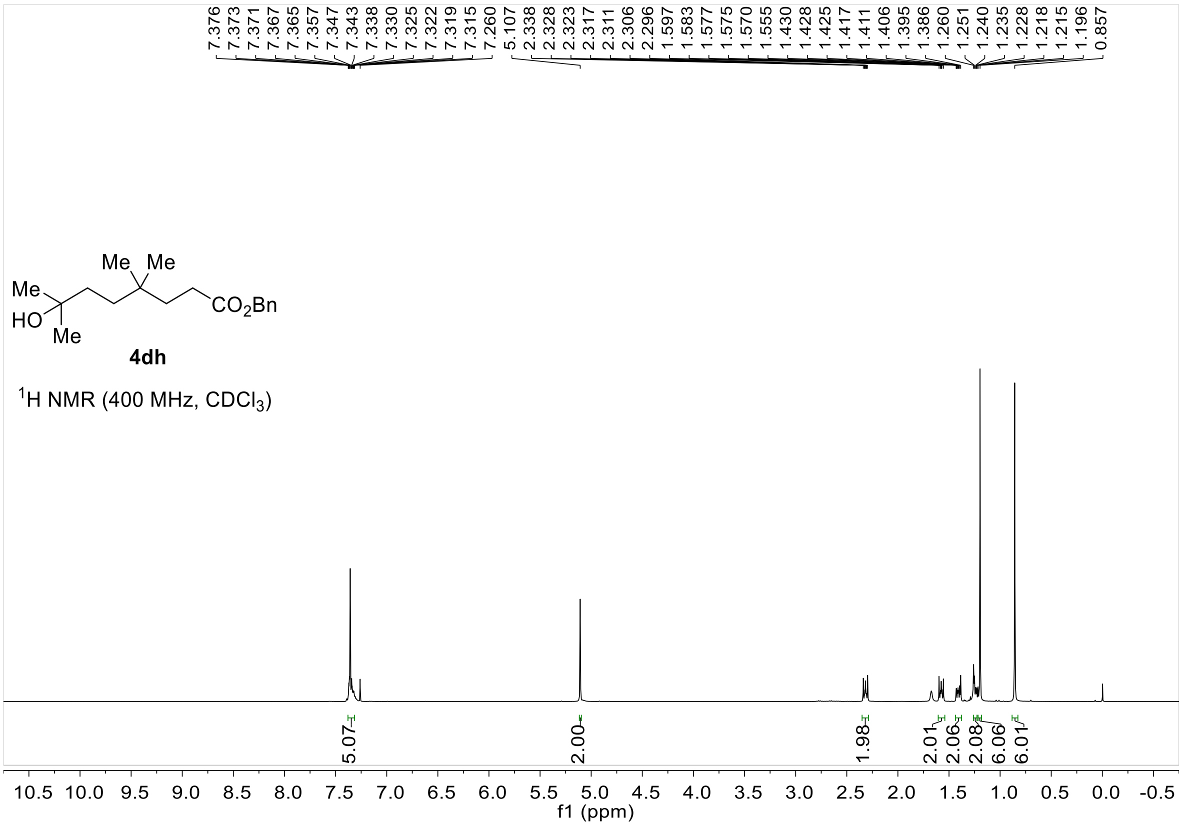

Supplementary Figure 141. ^{1}H NMR spectra of compound **4dh** (400 MHz, $CDCl_3$)

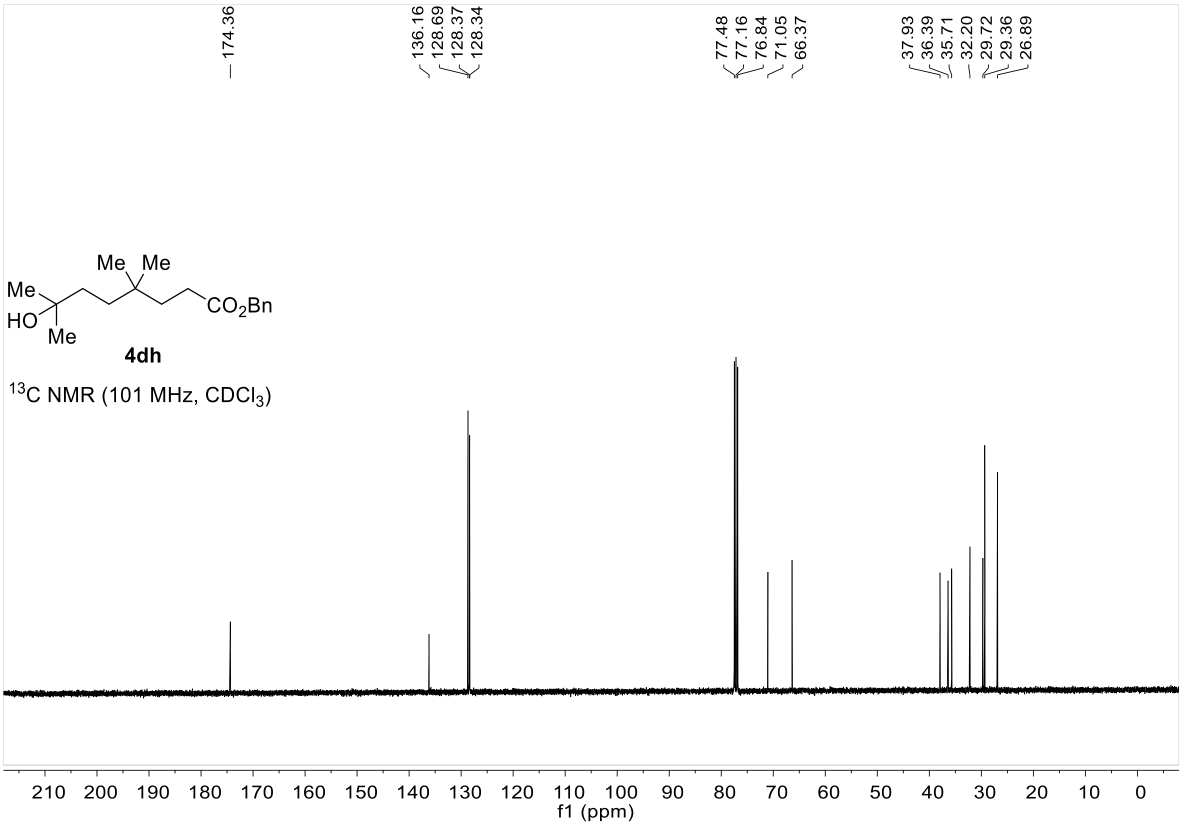

Supplementary Figure 142. ^{13}C NMR spectra of compound **4dh** (101 MHz, $CDCl_3$)

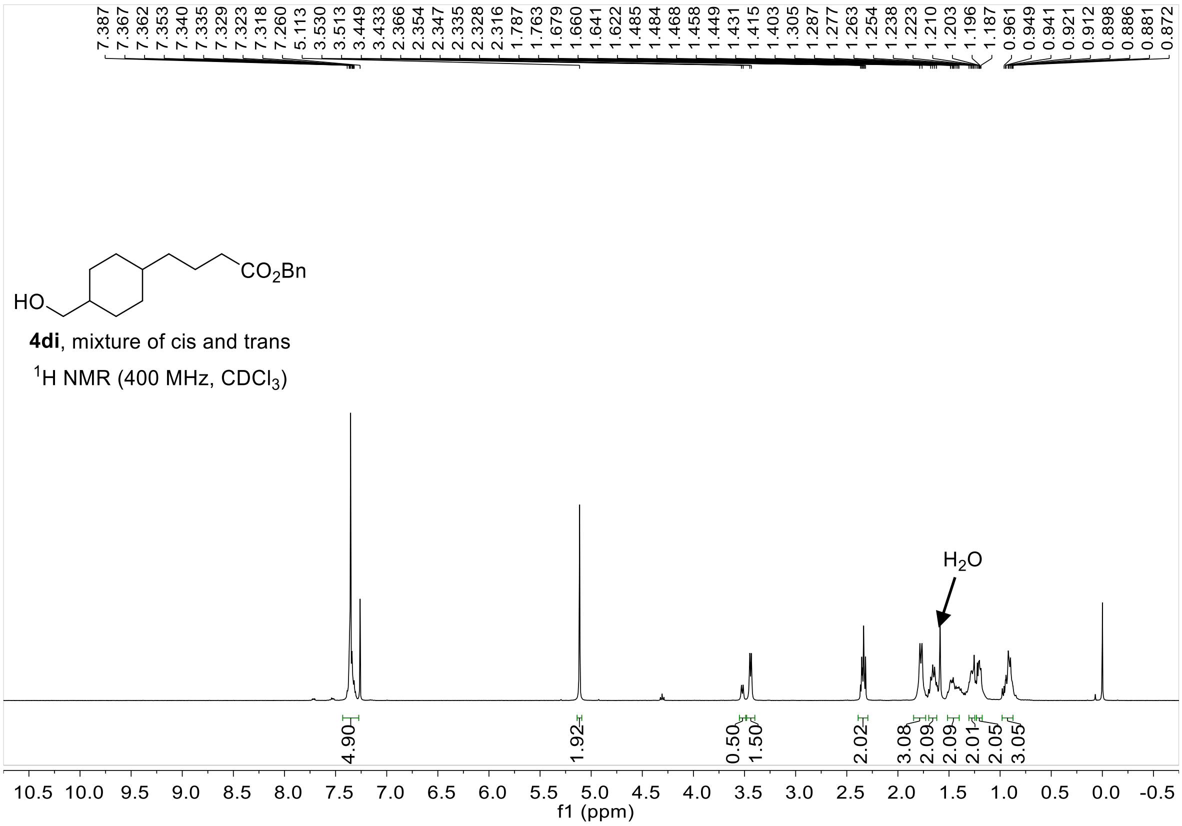

Supplementary Figure 143. ^{1}H NMR spectra of compound **4di** (400 MHz, $CDCl_3$)

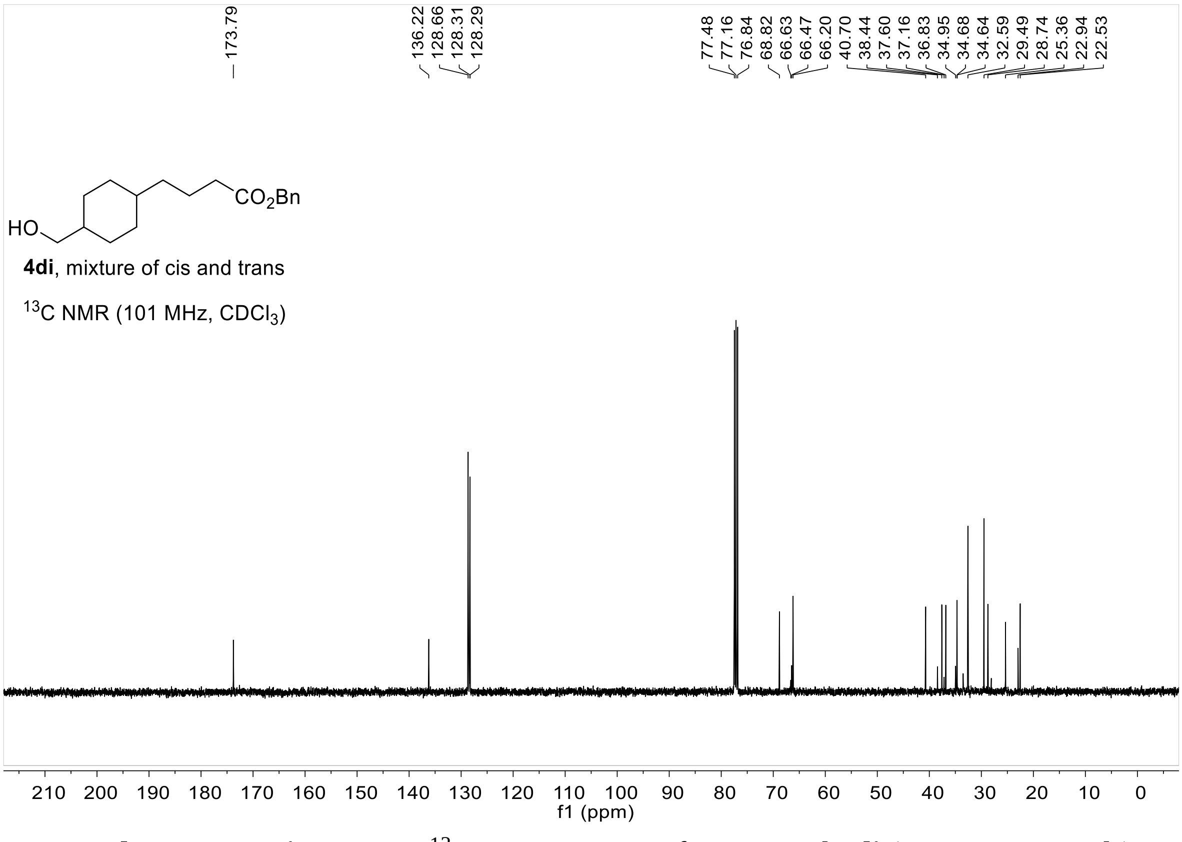

Supplementary Figure 144. ^{13}C NMR spectra of compound **4di** (101 MHz, $CDCl_3$)

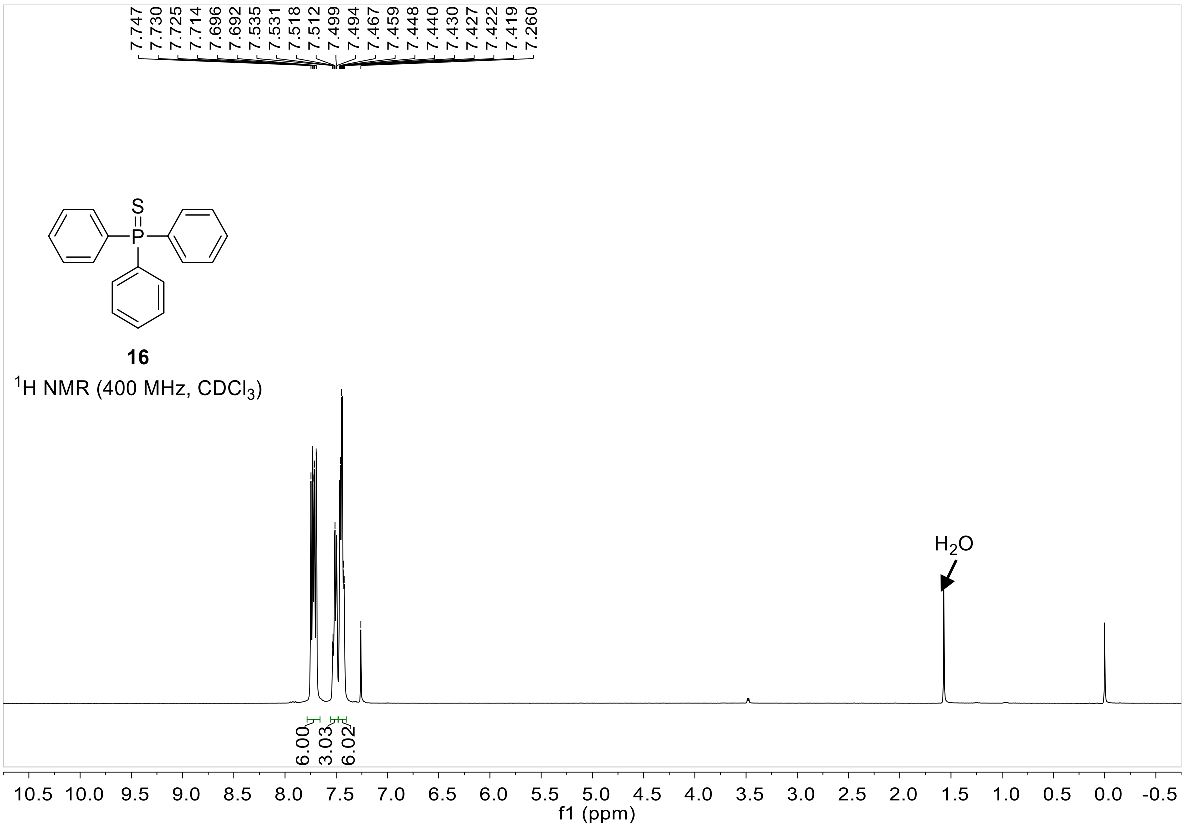

Supplementary Figure 145. ^{1}H NMR spectra of compound **16** (400 MHz, $CDCl_3$)

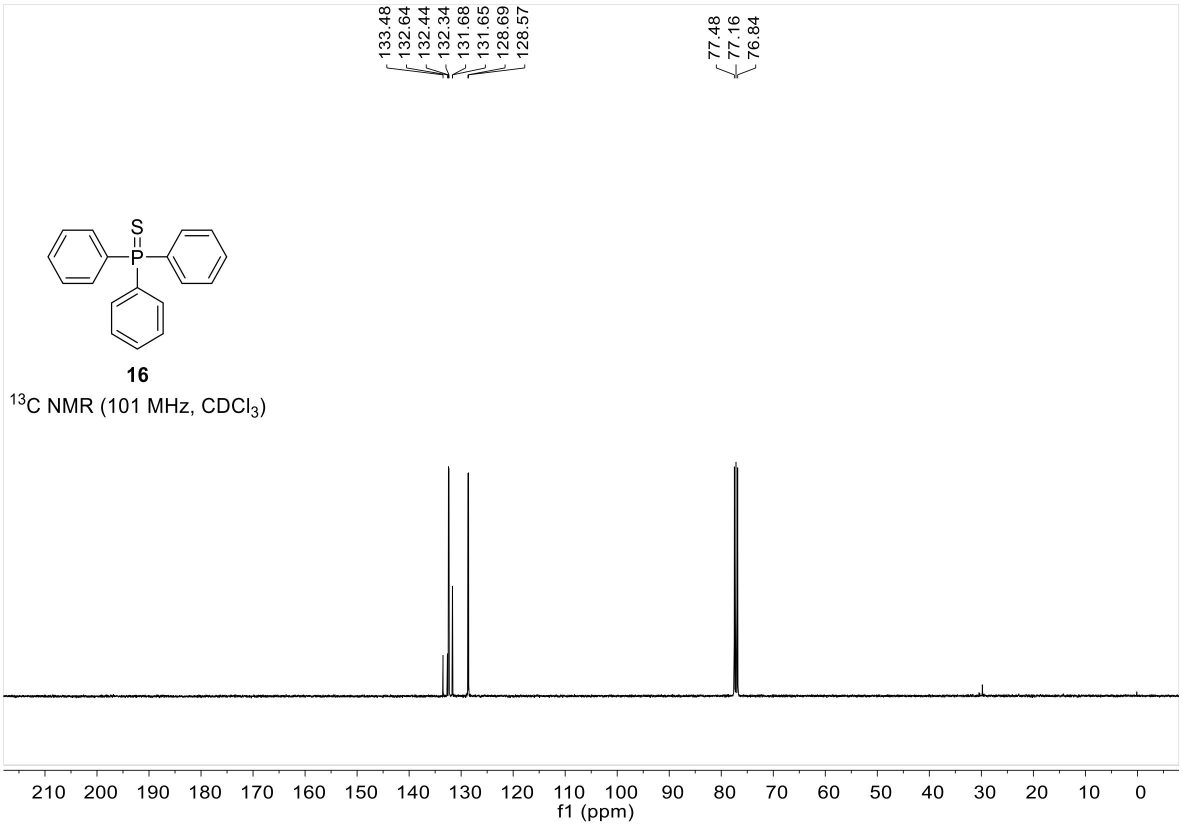

Supplementary Figure 146. ^{13}C NMR spectra of compound **16** (101 MHz, $CDCl_3$)

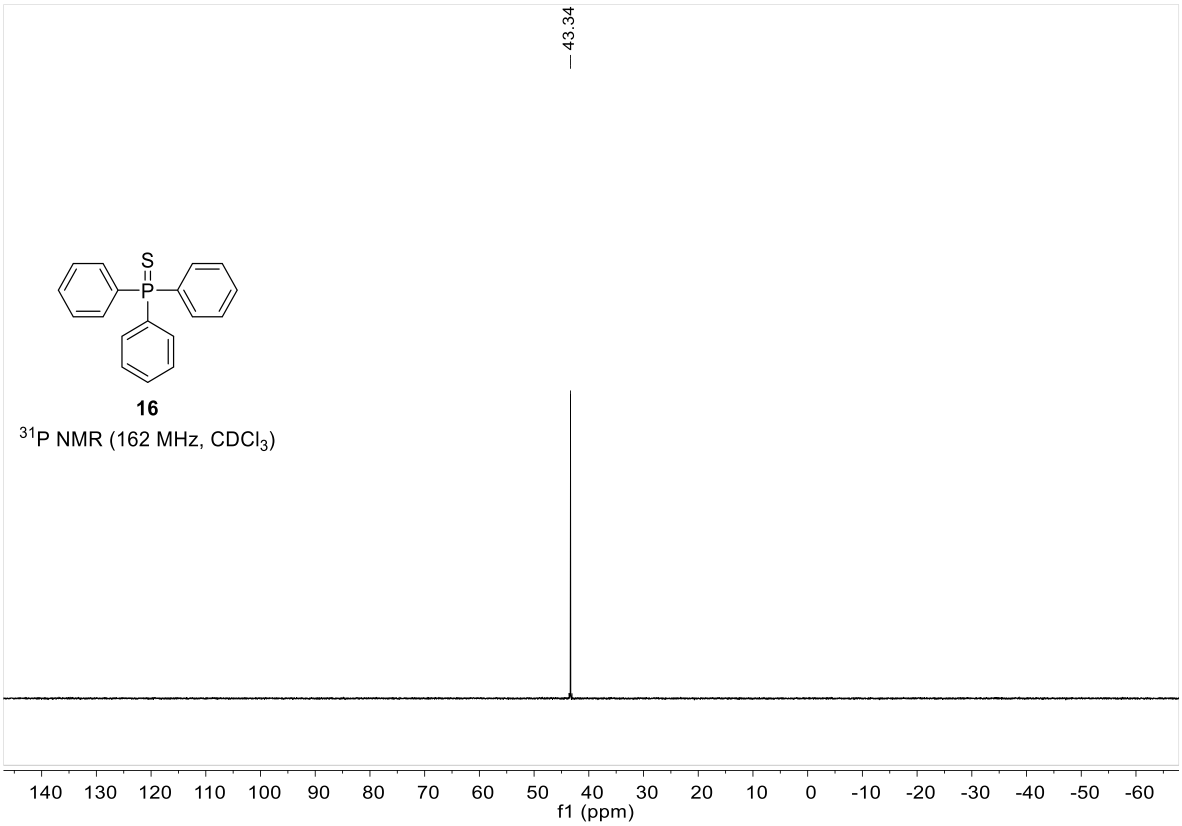

Supplementary Figure 147. ^{31}P NMR spectra of compound **16** (162 MHz, $CDCl_3$)

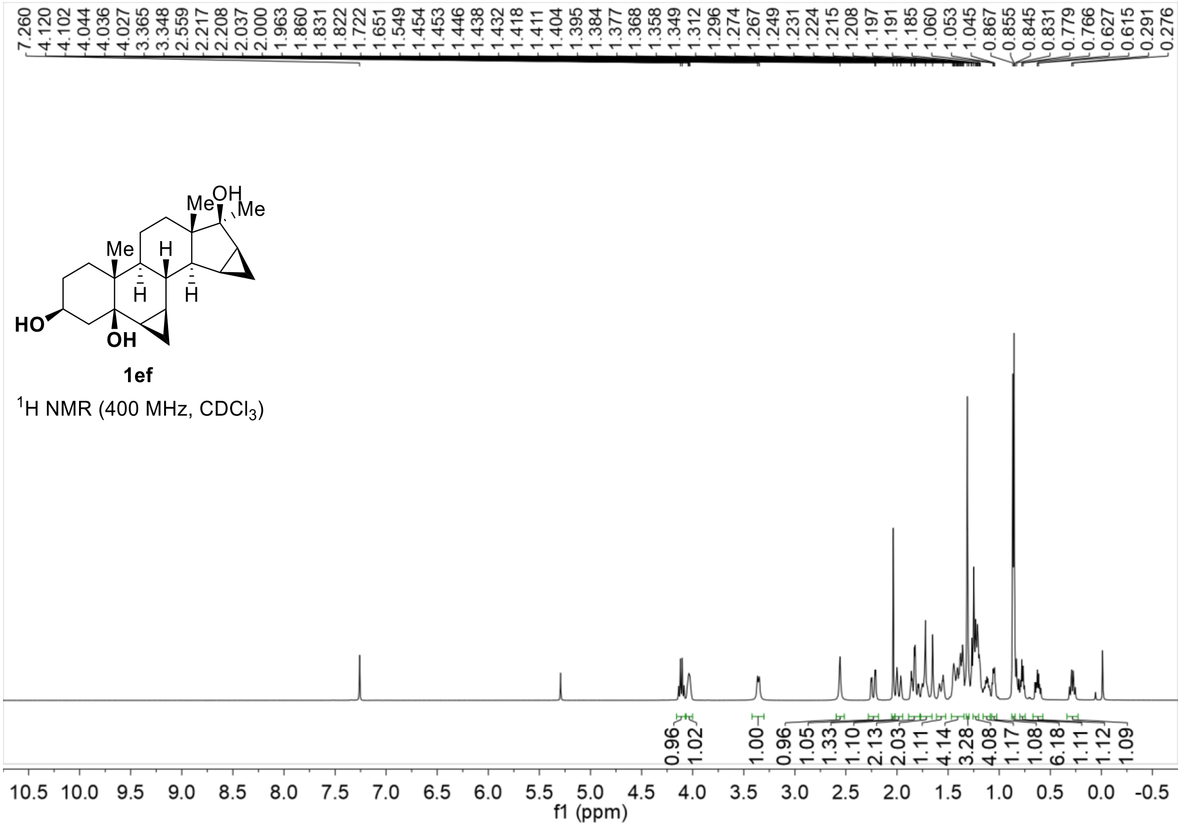

Supplementary Figure 148. ^{1}H NMR spectra of compound **1ef** (400 MHz, $CDCl_3$)

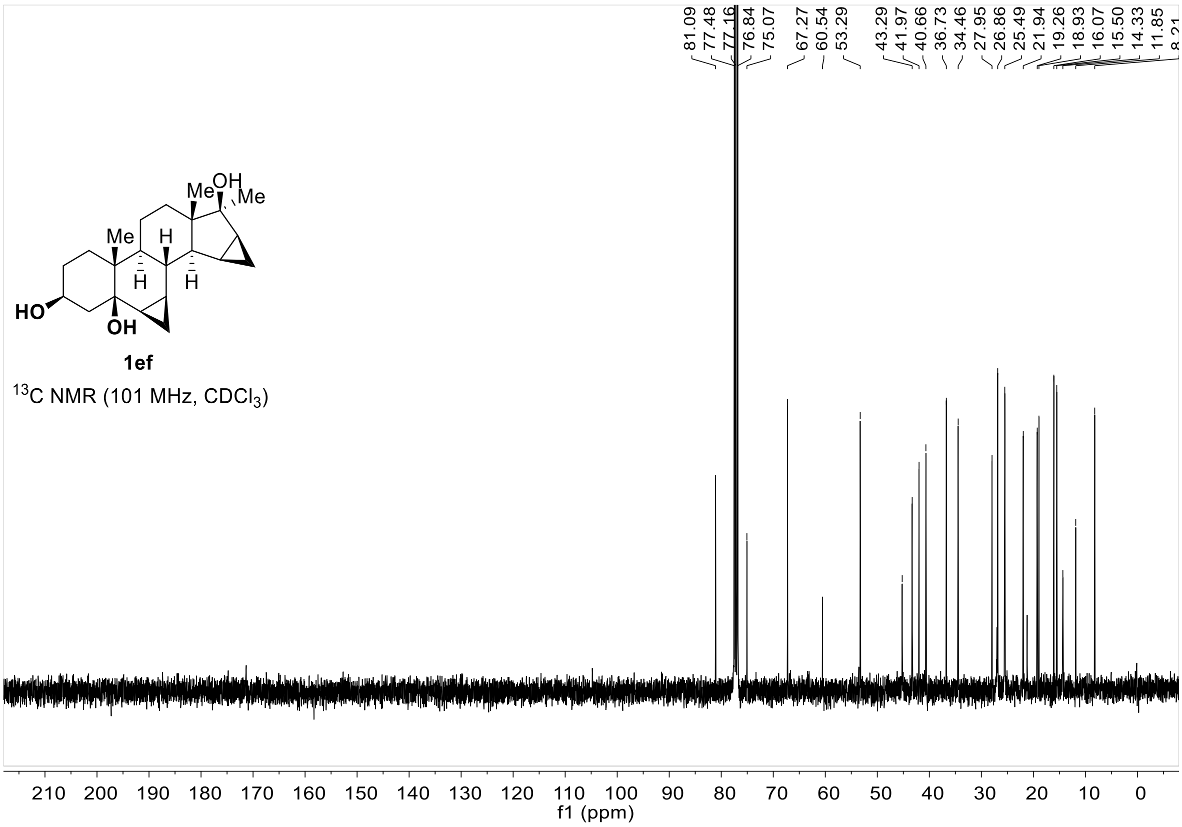

Supplementary Figure 149. ^{13}C NMR spectra of compound **1ef** (101 MHz, $CDCl_3$)

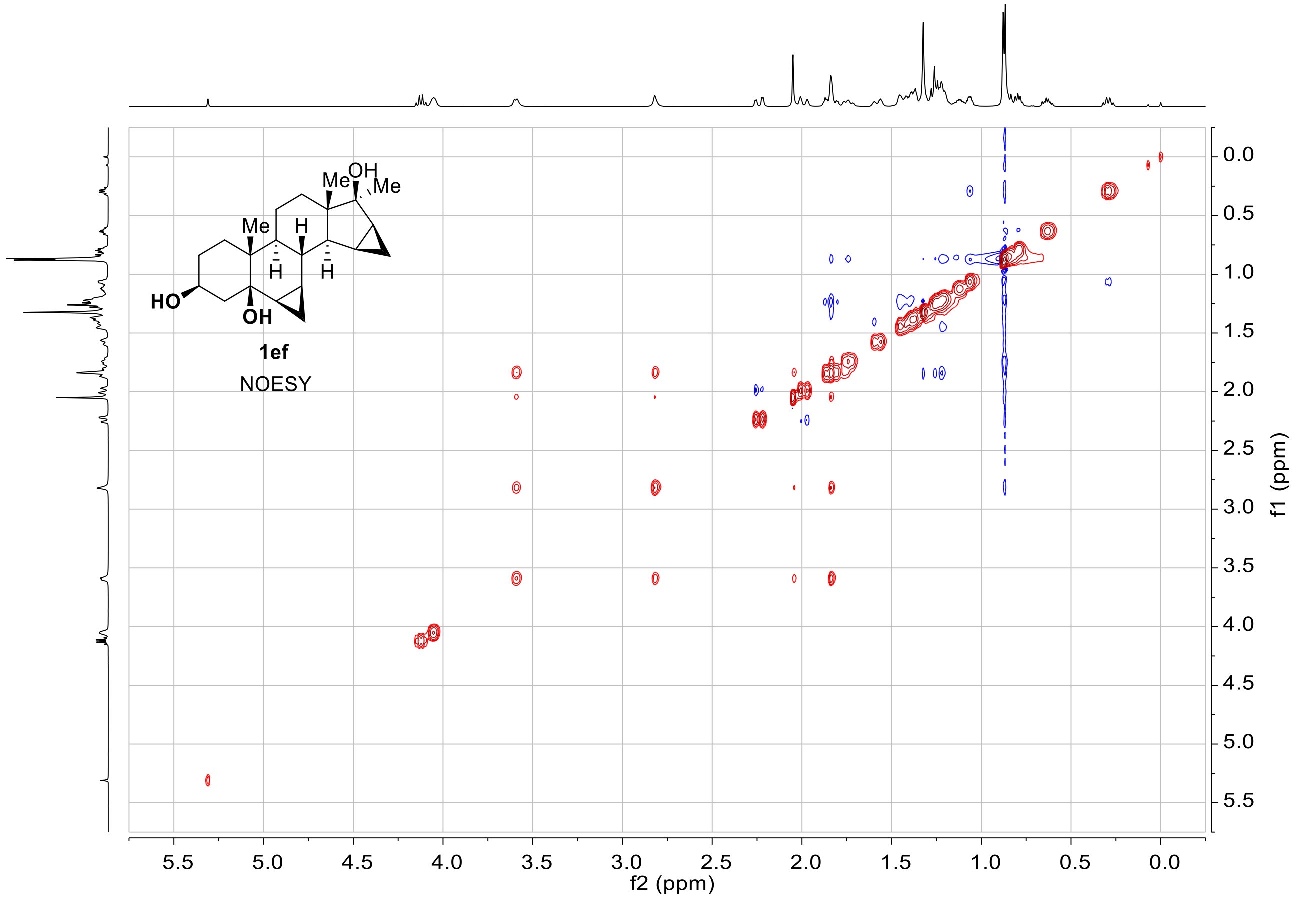

Supplementary Figure 150. NOESY spectra of compound **1ef**

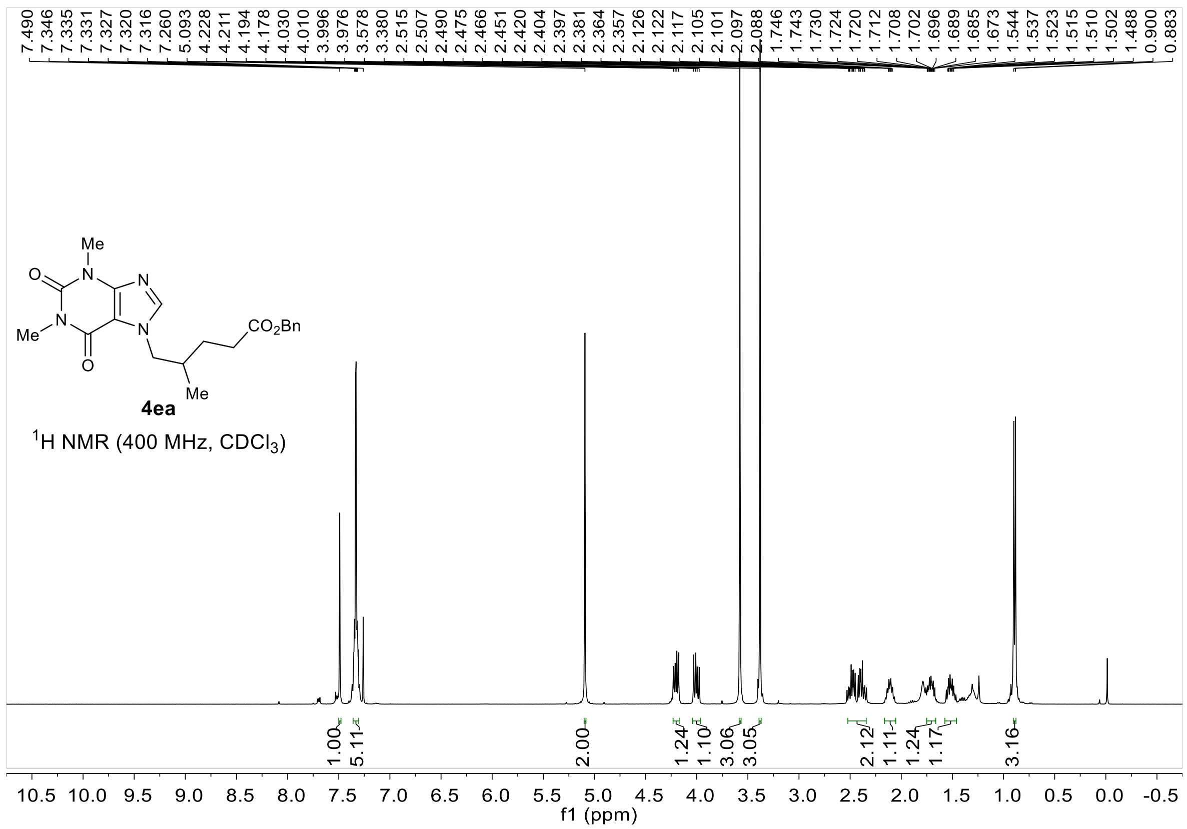

Supplementary Figure 151. ^{1}H NMR spectra of compound **4ea** (400 MHz, $CDCl_3$)

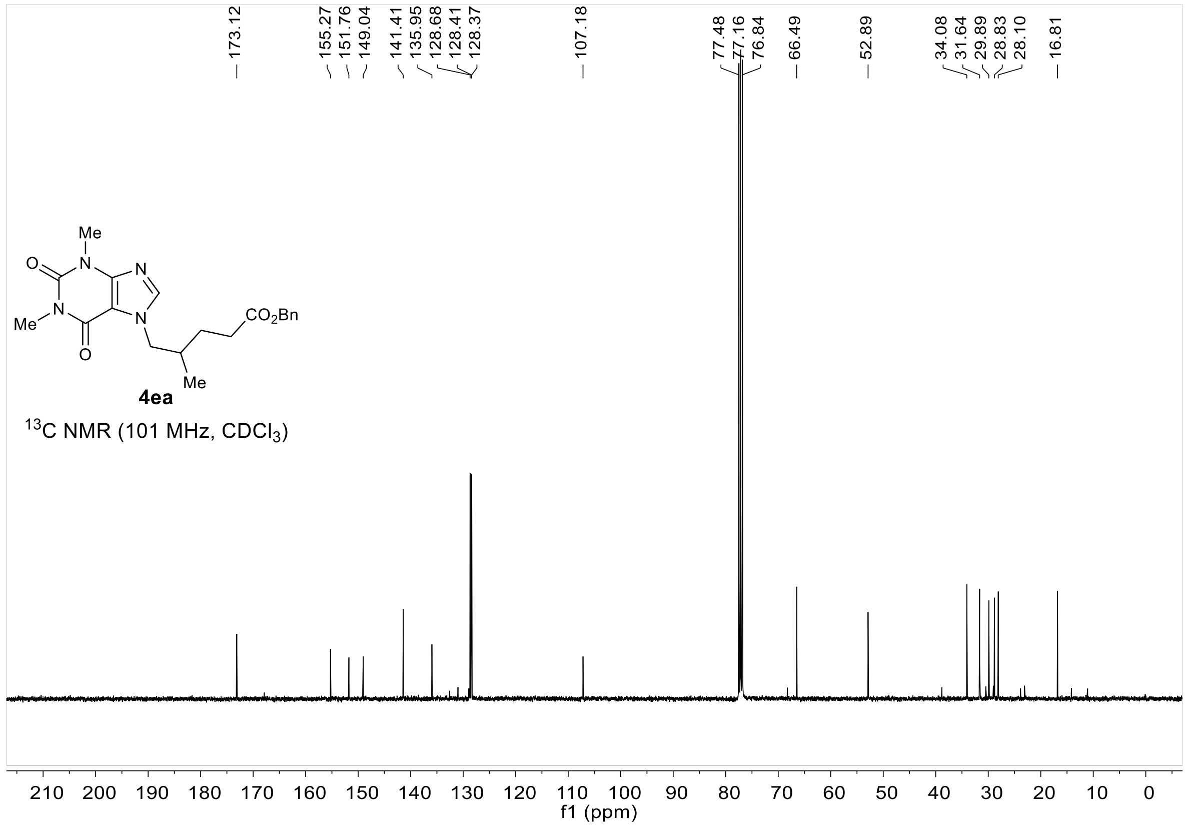

Supplementary Figure 152. ^{13}C NMR spectra of compound **4ea** (101 MHz, $CDCl_3$)

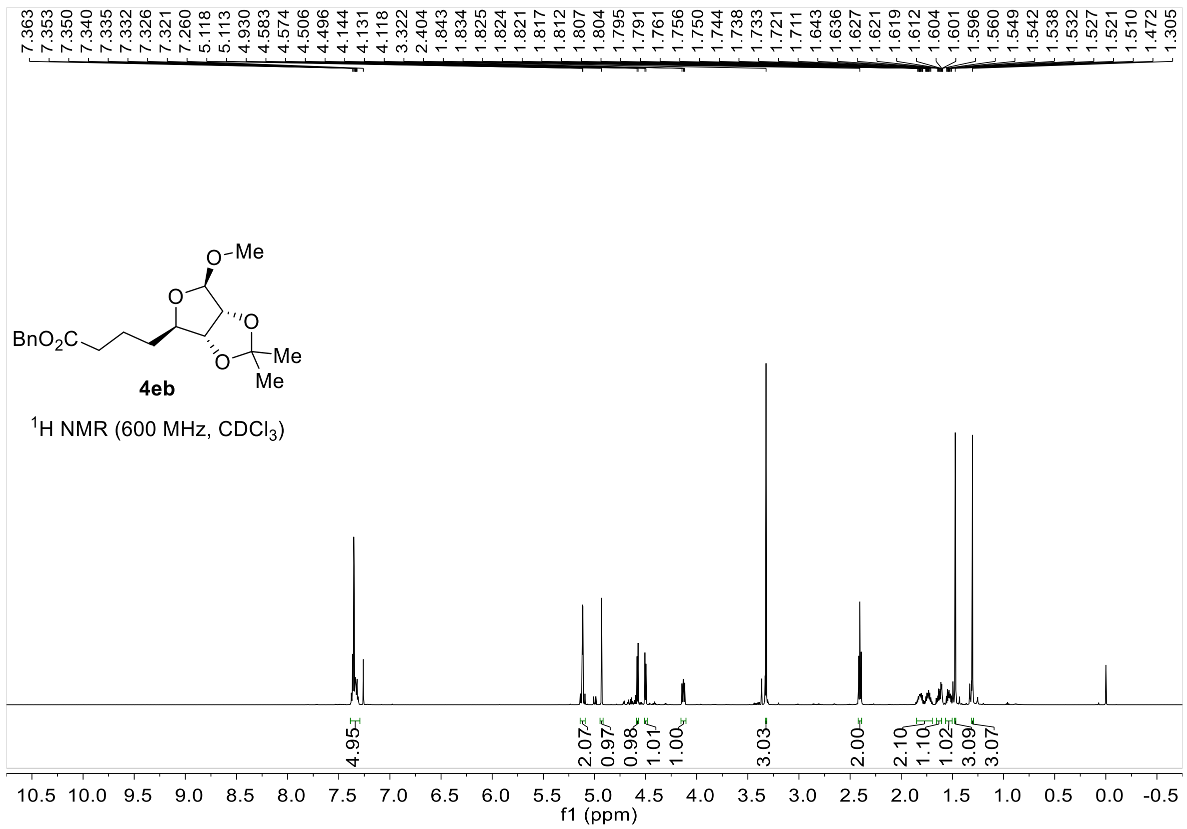

Supplementary Figure 153. ^{1}H NMR spectra of compound **4eb** (600 MHz, $CDCl_3$)

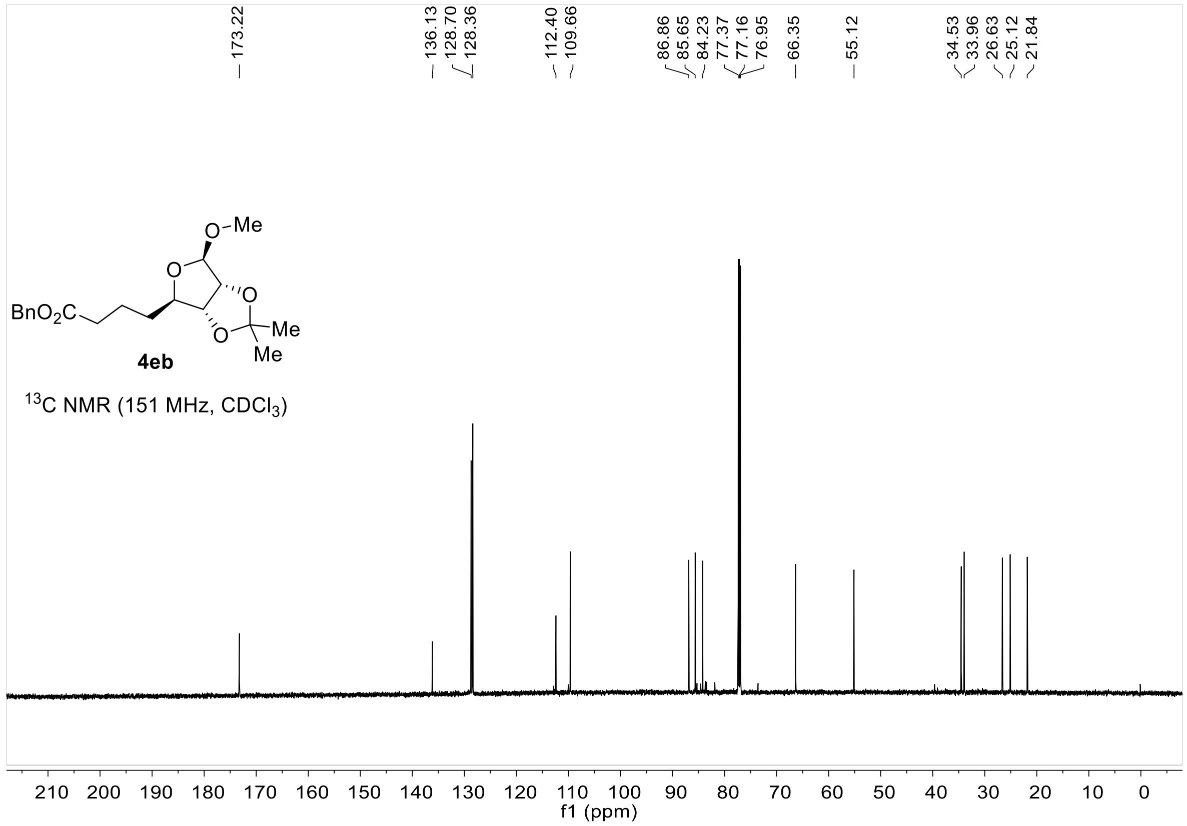

Supplementary Figure 154. ^{13}C NMR spectra of compound **4eb** (151 MHz, $CDCl_3$)

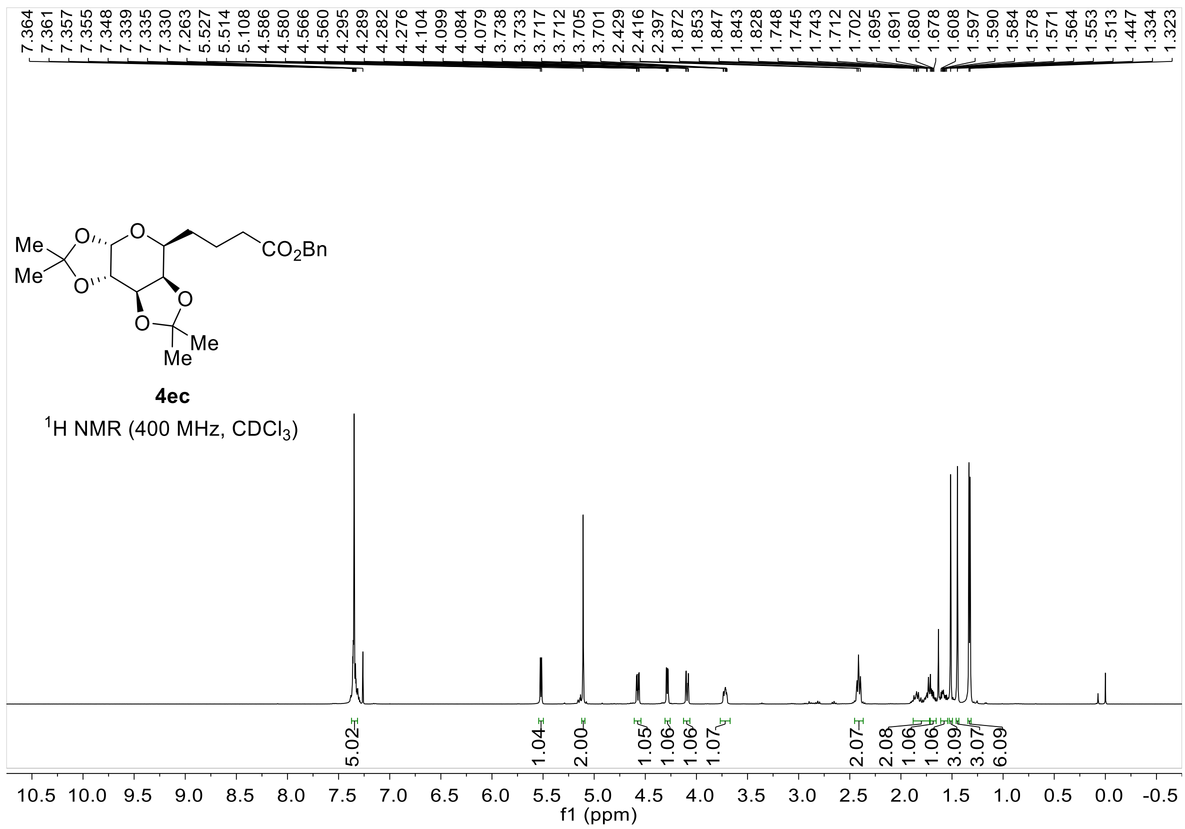

Supplementary Figure 155. ^{1}H NMR spectra of compound **4ec** (400 MHz, $CDCl_3$)

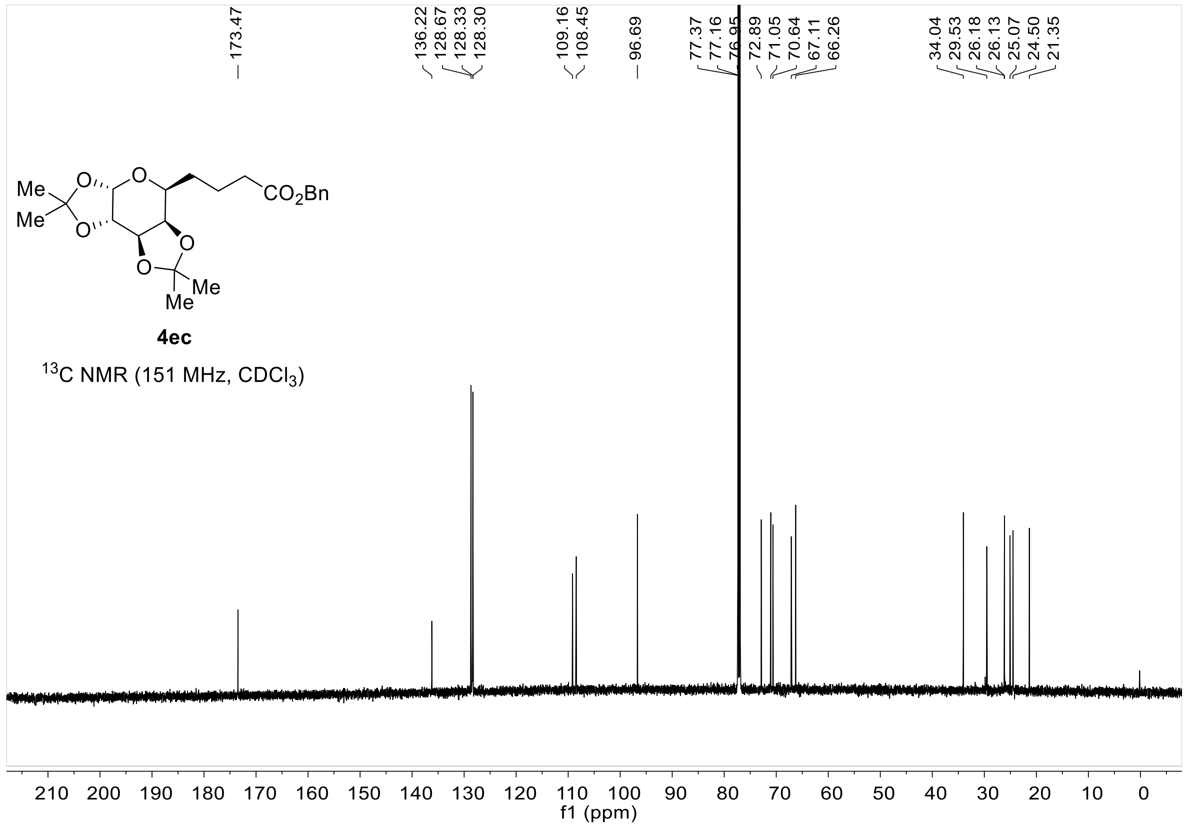

Supplementary Figure 156. ^{13}C NMR spectra of compound **4ec** (151 MHz, $CDCl_3$)

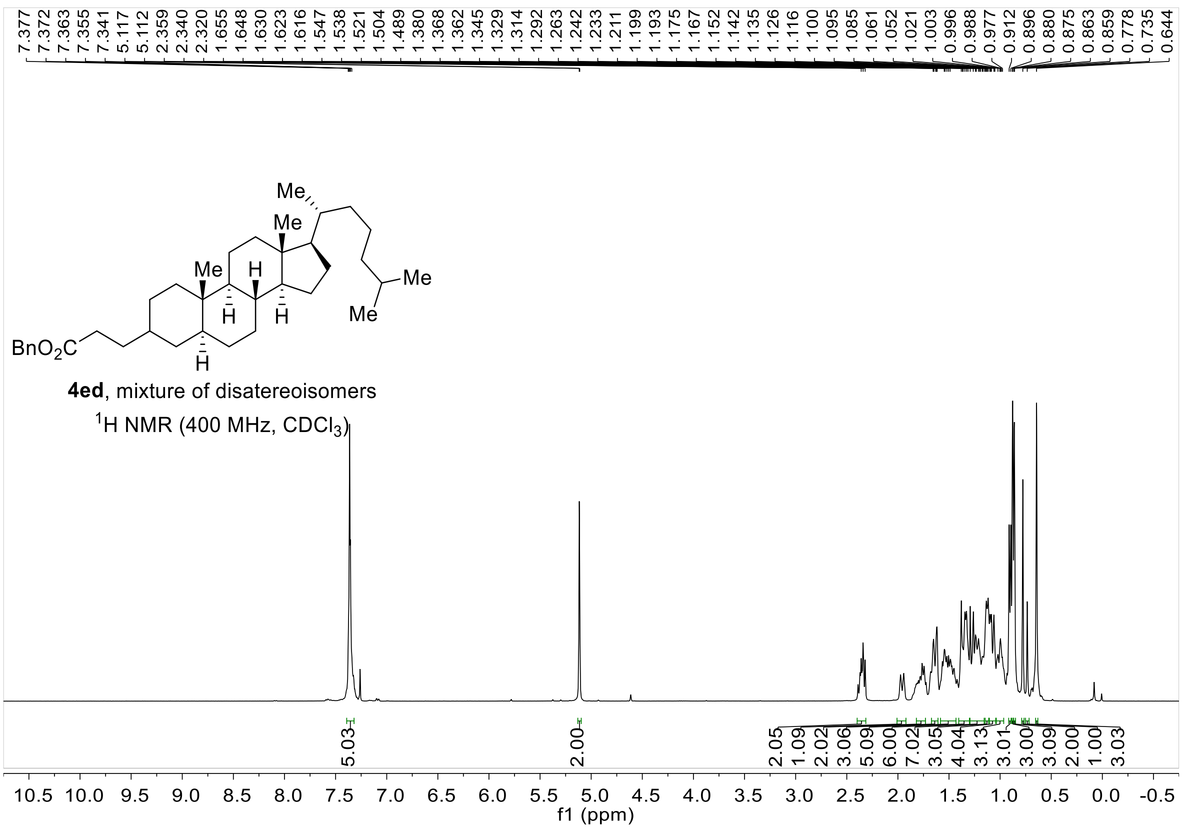

Supplementary Figure 157. ^{1}H NMR spectra of compound **4ed** (400 MHz, $CDCl_3$)

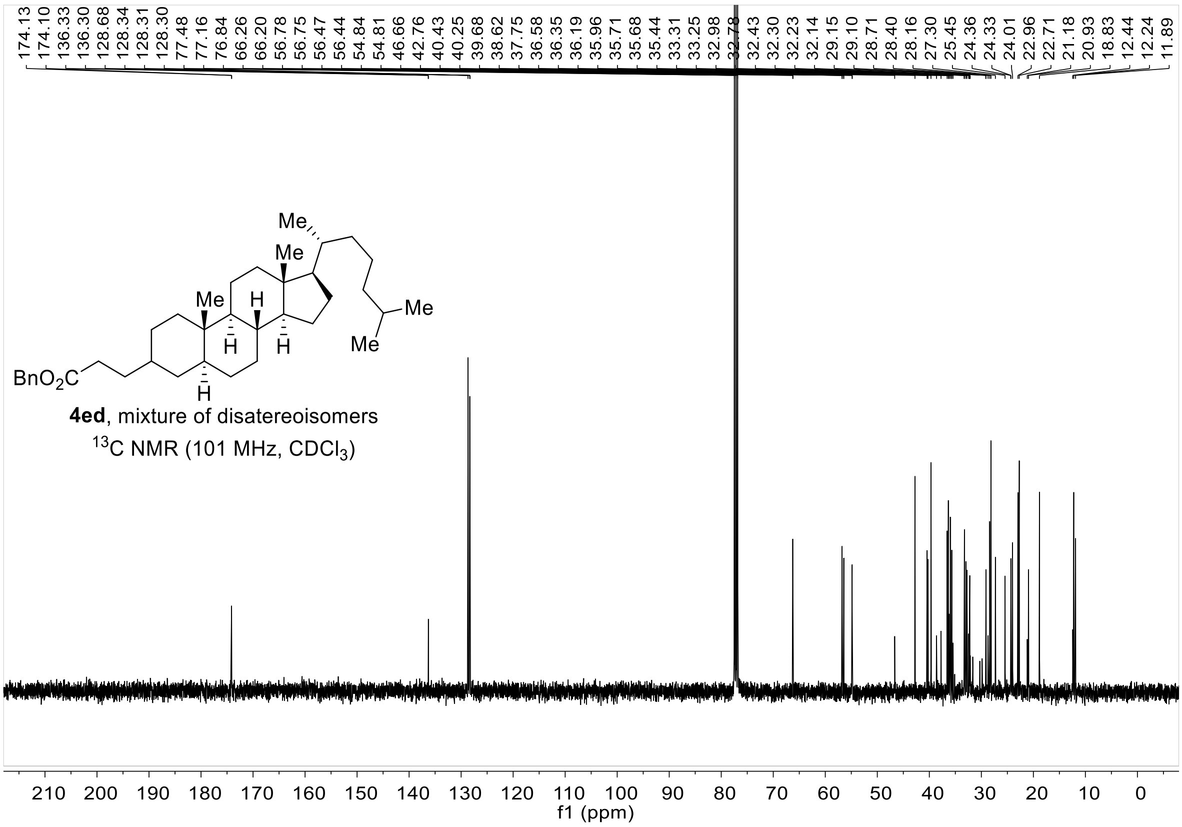

Supplementary Figure 158. ^{13}C NMR spectra of compound **4ed** (101 MHz, $CDCl_3$)

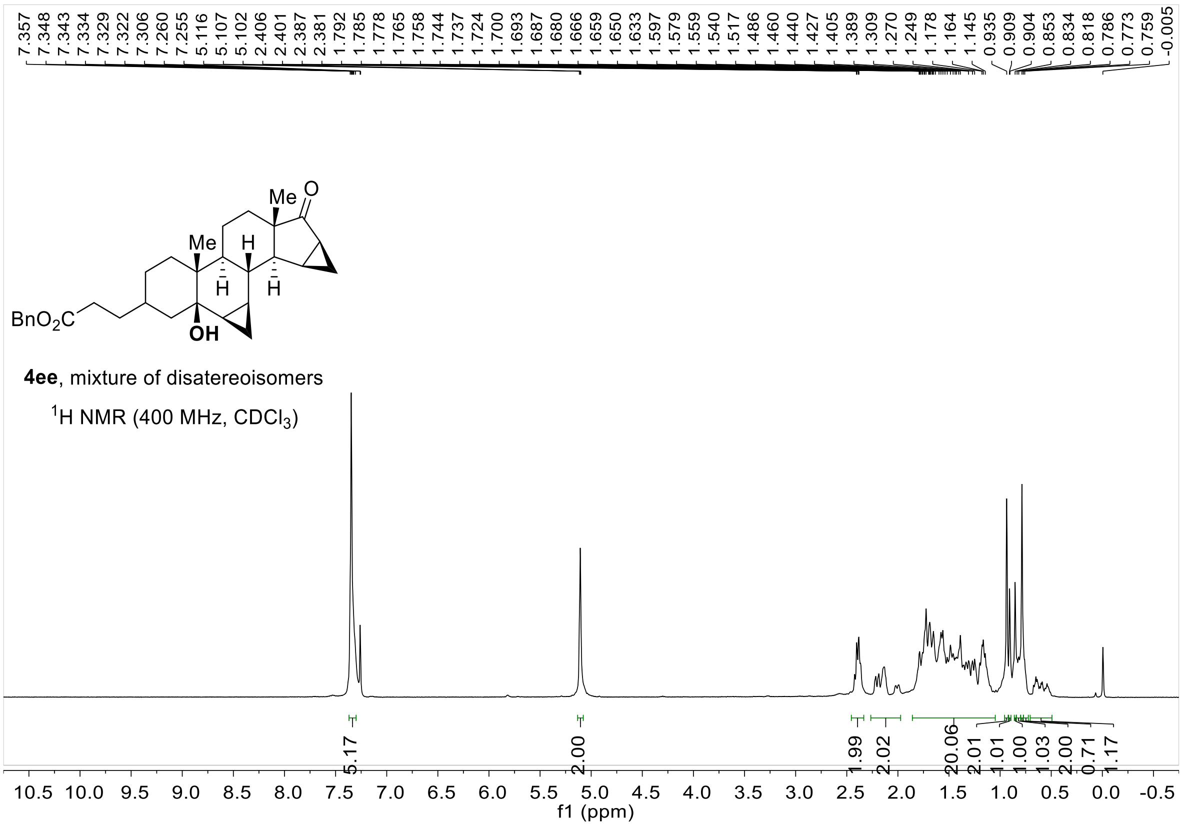

Supplementary Figure 159. ^{1}H NMR spectra of compound **4ee** (400 MHz, $CDCl_3$)

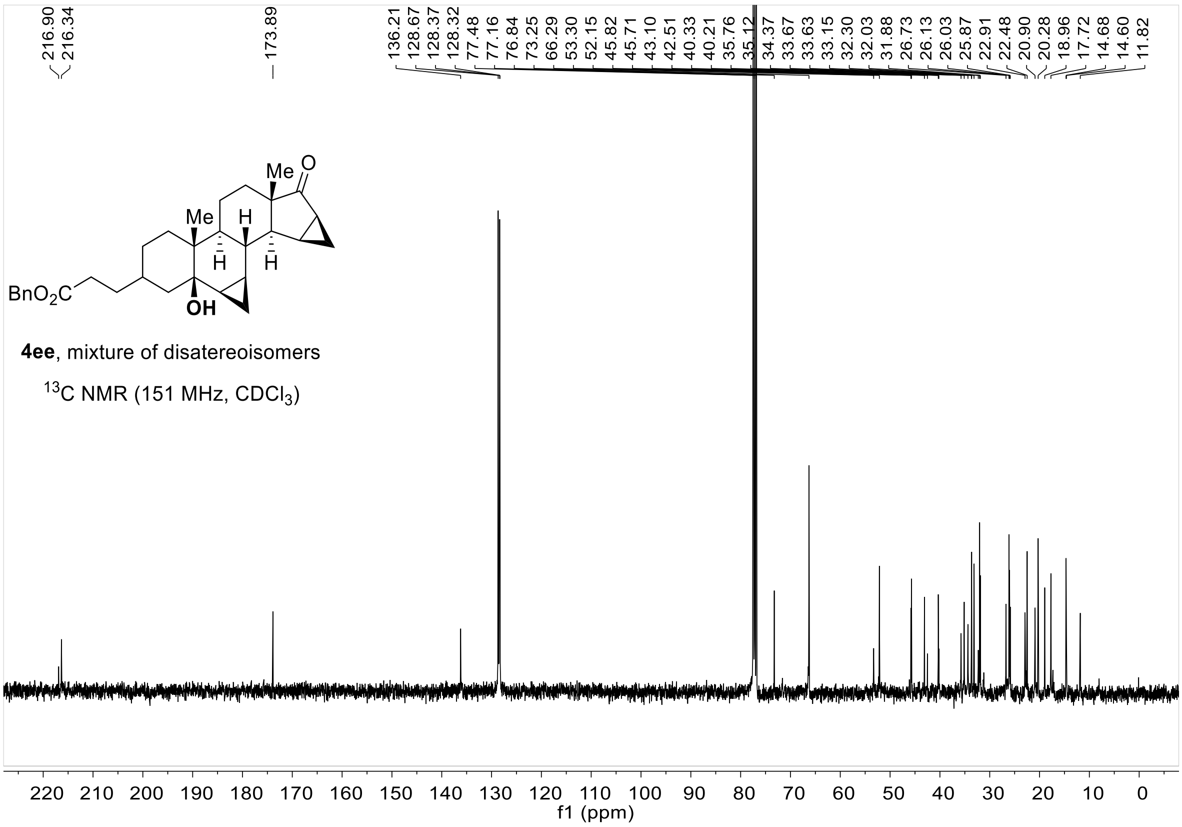

Supplementary Figure 160. ^{13}C NMR spectra of compound **4ee** (151 MHz, $CDCl_3$)

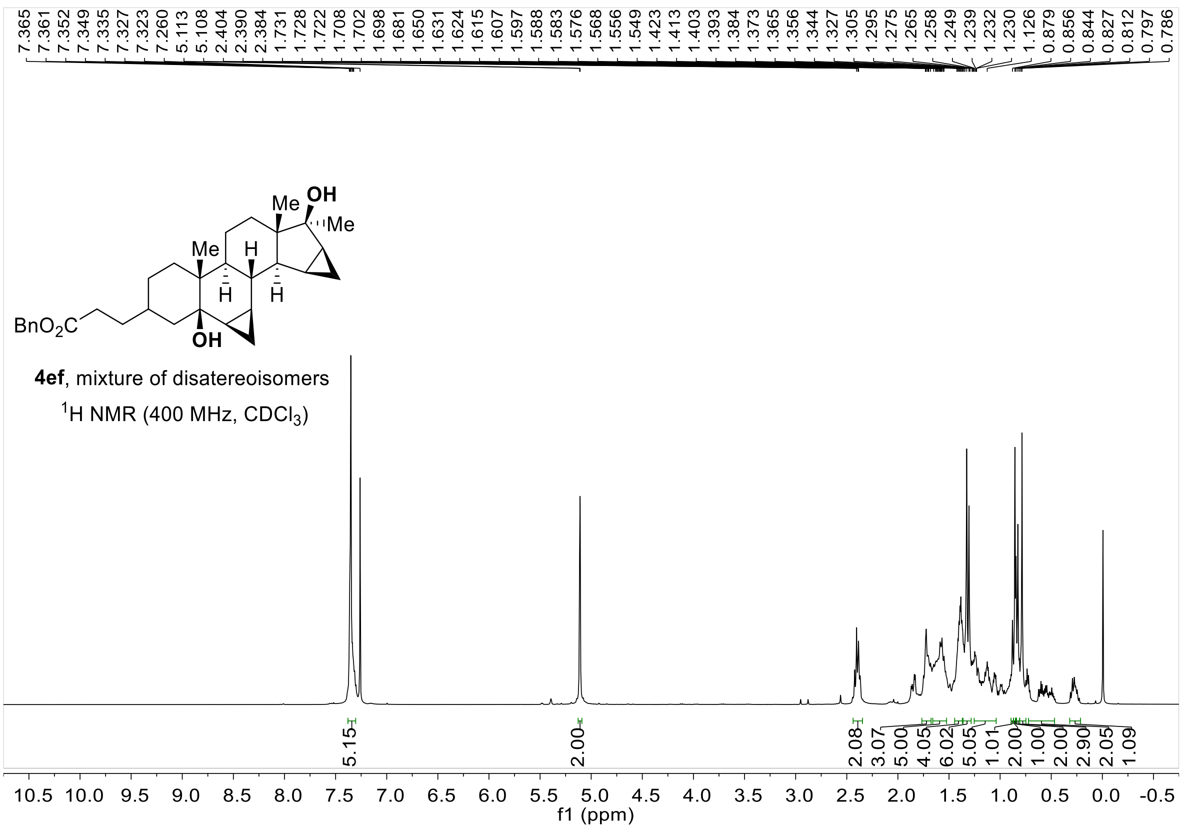

Supplementary Figure 161. ^{1}H NMR spectra of compound **4ef** (400 MHz, $CDCl_3$)

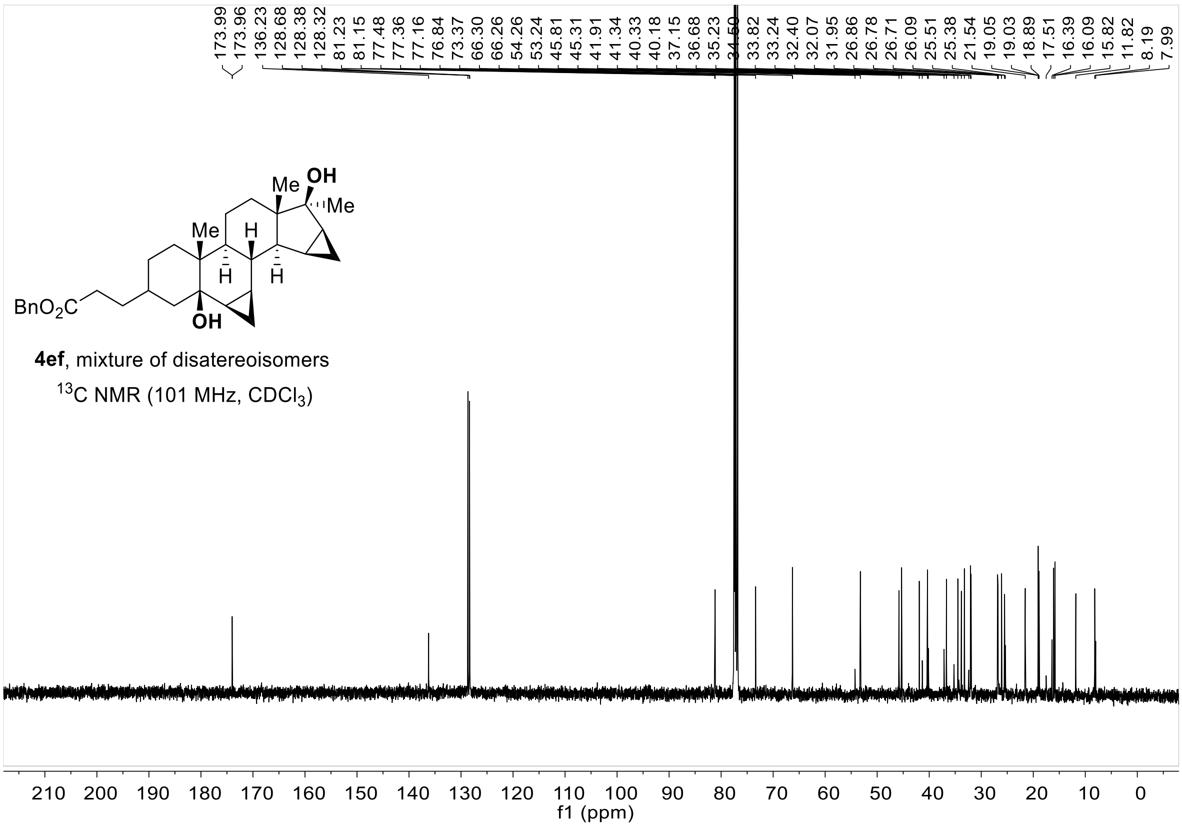

Supplementary Figure 162. ^{13}C NMR spectra of compound **4ef** (101 MHz, $CDCl_3$)

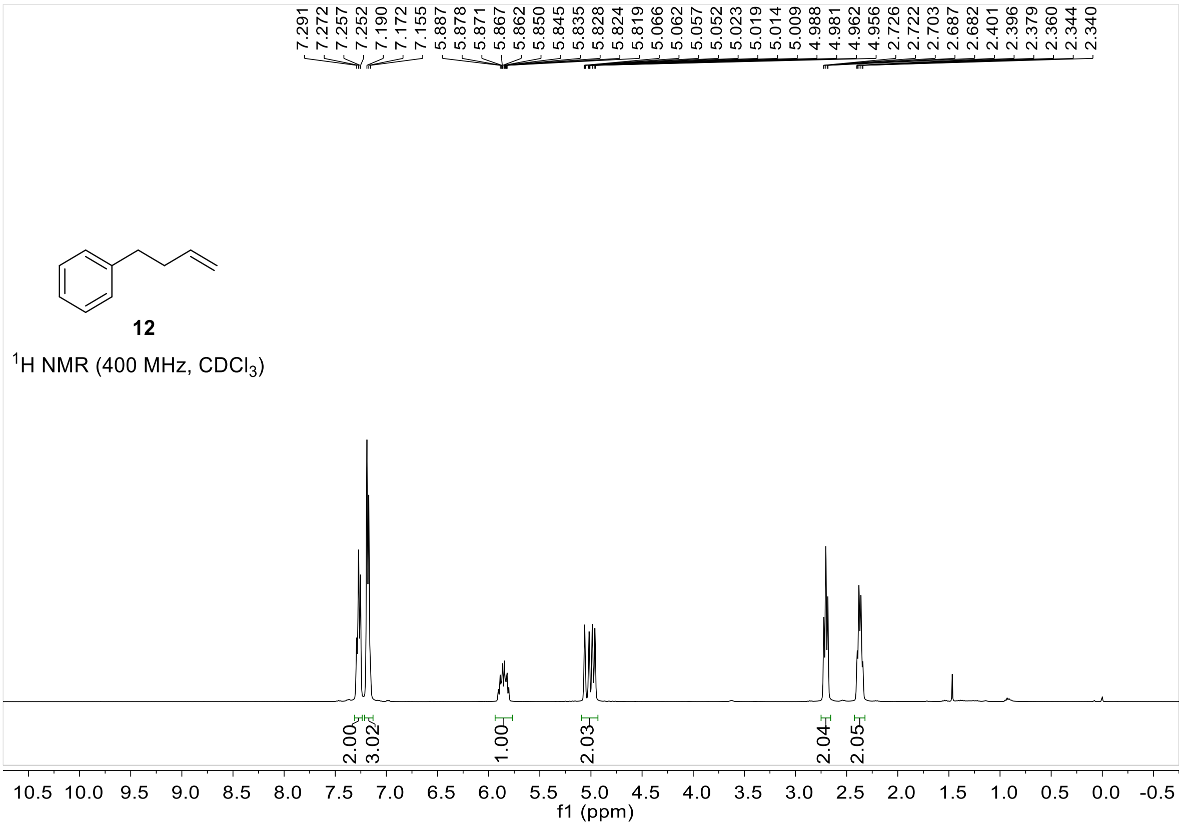

Supplementary Figure 163. ^{1}H NMR spectra of compound **12** (400 MHz, $CDCl_3$)

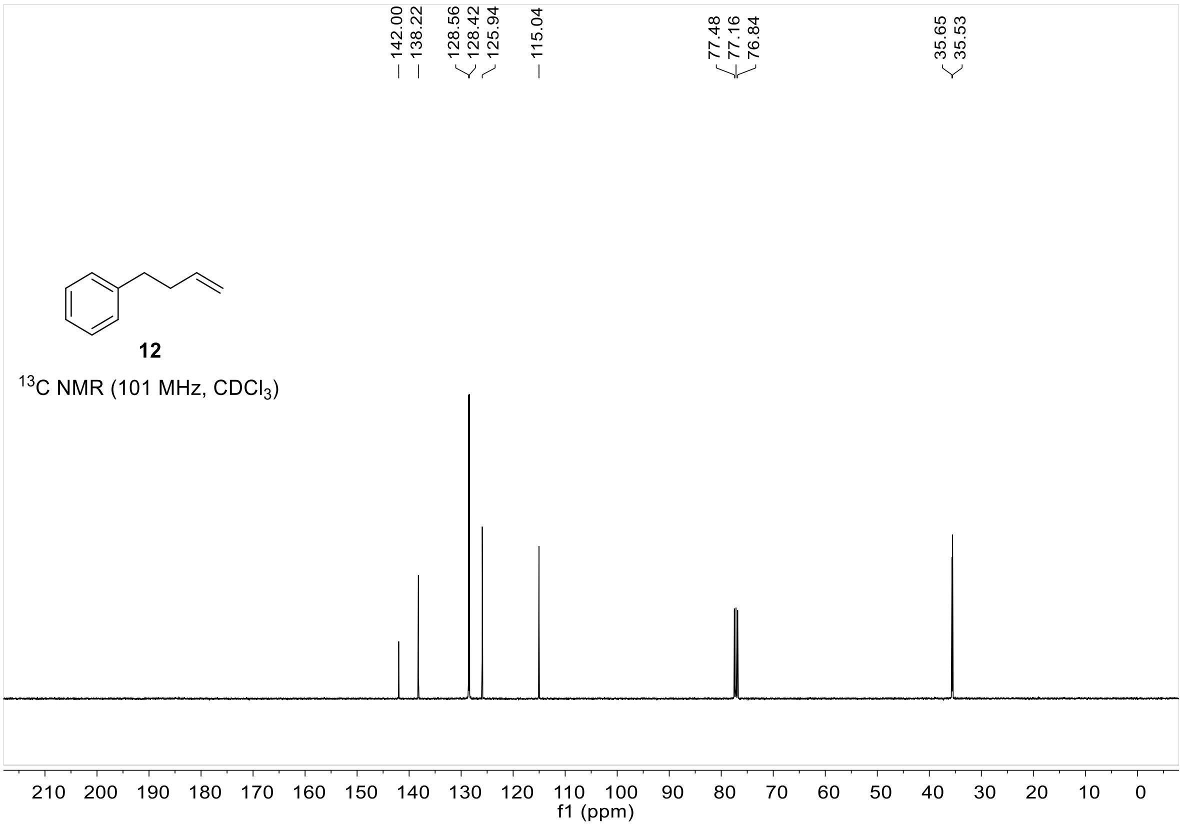

Supplementary Figure 164. ^{13}C NMR spectra of compound **12** (101 MHz, $CDCl_3$)

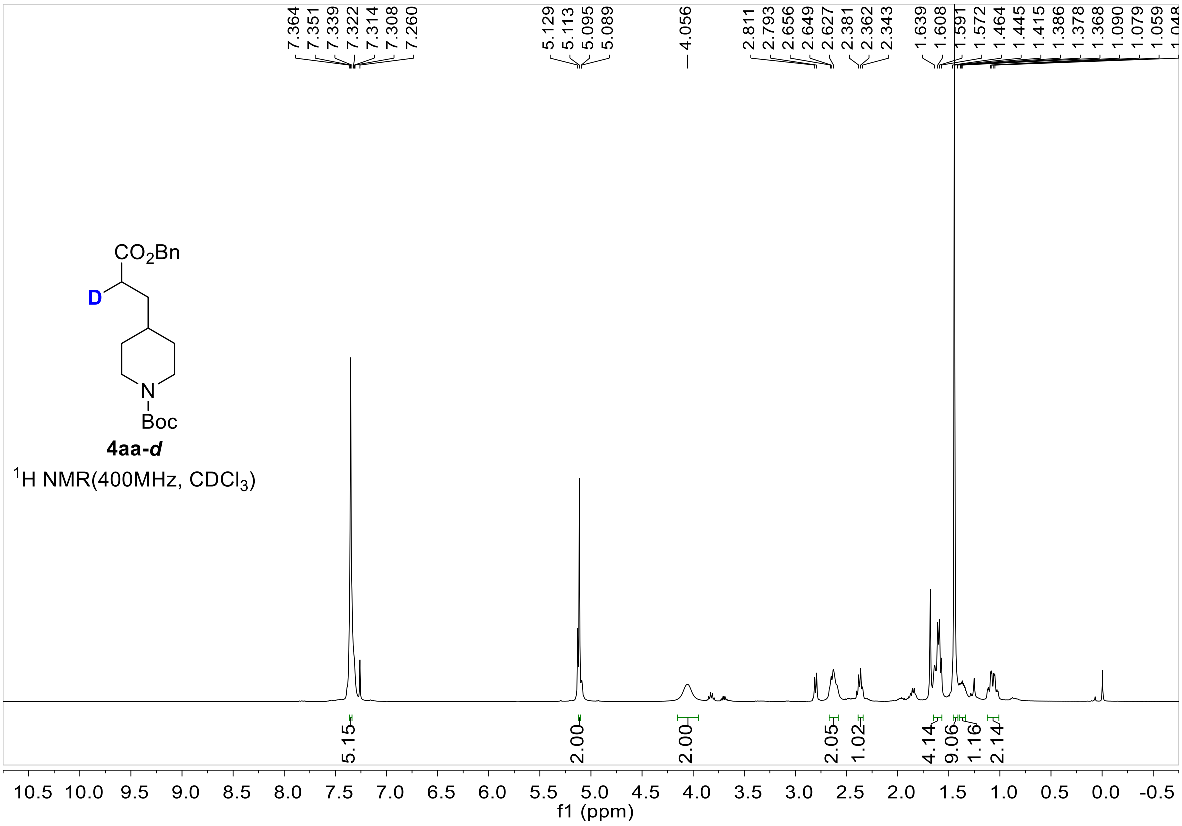

Supplementary Figure 165. ^{1}H NMR spectra of compound **4aa-*d*** (400 MHz, $CDCl_3$)

8. Supplementary References

(1) Carta, F.; Akdemir, A.; Scozzafava, A.; Masini, E.; Supuran, C. T. Xanthates and trithiocarbonates strongly inhibit carbonic anhydrases and show antiglaucoma effects in vivo. *J. Med. Chem.* **56**, 4691–4700 (2013).

(2) Dong, J.; Wang, X.; Wang, Z.; Song, H.; Liu, Y.; Wang, Q. Visible-light-initiated manganese-catalyzed giese addition of unactivated alkyl iodides to electron-poor olefins. *Chem. Commun.* **55**, 11707–11710 (2019).

(3) Wang, Y.; Li, G.-X.; Yang, G.; He, G.; Chen, G. A visible-light-promoted radical reaction system for azidation and halogenation of tertiary aliphatic C–H bonds. *Chem. Sci.* **7**, 2679–2683 (2016).

(4) Lopp, J. M.; Schmidt, V. A. Intermolecular phosphite-mediated radical desulfurative alkene alkylation using thiols. *Org. lett.* **21**, 8031–8036 (2019).

(5) Ye, Y.; Chen, H.; Sessler, J. L.; Gong, H. Zn-mediated fragmentation of tertiary alkyl oxalates enabling formation of alkylated and arylated quaternary carbon centers. *J. Am. Chem. Soc.* **141**, 820–824 (2019).

(6) Devlin, R.; Jones, D. J.; McGlacken, G. P. One-pot, tandem wittig hydrogenation: formal C(sp^3)–C(sp^3) bond formation with extensive scope. *Org. lett.* **22**, 5223–5228 (2020).

(7) Hayashi, M.; Matsuura, T.; Tanaka, I.; Ohta, H.; Watanabe, Y. Pd-catalyzed P–C cross-coupling reactions for versatile triarylphosphine synthesis. *Org. lett.* **15**, 628–631 (2013).

(8) Speck, K.; Karaghiosoff, K.; Magauer, T. Sequential O–H/C–H bond insertion of phenols initiated by the gold(I)-catalyzed cyclization of 1-bromo-1,5-enynes. *Org. lett.* **17**, 1982–1985 (2015).

(9) Slinker, J. D.; Gorodetsky, A. A.; Lowry, M. S.; Wang, J.; Parker, S.; Rohl, R.; Bernhard, S.; Malliaras, G. G. Efficient yellow electroluminescence from a single layer of a cyclometalated iridium complex. *J. Am. Chem. Soc.* **126**, 2763–2767 (2004).

(10) Panferova, L. I.; Zubkov, M. O.; Kokorekin, V. A.; Levin, V. V.; Dilman, A. D. Using the thiyl radical for aliphatic hydrogen-atom transfer: thiolation of unactivated C–H bonds. *Angew. Chem. Int. Ed.* **60**, 2849–2854 (2021).
